# CO reductive oligomerization by a divalent thulium complex and CO_2_-induced functionalization†

**DOI:** 10.1039/d2sc01798a

**Published:** 2022-05-09

**Authors:** Thomas Simler, Karl N. McCabe, Laurent Maron, Grégory Nocton

**Affiliations:** LCM, CNRS, Ecole Polytechnique, Institut Polytechnique de Paris, Route de Saclay Palaiseau 91120 France gregory.nocton@polytechnique.edu thomas.simler@polytechnique.edu; LPCNO, UMR 5215, Université de Toulouse-CNRS, INSA, UPS Toulouse France

## Abstract

The divalent thulium complex [Tm(Cp^ttt^)_2_] (Cp^ttt^ = 1,2,4-tris(*tert*-butyl)cyclopentadienyl) reacts with CO to afford selective CO reductive dimerization and trimerization into ethynediolate (C_2_) and ketenecarboxylate (C_3_) complexes, respectively. DFT calculations were performed to shed light on the elementary steps of CO homologation and support a stepwise chain growth. The attempted decoordination of the ethynediolate fragment by treatment with Me_3_SiI led to dimerization and rearrangement into a 3,4-dihydroxyfuran-2-one complex. Investigation of the reactivity of the C_2_ and C_3_ complexes towards other electrophiles led to unusual functionalization reactions: while the reaction of the ketenecarboxylate C_3_ complex with electrophiles yielded new multicarbon oxygenated complexes, the addition of CO_2_ to the ethynediolate C_2_ complex resulted in the formation of a very reactive intermediate, allowing C–H activation of aromatic solvents. This original intermolecular reactivity corresponds to an unprecedented functionalization of CO-derived ligands, which is induced by CO_2_.

## Abstract 2

The divalent thulium complex [Tm(Cp^ttt^)_2_] activates CO to form reductive CO dimerization or trimerization products. These complexes further react with electrophiles, including CO_2_, yielding multicarbon oxygenates and original C–H activation products.

## Graphical abstract

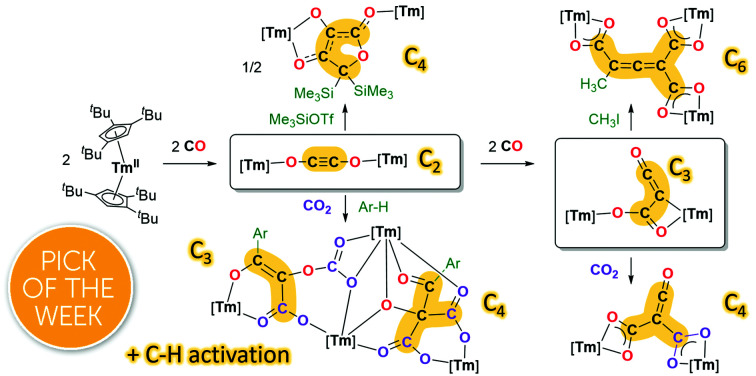

## Introduction

Owing to environmental and economic reasons, the transformation and valorization of abundant and readily available chemical feedstock such as CO and CO_2_ are very topical goals.^1^ Carbon monoxide is a C_1_ gas that is used in the industrial production of organic feedstock molecules such as methanol^2^ and acetic acid.^3^ Synthesis gas (syn-gas: CO/H_2_) is converted into liquid hydrocarbons and oxygenated molecules on industrial scales by using the Fischer–Tropsch (F–T) process.^4^ As the C≡O triple bond is one of the strongest chemical bonds (bond dissociation energy of 1075 kJ mol^−1^),^5^ this process typically operates under harsh temperature and pressure conditions (200–350 °C, 20–45 bars) using heterogeneous transition metal catalysts (Fe, Co).^6^ The precise mechanism in the formation of F–T products is still under debate and, to gain more insight into the elementary steps involved, several organometallic complexes have been used as soluble models.^7^ Besides, recent advances in transition-metal-free F–T chemistry have been reported, describing the formation of growing carbon chains.^8^

**Fig. 1:**
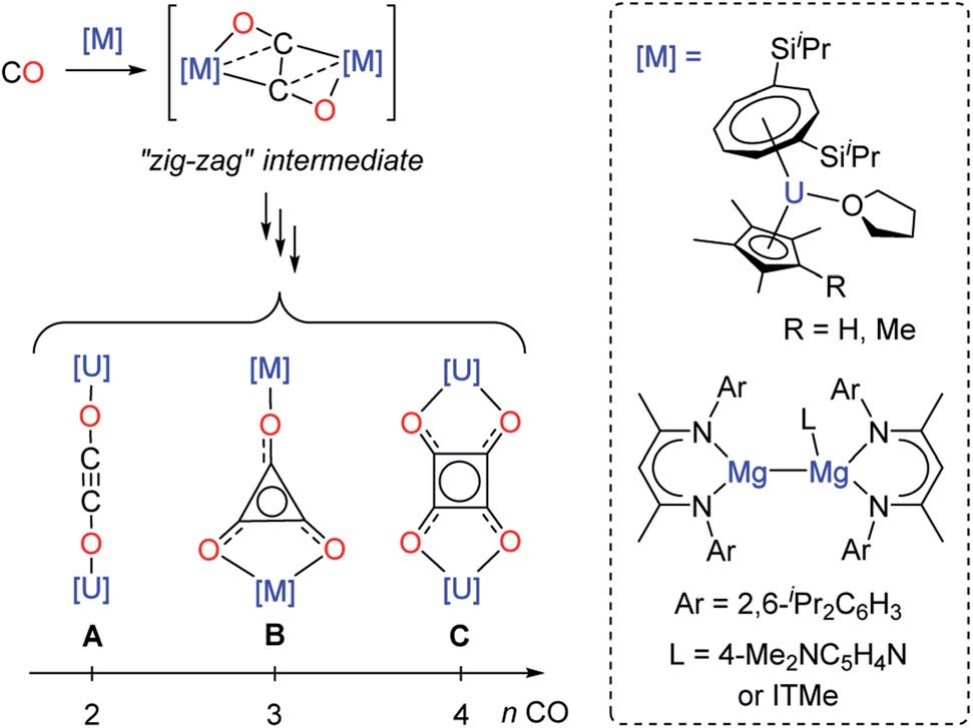
Evolution of the “zig-zag” intermediate into CO oligomerization products upon reaction of selected U(iii) and Mg(i) complexes with CO (ITMe = 1,3,4,5-tetramethylimidazol-2-ylidene).

Although CO homologation has been observed by the coupling between two or more fragments upon insertion of CO into metal–alkyl,^9^ –aryl,^10^ –hydride,^11^ –imide^12^ or –boryl bonds,^13^ the direct reductive coupling of CO molecules has only been achieved by a very limited number of systems, typically involving low-valent oxophilic—metallic or non-metallic—elements.^14^ For example, homogeneous p-,^15^ and d-block compounds^16^ have been used to promote CO reductive coupling. Molecular low-valent f-block complexes based on U(iii) and Ln(ii) metal centers have shown very promising reactivity in the reductive coupling of CO, which can be traced back to their high reducing character and oxophilic nature.^16*e*,17^ Detailed experimental^17*d*–*f*,17*j*^ and computational studies^17*f*,18^ have been performed by Cloke, Green, Maron and co-workers in order to rationalize the CO reductive coupling reactivity by bulky U(iii) sandwich complexes. Depending on the reaction conditions and supporting ligands, selective formation of oxocarbon dianions of the type ethynediolate (A), deltate (B) or squarate (C) was reported (Fig. 1).

In these examples, DFT calculations pointed to a “zig-zag” intermediate complex featuring a bridging *trans*-bent [C_2_O_2_]^2−^ dianion as a key intermediate in the formation of the different species. A similar intermediate was also recently suggested by Jones and co-workers in the reductive coupling of CO using activated β-diketiminate Mg(i) complexes (Fig. 1).^15*e*,*g*^ However, typically, the reactivity of the CO oligomerization products was not further explored. Very recently, Crimmin and co-workers described the sequential formation of C_1_–C_4_ chain compounds starting from CO and CO_2_ feedstocks in the presence of transition metal carbonyl complexes and using β-diketiminate Al(i) reductants.^15*c*,*k*^ Other systems allowing sequential chain growth by reductive homologation of CO from isolable intermediates or functionalization reactions of the products are exceedingly rare.^15*a*,*l*^

We have been interested in the use of highly reducing Tm(ii)^19^ and other divalent lanthanide complexes for homogeneous transformations and small molecule activation.^20^ Herein, we report the reactivity of the Tm(ii) complex [Tm(Cp^ttt^)_2_]^21^ (Cp^ttt^ = 1,2,4-tris(*tert*-butyl)cyclopentadienyl) towards CO, leading to selective CO reductive di- and trimerization products. The reactivity of the corresponding complexes, which feature central dianionic {C_*n*_O_*n*_}^2−^ (*n* = 2,3) oxocarbon moieties, was systematically investigated towards electrophiles (CO_2_, silylating/alkylating agents). Novel multicarbon oxygenated frameworks were obtained through unusual reactivities, opening new avenues for the functionalization of CO and CO_2_.

## Results and discussion

### Synthesis and structure of 1

**Fig. 2:**
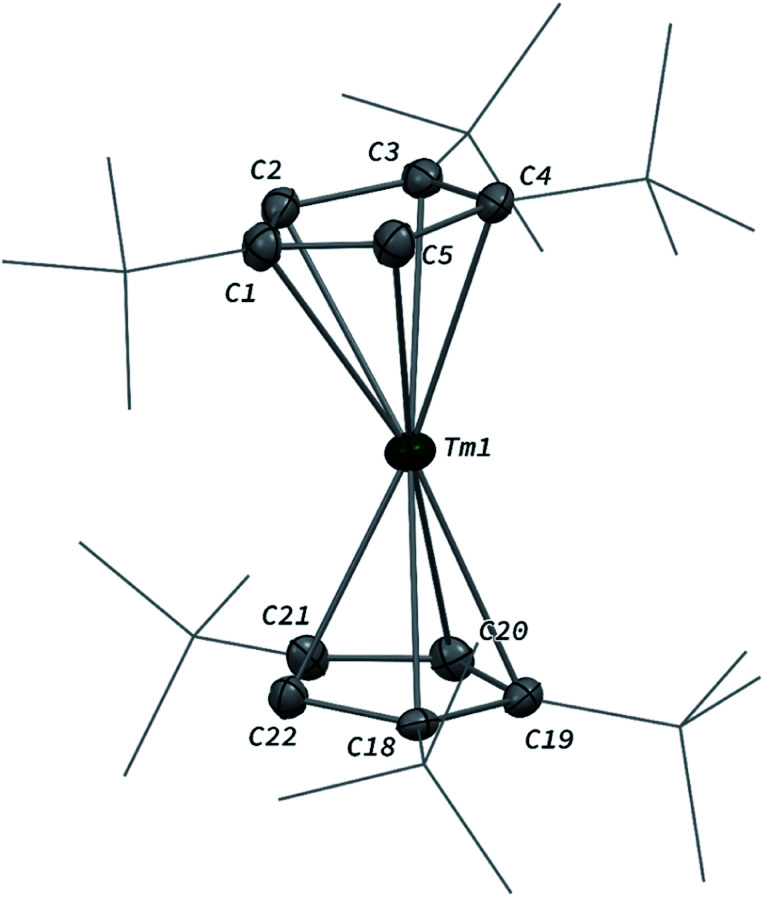
Molecular structure of one of the two independent molecules of 1 in the solid state with thermal ellipsoids at the 40% probability level (except for the ^*t*^Bu groups depicted in wireframe). Only one disordered position for the ^*t*^Bu groups has been depicted and H atoms have been omitted for clarity. Selected bond distances (Å): Tm1–C1 2.712(4), Tm1–C2 2.676(4), Tm1–C3 2.681(4), Tm1–C4 2.664(4), Tm1–C5 2.670(4), Tm1–C18 2.673(4), Tm1–C19 2.677(4), Tm1–C20 2.686(4), Tm1–C21 2.703(4), Tm1–C22 2.658(4).

The divalent thulium complex [Tm(Cp^ttt^)_2_] (1) was obtained by slightly modifying the original procedure by Nief and co-workers,^21^ in order to ensure better reproducibility and a higher isolated yield (for details, see ESI†). Crystals of 1 suitable for X-ray diffraction (XRD) studies could be successfully obtained from a concentrated pentane solution of the complex at low temperature (−40 °C). Owing to the very high solubility of 1 in hydrocarbon solvents, only the crystal structure of the less soluble THF adduct was reported in the seminal publication.^21*a*^ The molecular structure of 1 in the solid state (Fig. 2) confirmed the formation of the base-free complex with two independent molecules of 1 in the asymmetric unit, displaying very similar metrical data. In this divalent lanthanide sandwich complex, the Tm(ii) metal center is surrounded by two η^5^-coordinated Cp^ttt^ ligands with Tm–C bonds ranging from 2.652(4) to 2.717(4) Å (in average 2.68 ± 0.02 Å) and Tm–Cp(ctr) (ctr = ring centroid) distances of *ca.* 2.39 Å (see also Table S4†). In each crystallographically independent molecule, the sum of Tm–Cp(ctr) distances is *ca.* 4.78 Å, a value only slightly larger than that in the trivalent complex [Tm(Cp^ttt^)_2_I] (4.74 Å, see Table S4†).

Although the metal center in 1 is only surrounded by two Cp^ttt^ ligands, a slightly bent arrangement is observed in the solid state, with angles between the mean C_5_ planes in the range 11.0–13.9° and Cp(ctr)–Tm–Cp(ctr) angles of 164.3–167.7°. Similar bent arrangements were observed for the base-free complexes [Ln(Cp^ttt^)_2_] (Ln = Sm, Eu),^22^ although 1 presents a Cp(ctr)–Ln–Cp(ctr) arrangement closer to linearity, which may be the result of the smaller ionic radius of Tm(ii) compared to Sm(ii) (1.03 and 1.17 Å, respectively, in a six-coordinate environment).^23^ Consistently, the Ln–Cp(ctr) distance is *ca.* 0.13 Å shorter in 1 in comparison to that in [Sm(Cp^ttt^)_2_] (see Table S4†).

Despite the established high reducing character of Tm(ii) complexes,^19,24^ no reaction of 1 was observed with either N_2_ or H_2_ and no traces of decomposition could be noticed upon heating a C_6_D_6_ solution of the complex at 80 °C for several days. This unusual stability contrasts with the reported reactivity of base-free samarocene [Sm(Cp*)_2_] (Cp* = η^5^-C_5_Me_5_) with N_2_.^25^ The bulky nature of the Cp^ttt^ ligand is vital to kinetically stabilize the Tm(ii) complex and avoid thermal decomposition, as observed when using the smaller Cp* or disubstituted 1,2-R_2_C_5_H_3_ (R = ^*t*^Bu, SiMe_3_) ligands.^26^ Despite the steric protection imposed by the Cp^ttt^ ligands, coordination of a Lewis base to the metal center has been achieved in related [Ln(Cp^ttt^)_2_] complexes (Ln = Yb and Sm).^22*a*,*b*^ We therefore reasoned than the high reducing character of the Tm(ii) center^19,27^ in 1 in addition to the oxophilic nature of f-elements^28^ may lead to interesting reactivity towards the gaseous oxocarbons CO and CO_2_.

### Reactivity of 1 towards CO and CO_2_

**Scheme 1:**
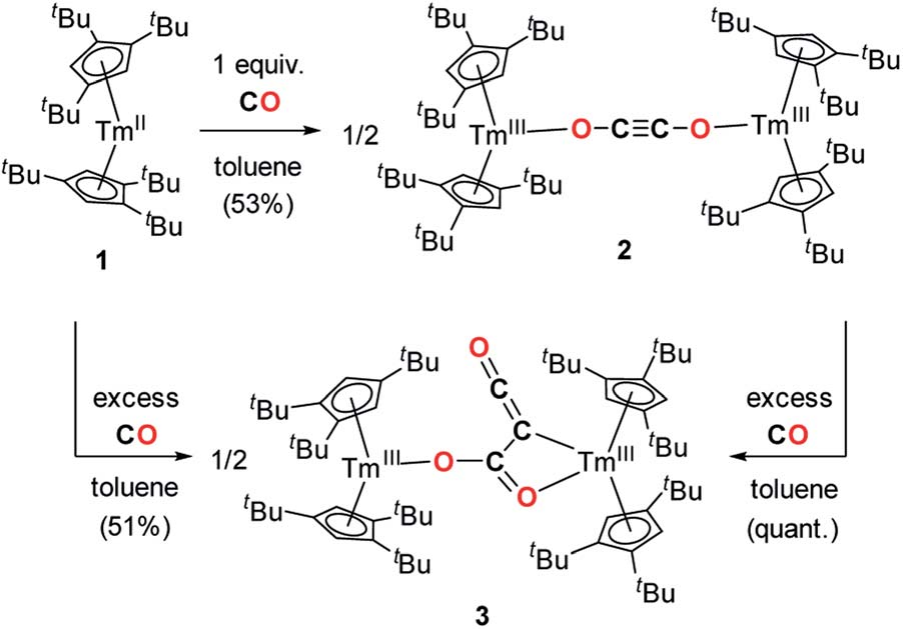
Synthesis of the CO dimerization and trimerization products 2 and 3.

Addition of CO to a degassed solution of 1 in toluene led to an immediate color change from deep purple to light brown, indicating prompt consumption of the highly colored Tm(ii) complex 1. Depending on the stoichiometry in CO, two different complexes were isolated (Scheme 1).

**Fig. 3:**
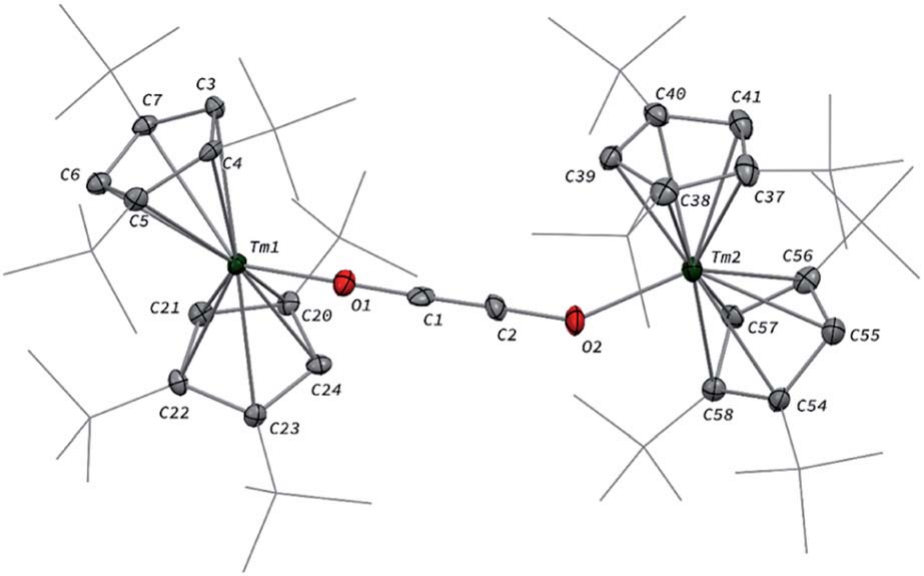
Molecular structure of 2 in the solid state with thermal ellipsoids at the 40% probability level (except for the ^*t*^Bu groups depicted in wireframe). H atoms have been omitted for clarity. Selected bond distances (Å) and angles [°]: C1–C2 1.226(10), C1–O1 1.265(9), C2–O2 1.296(9), Tm1–O1 2.066(5), Tm2–O2 2.078(5); C2–C1–O1 178.4(8), C1–C2–O2 178.1(9), C1–O1–Tm1 151.9(5), C2–O2–Tm2 146.7(5).

When the reaction was performed using one molar equivalent of CO per 1, the CO dimerization product 2 was isolated in 53% crystalline yield. The molecular structure of 2 in the solid state (Fig. 3) was unambiguously confirmed by XRD studies and revealed the formation of the dinuclear complex [Tm(Cp^ttt^)_2_]_2_(μ-κ(*O*):κ(*O*′)-C_2_O_2_) featuring a bridging ethynediolate ligand between the two oxidized Tm(iii) metal centers. The C–C bond distance in the C_2_O_2_^2−^ moiety of 1.226(10) Å is within the typical range for related ethynediolate bridged dimers of f-block elements,^16*e*,17*f*–*j*,17*l*^ and is consistent with a C≡C triple bond. The Tm–O–C angles of 146.7(5) and 151.9(5)° depart from linearity and the Tm–O bond distances of 2.066(5) and 2.078(5) Å are at the long end of the range reported for Tm(iii) complexes featuring terminal alkoxy ligands (from *ca.* 1.96 to 2.09 Å).^29^ In line with the oxidation of the thulium metal center from the +II to the +III oxidation state, the sum of the two Tm–Cp(ctr) separations for each metal center in 2, in the range 4.69–4.71 Å, is *ca.* 0.08 Å shorter than that in 1.

Analysis of the ^1^H NMR spectrum of 2 in toluene-*d*_8_ revealed only very broad resonances at room temperature, possibly resulting from a fluxional behavior of the Cp^ttt^ ligands.^20*a*,22*b*^ A better resolved spectrum featuring three broad resonances at *δ* ≈ 171, 32 and 2 ppm was obtained upon recording the spectrum at 80 °C (see Fig. S2–S3†), and is consistent with free rotation of the Cp^ttt^ ligands leading to an overall *D*_2h_ symmetric species in solution at high temperature. In the IR spectrum of 2, no significant absorption band was observed in the range 1500–2800 cm^−1^, all the more so in the expected region for C≡C triple bonds, which can be explained by the IR selection rules (lack of change in the molecular dipole). The isotopically labelled complex [Tm(Cp^ttt^)_2_]_2_(μ-κ(*O*):κ(*O*′)-^13^C_2_O_2_) (2-^13^C) was obtained by the same procedure using ^13^C-enriched CO. As expected, the IR spectra of 2 and 2-^13^C are nearly identical (see Fig. S37†), one only notable difference being the band at 1329 cm^−1^ in 2 that is red-shifted to 1304 cm^−1^ upon ^13^C labelling. The corresponding absorption band can be confidently assigned to the C–O stretching vibration, as the experimental ratio between these numbers, 1.019, compares well with the theoretical value of 1.023 obtained from reduced mass considerations. Owing to the highly paramagnetic nature of Tm(iii) complexes, no ^13^C NMR resonances could be detected, even for the isotopically enriched 2-^13^C.

The formation of ethynediolate fragments upon reductive coupling of CO has been reported for a handful of low-valent f-block complexes, mostly based on U(iii) metal centers,^17*f*–*j*^ and recently by Evans and co-workers upon treatment of *in situ* generated Ln(ii) species or [K(18-*c*-6)_2_][Tm{N(SiMe_3_)_2_}_3_] with CO.^16*e*,17*l*^ The sensitivity of the corresponding ethynediolate products to thermolysis was found to be ligand dependent.^17*g*–*i*^ In the case of 2, no traces of decomposition could be detected upon heating a toluene solution of the complex at 100 °C for several days.

**Fig. 4:**
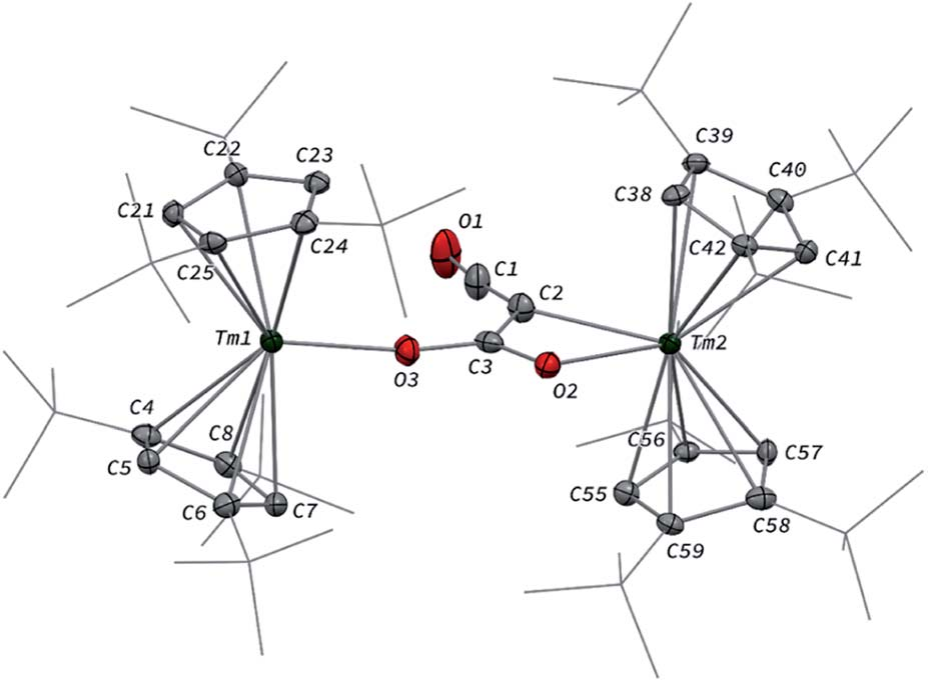
Molecular structure of 3 in the solid state with thermal ellipsoids at the 40% probability level (except for the ^*t*^Bu groups depicted in wireframe). H atoms have been omitted for clarity. Selected bond distances (Å) and angles [°]: O1–C1 1.181(9), O2–C3 1.261(8), O3–C3 1.296(8), C1–C2 1.284(10), C2–C3 1.430(9), Tm1–O3 2.098(5), Tm2–O2 2.307(5), Tm2–C2 2.473(7); C1–C2–C3 128.6(7), C3–O2–Tm2 99.2(4), C3–O3–Tm1 170.8(4), O1–C1–C2 172.2(8), O2–C3–C2 116.0(6), O3–C3–C2 123.5(6), C1–C2–Tm2 143.5(5), C3–C2–Tm2 87.5(4).

In contrast to the formation of 2, the reaction of 1 with excess CO led to the CO trimerization product [Tm(Cp^ttt^)_2_]_2_(μ-κ(*O*):κ^2^(*C*,*O*′)-C_2_O_3_) (3) isolated in 51% crystalline yield (Scheme 1). The isotopically labelled complex [Tm(Cp^ttt^)_2_]_2_(μ-κ(*O*):κ^2^(*C*,*O*′)-^13^C_2_O_3_) (3-^13^C) was obtained analogously using ^13^C-enriched carbon monoxide. The X-ray structure of 3 confirmed the formation of a ketenecarboxylate (O_2_C–C=C=O)^2−^ fragment bridging the two Tm(iii) centers (Fig. 4). The reductive trimerization of CO into such a motif has previously only been described in two reports by Evans and co-workers using reducing lanthanide complexes based on Cp* ligands.^17*a*,*b*^ The Tm–C bond distances in 3, ranging from 2.601(6) to 2.769(6) Å (in average 2.67 ± 0.06 Å) (see also Table S5†) are slightly longer than those observed in 2, which may be the result of the larger steric demand of the (μ-κ(*O*):κ^2^(*C*,*O*′)-C_2_O_3_)^2−^ bridging ligand. The latter is coordinated to the two Tm metal centers *via* a bridging carboxylate unit (Tm2–O2 2.307(5), Tm1–O3 2.098(5) Å) and further coordinated to Tm2 with a Tm2–C2 bond distance of 2.473(7) Å. The sum of the bonding angles around C2 (359.6°) is pointing to a planar arrangement for C1, C2, C3 and Tm2. In the ketenecarboxylate fragment, the bond distances within the ketene moiety (C1–C2 1.284(10) Å, C1–O1 1.181(9) Å) are indicative of double bonds while the adjacent C2–C3 bond is much longer (1.430(9) Å) and in the range of single C–C bonds. The carboxylate C3–O2 and C3–O3 bond distances of 1.261(8) and 1.296(8) Å, respectively, are very similar, supporting a delocalized negative charge over the carboxylate moiety. Overall, the metrical data within the ketenecarboxylate fragment are in good agreement with those previously reported by Evans and co-workers, the only major structural difference lying in the coordination mode of the trimerized CO unit.^17*a*,*b*^ While the (O_2_C–C=C=O)^2−^ fragment is only bridging two Tm(Cp^ttt^)_2_ subunits in 2, further dimerization and association into tetranuclear clusters were observed when using the smaller Cp* ligands.^17*a*,*b*^

The ^1^H NMR spectrum of 3 at room temperature revealed several broad resonances in the range *δ* −100 to 400 ppm, as a result of the paramagnetic nature of the complex and restricted free rotation of the Cp^ttt^ ligands.^20*a*^ Upon heating to 80 °C (see Fig. S6†), a simpler baseline was observed featuring four main and relatively broad (Δ*ν*_1/2_ ≈ 1500–3200 Hz) resonances at *δ* 185, 153, 32 and −33 ppm, assigned to the ^*t*^Bu groups. The number of signals and the corresponding integrations support free rotation of the Cp^ttt^ ligands at high temperature, resulting in an overall *C*_s_ symmetry in solution. It should be noted that complex 3 was found to be thermally stable at least up to 110 °C in toluene solution. As a result of the highly paramagnetic nature of the complex, no signals could be observed in the ^13^C{^1^H} NMR spectra of both 3 and 3-^13^C. In the IR spectrum of 3, a very strong absorption band was observed at 2066 cm^−1^ in the ketene region, shifting to 2003 cm^−1^ in 3-^13^C upon ^13^CO isotope labelling (see Fig. S38†). In order to confidently assign the corresponding absorption band, the vibrational spectra of 3 and 3-^13^C were calculated at the DFT level of theory (B3PW91, see details in the ESI†). In the computed spectra (Fig. S66–S68†), the asymmetric stretch of the O1–C1–C2 ketene fragment gives rise to an intense absorption band at 2190 cm^−1^ in 3 and 2121 cm^−1^ in 3-^13^C. Although the (uncorrected) computed values are *ca.* 120 cm^−1^ larger than the experimental ones, the ratios between the wavenumbers of two isotopologues (1.031 experimentally and 1.033 computationally) are almost identical, confirming the assignment of the band.

The overall reaction leading to the (O_2_C–C=C=O)^2−^ fragment involves a reductive trimerization of CO, more precisely the net transfer of two electrons from two Tm(ii) centers to three molecules of CO. Since the central carbon atom (C2, Fig. 4) in 3 presents no C–O connectivity, complete cleavage of one CO triple bond has occurred. Although CO cleavage and further homologation is thought to occur in heterogeneous Fischer–Tropsch systems, such reactivity is not common in homogeneous systems.^4*c*,7*b*,14*a*,17*a*,17*b*^

The observed CO reductive homologation induced by the Tm(ii) complex 1, leading to the selective formation of either the ethynediolate complex 2 or the ketenecarboxylate complex 3, is the result of a fine balance between steric protection and coordinative unsaturation. A similar observation was already reported in the case of U(III) mixed-sandwich complexes reported by Cloke and co-workers, which allowed possible reductive CO di-, tri- or tetramerization depending on the steric demand of the supporting ligands and amount of CO.^17*j*^ Since selective formation of the CO dimerized product 2 is observed in the presence of one molar equivalent of CO per Tm(ii) center, we further examined whether 2 may be an intermediate in the formation of 3. In a control experiment, the reaction of isolated 2 in toluene solution with excess CO was monitored by paramagnetic ^1^H NMR spectroscopy (see Fig. S7†), revealing clean and quantitative transformation into 3. This reaction is relatively slow at room temperature, requiring *ca.* 1–2 days to reach completion, which suggests a relatively high activation barrier. To gain further insights in the elementary steps leading to the formation of 2 and 3, *i.e.* the overall mechanism of this unusual reaction, DFT calculations were performed and confirmed that 2 appears indeed as an intermediate in the formation of 3 (see below). To the best of our knowledge, this result is unprecedented since all reported ethynediolate complexes have been either synthesized under excess of CO,^15*j*,17*g*–*i*,17*l*^ or the isolated products were found inert towards external CO.^17*f*^ To date, systems allowing controlled carbon chain growth by sequential insertions of CO on isolated intermediates are exceedingly rare. Such examples include CO addition on the boron–boron triple bond of a diboryne compound described by the group of Braunschweig,^15*a*^ and recent work by Crimmin and co-workers on systems based on transition metal carbonyls together with aluminum(i) reductants.^15*c*,*k*^

Finally, to investigate whether other reaction conditions have an influence on the selectivity, temperature and solvent effects were examined. Performing the addition of CO at low temperature (−78 °C) or in different solvents (pentane, benzene, Et_2_O and THF) led to similar results signifying that the stoichiometry in CO is the only parameter controlling the selectivity in this reaction. It should be however noted that the reaction proceeds much more slowly in coordinating solvents such as THF, requiring several days until complete consumption of 1 (see Fig. S4†), which can be traced back to competitive coordination of THF and CO to the metal center and a relatively high activation barrier (see below). An inhibitory role of THF in the reduction of organic substrates by Sm(ii) complexes has previously been observed in the literature.^30^ Interestingly, in the U(iii) systems reported by Arnold and co-workers, THF coordination was found to completely stop the reactivity towards CO.^17*g*,*h*^ Such a different behavior may originate in the steric demand of the Cp^ttt^ ligands that induces weak and reversible coordination of the usually strongly bound THF donor.^21*a*^ A greater degree of covalency of U–L *vs.* Ln–L bonds (L = THF or other donor ligands) may also contribute to decrease the L lability and suppress reactivity for U(iii) systems in donor solvents.^31^ The unique steric properties of the Cp^ttt^ ligand may also explain the different reactivity of [Sm(Cp^ttt^)_2_] and [Sm(Cp*)_2_] towards CO. Although both Sm^II^ complexes have very similar redox potentials associated with the Sm^III^/Sm^II^ couple (*ca.* −2.12 and −2.10 V *vs.* ferrocenium/ferrocene in THF, respectively),^22*b*,32^ [Sm(Cp^ttt^)_2_] is inert towards CO,^20*a*^ while the Cp* analog leads to CO reductive trimerization.^17*a*^

The reactivity of the Tm(ii) complex 1 was also investigated towards CO_2_ as divalent lanthanide complexes have been reported to yield oxalate and/or carbonate complexes upon reduction of CO_2_.^33^ The reaction of 1 with excess CO_2_ led to the formation of the carbonate complex 4 isolated in 72% yield. Its IR spectrum features a strong absorption band at 1430 cm^−1^, assigned to C–O stretching in the coordinated carbonate ligand.^34^ The X-ray structure of the complex (see Fig. S44†) confirmed the presence of a bridging carbonate ligand with a μ-κ(*O*):κ^2^(*O*′,*O*′′) coordination mode between the two Tm^III^(Cp^ttt^)_2_ fragments, as previously observed for the Sm(iii) analogue [Sm(Cp^ttt^)_2_(CO_3_)].^20*a*^ A more detailed description of the structure can be found in the ESI.†

### Mechanistic insights by DFT calculations

**Fig. 5:**
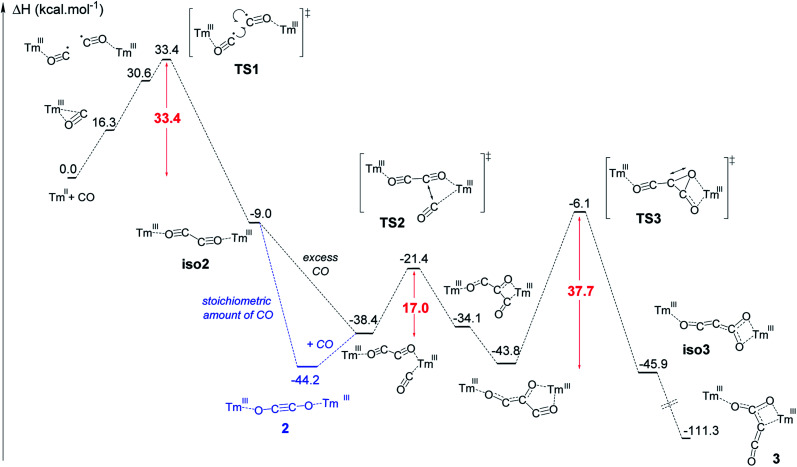
Computed enthalpy profile at room temperature for the formation of 2 and 3.

The formation of complex 3 has been investigated at the DFT level (B3PW91) and the computed enthalpy profile at room temperature is depicted in Fig. 5. The reaction begins with the side-on coordination of CO to 1. This coordination induces the reduction of CO and the oxidation of Tm(ii) to Tm(iii), the radical on CO being stabilized by η^2^ coordination to the metal center. This step is computed to be endothermic by 16.3 kcal mol^−1^, which fits well with immediate changes in the color and ^1^H NMR spectrum of the solution in toluene-*d*_8_. Unlike the calculated pathway for CO reductive coupling at U(iii) mixed-sandwich complexes,^18*b*^ the CO moiety is not further reduced by the coordination of a second molecule of 1 but rather a radical coupling between two Tm(iii)(CO^˙−^) fragments is observed. This step also contrasts with that calculated for CO reductive oligomerization on Mg(i) dimers where the addition of CO occurs sequentially (see additional details in the ESI†).^15*e*,*g*,15*j*^ It can be noted that a radical pathway for CO oligomerizations has been recently suggested on the basis of fragmentation reactions by mass spectrometry and computational quantum calculations on squarate species, *i.e.* CO tetramerization products.^35^

In addition, the possible formation of a lanthanide complex featuring a reduced (CO)^˙−^ ligand has already been reported in the case of highly reducing and transient Y(ii) and Lu(ii) complexes,^16*e*,17*l*^ which supports the generation of a related (CO)^˙−^ intermediate upon reaction of 1 with CO. The associated transition state (TS1) has been located on the Potential Energy Surface (PES). The radical coupling barrier is 33.4 kcal mol^−1^ from 1 (but only 17.1 kcal mol^−1^ from the Tm(iii) intermediate complex), in line with a kinetically accessible but relatively slow reaction. Accordingly, slow evolution in the ^1^H NMR spectrum of the reaction between 1 and CO (1 equiv.) in toluene-*d*_8_ was observed over several hours at room temperature. The radical nature of the TS is highlighted by the computed unpaired spin densities (see Fig. S56†) where 0.6 unpaired electron is located on the two carbons that couple. Following the intrinsic reaction coordinate, the formation of the ethynediolate complex iso2 is favorable (−9.0 kcal mol^−1^ with respect to 1). This complex displays a “zig-zag” geometry which is the key intermediate in this reactivity (see Fig. 1) and is similar to the “zig-zag” intermediate suggested in previous studies.^15*e*,*g*,17*f*,*g*,18*b*^ This intermediate can either yield the more stable linear structure, 2, which is what happens with a stoichiometric amount of CO, or bind another CO molecule, as obtained for a CO excess. In our case, these two possibilities are thermodynamically competitive (the CO coordination is exothermic by 29.4 kcal mol^−1^ from iso2 while the linear ethynediolate complex 2 is 35.2 kcal mol^−1^ more stable than the “zig-zag” one). The formation of the stable CO adduct is easily explained by the nature of the HOMO of iso2, that can easily overlap with the π* orbital of CO (see Fig. S57†). It is interesting to note that the optimized geometry of a CO adduct on 2 yields the same structure, so that complex 2 is also linked to the computed reaction profile with an excess of CO. From the CO adduct, a C–C coupling TS has been located and corresponds to a nucleophilic attack of the ethynediolate to the π system of CO (see the HOMO at the TS in Fig. S58†). The associated barrier is low (17.0 kcal mol^−1^), yielding a stable ketene-type intermediate (−43.8 kcal mol^−1^). This ketene intermediate can isomerize with a barrier of 37.7 kcal mol^−1^ (TS3) to form complex 3 (see details in the ESI†), which is the most stable complex of the entire profile (−111.3 kcal mol^−1^). The relatively high activation barrier calculated for TS3 (*ca.* 38 kcal mol^−1^) is consistent with a relatively slow (1–2 days) transformation of 2 to 3 in the presence of excess CO at room temperature. It should be noted that an activation barrier of up to *ca.* 40 kcal mol^−1^ is not unprecedented for a reaction involving a lanthanide metallocene which is occurring slowly at room temperature.^36^

Additional single-point calculations using a larger basis set or other functionals (wB97xd and M062X) (see ESI and Fig. S63–S65†) showed slightly lower but still relatively high activation barrier energies. Despite this barrier being at the upper limit of that typically accepted for a reaction occurring at room temperature, it should be noted that, from a strictly thermodynamic point of view, 3 is much more stable than 2 (67.1 kcal mol^−1^ lower in energy), which drives the reaction towards formation of the CO trimerization product 3.

To further investigate the validity of the DFT calculation results, the reaction between 2 and excess ^13^CO was performed. The IR spectrum of the corresponding product (Fig. S40†) shows an intense absorption band at 2004 cm^−1^, very similar to that of 3-^13^C (2003 cm^−1^), indicating that scrambling of ^13^C labels occurred in the presence of excess ^13^CO. A possible mechanism to explain the ^13^C scrambling step, consistent with the computed energetic profile, can be found in the ESI (Scheme S1†).

### Reactivity of 3 towards electrophiles

Having complex 3 at hand with a reproducible procedure and in decent isolated yield, we investigated its reactivity towards electrophiles. Indeed, the ketenecarboxylate complex 3 presents a polarized lanthanide–carbon bond, which is prone to further functionalization or insertion reactions.^33,37^ It should be noted that, for the two previously reported ketenecarboxylate complexes based on Ln(Cp*)_2_ (Ln = La, Sm) fragments,^17*a*,*b*^ no study of their reactivity was described.

**Scheme 2:**
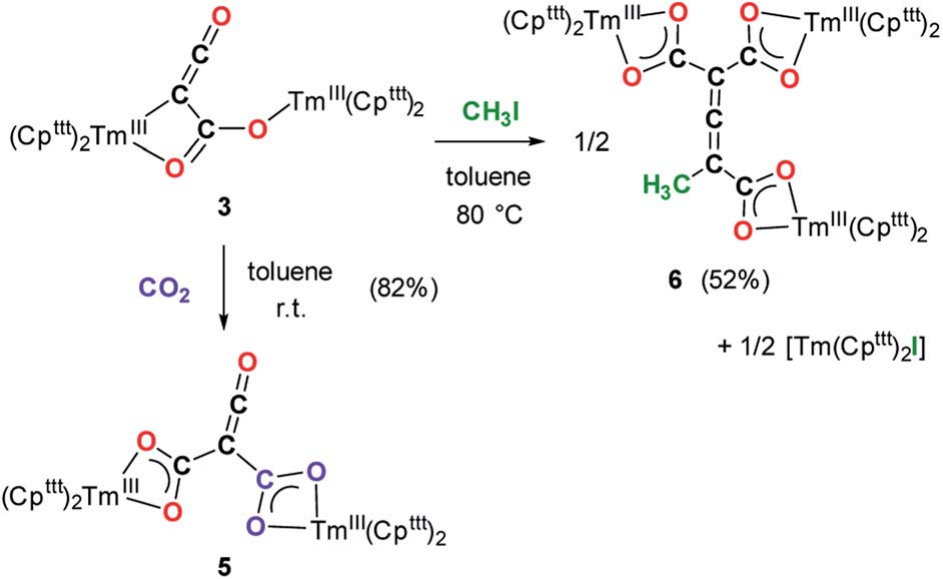
Reactivity of 3 towards CO_2_ and MeI.

**Fig. 6:**
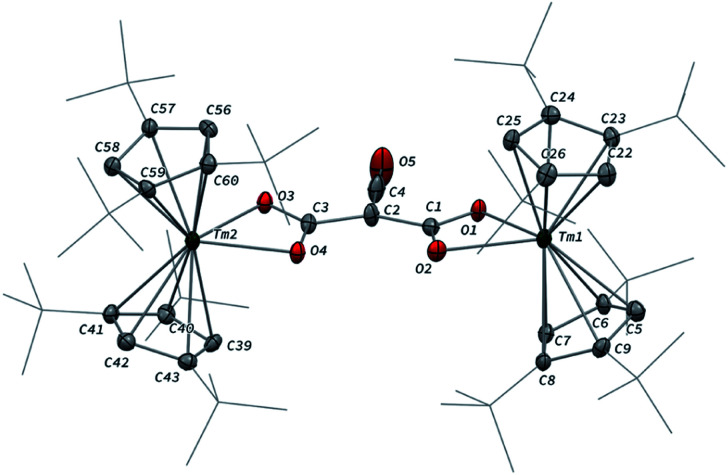
Molecular structure of 5 in the solid state with thermal ellipsoids at the 40% probability level (except for the ^*t*^Bu groups depicted in wireframe). H atoms have been omitted for clarity. Selected bond distances (Å) and angles [°]: Tm1–O1 2.282(5), Tm1–O2 2.354(5), Tm2–O3 2.305(5), Tm2–O4 2.318(5), O1–C1 1.283(8), O2–C1 1.263(8), O3–C3 1.273(8), O4–C3 1.267(8), O5–C4 1.142(10), C1–C2 1.460(9), C2–C3 1.461(10), C2–C4 1.343(10); O1–Tm1–O2 56.9(2), O3–Tm2–O4 57.0(2), O1–C1–C2 116.0(6), O2–C1–O1 120.3(6), O2–C1–C2 123.6(6), C1–C2–C3 131.7(6), C1–C2–C4 113.0(7), C3–C2–C4 115.3(6), O3–C3–C2 118.1(6), O3–C3–O4 120.5(6), O4–C3–C2 121.4(6), O5–C4–C2 178.5(10).

The reaction of 3 with CO_2_ in toluene-*d*_8_ proceeded instantaneously and cleanly at room temperature, as evidenced by paramagnetic ^1^H NMR monitoring of the reaction. The corresponding product 5 was isolated in 82% yield (Scheme 2) and crystallographically characterized. Analysis of the molecular structure of 5 in the solid state (Fig. 6) revealed the insertion of CO_2_ in the Tm–C bond of 3, yielding a nearly planar bridging μ-κ^2^(*O*):κ^2^(*O*)-ketenedicarboxylate ligand. The two Tm(iii) centers are each surrounded by a chelating carboxylate group and two η^5^-coordinated Cp^ttt^ ligands. The C–C bond distances between the ketene and carboxylate groups are identical within experimental error (C1–C2 1.460(9) Å and C2–C3 1.461(10) Å) resulting in an overall and non-crystallographically imposed *C*_2v_ symmetry (not taking into account the substitution patterns on the Cp^ttt^ ligands). The C2–C4 and O5–C4 bond distances, 1.343(10) and 1.142(10) Å, respectively, are consistent with double bonds and a ketene fragment, and are similar to the corresponding distances in 3 (C1–C2 and O1–C1 bond distances of 1.284(10) and 1.181(9) Å, respectively). The sums of the two Tm–Cp(ctr) separations for both metal center in 5 are 4.735 and 4.746 Å, with Tm–C bond distances ranging from 2.618(7) to 2.733(7) Å (in average 2.66 ± 0.04 Å) (See also Table S5†).

The ^1^H NMR spectrum of 5 in toluene-*d*_8_ revealed six main broad and paramagnetically shifted resonances at room temperature (see Fig. S11 and S12†), resulting from restricted rotation of the Cp^ttt^ ligands around the metal center. Upon heating to 60 °C, the spectrum evolved into three very broad (Δ*ν*_1/2_ ≈ 10 000–17 000 Hz) resonances at *δ* −16, 51 and 197 ppm, which is in agreement with freely rotating Cp^ttt^ ligands and an overall *C*_2v_ symmetry in solution. In the IR spectrum of 5, the two bands were detected at 2157 and 2146 cm^−1^, assigned to the asymmetric ketene stretching vibration,^38^ along with an intense absorption band at 1572 cm^−1^ corresponding to the asymmetric stretching of the coordinated carboxylate groups.^39^

**Fig. 7:**
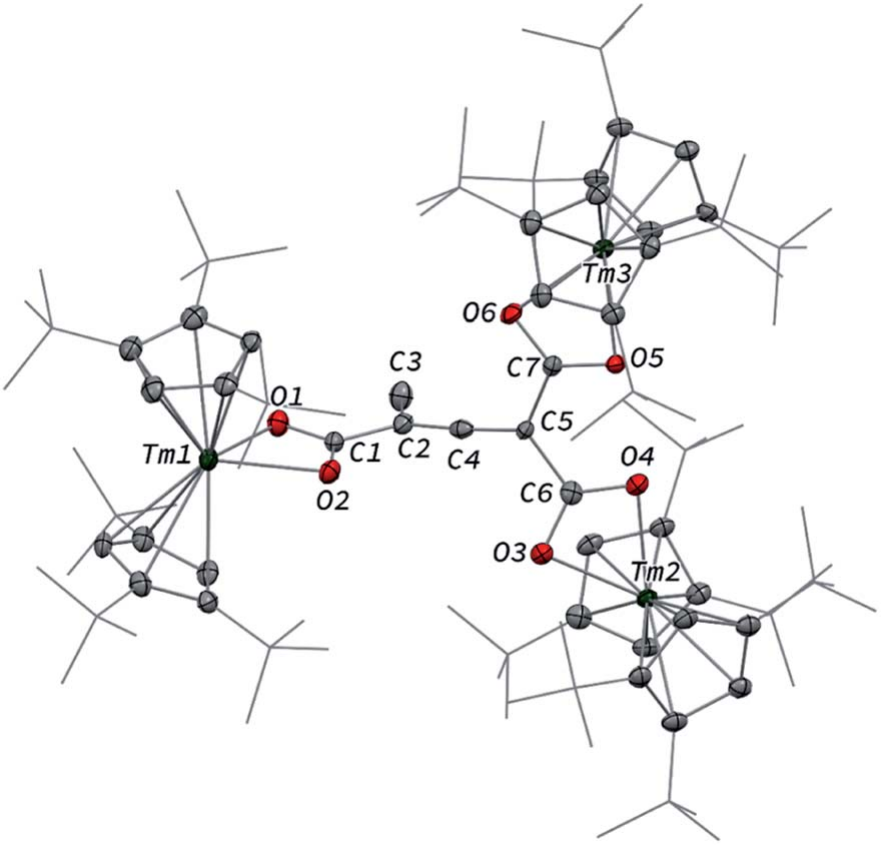
Molecular structure of 6 in the solid state with thermal ellipsoids at the 40% probability level (except for the ^*t*^Bu groups depicted in wireframe). H atoms have been omitted for clarity. Selected bond distances (Å) and angles [°]: Tm1–O1 2.299(3), Tm1–O2 2.315(3), Tm2–O3 2.319(3), Tm2–O4 2.307(3), Tm3–O5 2.304(3), Tm3–O6 2.333(3), O1–C1 1.274(5), O2–C1 1.268(5), O3–C6 1.284(5), O4–C6 1.269(5), O5–C7 1.271(4), O6–C7 1.268(4), C1–C2 1.486(6), C2–C3 1.501(6), C2–C4 1.300(6), C4–C5 1.325(5), C5–C6 1.512(5), C5–C7 1.492(5); O1–C1–C2 118.1(4), O1–C1–O2 119.6(4), O2–C1–C2 122.2(4), C1–C2–C3 116.6(4), C1–C2–C4 121.1(4), C3–C2–C4 122.3(4), C2–C4–C5 172.2(4), C4–C5–C6 117.6(3), C4–C5–C7 119.4(3), C6–C5–C7 122.3(3), O3–C6–C5 118.2(4), O3–C6–O4 121.8(4), O4–C6–C5 119.9(3), O5–C7–C5 119.7(3), O5–C7–O6 121.4(4), O6–C7–C5 119.0(3).

Although 3 was found to react with Me_3_SiCl upon heating to 60 °C, no crystals suitable for XRD studies could be obtained to unambiguously assign the nature of the formed products. When the reaction was performed using MeI as electrophile (Scheme 2), no reaction occurred at room temperature but slow conversion of 3 was observed in toluene-*d*_8_ upon heating the reaction mixture at 80 °C for 3 days. Paramagnetic ^1^H NMR monitoring of the reaction revealed the formation [Tm(Cp^ttt^)_2_I], identified by comparison with the ^1^H NMR spectrum of an authentic sample, along with a new set of signals (see Fig. S15†). After evaporation of the solvent, the crude product was washed several times with pentane to remove [Tm(Cp^ttt^)_2_I]. Recrystallization of the remaining solid from hot toluene afforded crystals of 6 suitable for XRD studies. Its molecular structure (Fig. 7) features a trinuclear Tm(iii) complex in which each thulium center is coordinated by a chelating carboxylate group and two η^5^-coordinated Cp^ttt^ ligands. The C2–C4 and C4–C5 bond distances in 6 of 1.300(6) and 1.325(5) Å, respectively, together with the almost linear C2–C4–C5 arrangement (bonding angle of 172.2(4)°), are consistent with C=C double bonds in an allene-type structure. In contrast, the C1–C2, C2–C3, C5–C6 and C5–C7 bonds, with distances from 1.486(6) to 1.512(5) Å, are in the typical range for C–C single bonds. The carbon atoms C2 and C5 present a trigonal planar geometry (sum of the bonding angles around C2 and C5 of 360.0 and 359.3°), consistent with sp^2^ hybridization. The Tm–O bond distances in 6, in the range 2.299(3)–2.333(3) Å, are similar to those observed in the dicarboxylate complex 5 (from 2.282(5) to 2.354(5) Å).

The ^1^H NMR spectra of 6 in the 20–80 °C temperature range display a complex pattern of paramagnetically shifted signals (Fig. S14†), as a result of the non-symmetric nature of the complex. In the IR spectrum of 6, the intense absorption band at 1583 cm^−1^ corresponds to the asymmetric stretching band of the coordinated carboxylate groups, similarly to the absorption band observed in 5. In addition, a weak band is observed at 1938 cm^−1^ and assigned to the asymmetric C=C=C stretching vibration.^39^

Although the mechanism for the formation of 6 is elusive to date and involves several rearrangement steps, the observed reactivity finds its origin in the reactive Tm–C bond in 3. It should be stressed that the stoichiometry of the reaction is strictly respected as one equivalent of both 6 and [Tm(Cp^ttt^)_2_I] are formed from two equivalents of 3, in other words, one tris-anionic {MeC_3_(CO_2_)_3_}^3−^ framework and one iodide ligand are the result of the reaction of two di-anionic {C_3_O_3_}^2−^ fragments with one molecule of MeI. Overall, this reaction corresponds to an unprecedented oligomerization and functionalization of CO through formation of an allene–tricarboxylate complex in which the {C_6_O_6_} core exclusively arises from six CO molecules.

### Reactivity of 2 towards electrophiles

**Scheme 3:**
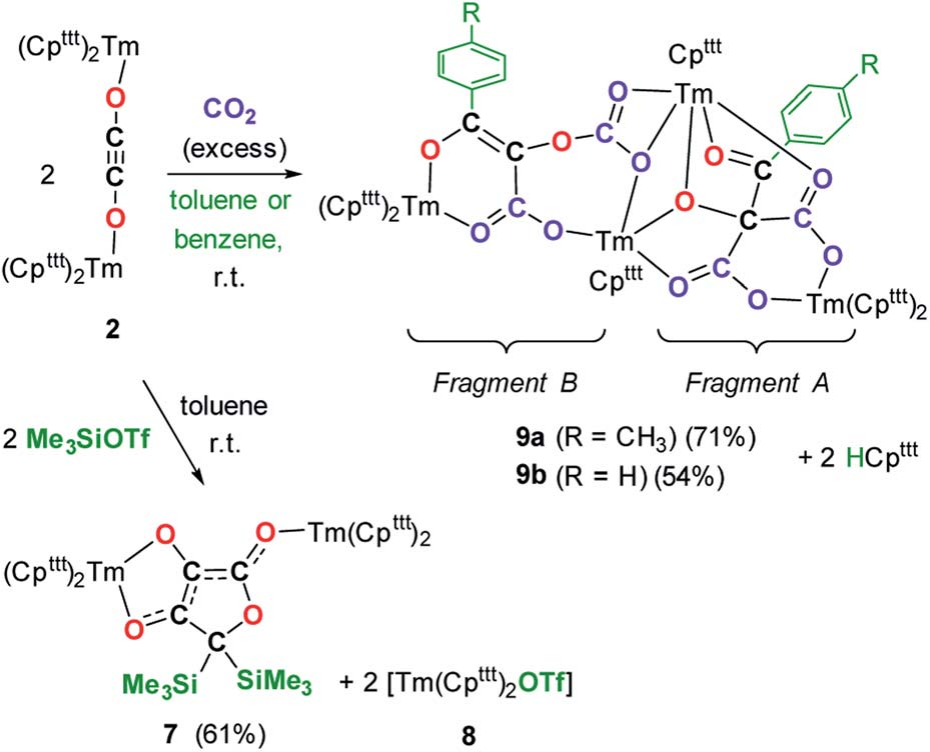
Reaction of 2 with CO_2_ and Me_3_SiOTf.

**Fig. 8:**
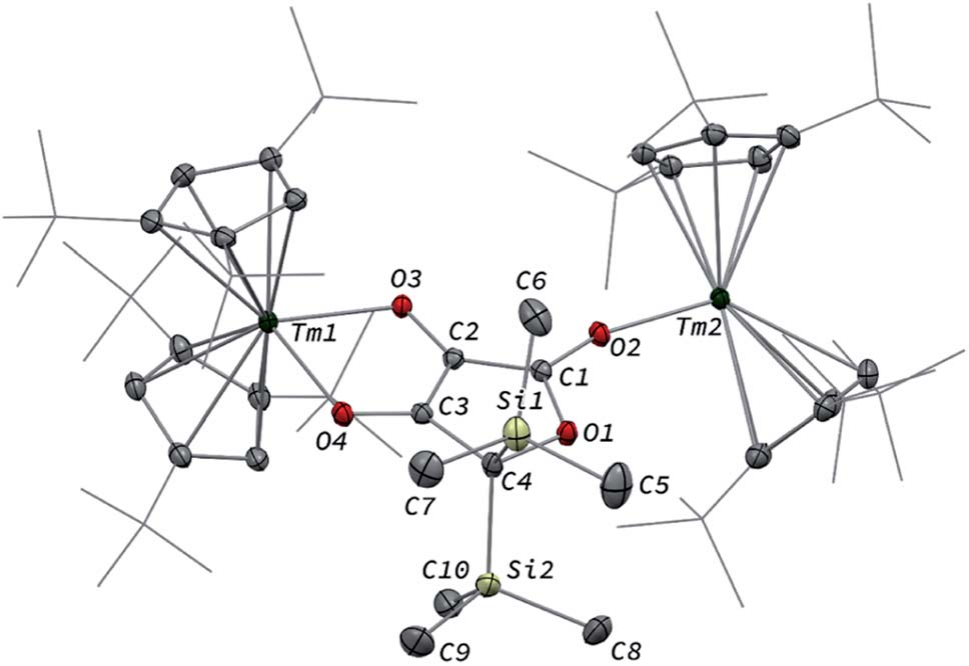
Molecular structure of 7 in the solid state with thermal ellipsoids at the 40% probability level (except for the ^*t*^Bu groups depicted in wireframe). H atoms have been omitted for clarity. Selected bond distances (Å) and angles [°]: Tm1–O3 2.200(2), Tm1–O4 2.333(2), Tm2–O2 2.146(2), O1–C1 1.368(3), O1–C4 1.486(3), O2–C1 1.280(3), O3–C2 1.339(3), O4–C3 1.290(3), C1–C2 1.397(3), C2–C3 1.396(3), C3–C4 1.494(3); O3–Tm1–O4 74.78(6), C1–O2–Tm2 163.1(2), C2–O3–Tm1 112.88(13), C3–O4–Tm1 109.86(14).

Intrigued by the reactivity of 3 towards electrophiles, we examined the reactivity of 2 towards CO_2_ and silyl electrophiles. The reactivity of 2 was investigated towards silylating agents to evaluate the possibility of decoordination and liberation of the anionic oxocarbon ligand. It should be noted that decoordination of CO reduction products from oxophilic U(iv) centers has previously been achieved upon treatment with silyl electrophiles.^17*i*,*j*,40^ No reaction occurred upon addition of excess Me_3_SiCl to 2, even upon heating to 80 °C. In contrast, the addition of Me_3_SiOTf to a solution of 2 in toluene-*d*_8_ led to a complete conversion after 15 h at room temperature (Scheme 3). Analysis of the reaction mixture by paramagnetic ^1^H NMR spectroscopy revealed resonances corresponding to [Tm(Cp^ttt^)_2_OTf] (8), identified by comparison with the ^1^H NMR spectrum of an authentic sample of 8 (see ESI and Fig. S18†), along with a new set of paramagnetically shifted signals. Crystallization from a concentrated pentane solution afforded yellow crystals of 7 suitable for XRD studies. Its molecular structure (Fig. 8) showed an unusual CO tetramerization product featuring a deprotonated 3,4-dihydroxyfuran-2-one moiety bridging two Tm(iii) metal centers.

The bond distances within the oxygen heterocycle are consistent with a conjugated system and delocalization of one negative charge over O2 and O4, similar to the case in β-diketonate ligands. As a result, the C1–C2 and C2–C3 bond distances are almost identical (1.397(3) and 1.396(3) Å, respectively), as are the O2–C1 and O4–C3 bond distances (1.280(3) and 1.290(3) Å, respectively). In contrast, the unconjugated O3–C2 bond is longer with a distance of 1.339(3) Å. The Tm–O bond distances are lying in the range 2.200(2)–2.333(2) Å. The paramagnetic ^1^H NMR spectrum of 7 features a complex baseline over the temperature range 20–80 °C (see Fig. S17†), which results from the low symmetry of the complex in solution. In the IR spectrum, the coordinated β-diketonate group gives rise to an intense absorption band at 1639 cm^−1^.

Interestingly, reaction with Me_3_SiOTf did not lead to decoordination of the ethynediolate ligand, contrary to the reactivity observed by Liddle and co-workers upon treatment of a related *tris*-amido U(iv) ethynediolate complex with a similar strong silyl electrophile.^17*i*^ In this previous study, the reaction with Me_3_SiI led to the silylation of the hydroxy groups and liberation of the ethynediolate fragment, which subsequently dimerized and rearranged into a spectroscopically identified furanone product. Indeed, acetylene diether compounds have been found to be stable at room temperature only in the presence of bulky substituents,^41^ or η^2^-coordinated to transition metals.^16*b*^ The X-ray authenticated complex 7 unambiguously shows the formation of a similar furanone moiety in which the two trimethylsilyl groups are attached at the C4 position of the heterocycle rather than the O3 and O4 oxygen atoms, the latter remaining coordinated to the metal center. The mechanism leading to the formation of 7 is thought to involve electrophilic activation of the ethynediolate by the silyl electrophile, which contrasts with the proton-induced transformation reported earlier.^17*i*^ The exploration of other functionalization agents to allow the liberation of the ethynediolate moiety is currently in progress.

**Fig. 9:**
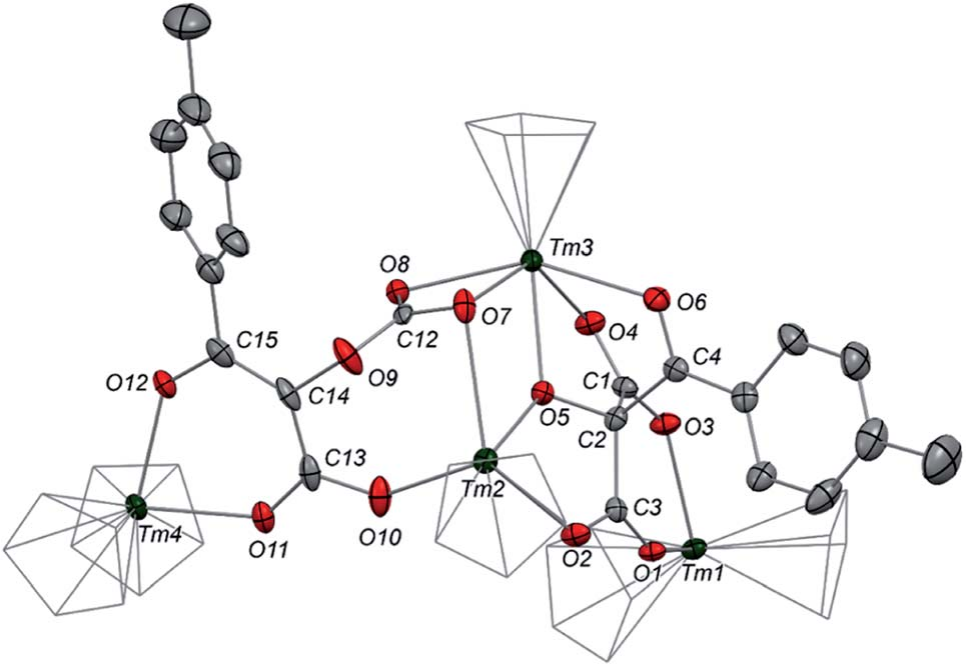
Molecular structure of 9a in the solid state with thermal ellipsoids at the 40% probability level (except for the Cp groups depicted in wireframe). H atoms, ^*t*^Bu groups and non-coordinating solvent molecules have been omitted for clarity. Selected bond distances (Å): Tm1–O1 2.273(3), Tm1–O3 2.242(3), Tm2–O2 2.266(3), Tm2–O5 2.263(3), Tm2–O7 2.643(3), Tm2–O10 2.156(3), Tm3–O4 2.261(3), Tm3–O5 2.294(3), Tm3–O6 2.386(3), Tm3–O7 2.428(3), Tm3–O8 2.333(3), Tm4–O11 2.234(3), Tm4–O12 2.195(3), O1–C3 1.244(4), O2–C3 1.252(5), O3–C1 1.248(4), O4–C1 1.259(4), O5–C2 1.402(4), O6–C4 1.235(5), O7–C12 1.271(4), O8–C12 1.231(5), O9–C12 1.338(5), O9–C14 1.428(5), O10–C13 1.291(5), O11–C13 1.260(5), O12–C15 1.300(5), C1–C2 1.565(5), C2–C3 1.550(5), C2–C4 1.567(5), C13–C14 1.426(6), C14–C15 1.384(6).

We next evaluated the reactivity of 2 towards CO_2_ (Scheme 3). The addition of CO_2_ (1.3 bar, excess) to a solution of 2 in toluene led to an immediate reaction at room temperature. After work-up, red crystals of 9a suitable for XRD studies were obtained in good yield (71%) from a concentrated pentane solution. The molecular structure of the complex is depicted in Fig. 9 and reveals an unexpected product in which C–H activation at the *para* position of the toluene solvent occurred, yielding an unprecedented polyoxygenated ligand framework. In the resulting tetranuclear Tm(iii) complex, only six Cp^ttt^ ligands are present, suggesting that two Cp^ttt^ ligands have been released in their protonated form. The formation of HCp^ttt^ was confirmed by ^1^H NMR analysis of the volatiles of the reaction (see details in the ESI and Fig. S23†). The thulium centers are hold together *via* two isomeric bridging {μ_3_-C_7_H_7_(C_4_O_6_)}^2−^ fragments. Fragment A (see Scheme 3) is best described by a μ_3_-κ^2^(*O*^1^,*O*^3^):κ^2^(*O*^2^,*O*^5^):κ^3^(*O*^4^,*O*^5^,*O*^6^)–C_7_H_7_(C_4_O_6_) ligand based on a 2-hydroxy-2-(*p*-toluoyl)malonate framework while fragment B corresponds to a μ_3_-κ^2^(*O*^7^,*O*^8^):κ^2^(*O*^7^,*O*^10^):κ^2^(*O*^11^,*O*^12^)–C_7_H_7_(C_4_O_6_) ligand derived from 2-(carboxyoxy)-3-hydroxy-3-(*p*-tolyl)acrylic acid (a simplified view in which the Cp^ttt^ ligands have been omitted is depicted in Fig. S52†). Both Tm1 and Tm4 are coordinated by two η^5^–Cp^ttt^ ligands and two oxygen atoms with Tm–O bond distances in the range 2.195(3)–2.273(3) Å. In contrast, Tm2 and Tm3 feature half-sandwich arrangements, both surrounded by one η^5^–Cp^ttt^ ligand along with four and five oxygen donors, respectively. The corresponding Tm–O bond distances are spanning a larger range (2.156(3)–2.643(3) Å), as a result of the different types of oxygen donors, namely alkoxide, ketone, carboxylate and carbonate groups. The tetrasubstituted carbon C2 in fragment A features C–C bond distances of 1.550(5)–1.567(5) Å corresponding to single bonds, whereas the C13–C14 and C14–C15 bond distances in fragment B of 1.426(6) and 1.384(6) Å, respectively, are more consistent with a delocalized double bond within the 3-hydroxyacrylate moiety. Accordingly, the sum of the bonding angles around C14 and C15 of 360.0° is indicative of sp^2^ hybridization.

The ^1^H NMR spectrum of 9a in toluene-*d*_8_ (Fig. S20†) shows a very complex pattern with several paramagnetically shifted resonances in the range *δ* −360 to +300 ppm, as a result of the *C*_1_ symmetric nature of 9a. The signals corresponding to the *p*-tolyl fragments could be successfully assigned by comparison with the spectrum of the partly deuterated 9a-^2^H (see Fig. S21–S22†), the latter prepared using the same procedure but in toluene-*d*_8_ instead of protio-toluene.

The IR spectrum of 9a features a very intense absorption band at 1638 cm^−1^, consistent with a coordinated ketone group, along with strong absorption bands at 1585 and 1552 cm^−1^, which can be assigned to the carboxylate groups or delocalized C=C bond in the newly formed ligand. Performing the same reaction in benzene instead of toluene as solvent led to the isolation of complex 9b which X-ray structure revealed identical bonding and substitution patterns as observed in 9a (see Fig. S53 and S54†).

**Scheme 4:**
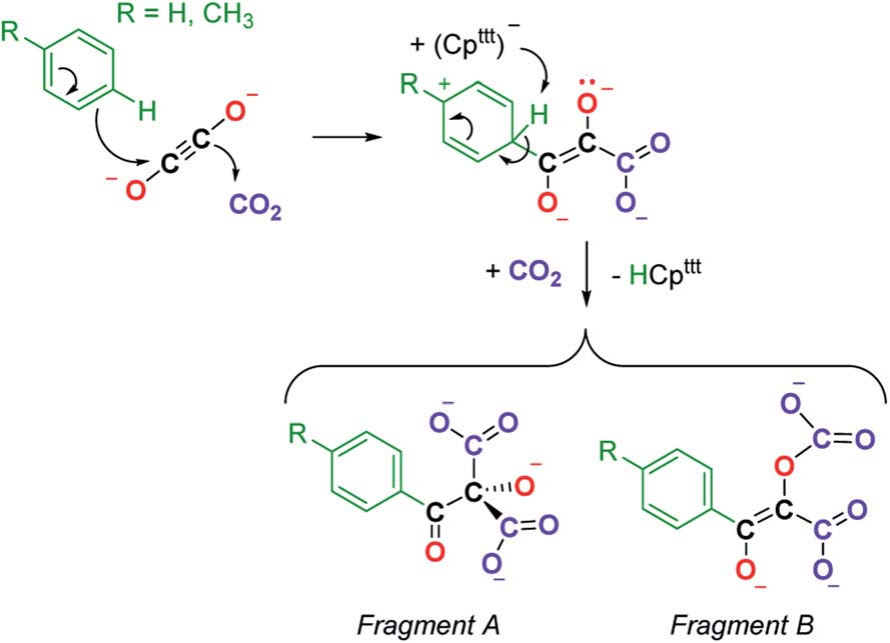
Possible simplified mechanism in the formation of 9a–9b leading to the formation of fragments A and B.

A detailed study of the elementary steps leading to the formation of 9a–9b is outside the scope of this publication and will be disclosed in due course. A possible simplified mechanism to account for the formation of the two polyoxygenated fragments is depicted in Scheme 4. In both cases, it would begin with an electrophilic aromatic substitution on the toluene or benzene solvent induced by the interaction of the ethynediolate complex 2 with CO_2_. The acidic proton in the Wheland intermediate is trapped by one Cp^ttt^ ligand, resulting in the release of HCp^ttt^, which was observed spectroscopically. This first step could therefore be seen as a Friedel–Crafts-type reaction leading to the functionalization of toluene or benzene and formation of a deprotonated form of 2,3-dihydroxy-3-(*p*-tolyl)acrylate. This polyoxygenated intermediate further reacts with one more equivalent of CO_2_ either at the nucleophilic carbon atom or at the non-conjugated anionic oxygen atom, yielding fragments A or B, respectively. It should be noted that this reaction proceeds smoothly at room temperature and contrasts with the thermolysis reactivity of some uranium(iv) ethynediolate complexes that were found to undergo ligand degradation or intramolecular C–H activation at elevated temperature, leading to ethenediolate-type complexes.^17*h*,*i*^

The observed C–H activation of toluene and benzene in 9a and 9b, respectively, is reminiscent of the C–H activation at the coordinated 4-dimethylaminopyridine (DMAP) ligand that has been described upon addition of CO to an activated Mg(i) dimer complex (see Fig. 1).^15*g*^ In this case, DFT calculations suggested that the C–H activation is induced by a “zig-zag” {C_2_O_2_}^2−^ intermediate similar to iso2 (Fig. 5).

The major difference in the case of 2 in comparison to other systems lies in its lack of reactivity in toluene in the absence of CO_2_, even at elevated temperatures (up to 100 °C). Only upon addition of CO_2_ is a highly reactive system formed that allows intermolecular C–H activation on toluene or benzene.

## Conclusions

**Scheme 5:**
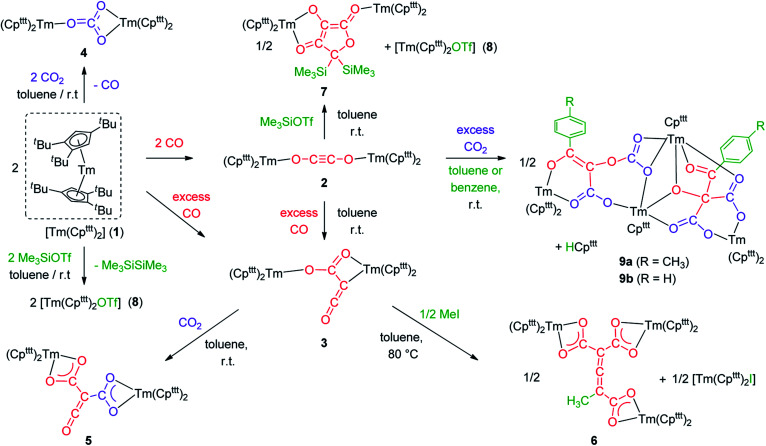
Summary scheme of all the reactivity described in this study starting from [Tm(Cp^ttt^)_2_] (1).

In conclusion, the divalent thulium complex [Tm(Cp^ttt^)_2_] exhibits a rich reactivity towards CO and CO_2_ allowing the selective formation of carbonate (C_1_), ethynediolate (C_2_) and ketenecarboxylate (C_3_) complexes. The reactivity of the CO dimerization product 2 with external CO at room temperature is remarkable and proves the intermediacy of 2 in the formation of the ketenecarboxylate complex 3, which was also supported by DFT calculations. The reactivity of 2 and 3 was systematically investigated towards electrophiles (see Scheme 5 for a summary) leading to the following observations: (a) while 3 features an expected nucleophilic reactivity towards CO_2_, the addition of methyl iodide yields a C_7_ allene–tricarboxylate complex in which the C_6_O_6_ core is exclusively built from CO molecules; (b) the ethynediolate complex 2 shows unexpected reactivity towards CO_2_ and silylating agents. In the case of CO_2_, a highly reactive species is formed, which is able to induce an intermolecular C–H activation on the solvent. This novel reactivity opens new avenues for the formation of multicarbon oxygenated compounds by small molecule activation and the functionalization of CO and CO_2_ to value-added chemicals. The exploration of new procedures to enable decoordination of the oxygenated products and further catalytic applications are currently in progress in our laboratory.

## Data availability

Crystallographic data for 1–7, 9a and 9b have been deposited at the Cambridge Crystallographic Data Centre (CCDC 2118417–2118424, 2128950) and can be obtained from https://www.ccdc.cam.ac.uk/structures. Other primary data (NMR and IR spectra) can be downloaded from the Zenodo repository under DOIs: https://doi.org/10.5281/zenodo.6545548 (for 1), https://doi.org/10.5281/zenodo.6545724 (for 2), https://doi.org/10.5281/zenodo.6545746 (for 3), https://doi.org/10.5281/zenodo.6545760 (for 4), https://doi.org/10.5281/zenodo.6545770 (for 5), https://doi.org/10.5281/zenodo.6545778 (for 6), https://doi.org/10.5281/zenodo.6545826 (for 7), https://doi.org/10.5281/zenodo.6545851 (for 8), https://doi.org/10.5281/zenodo.6545875 (for 9a), https://doi.org/10.5281/zenodo.6545898 (for 9b).

## Author contributions

TXS: conceptualization, experimental data collection, and analysis; KNM: computational analysis; LM: computational analysis, validation; GN: conceptualization, project administration, data analysis, validation, funding acquisition. All authors contributed to the writing, editing, and revision of the manuscript.

## Conflicts of interest

There are no conflicts to declare.

## Supplementary Material

SC-013-D2SC01798A-s001

SC-013-D2SC01798A-s002

SC-013-D2SC01798A-s003

SC-013-D2SC01798A-s004
