# Supplementary material for: CO reductive oligomerization by a divalent thulium complex and CO2-induced functionalization
Source: Chem Sci. 2022 May 9;13(25):7449–61. doi: 10.1039/d2sc01798a (PMC9241974; doi:10.1039/d2sc01798a)
Supplement: SC-013-D2SC01798A-s001 [file SC-013-D2SC01798A-s001.pdf]

Electronic Supplementary Material (ESI) for Chemical Science.
This journal is © The Royal Society of Chemistry 2022

SUPPORTING INFORMATION

CO reductive oligomerization by a divalent thulium complex and CO_2-induced functionalization

Thomas Simler,*[a] Karl N. McCabe,[b] Laurent Maron [b] and Grégory Nocton*[a]

a) LCM, CNRS, Ecole polytechnique, Institut polytechnique de Paris, Route de Saclay, 91120 Palaiseau, France. E-mail: thomas.simler@polytechnique.edu; gregory.nocton@polytechnique.edu

b) LPCNO, UMR 5215, Université de Toulouse-CNRS, INSA, UPS, Toulouse, France

Table of content

I. Synthesis and characterization S2
I.1. General considerations S2
I.2. Syntheses of the complexes S2
II. NMR spectra S10
II.1. ^{1}H NMR spectrum of **1** S10
II.2. NMR spectra of **2** S10
II.3. NMR spectra of **3** S12
II.4. NMR spectra of **4** S14
II.5. NMR spectra of **5** S15
II.6. NMR spectra of **6** S16
II.7. NMR spectra of **7** S17
II.8. ^{1}H NMR spectrum of **8** S19
II.9. NMR spectra of **9a-b** S19
III. IR spectra S23
III.1. IR spectra of complexes **2**–**9** S23
III.2. IR spectra of **2-^{13}C** and **3-^{13}C** S27
III.3. Comparison of IR spectra with and without ^{13}CO labelling S28
IV. X-ray crystallography S32
IV.1. General methods S32
IV.2. Summary of crystal data S34
IV.3. Crystal structures S37
V. DFT calculations S46
V.1. Mechanistic insights in the formation of **2** and **3** S46
V.2. Computed IR spectra of **3** and **3-^{13}C** S52
V.3. Computed coordinates S54
VI. References S107

I. Synthesis and characterization

I.1. General considerations

All air- and moisture-sensitive reactions were performed using standard Schlenk-line techniques under dry N_2 or Ar atmosphere or in argon-filled gloveboxes (MBraun). All glassware was dried at 140 °C for at least 12 h prior to use. All solvents (Et_2O, toluene, benzene, pentane, C_6D_6, tol-d_8, THF-d_8) were dried over sodium, degassed, and transferred under reduced pressure in a cold flask. TmI_2 was prepared from Tm metal and I_2 following the literature procedure for DyI_2.[1] Commercial $KN(SiMe_3)_2$ (Sigma–Aldrich, 95%) was purified by recrystallization from toluene. KCp^{ttt} was prepared by deprotonation of HCp^{ttt} with $KN(SiMe_3)_2$ in Et_2O. HCp^{ttt},[2] $[Tm(Cp^{ttt})_2I]$[3] and KC_8[4] were prepared following the published procedures. All other chemicals were obtained from commercial sources and used without further purification.

High purity CO gas (CO-N47, CO ≥ 99,997 %, < 1 ppm CO_2, < 3 ppm O_2, < 3 ppm H_2O) was purchased from Air Liquide. Isotopically enriched ^{13}CO (99.3% enrichment, CO > 99.9%, 17 ppm O_2, 14 ppm CO_2) was purchased form Eurisotop. The control of the stoichiometry in the gas addition reactions was achieved by the use of standard 5 mm NMR tubes of known volumes equipped with J. Young valves. Upon addition of a precise volume of solvent, the headspace volume is pressurized accordingly following the ideal gas law.

Elemental analyses were obtained from Mikroanalytisches Labor Pascher (Remagen, Germany). ^1H NMR spectra were recorded on a Bruker Avance III-300 MHz spectrometer and ^1H chemical shifts are given relative to TMS in ppm. Infrared (IR) spectra were recorded at room temperature under argon atmosphere on a Thermo Scientific Nicolet iS5 FTIR spectrometer equipped with an iD7 ATR-Diamond unit.

I.2. Syntheses of the complexes

Synthesis of $[Tm(Cp^{ttt})_2]$ (1)

$$TmI_2 + 2\ KCp^{ttt} \xrightarrow[\text{- 2 KI}]{Et_2O,\ \text{r.t.}} [Tm(Cp^{ttt})_2]\ \textbf{(1)}$$

This compound was best prepared from TmI_2, as described below, by slightly adapting the procedure by Nief and co-workers.[3] It could also be obtained by reduction of $[Tm(Cp^{ttt})_2I]$ with KC_8.[3]

Under Ar atmosphere, a suspension of KCp^{ttt} (500 mg, 1.83 mmol) and TmI_2 (388 mg, 0.917 mmol) in Et_2O (10 mL) was stirred for 2 days at room temperature, resulting in the gradual formation of a dark purple solution along with a white precipitate. KC_8 (*ca.* 100 mg, 0.740 mmol) was added (to ensure the reduction of Tm^{III} species that are formed by reaction with traces of TmI_3, the latter being usually present in small amounts in the synthesis of TmI_2) and the slurry was stirred for 1 h. After filtration over a syringe filter, the resulting dark purple solution was evaporated to dryness, redissolved in pentane (3–5 mL) and filtered once more. Evaporation of the solvent under vacuum afforded **1** as a dark purple solid (395 mg, 0.621 mmol, 68%). Single crystals suitable for X-ray diffraction studies were obtained upon storing a concentrated pentane solution of the complex at –40 °C.

^{1}H NMR (300 MHz, C_6D_6, 293 K): δ (ppm): 33.0 (br s, $\Delta\nu_{1/2}$ ≈ 55 Hz, 18H, ^{t}Bu), 22.5 (br s, $\Delta\nu_{1/2}$ ≈ 80 Hz, 18H, ^{t}Bu), –62.4 (br s, $\Delta\nu_{1/2}$ ≈ 600 Hz, 4H, *H*C ring system).
These data are consistent with those described in the literature.[3]

Synthesis of [Tm(Cp^{ttt})$_2$]$_2$(μ-κ(*O*):κ(*O′*)-C_2O_2) (2)

$$2\,[\mathrm{Tm(Cp^{ttt})_2}]\ \mathbf{(1)} + 2\,\mathrm{CO} \xrightarrow[\text{r.t.}]{\text{toluene}} \mathrm{(Cp^{ttt})_2Tm{-}O{-}C{\equiv}C{-}O{-}Tm(Cp^{ttt})_2}\ \mathbf{(2)}$$

In a J. Young NMR tube, **1** (82 mg, 0.13 mmol) was dissolved in toluene (0.5 mL). The resulting deep purple solution was degassed twice by freeze–pump–thaw cycles and 1.0 equiv. CO was added in the tube (0.13 mmol *i.e.* 1.6 bar in 2.0 mL headspace). Light yellow crystals suitable for X-ray diffraction studies formed over a period of 2 days at room temperature. The crystals were isolated by filtration, washed with pentane and dried under reduced pressure. Yield of the crystals: 46 mg (35 μmol), 53% (based on the metal). Anal. Calcd for $C_{70}H_{116}O_2Tm_2$ (1327.54): C, 63.33; H, 8.81. Found: C, 63.39; H, 8.81.
A very broad ^{1}H NMR spectrum was observed at room temperature in tol-*d*$_8$ (see Fig. S3), possibly the result of a fluxional behavior of the Cp^{ttt} ligands. A better resolved spectrum was obtained upon heating the sample at 80 °C. ^{1}H NMR (300 MHz, tol-*d*$_8$, 353 K): δ (ppm): 170.7 (br s, $\Delta\nu_{1/2}$ ≈ 3400 Hz), 32.0 (br s, $\Delta\nu_{1/2}$ ≈ 5200 Hz).
No ^{13}C{^{1}H} NMR spectrum could be obtained as a result of the highly paramagnetic nature of the complex.
IR (ATR): $\tilde{\nu}$ (cm^{-1}) = 2955 (m), 2904 (m), 2867 (m), 1483 (w), 1460 (m), 1387 (m), 1357 (s), 1329 (vs), 1271 (w), 1238 (s), 1197 (m), 1163 (m), 1112 (w), 1021 (w), 1002 (m), 958 (w), 918 (w), 879 (w), 851 (m), 831 (w), 821 (m), 809 (m), 775 (m), 689 (m), 679 (m), 639 (w).

Synthesis of [Tm(Cp^{ttt})$_2$]$_2$(μ-κ(*O*):κ^{2}(*C,O′*)-C_2O_3) (3)

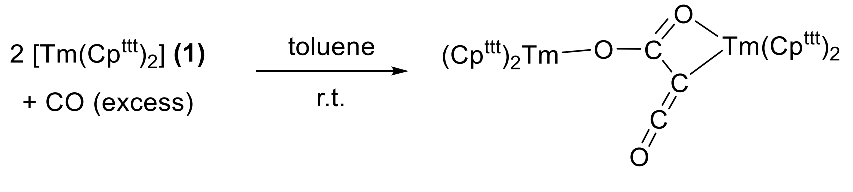

(3)

A Schlenk flask equipped with a J. Young stopper was charged with **1** (200 mg, 0.315 mmol) and toluene (5 mL) was added. The resulting deep purple solution was degassed twice by freeze–pump–thaw cycles and put under CO atmosphere (1.5 bar, excess CO). Light yellow crystals suitable for X-ray diffraction studies formed over a period of 2–3 days at room temperature. The mother liquor was removed and the crystals were washed with pentane and dried under reduced pressure. Concentration of the mother liquor afforded an additional amount of **3** as a microcrystalline powder. Total yield: 109 mg (80 μmol), 51% (based on the metal). Anal. Calcd for $C_{71}H_{116}O_3Tm_2$ (1355.55): C, 62.91; H, 8.63. Found: C, 63.05; H, 8.68.

A very broad ^{1}H NMR spectrum was observed at room temperature in C_6D_6 (see Fig. S6), possibly the result of a fluxional behavior of the Cp^{ttt} ligands. A better resolved spectrum was obtained upon heating the sample at 80 °C. ^{1}H NMR (300 MHz, C_6D_6, 353 K): δ (ppm): 184.7 (br s, $\Delta\nu_{1/2} \approx$ 3200 Hz), 153.3 (br s, $\Delta\nu_{1/2} \approx$ 3000 Hz), 32.1 (br s, $\Delta\nu_{1/2} \approx$ 3200 Hz), -32.6 (br s, $\Delta\nu_{1/2} \approx$ 1500 Hz).
No $^{13}C\{^{1}H\}$ NMR spectrum could be obtained as a result of the highly paramagnetic nature of the complex. IR (ATR): $\tilde{\nu}$ (cm^{-1}) = 2957 (m), 2903 (m), 2868 (m), 2205 (w), 2066 (vs), 2015 (w), 1979 (w), 1600 (s), 1467 (s), 1449 (vs), 1436 (vs), 1395 (m), 1359 (s), 1269 (s), 1257 (s), 1238 (s), 1203 (m), 1166 (m), 1110 (w), 1024 (w), 1001 (m), 961 (w), 927 (w), 872 (w), 826 (s), 814 (m), 777 (m), 757 (m), 717 (w), 682 (m).

Synthesis of ^{13}CO labelled complexes

The ^{13}C labelled complex $[Tm(Cp^{ttt})_2]_2(\mu\text{-}\kappa(O)\text{:}\kappa(O')\text{-}^{13}C_2O_2)$ (**2-^{13}C**) was prepared following the procedure for the synthesis of **2** starting with **1** (40 mg, 0.063 mmol) and ^{13}CO (0.063 mmol *i.e.* 0.76 bar in 2.0 mL headspace). Yield of the crystals: 6.0 mg (4.5 µmol), 14% (based on the metal). IR (ATR): $\tilde{\nu}$ (cm^{-1}) = 2955 (s), 2903 (m), 2867 (m), 1549 (w), 1482 (w), 1460 (m), 1387 (m), 1357 (s), 1304 (vs), 1238 (vs), 1201 (m), 1163 (m), 1113 (w), 1021 (w), 1002 (m), 958 (w), 918 (w), 851 (s), 834 (w), 821 (s), 809 (m), 775 (m), 690 (m), 679 (m), 674 (m), 639 (w).
The ^{13}C labelled complex $[Tm(Cp^{ttt})_2]_2(\mu\text{-}\kappa(O)\text{:}\kappa^2(C,O')\text{-}^{13}C_3O_3)$ (**3-^{13}C**) was prepared following the procedure for the synthesis of **3** starting with **1** (100 mg, 0.157 mmol) and excess ^{13}CO (1.3 bar). Yield of the crystals: 31 mg (23 µmol), 29%. IR (ATR): $\tilde{\nu}$ (cm^{-1}) = 2956 (m), 2902 (m), 2868 (m), 2003 (vs), 1984 (w), 1556 (w), 1489 (w), 1464 (m), 1408 (vs), 1393 (vs), 1359 (s), 1269 (w), 1241 (s), 1166 (m), 1110 (w), 1051 (w), 1024 (w), 1000 (m), 960 (w), 927 (w), 826 (s), 814 (m), 777 (w), 731 (m), 982 (m).
For both complexes, no suitable $^{13}C\{^{1}H\}$ NMR spectra could be obtained as a result of their highly paramagnetic nature. The IR spectra of the ^{13}CO-labelled complexes as well as the IR spectrum of the product from the reaction of **2** with excess ^{13}CO are displayed in section III.3.

Synthesis of $[Tm(Cp^{ttt})_2]_2(\mu\text{-}\kappa(O)\text{:}\kappa^2(O',O'')\text{-}CO_3)$ (4)

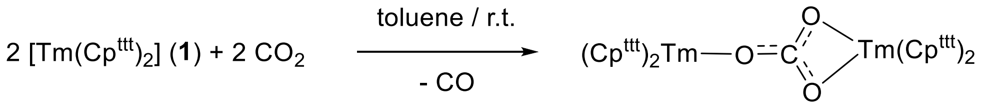

(**4**)

In a J. Young NMR tube, **1** (40 mg, 0.063 mmol) was dissolved in toluene (0.5 mL). The resulting deep purple solution was degassed twice by freeze–pump–thaw cycles and CO_2 was added in the tube (0.13 mmol *i.e.* 1.6 bar in 2.0 mL headspace). The solution turned brown and a colorless microcrystalline powder precipitated in the tube. The resulting mixture was transferred in a vial in the glovebox, layered with pentane and stored at –40 °C, leading to the formation of colorless crystals suitable for X-ray diffraction studies. The resulting solid was isolated by filtration, washed with a small amount of cold pentane and dried under reduced pressure. Yield: 30 mg (23 µmol), 72% (based on the metal).

Anal. Calcd for $C_{69}H_{116}O_3Tm_2$ (1331.53): C, 62.24; H, 8.78. Found: C, 62.65; H, 9.14. These values fit better with $C_{69}H_{116}O_3Tm_2\cdot(0.5\ C_5H_{12})$ (1366.81): C, 62.79; H, 8.99, the compound crystallizing with half a molecule of pentane in the asymmetric unit (see X-ray crystallography section).

^{1}H NMR (300 MHz, tol-d_8, 293 K): δ (ppm): 347.2 (br s, $\Delta\nu_{1/2} \approx$ 6000 Hz), 243.0 (br s, $\Delta\nu_{1/2} \approx$ 2400 Hz), 221.5 (br s, $\Delta\nu_{1/2} \approx$ 2300 Hz), 93.9 (br s), 79.7 (br s), –58.6 (br s, $\Delta\nu_{1/2} \approx$ 2200 Hz). No ^{13}C{^{1}H} NMR spectrum could be recorded as a result of the highly paramagnetic nature of the complex. IR (ATR): $\tilde{\nu}$ (cm^{-1}) = 2956 (s), 2916 (m), 2868 (m), 1489 (m), 1471 (s), 1451 (vs), 1430 (vs), 1392 (s), 1358 (s), 1238 (s), 1209 (m), 1167 (m), 1160 (m), 1113 (w), 1099 (m), 1001 (m), 961 (w), 840 (m), 824 (s), 816 (s), 781 (m), 683 (m), 674 (m).

Synthesis of [Tm(Cp^{ttt})$_2$]$_2$(μ-κ^2(*O*):κ^2(*O*)-C_4O_5) (5)

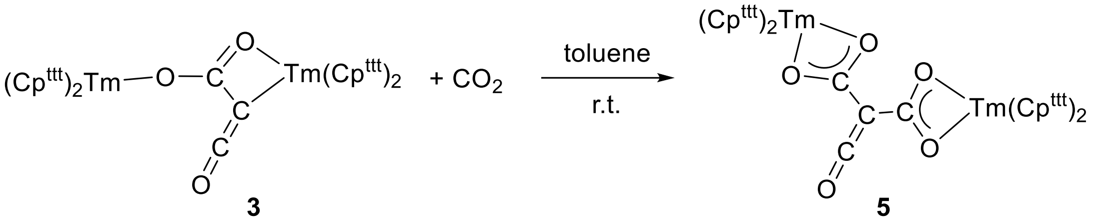

3 **5**

In a J. Young NMR tube, **3** (55 mg, 0.041 mmol) was dissolved in tol-d_8 (0.5 mL). The solution was degassed twice by freeze–pump–thaw cycles and put under CO_2 atmosphere (1.3 bar, excess CO_2). Analysis by paramagnetic ^{1}H NMR spectroscopy immediately after the addition of CO_2 indicated complete conversion of **3**. The solution was concentrated under reduced pressure and let stand a room temperature for 2-3 days, leading to the formation of light yellow crystals suitable for X-ray diffraction studies. The crystals were washed with a small amount of cold pentane and dried under vacuum. Yield of the crystals: 47 mg (0.034 mmol), 82%. Anal. Calcd for $C_{72}H_{116}O_5Tm_2$ (1399.56): C, 61.79; H, 8.35. Found: C, 62.65; H, 7.93. Although these results are outside the range viewed as establishing analytical purity, they are provided to illustrate the best values obtained to date.

^{1}H NMR (300 MHz, tol-d_8, 293 K): δ (ppm): 304.2 (br s, $\Delta\nu_{1/2} \approx$ 6500 Hz), 271.9 (br s), 231.8 (br s, $\Delta\nu_{1/2} \approx$ 8000 Hz), 116.8 (br s), 68.1 (br s), -25.6 (br s, $\Delta\nu_{1/2} \approx$ 8500 Hz).

^{1}H NMR (300 MHz, tol-d_8, 333 K): δ (ppm): 196.9 (br s, $\Delta\nu_{1/2} \approx$ 10 000 Hz), 50.9 (br s, $\Delta\nu_{1/2} \approx$ 17 000 Hz), –16.2 (br s, $\Delta\nu_{1/2} \approx$ 12 000 Hz).

No ^{13}C{^{1}H} NMR spectrum could be obtained as a result of the highly paramagnetic nature of the complex. IR (ATR): $\tilde{\nu}$ (cm^{-1}) = 2957 (m), 2902 (m), 2868 (m), 2157 (s), 2146 (m), 1572 (s), 1491 (w), 1442 (m), 1415 (vs), 1395 (s), 1360 (s), 1239 (s), 1199 (w), 1166 (m), 1157 (m), 1111 (w), 1001 (m), 961 (w), 872 (w), 829 (s), 797 (vs), 683 (m), 672 (m).

Synthesis of $[Tm(Cp^{ttt})_2]_3\{\mu_3\text{-}\kappa^2(O)\text{:}\kappa^2(O)\text{:}\kappa^2(O)\text{-}H_3CC_3(CO_2)_3\}$ (6)

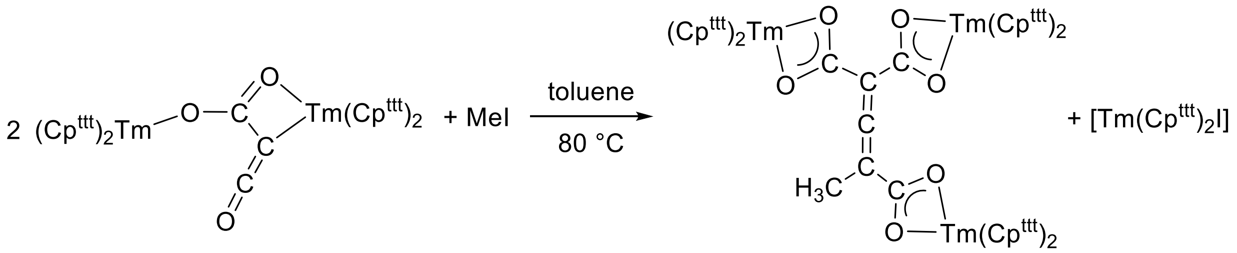

3 **6**

In a J. Young NMR tube, **3** (50 mg, 0.037 mmol) was dissolved in tol-d_8 (0.5 mL) and MeI (23 μL, 52 mg, 0.37 mmol) was added. The resulting solution was heated at 80 °C and the reaction was monitored by paramagnetic ^{1}H NMR spectroscopy, showing the gradual formation of $[Tm(Cp^{ttt})_2I]$ along with another species (see Fig. S15). After 3 days, total conversion of **3** was evidenced and the solution was evaporated to dryness. The resulting solid was carefully washed with 3 times 2.5 mL pentane and dried under reduced pressure, affording **6** as a light yellow powder. Yield: 20 mg (9.6 μmol), 52% (based on the metal). Crystals suitable for X-ray diffraction studies were obtained by slowly cooling a saturated solution of **6** in boiling toluene.

Anal. Calcd for $C_{109}H_{177}O_6Tm_3$ (2090.37): C, 62.63; H, 8.53. Found: C, 62.18; H, 8.10.

^{1}H NMR (300 MHz, tol-d_8, 293 K): δ (ppm): 234.6 (br s, $\Delta\nu_{1/2}$ ≈ 900 Hz), 219.2 (br s, $\Delta\nu_{1/2}$ ≈ 850 Hz), 192.1 (br s, $\Delta\nu_{1/2}$ ≈ 600 Hz), 166.0 (br s, $\Delta\nu_{1/2}$ ≈ 700 Hz), 128.0 (br s, $\Delta\nu_{1/2}$ ≈ 700 Hz), –3.8 (br s, $\Delta\nu_{1/2}$ ≈ 800 Hz), –22.6 (br s, $\Delta\nu_{1/2}$ ≈ 600 Hz), –36.0 (br s, $\Delta\nu_{1/2}$ ≈ 900 Hz), –252.6 (br s, $\Delta\nu_{1/2}$ ≈ 600 Hz). Only the most intense resonances are reported.

No ^{13}C{^{1}H} NMR spectrum could be obtained as a result of the highly paramagnetic nature of the complex.

IR (ATR): $\tilde{\nu}$ (cm^{-1}) = 2957 (m), 2902 (m), 2870 (w), 2164 (w), 1938 (w), 1583 (vs), 1559 (m), 1534 (m), 1511 (w), 1491 (w), 1456 (m), 1407 (s), 1394 (s), 1360 (s), 1325 (w), 1238 (s), 1201 (w), 1167 (m), 1095 (w), 1001 (m), 960 (w), 828 (s), 817 (s), 803 (m), 790 (m), 683 (m), 673 (m).

Synthesis of [Tm(Cp^{ttt})$_2$]$_2${μ-κ(*O*):κ^2(*O*′,*O*″)-$C_4O_4(SiMe_3)_2$} (7)

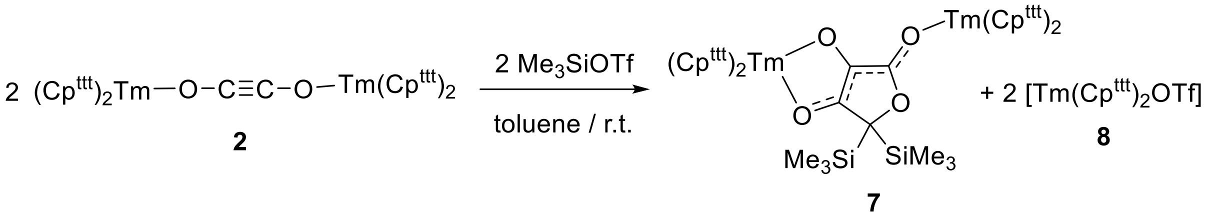

In a J. Young NMR tube, **2** (40 mg, 0.030 mmol) was dissolved in tol-*d*$_8$ (0.5 mL) and Me_3SiOTf (55 μL, 67 mg, 0.30 mmol) was added. The reaction was monitored by paramagnetic ^{1}H NMR spectroscopy, indicating complete conversion of **2** after 15 h at room temperature with formation of [Tm(Cp^{ttt})$_2$OTf] (**8**) along with another species (see Fig. S18). All volatiles were removed under reduced pressure and the solid residue was redissolved in pentane (2 mL). Yellow crystals suitable for X-ray diffraction studies formed over a period of 10–15 days upon letting the solution stand at room temperature. The crystals were washed with three times 2.5 mL pentane and dried under reduced pressure.

Yield of the crystals: 14 mg (9.2 μmol), 61% (based on the OCCO ligand). Anal. Calcd for $C_{78}H_{134}O_4Si_2Tm_2$ (1529.94): C, 61.23; H, 8.83. Found: C, 60.50; H, 8.17. Although these results are outside the range viewed as establishing analytical purity, they are provided to illustrate the best values obtained to date.

^{1}H NMR (300 MHz, tol-*d*$_8$, 293 K): δ (ppm): 526.8, 380.1, 271.2, 162.3, 139.0, 132.6, 122.1, 90.4, 64.5, 8.8, -31.9, -36.2, -81.3, -94.7, -106.3, -394.8, -576.4, -607.6, -670.6.

No ^{13}C{^{1}H} NMR spectrum could be obtained as a result of the highly paramagnetic nature of the complex.

IR (ATR): $\tilde{\nu}$ (cm^{-1}) = 2954 (m), 2915 (m), 2869 (m), 1639 (s), 1453 (s), 1432 (vs), 1394 (m), 1372 (m), 1359 (s), 1289 (m), 1261 (w), 1240 (s), 1204 (w), 1151 (vs), 1110 (w), 1082 (m), 1025 (w), 1001 (m), 960 (w), 875 (s), 828 (vs), 805 (s), 781 (w), 769 (w), 755 (w), 711 (w), 683 (m), 670 (m).

Synthesis of [Tm(Cp^{ttt})$_2$OTf] (8)

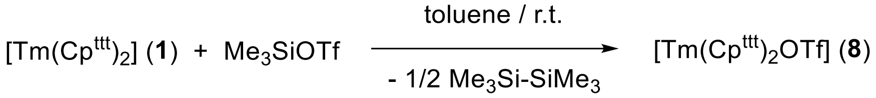

In a J. Young NMR tube, **1** (62 mg, 0.098 mmol) was dissolved in tol-*d*$_8$ (0.5 mL). Me_3SiOTf (21 μL, 26 mg, 0.12 mmol) was added and the resulting solution immediately turned from dark purple to light yellow. The solution was evaporated to dryness and the solid residue redissolved in pentane (0.5 mL). A light yellow microcrystalline solid was obtained upon storing the pentane solution at –40 °C. Yield: 32 mg (41 μmol), 42%. Anal. Calcd for $C_{35}H_{58}F_3O_3STm$ (784.83): C, 53.56; H, 7.45. Found: C, 51.88; H, 7.34. Although these results are outside the range viewed as establishing analytical purity, they are provided to illustrate the best values obtained to date. ^{1}H NMR (300 MHz, tol-*d*$_8$, 293 K): δ (ppm): 305.7 (br s, $\Delta\nu_{1/2}$ ≈ 2800 Hz, 9H, ^{t}Bu), 97.8 (br s, $\Delta\nu_{1/2}$ ≈ 1400 Hz, 9H, ^{t}Bu), -31.1 (br s, $\Delta\nu_{1/2}$ ≈ 900 Hz, 9H, ^{t}Bu) (the *H*C ring system resonances could not be detected). No ^{13}C{^{1}H} NMR spectrum could be obtained as a result of the highly paramagnetic

nature of the complex. IR (ATR): $\tilde{\nu}$ (cm^{-1}) = 2956 (s), 2904 (m), 2869 (m), 1460 (m), 1390 (w), 1359 (s), 1339 (s), 1238 (s), 1214 (s), 1196 (vs), 1165 (s), 1143 (w), 1111 (w), 1055 (s), 1010 (vs), 959 (m), 890 (w), 828 (s), 783 (w), 759 (w), 684 (m), 645 (vs), 633 (s).

Synthesis of [Tm$_2$(Cp^{ttt})$_3${μ$_3$-C$_7$H$_7$(C$_4$O$_6$)}]$_2$ (9a)

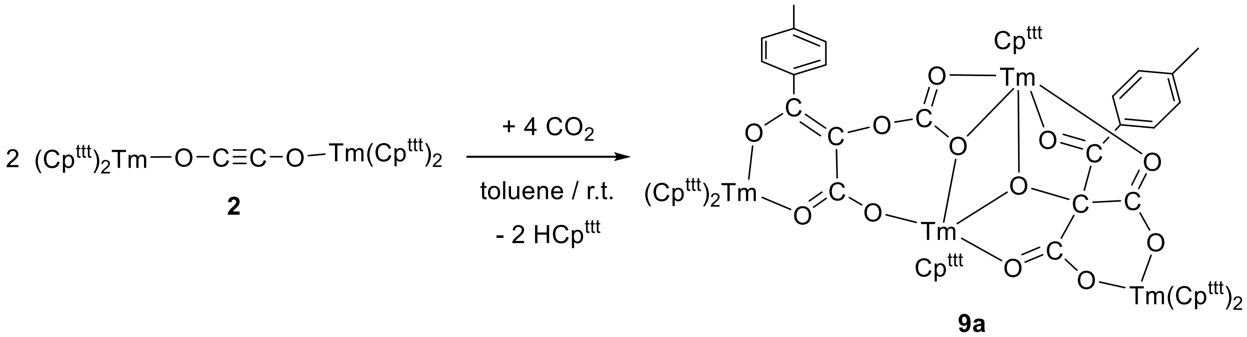

A Schlenk flask equipped with a J. Young stopper was charged with **2** (40 mg, 0.030 mmol) and toluene (2 mL) was added. The solution was degassed twice by freeze–pump–thaw cycles and put under CO_2 atmosphere (1.3 bar, excess CO_2). After one day, the solution was evaporated to dryness and the solid residue was redissolved in pentane (2–3 mL). The pentane solution was concentrated to *ca.* 0.5 mL and let stand at room temperature for 2–3 days leading to the formation of orange-red crystals suitable for X-ray diffraction studies. The mother liquor was removed and the crystals were washed with a small amount of cold pentane and dried under reduced pressure. Yield of the crystals: 27 mg (11 µmol), 71% (based on the metal). Anal. Calcd for $C_{124}H_{188}O_{12}Tm_4$ (2546.55): C, 58.48; H, 7.44. Found: C, 58.33; H, 6.44. Although the hydrogen value is outside the range viewed as establishing analytical purity, these results are provided to illustrate the best values obtained to date.

^{1}H NMR (300 MHz, tol-*d*$_8$, 293 K): δ (ppm): 248.8, 236.4, 231.9, 209.8, 204.3, 192.3, 188.0, 180.5, 86.4 (*p*-tol), 29.0, 5.0 (*p*-tol), -17.5 (*p*-tol), -22.6 (*p*-tol), -23.9, -25.8 (*p*-tol), -39.6, -52.9 (*p*-tol), -78.9, -118.6, -281.5, -348.8. The assignment of the *p*-tol resonances was possible by comparison with the spectrum of the deuterium labelled complex [Tm$_2$(Cp^{ttt})$_3${C$_7$D$_7$(C$_4$O$_6$)}]$_2$ (**9a-^{2}H**) prepared by the same procedure using tol-*d*$_8$ instead of protio-toluene as solvent (see Fig. S21–S22).

No ^{13}C{^{1}H} NMR spectrum could be obtained as a result of the highly paramagnetic nature of the complex. IR (ATR): $\tilde{\nu}$ (cm^{-1}) = 2955 (m), 2903 (m), 2869 (m), 1638 (vs), 1585 (s), 1552 (s), 1515 (w), 1483 (m), 1459 (m), 1427 (m), 1388 (w), 1360 (s), 1298 (s), 1254 (m), 1239 (s), 1188 (m), 1167 (s), 1076 (w), 1021 (w), 1002 (m), 960 (w), 936 (w), 920 (w), 854 (w), 826 (s), 816 (m), 802 (m), 792 (w), 773 (s), 720 (w), 682 (m).

The formation of HCp^{ttt} as a by-product was confirmed by ^{1}H NMR analysis of the volatiles upon reaction of **2** with CO_2 in toluene. In a typical control experiment, after one day of reaction, all volatiles were carefully transferred to a J. Young NMR tube by trap-to-trap distillation. The solvent was evaporated under vacuum at room temperature and the oily residue was redissolved in tol-*d*$_8$. Analysis by ^{1}H NMR spectroscopy revealed signals corresponding to HCp^{ttt} (see Fig. S23).

Synthesis of [$Tm_2(Cp^{ttt})_3\{\mu_3\text{-}C_6H_5(C_4O_6)\}]_2$ (9b)

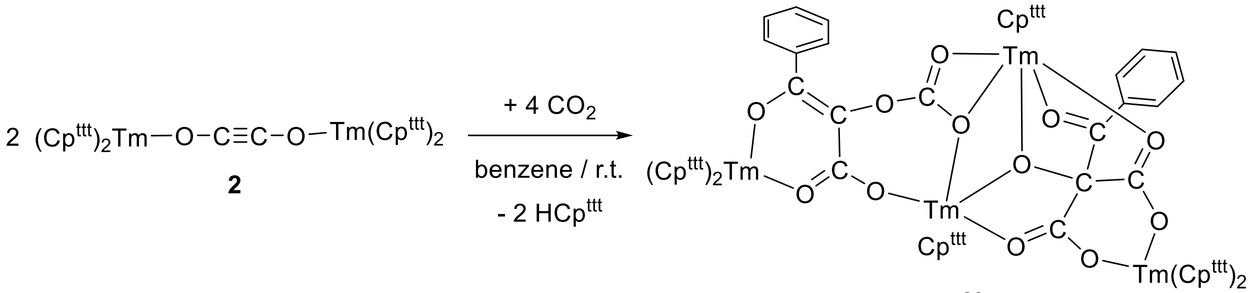

Complex **9b** was synthesized following the procedure for the synthesis of **9a** starting with **2** (40 mg, 0.030 mmol) and using benzene instead of toluene as solvent. Yield of the crystals: 21 mg (8 µmol), 54% (based on the metal).

^{1}H NMR (300 MHz, C_6D_6, 293 K): δ (ppm): 246.2, 231.4, 211.0, 208.3, 189.7, 176.4, 88.0, 28.5, -18.0, -29.3, -30.2, -32.4, -39.0, -54.2, -79.1, -117.8.

No $^{13}C\{^1H\}$ NMR spectrum could be obtained as a result of the highly paramagnetic nature of the complex.

IR (ATR): $\tilde{\nu}$ (cm^{-1}) = 2954 (m), 2902 (m), 2869 (m), 1639 (vs), 1590 (s), 1559 (s), 1503 (w), 1482 (w), 1447 (w), 1423 (m), 1387 (w), 1360 (s), 1296 (m), 1240 (s), 1166 (m), 1076 (w), 1002 (m), 960 (w), 919 (w), 852 (w), 825 (s), 816 (m), 791 (m), 776 (m), 750 (w), 733 (w), 695 (m), 674 (m), 661 (w), 632 (w).

II. NMR spectra

II.1. ^{1}H NMR spectrum of 1

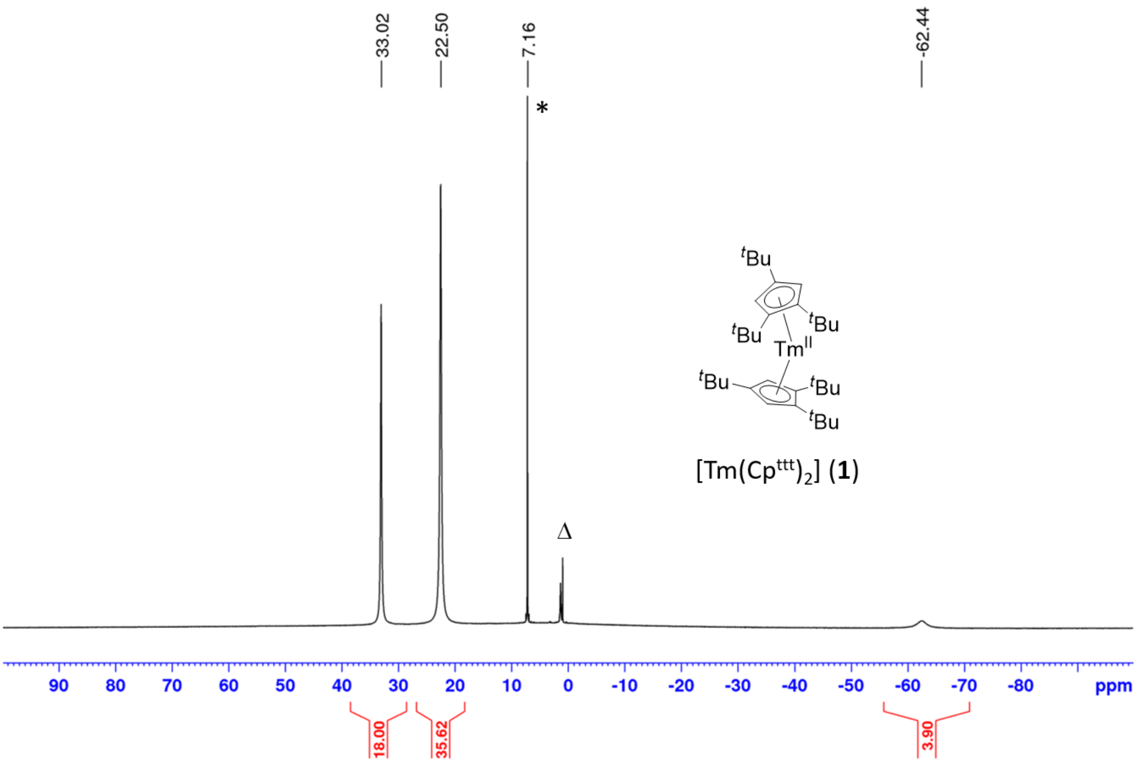

Fig. S1. ^{1}H NMR (300 MHz, C_6D_6, 293 K) spectrum of **1** (residual protio solvent signal at δ 7.16 (*)). Traces of H grease impurities can be detected at δ 0.9-1.5 ppm (Δ).

II.2. NMR spectra of 2

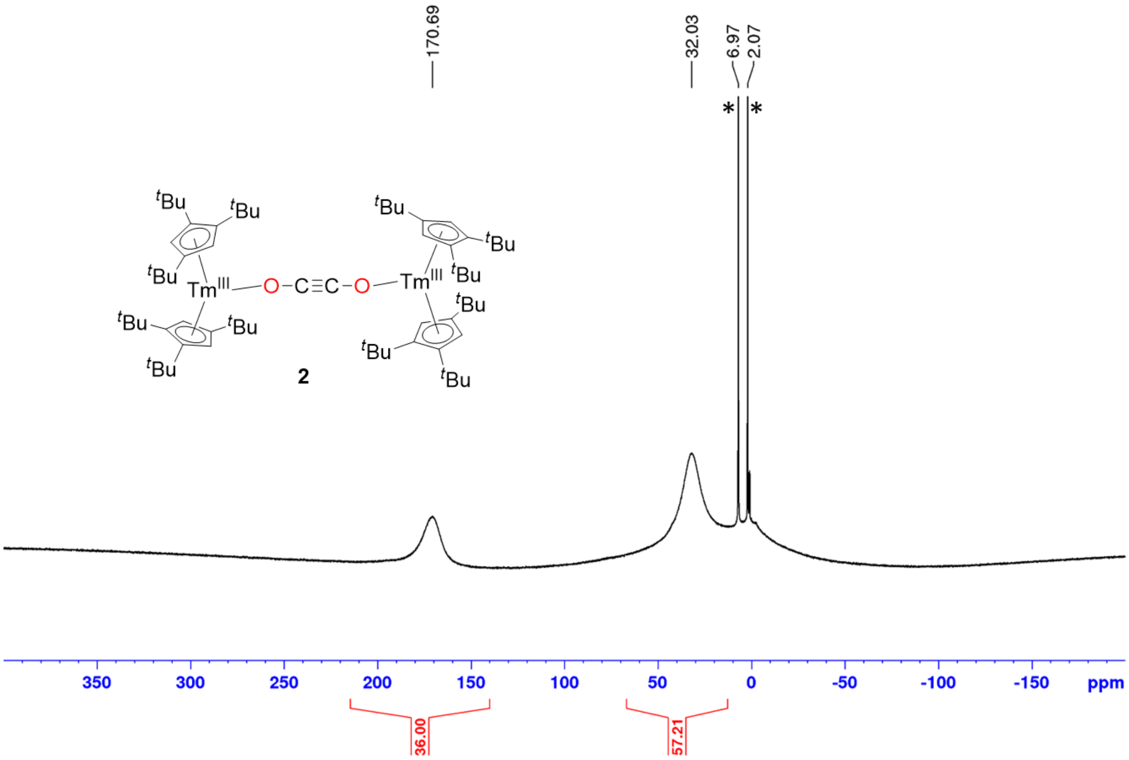

Fig. S2. ^{1}H NMR (300 MHz, tol-d_8, 353 K) spectrum of **2** (residual solvent signals at δ 6.97 and 2.07 (*)).

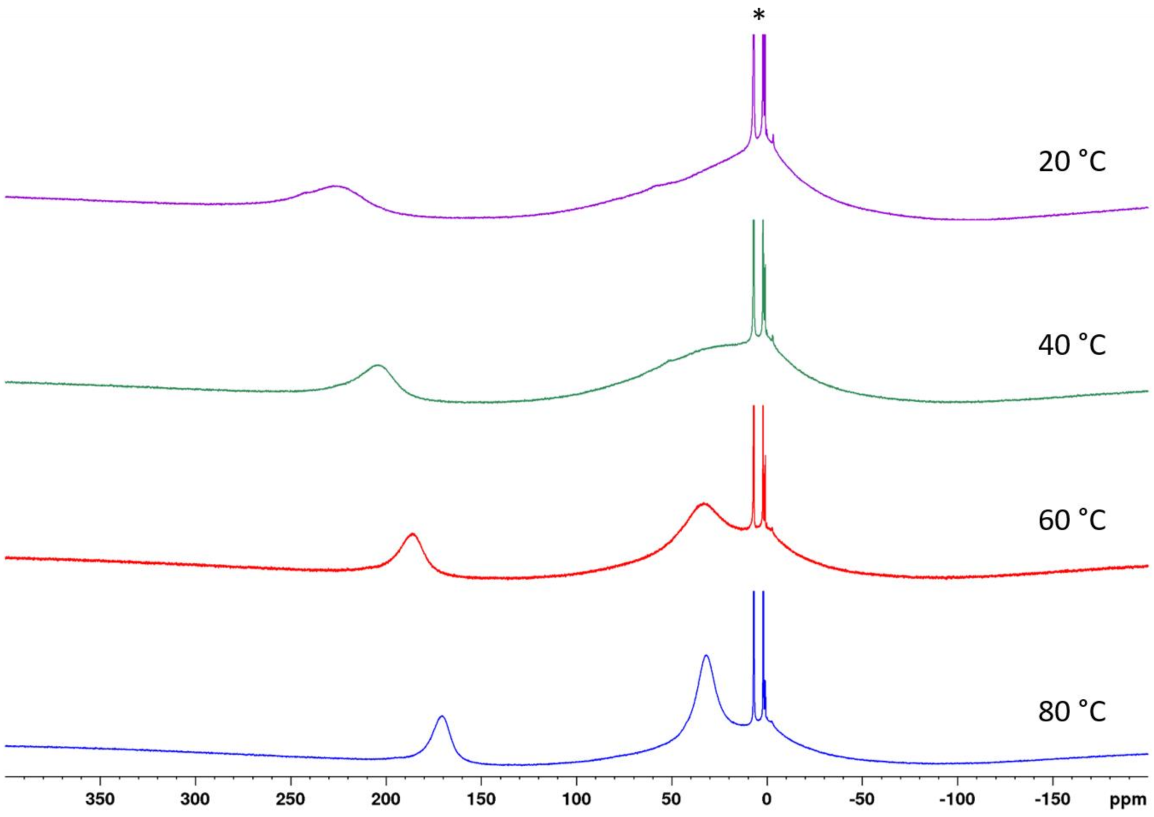

Fig. S3. ^{1}H NMR (300 MHz, tol-d_8) spectra of **2** at variable temperatures (residual protio solvent signals assigned with *).

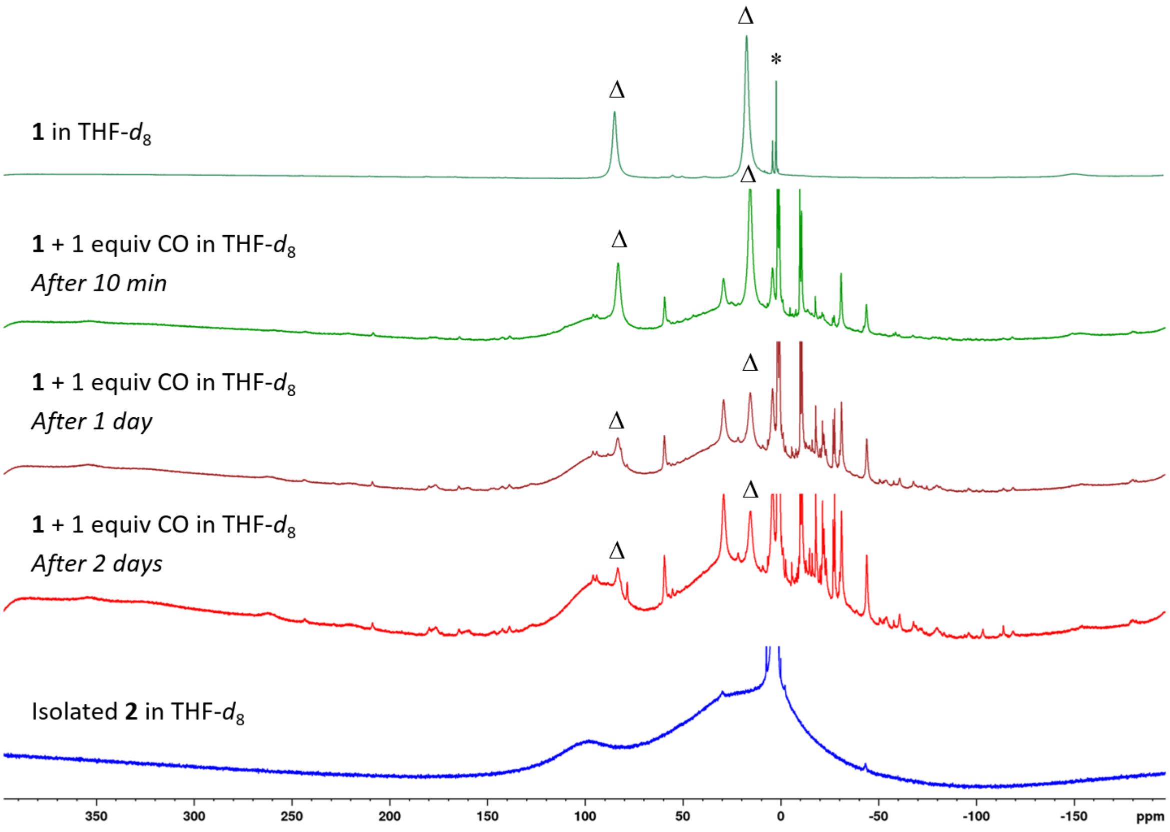

Fig. S4. ^{1}H NMR (300 MHz, THF-d_8, 293 K) monitoring of the reaction of **1** (main signals assigned with Δ) in THF-d_8 with 1 equiv. CO and comparison with the spectrum of isolated **2** in THF-d_8 (solvent signals assigned with *).

II.3. NMR spectra of 3

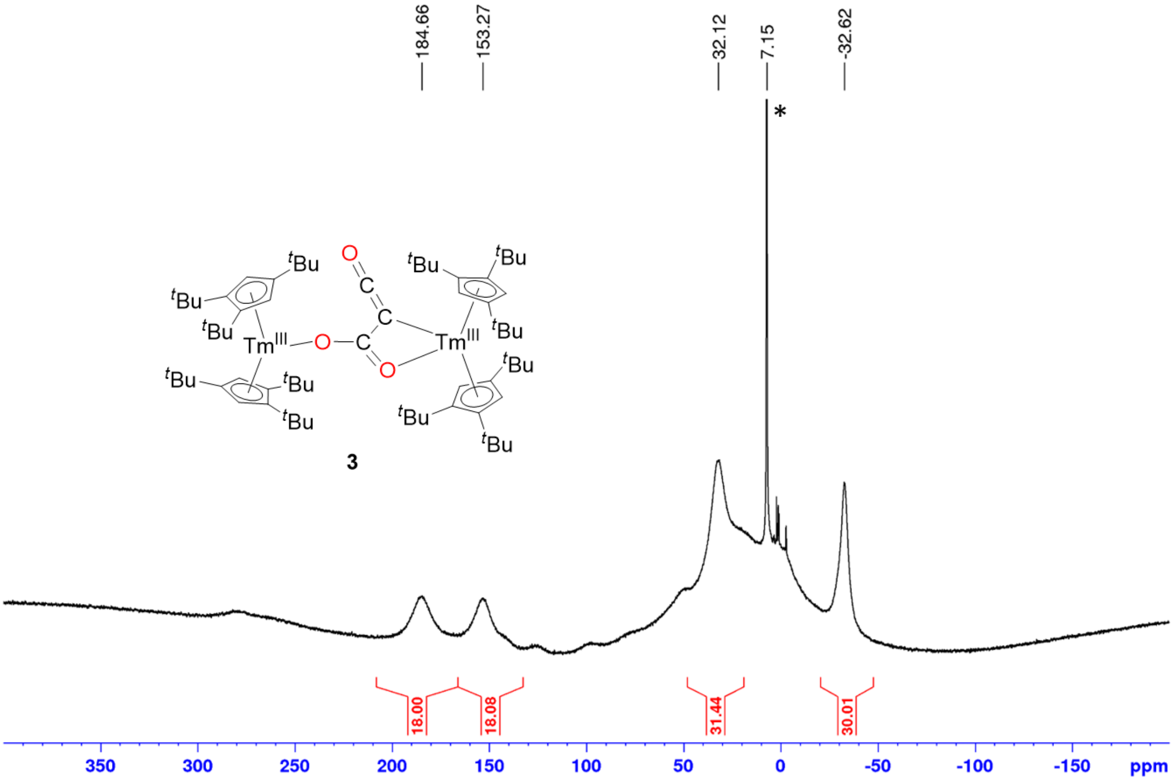

Fig. S5. ^{1}H NMR (300 MHz, C_6D_6, 353 K) spectrum of **3** (residual protio solvent signal at δ 7.15 (*)).

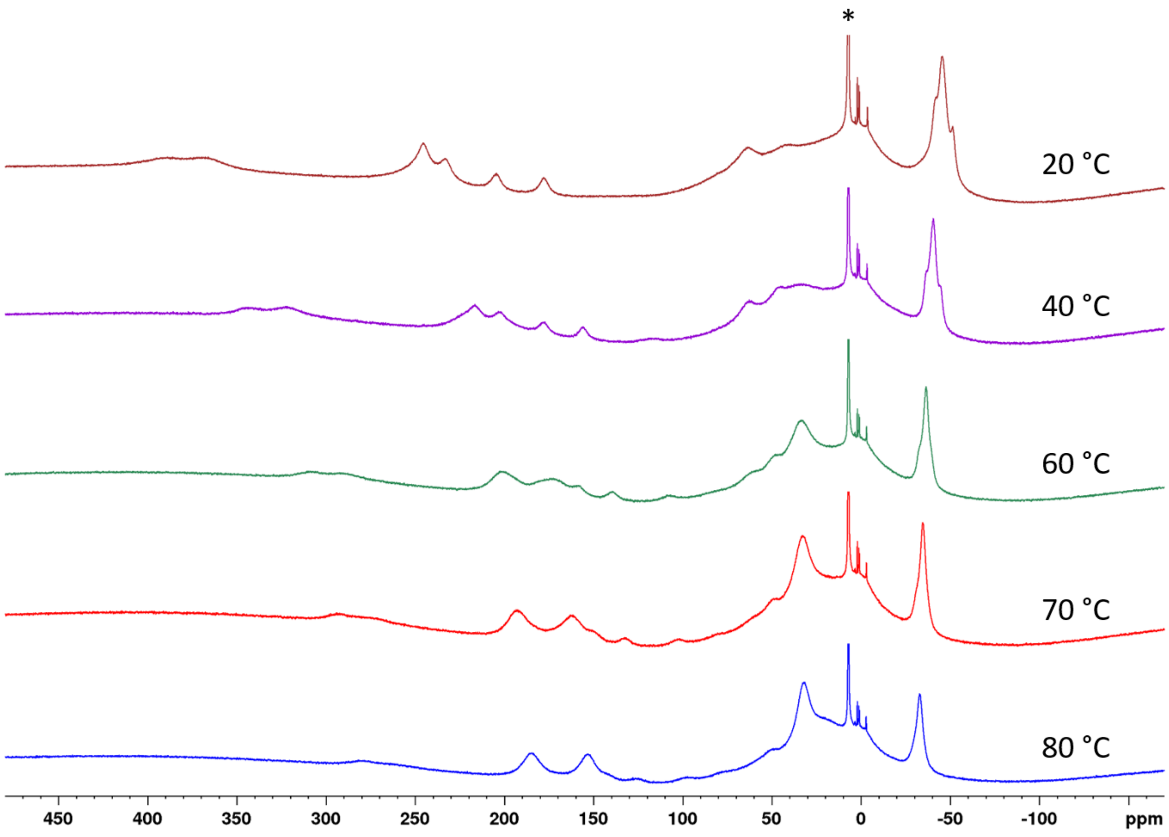

Fig. S6. ^{1}H NMR (300 MHz, C_6D_6) spectra of **3** at variable temperatures (solvent signal assigned with *).

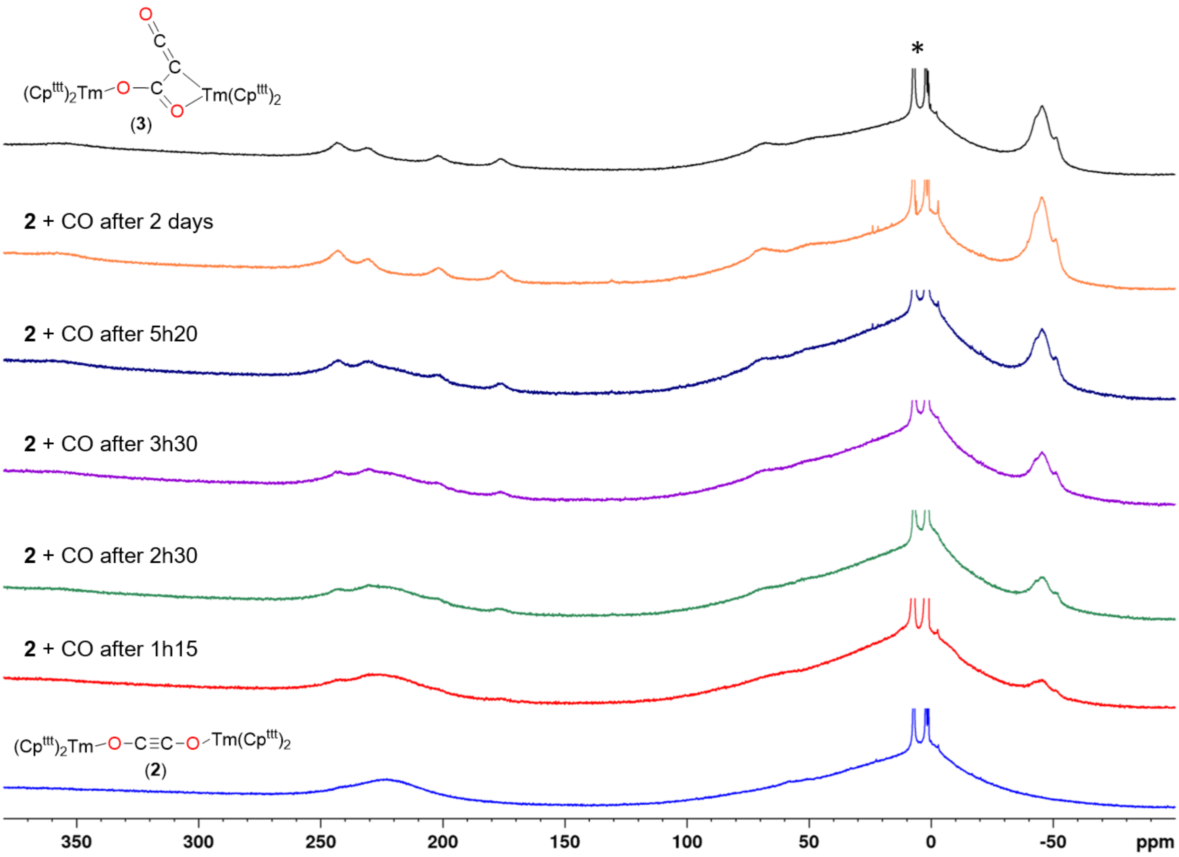

Fig. S7. ^{1}H NMR (300 MHz, tol-d_8) spectra of the evolution of **2** into **3** at room temperature upon addition of CO (solvent signals assigned with *).

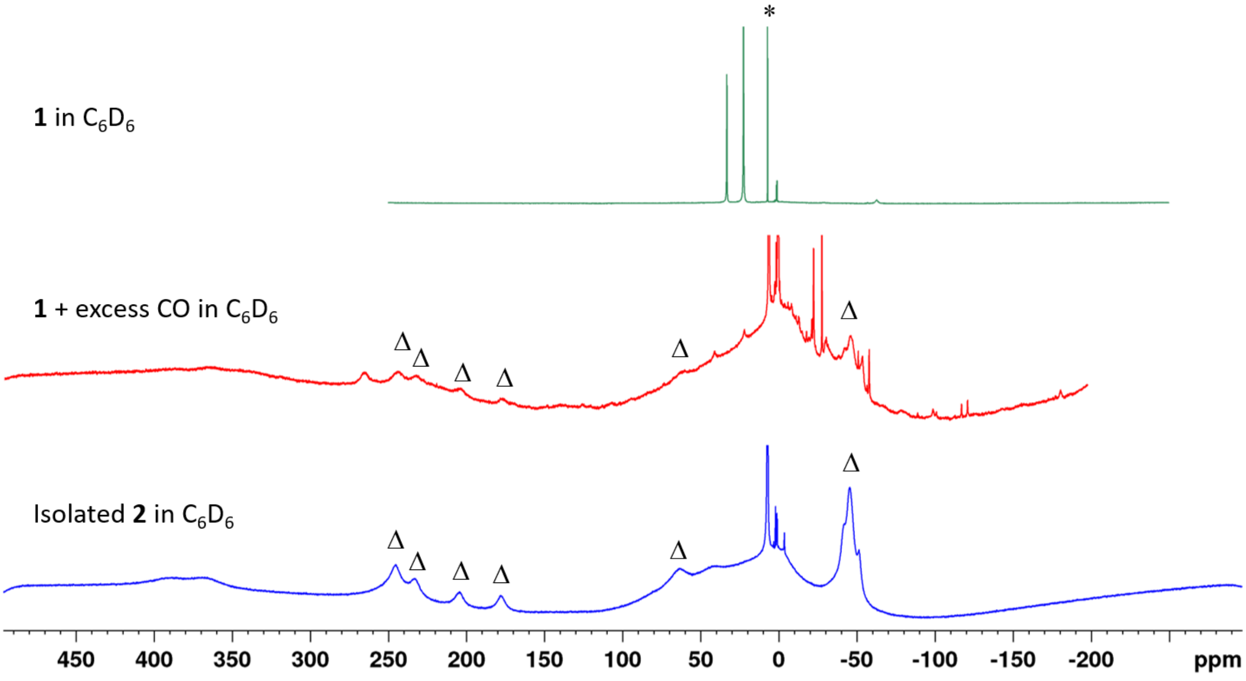

Fig. S8. ^{1}H NMR (300 MHz, C_6D_6, 293 K) spectrum of the reaction of **1** with excess CO in C_6D_6 (solvent signal assigned with *; the main signals corresponding to **2** have been assigned with Δ).

II.4. NMR spectra of 4

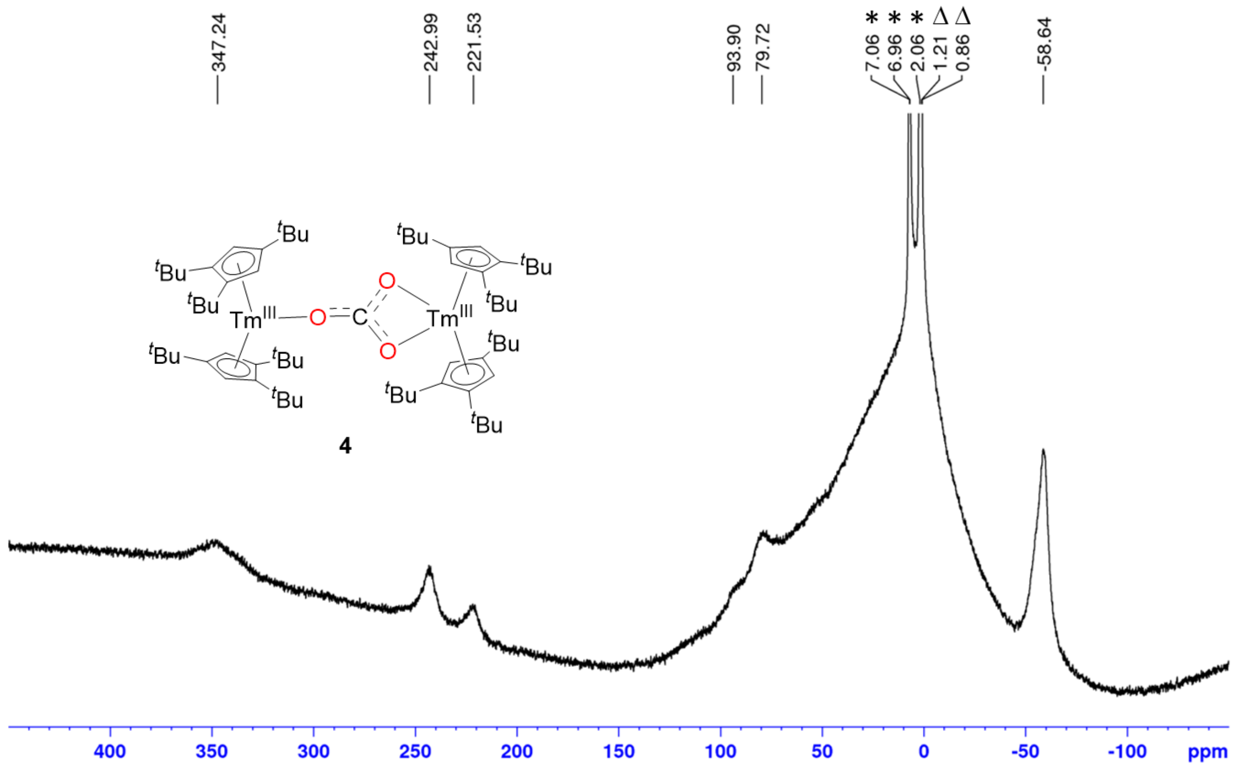

Fig. S9. ^{1}H NMR (300 MHz, tol-d_8, 293 K) spectrum of **4** (residual protio solvent signals at δ 7.06, 6.96 and 2.06 (*) and traces of pentane originating from the crystals appearing at δ 1.21 and 0.86 (Δ)).

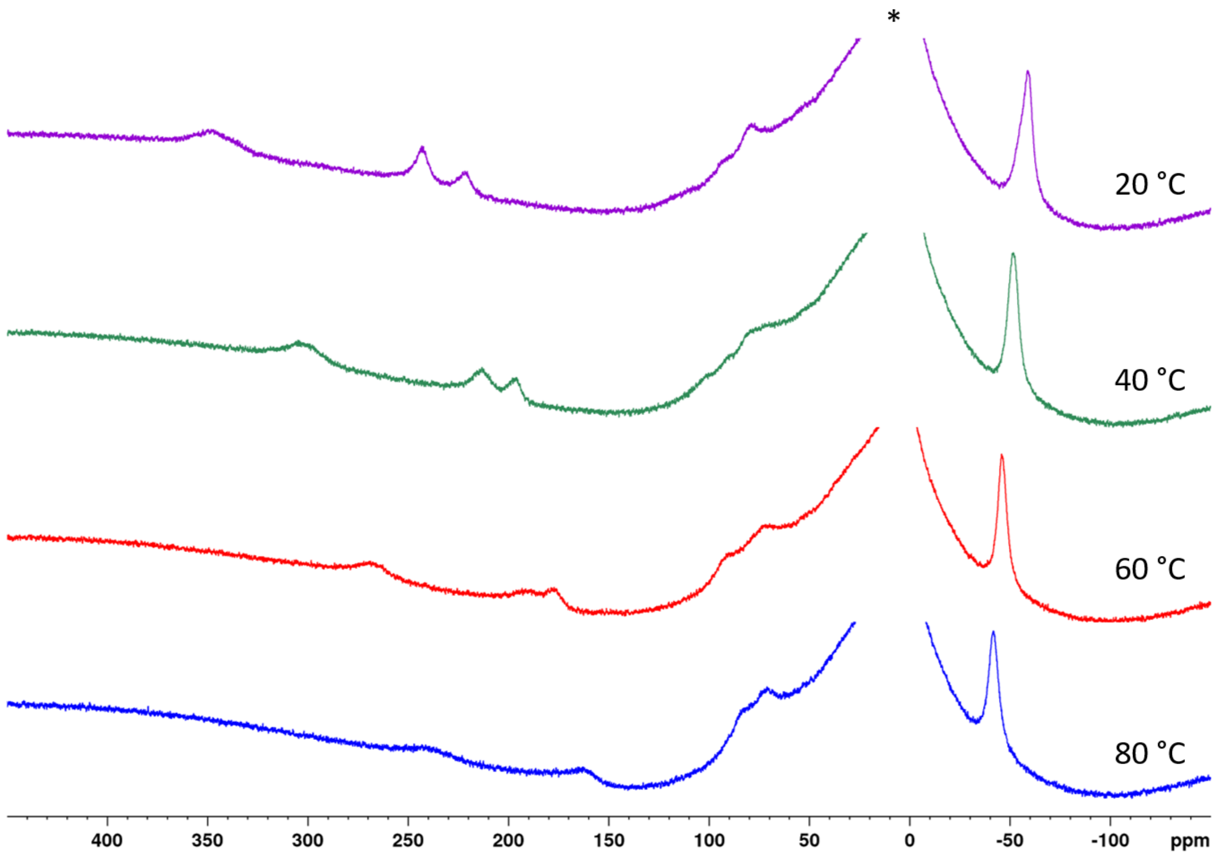

Fig. S10. ^{1}H NMR (300 MHz, tol-d_8) spectra of **4** at variable temperatures (residual protio solvent signals assigned with *).

II.5. NMR spectra of 5

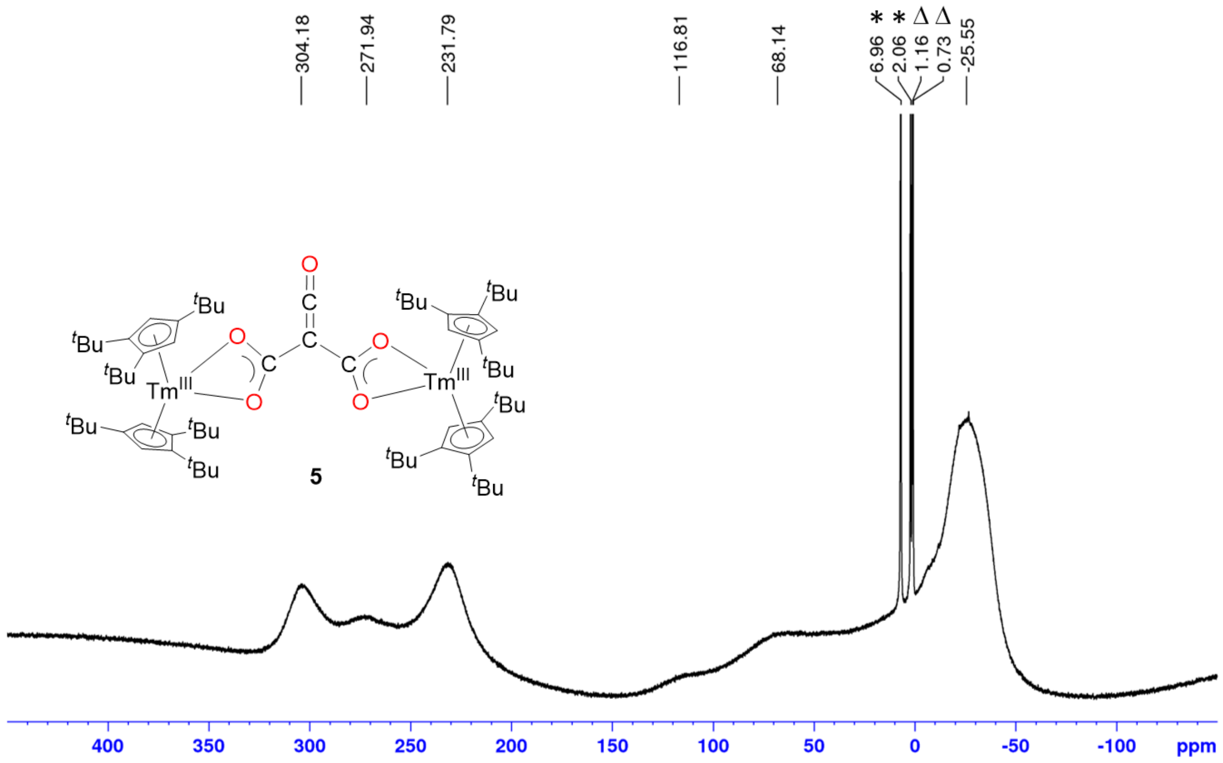

Fig. S11. ^{1}H NMR (300 MHz, tol-d_8, 293 K) spectrum of **5** (residual protio solvent signals at δ 6.96 and 2.06 (*) and traces of pentane originating from the crystals appearing at δ 1.16 and 0.73 (Δ)).

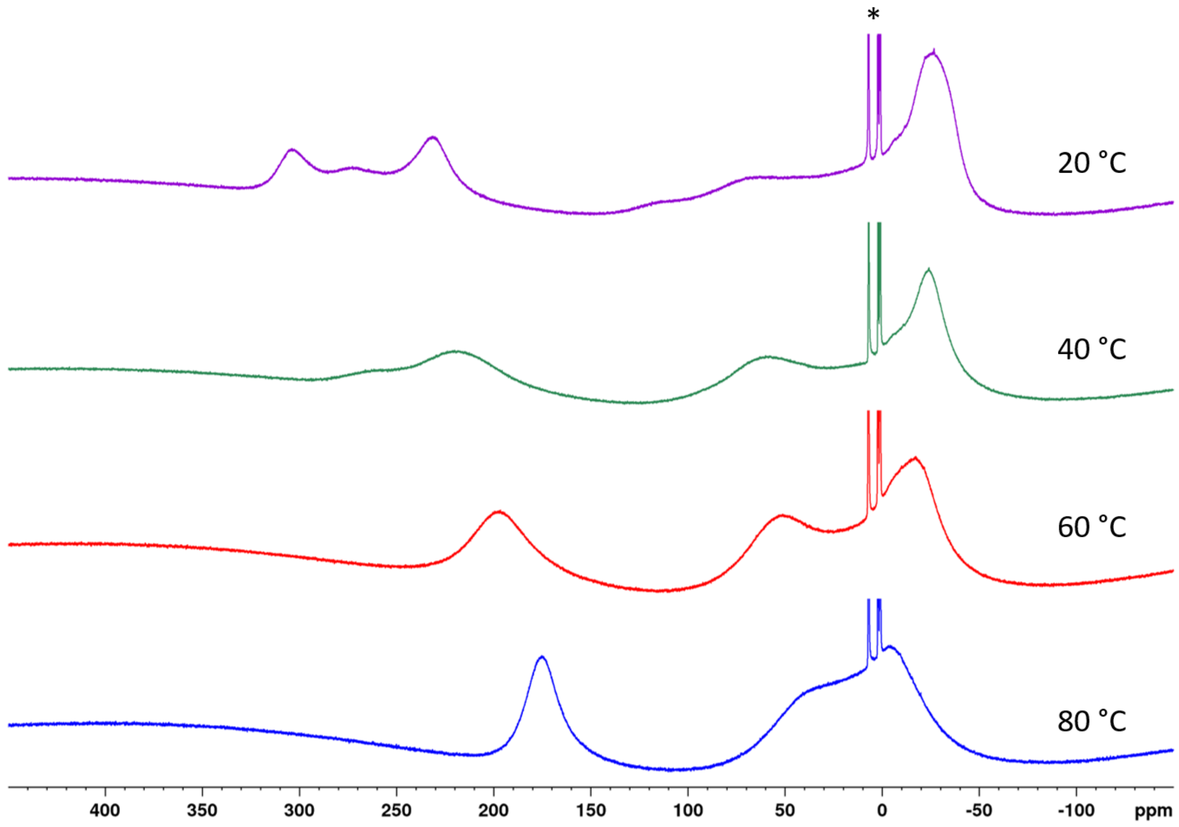

Fig. S12. ^{1}H NMR (300 MHz, tol-d_8) spectra of **5** at variable temperatures (residual protio solvent signals assigned with *).

II.6. NMR spectra of 6

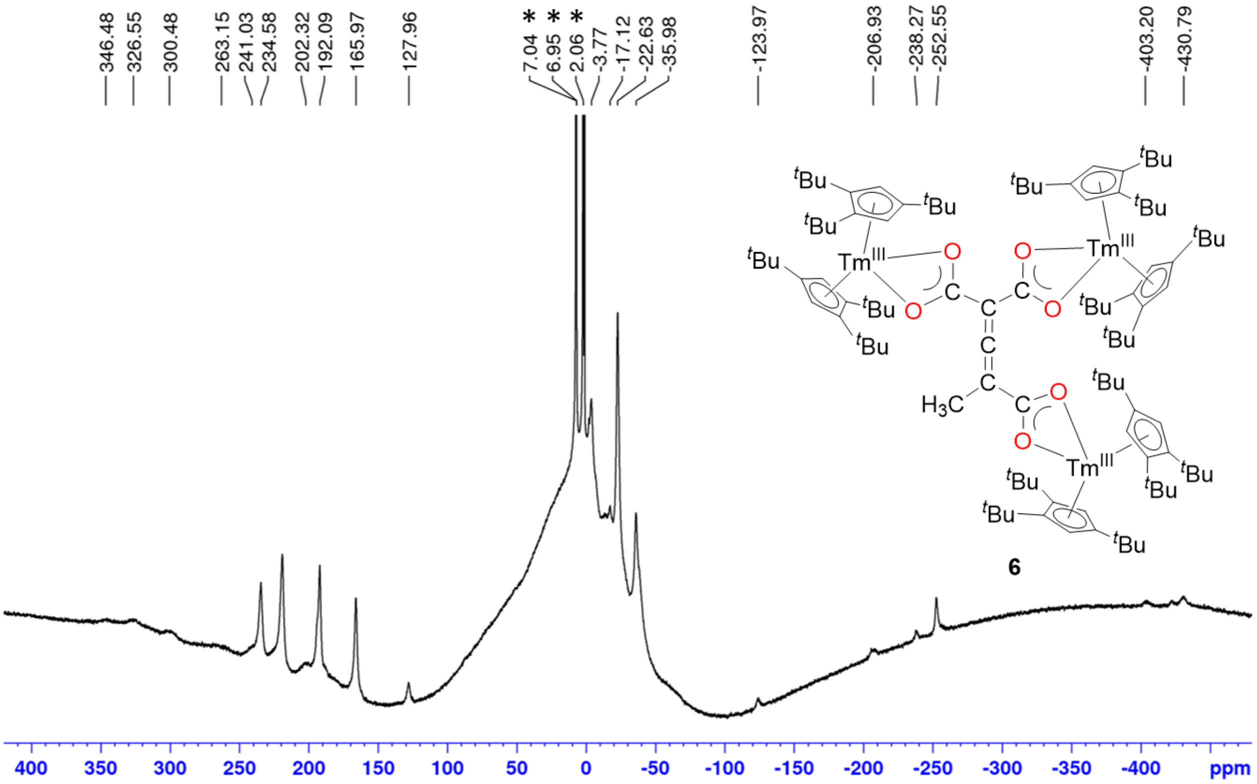

Fig. S13. ^{1}H NMR (300 MHz, tol-d_8, 293 K) spectrum of **6** (solvent signals assigned with *).

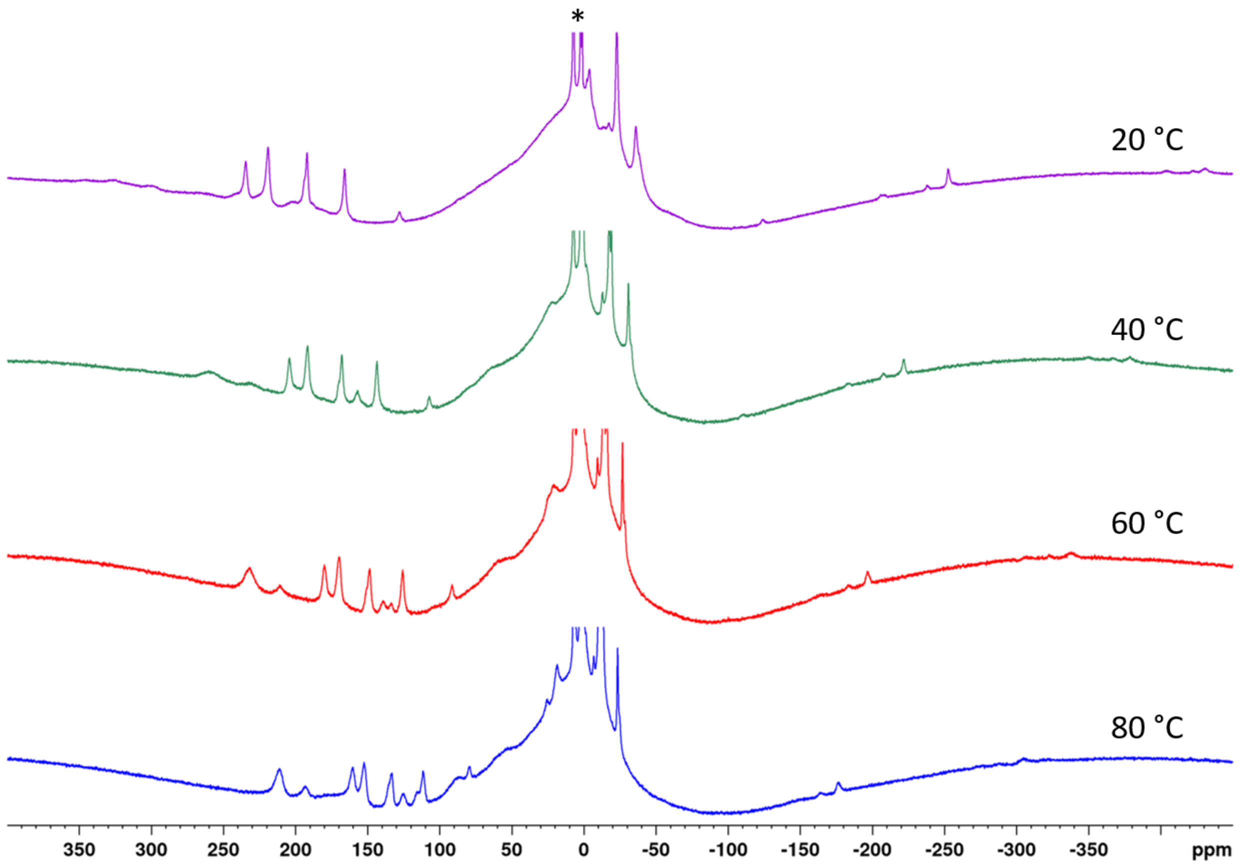

Fig. S14. ^{1}H NMR (300 MHz, tol-d_8) spectra of **6** at variable temperatures (residual protio solvent signals assigned with *).

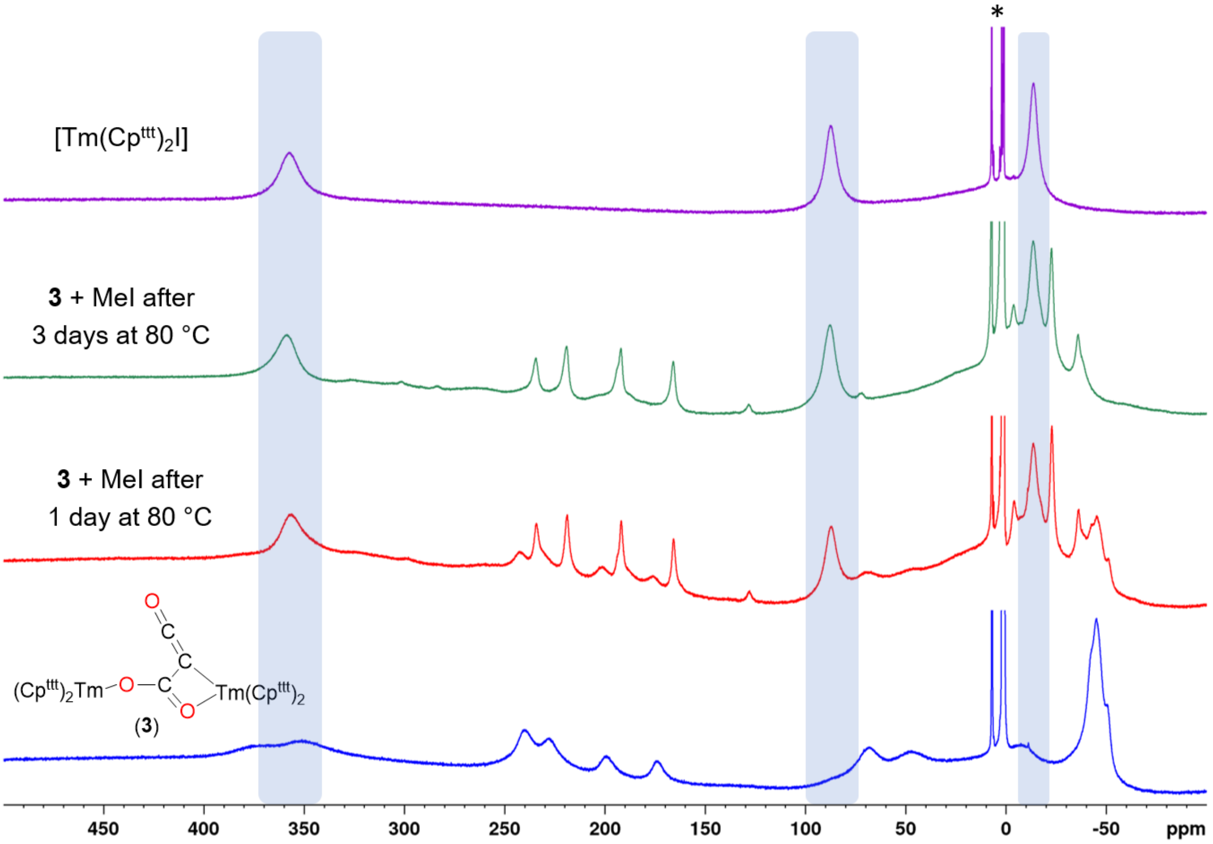

Fig. S15. Evolution of the ^{1}H NMR (300 MHz, tol-d_8) spectra upon reaction of **3** with MeI at 80 °C and comparison with the spectrum of isolated [Tm(Cp^{ttt})$_2$I] (solvent signals assigned with *).

II.7. NMR spectra of 7

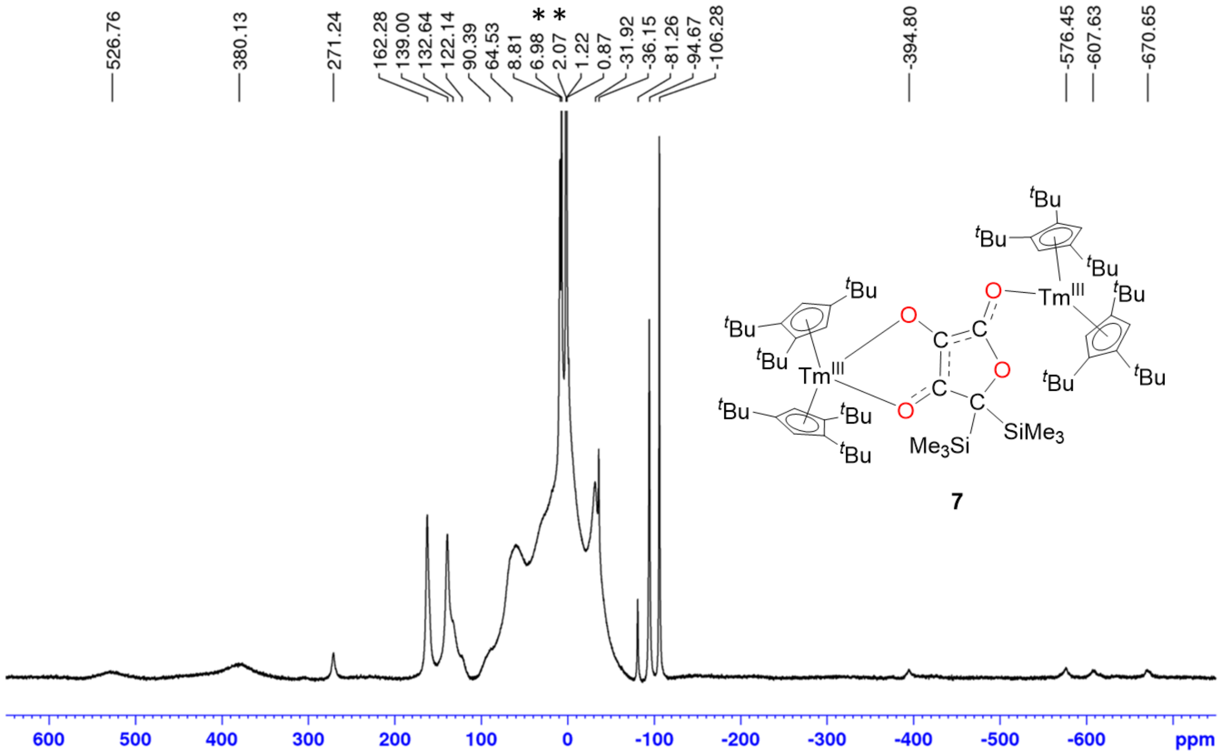

Fig. S16. ^{1}H NMR (300 MHz, tol-d_8, 293 K) spectrum of **7** after spline baseline correction (residual protio solvent signals assigned with *).

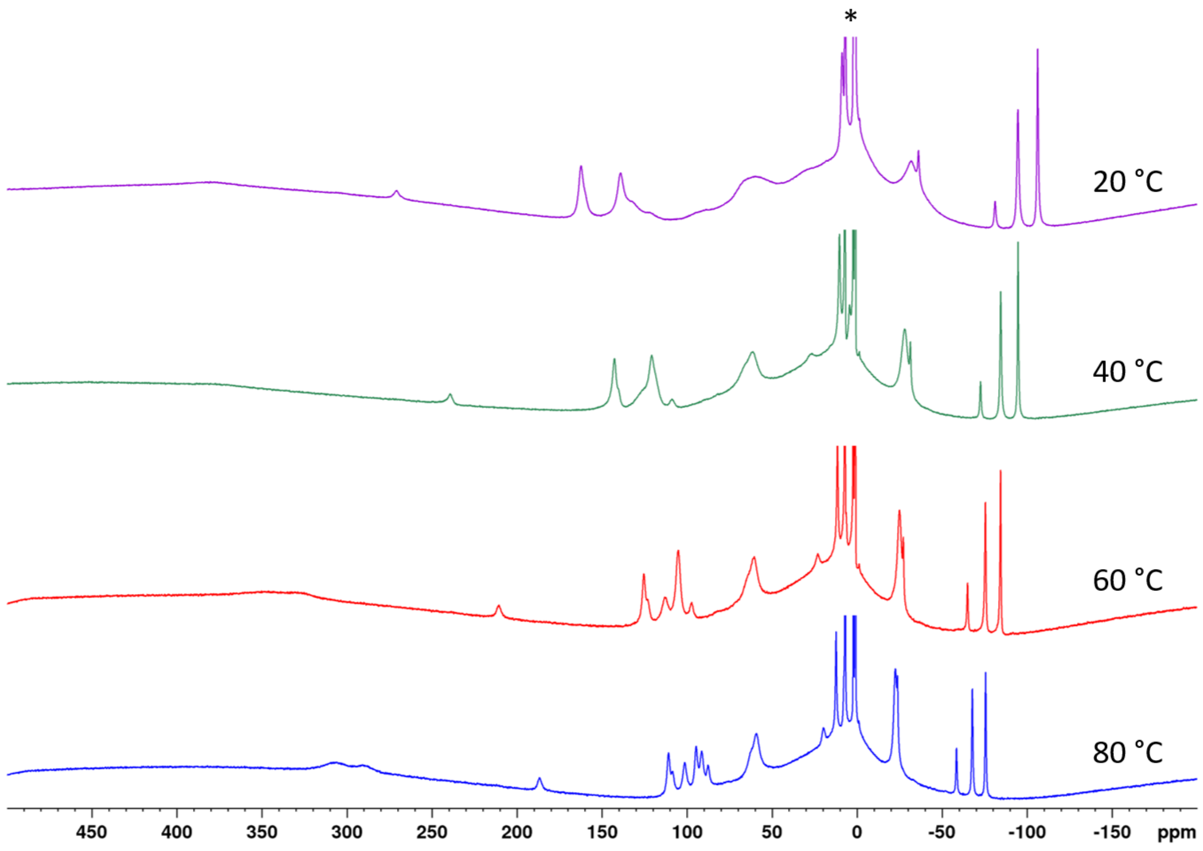

Fig. S17. ^{1}H NMR (300 MHz, tol-d_8) spectra of **7** at variable temperatures (residual protio solvent signals assigned with *) in the range δ -200 to +500 ppm.

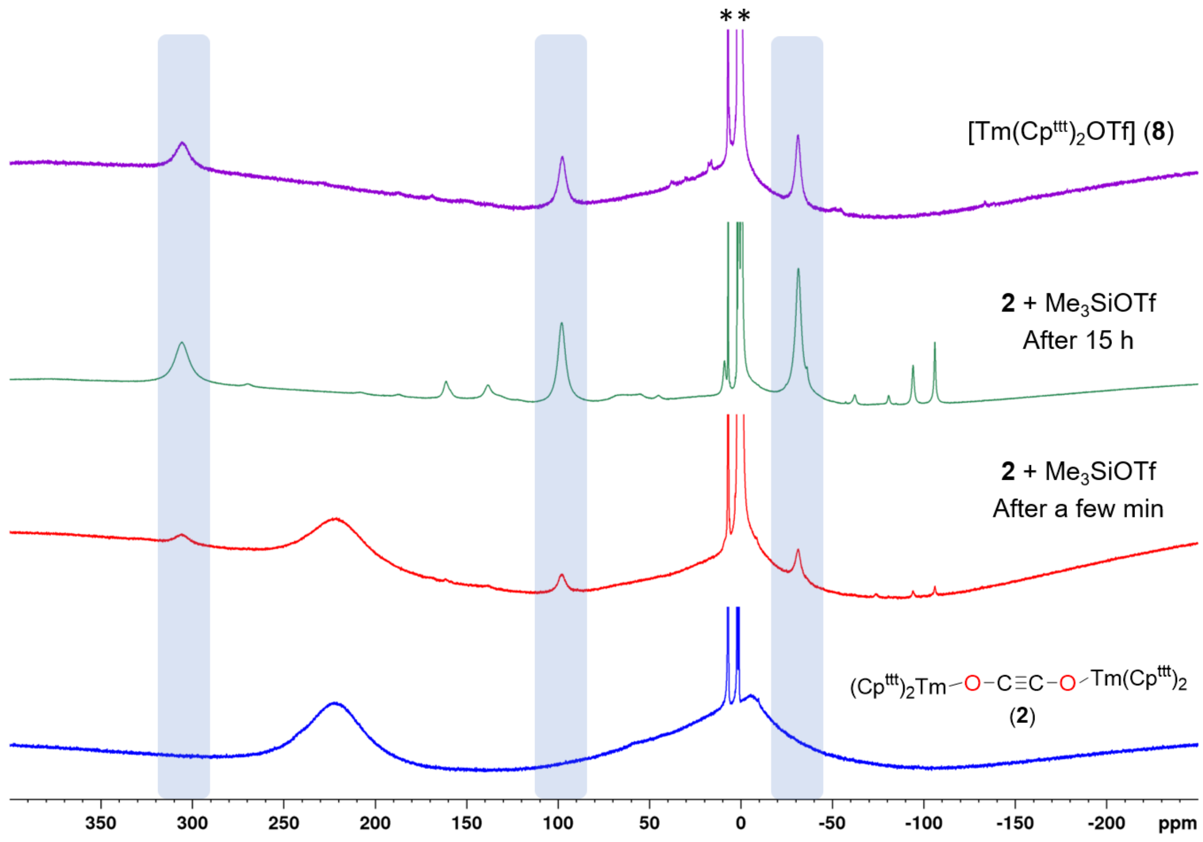

Fig. S18. Evolution of the ^{1}H NMR (300 MHz, tol-d_8) spectra upon reaction of **2** with Me_3SiOTf at room temp. and comparison with the spectrum of isolated [Tm(Cp^{ttt})$_2$OTf] (**8**) (solvent signals assigned with *).

II.8. ^{1}H NMR spectrum of 8

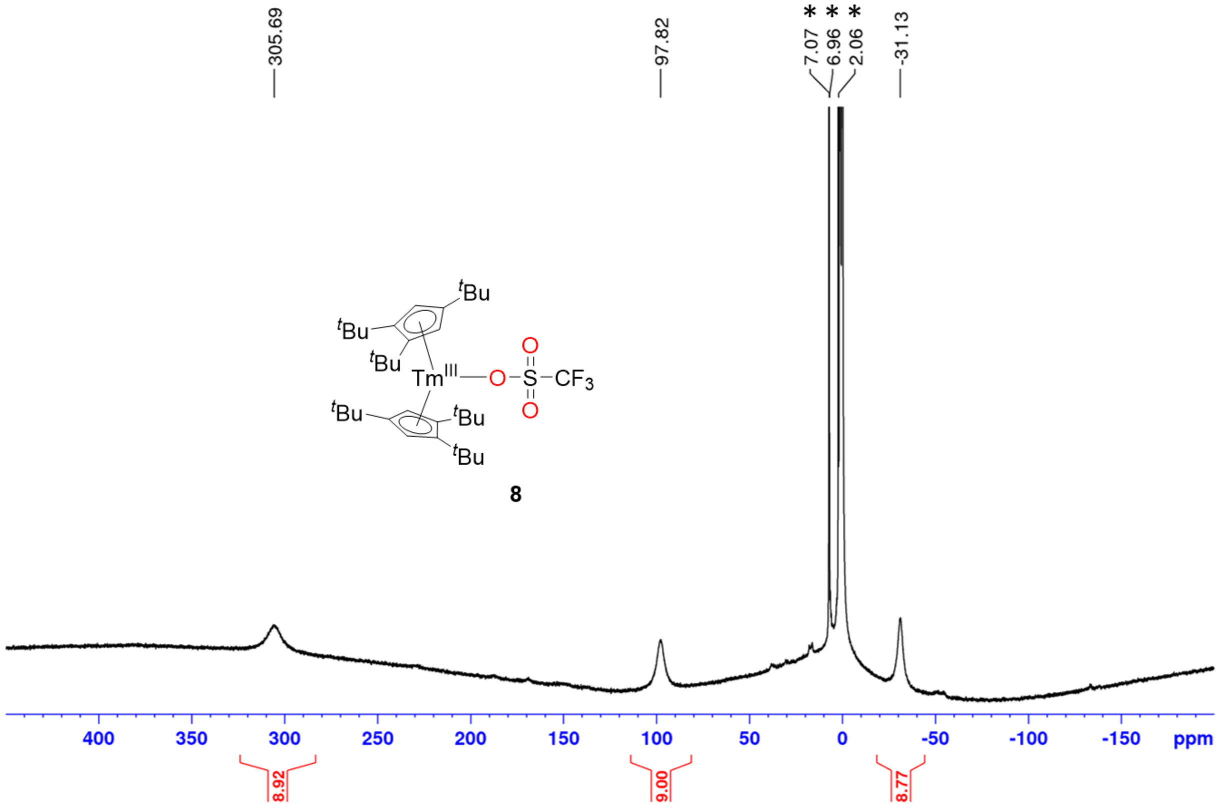

Fig. S19. ^{1}H NMR (300 MHz, tol-d_8, 293 K) spectrum of **8** (solvent signals assigned with *).

II.9. NMR spectra of 9a-b

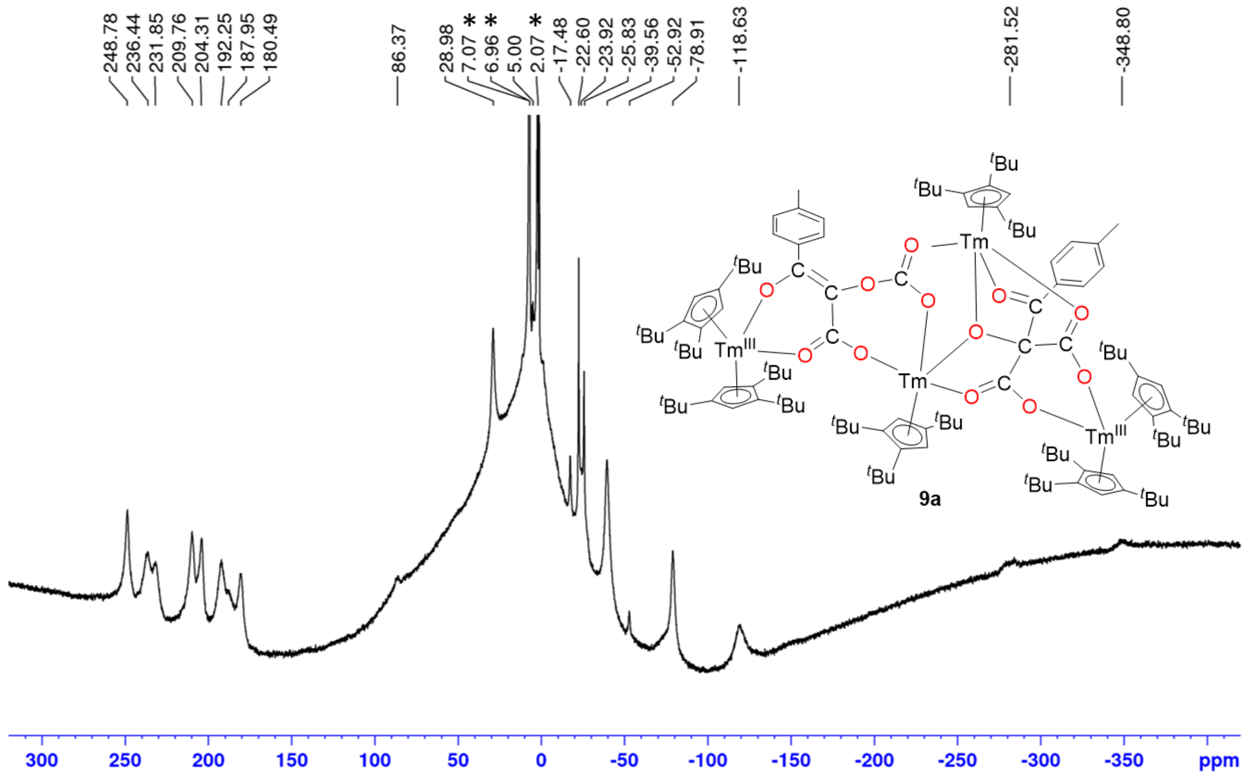

Fig. S20. ^{1}H NMR (300 MHz, tol-d_8, 293 K) spectrum of **9a** (solvent signals assigned with *).

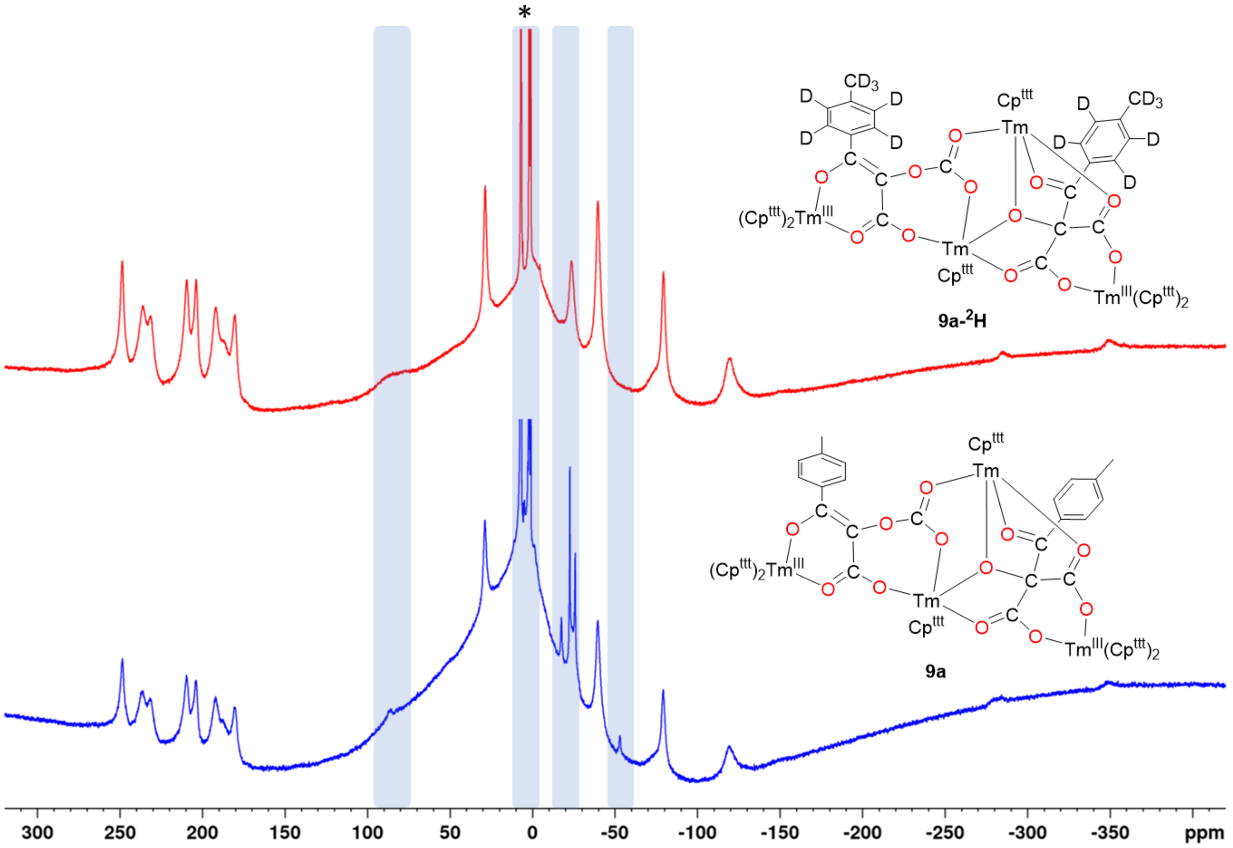

Fig. S21. Comparison of the ^{1}H NMR (300 MHz, tol-d_8) spectra of **9a** and **9a-²H** (residual protio solvent signals assigned with *).

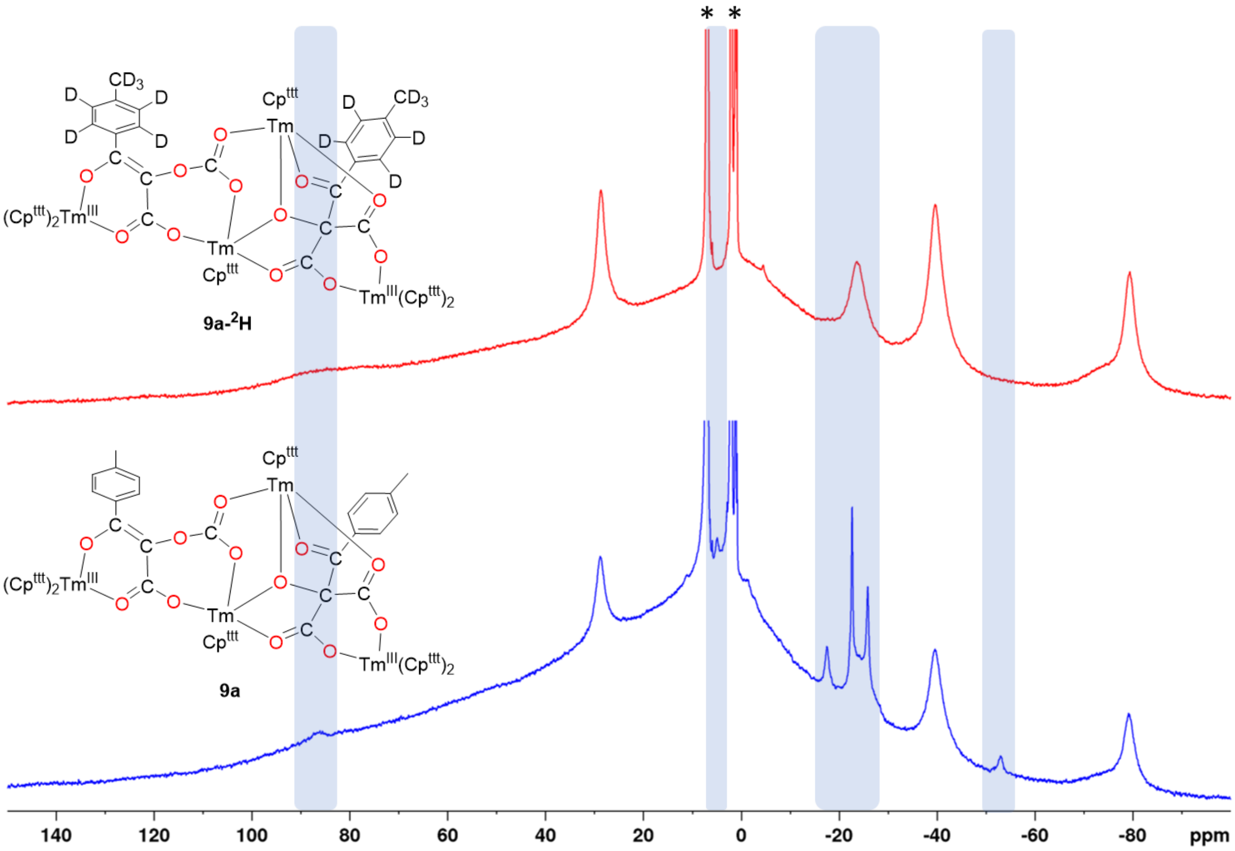

Fig. S22. Detail of the region from δ –100 to +150 ppm in the comparison of the ^{1}H NMR (300 MHz, tol-d_8) spectra of **9a** and **9a-²H** (solvent signals assigned with *).

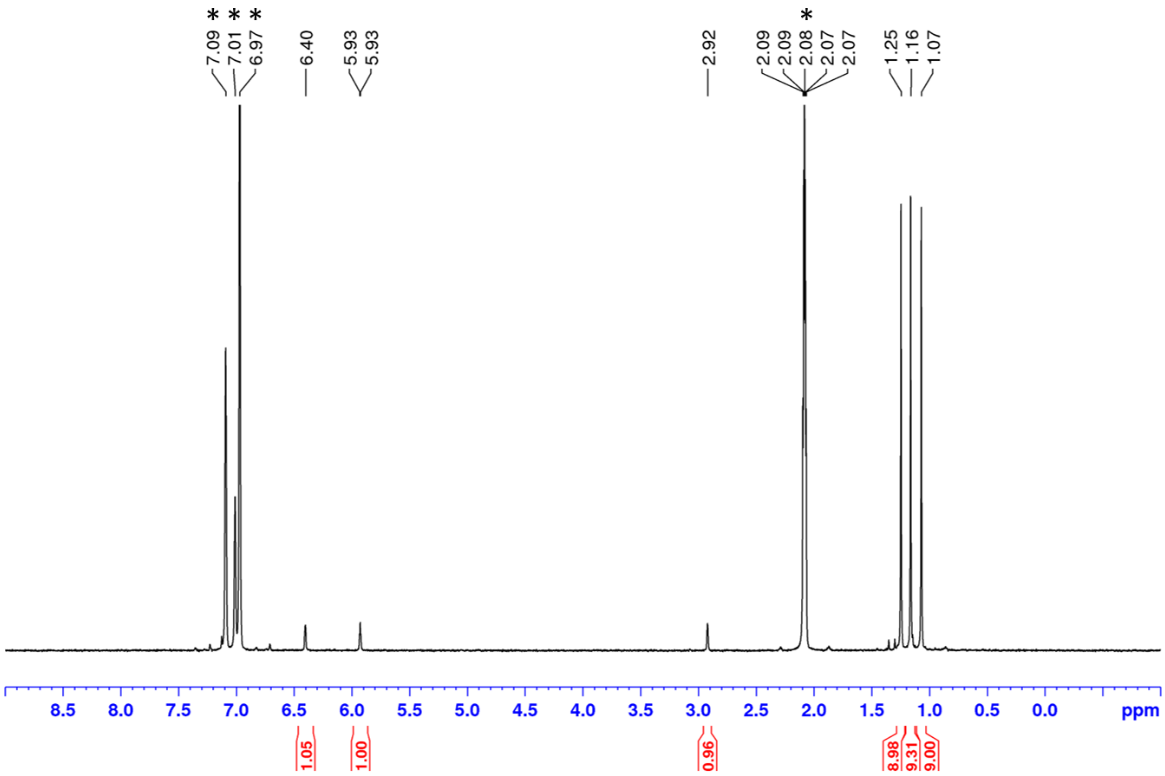

Fig. S23. ^{1}H NMR (300 MHz, tol-d_8, 293 K) spectrum of the oily residue obtained upon transfer of the volatiles from the reaction of **2** with CO_2 in toluene followed by evaporation under vacuum (residual protio solvent signals at δ 7.09, 7.01, 6.97 and 2.08 assigned with *).

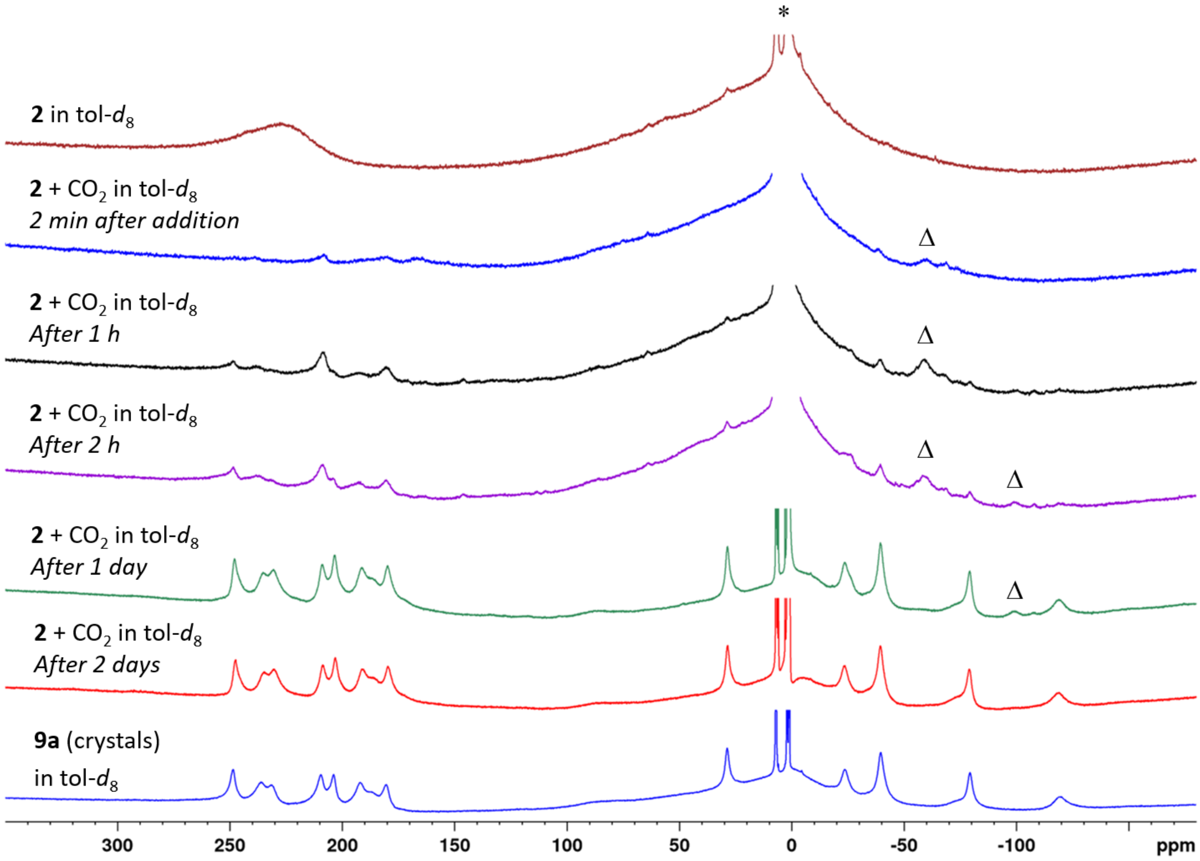

Fig. S24. ^{1}H NMR (300 MHz, tol-d_8, 293 K) monitoring of the reaction of **2** with CO_2 in tol-d_8 (solvent signals assigned with *). The signals for a possible intermediate species have been assigned with Δ.

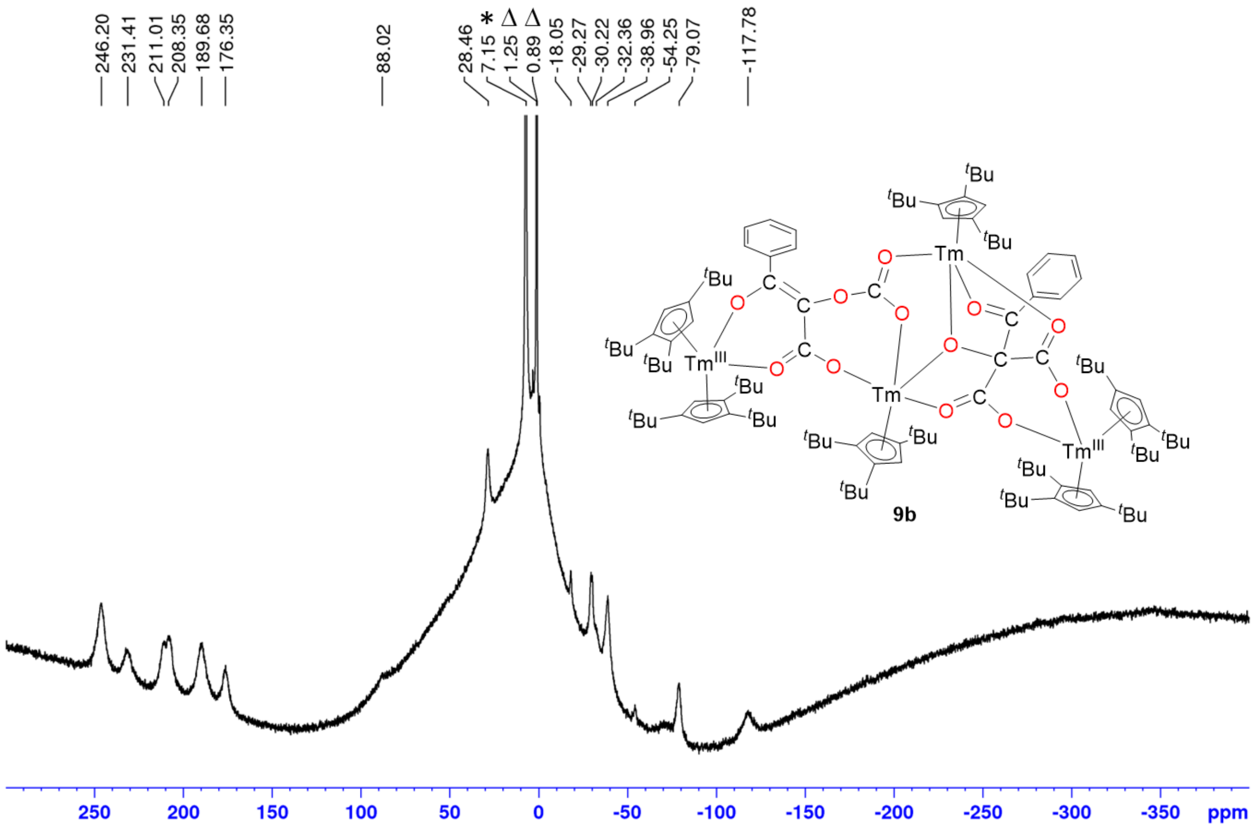

Fig. S25. ^{1}H NMR (300 MHz, C_6D_6, 293 K) spectrum of **9b** (solvent signal assigned with *). Traces of pentane (Δ) can be noticed at δ 1.25 and 0.89, originating from the crystals.

III. IR spectra

III.1. IR spectra of complexes 2–9

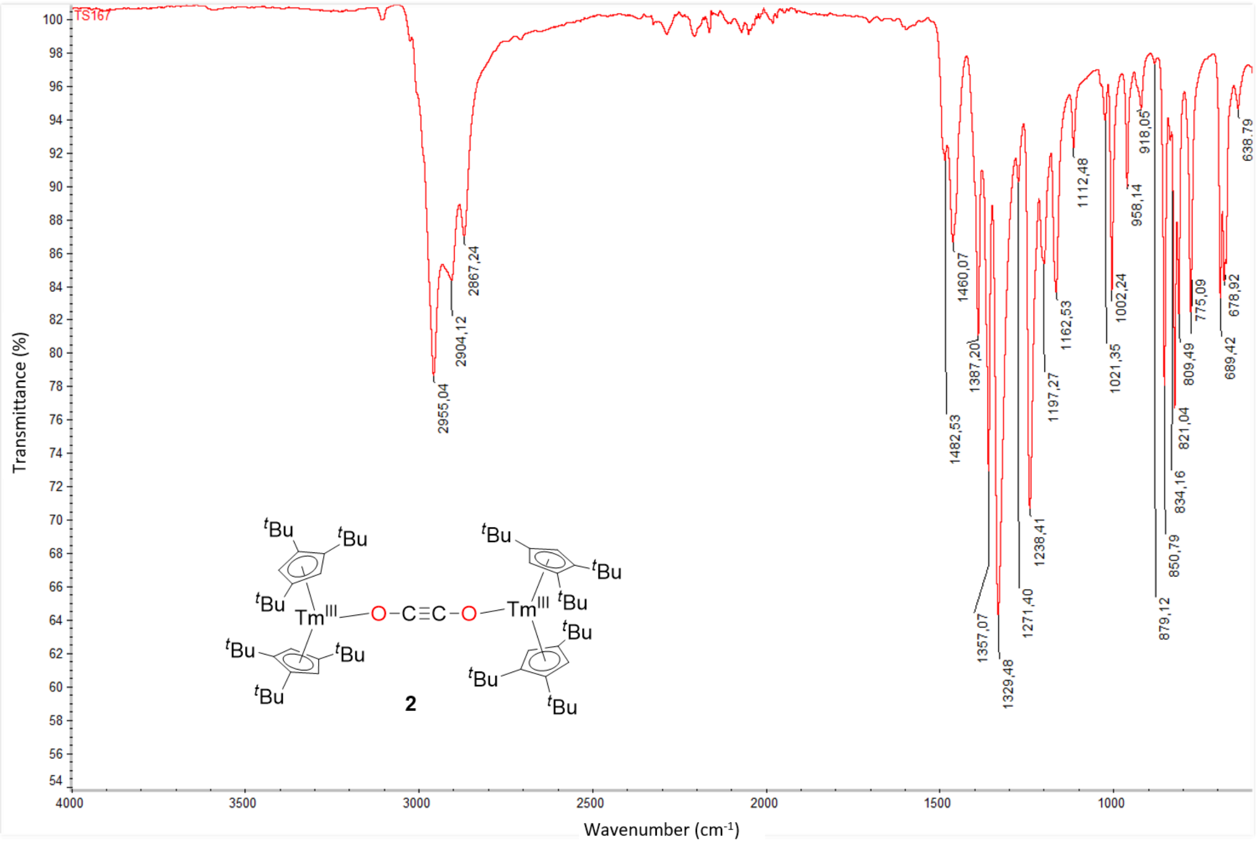

Fig. S26. IR spectrum of **2**.

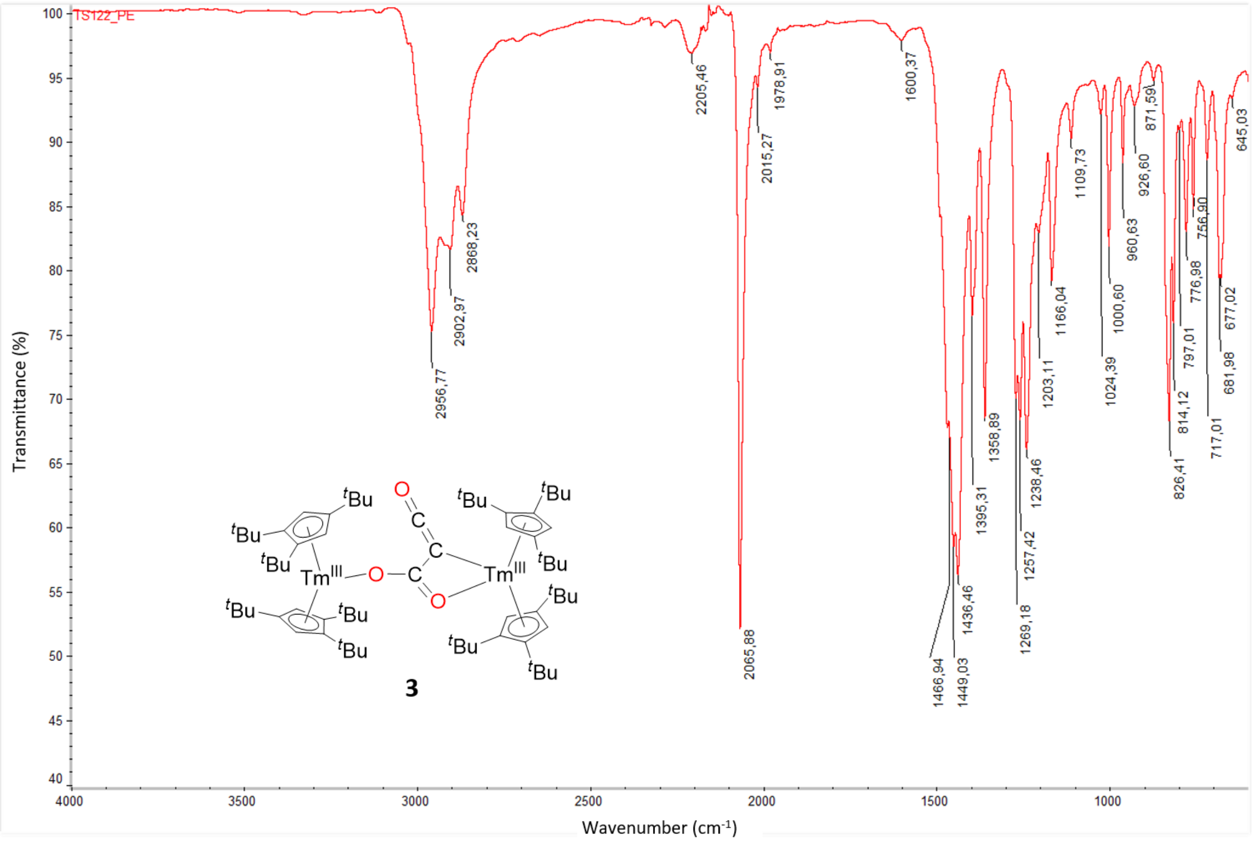

Fig. S27. IR spectrum of **3**.

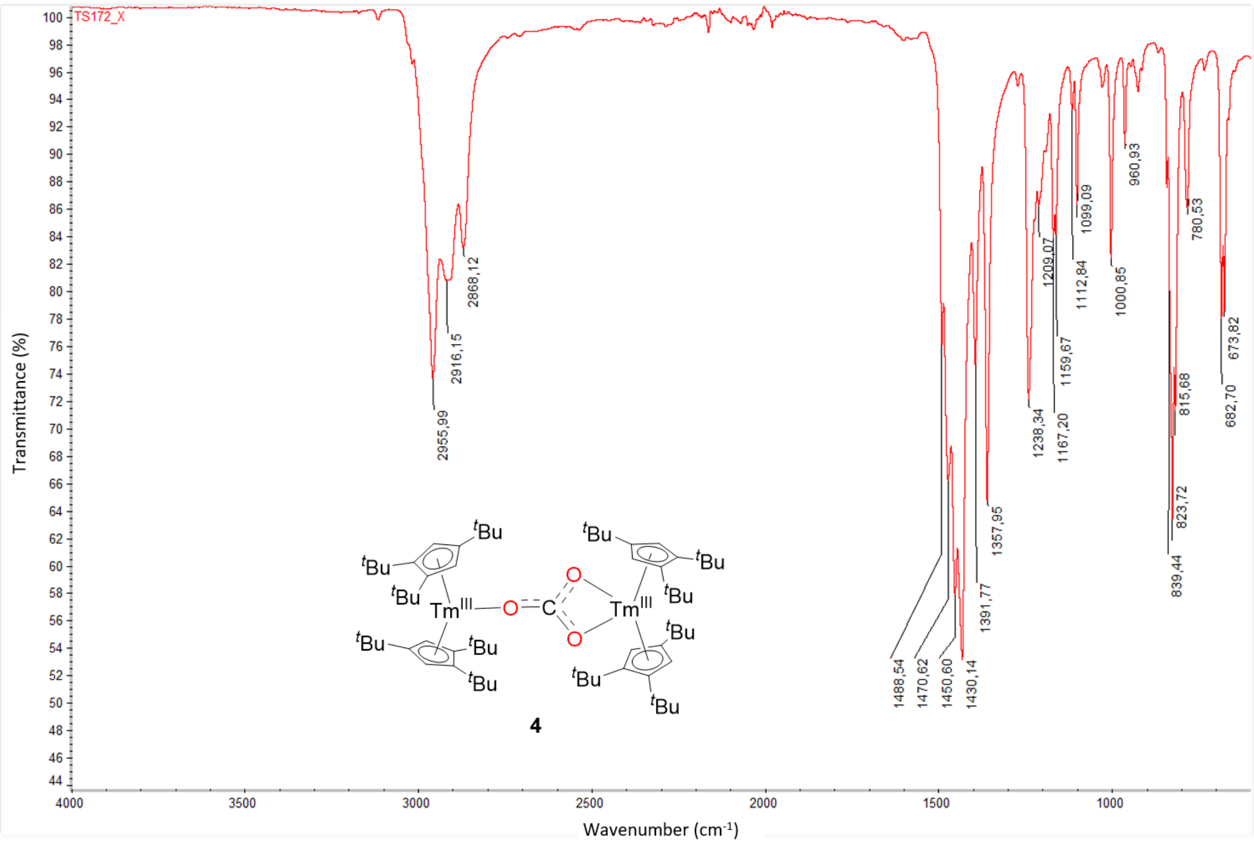

Fig. S28. IR spectrum of **4**.

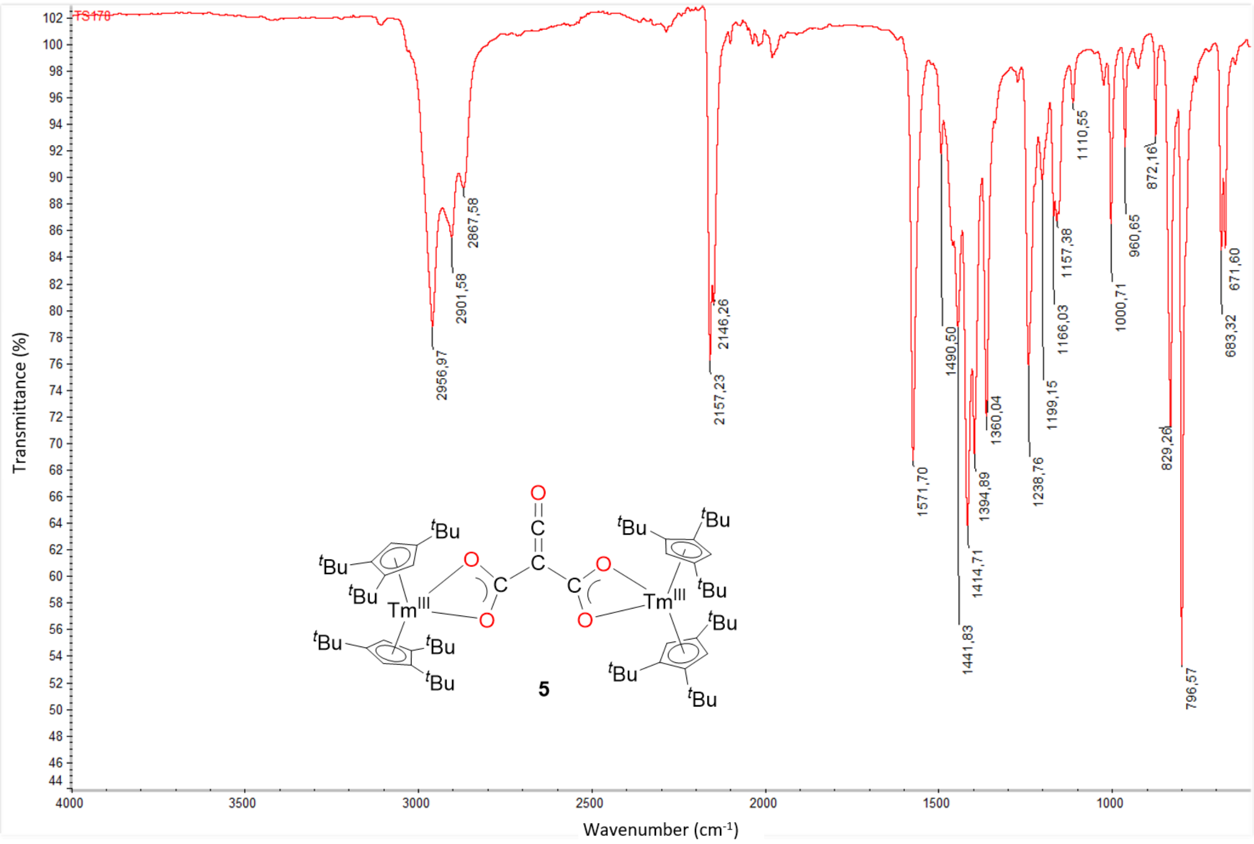

Fig. S29. IR spectrum of **5**.

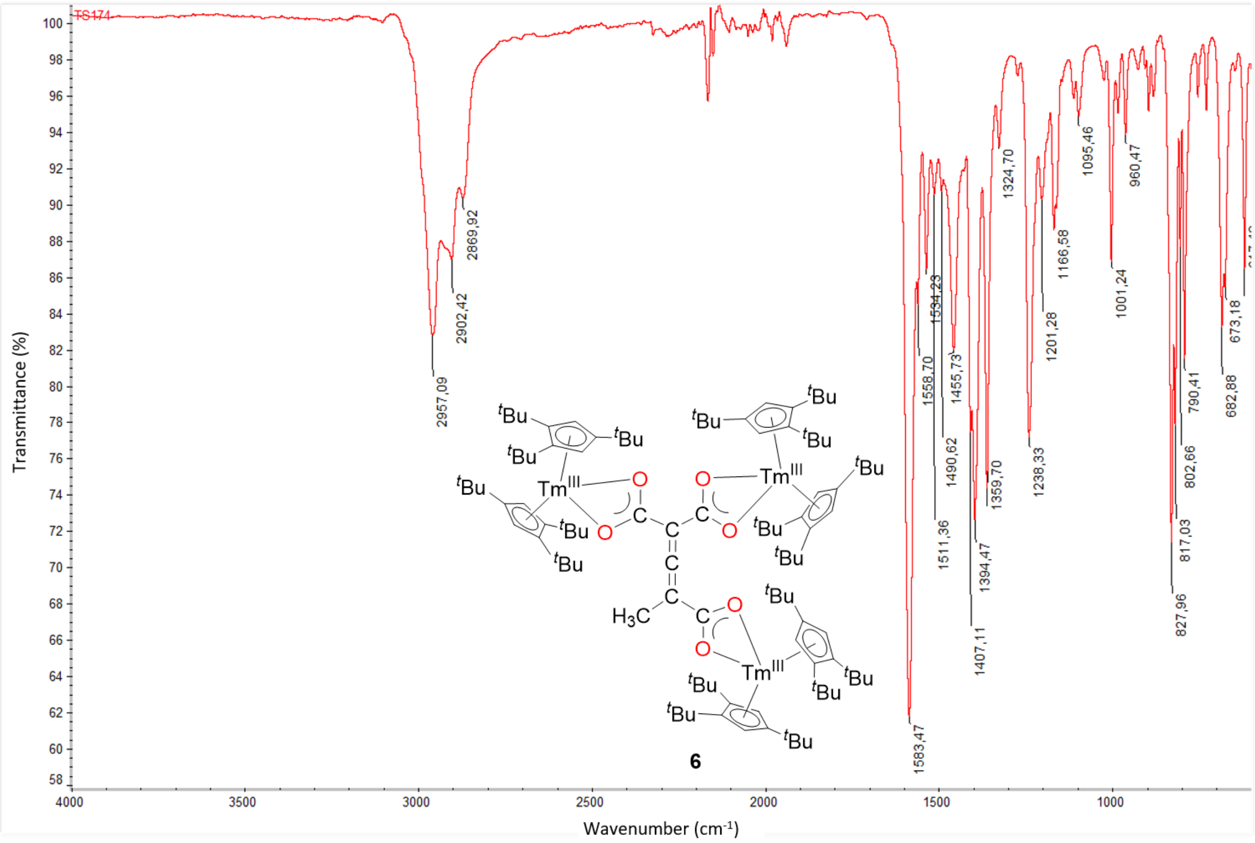

Fig. S30. IR spectrum of **6**.

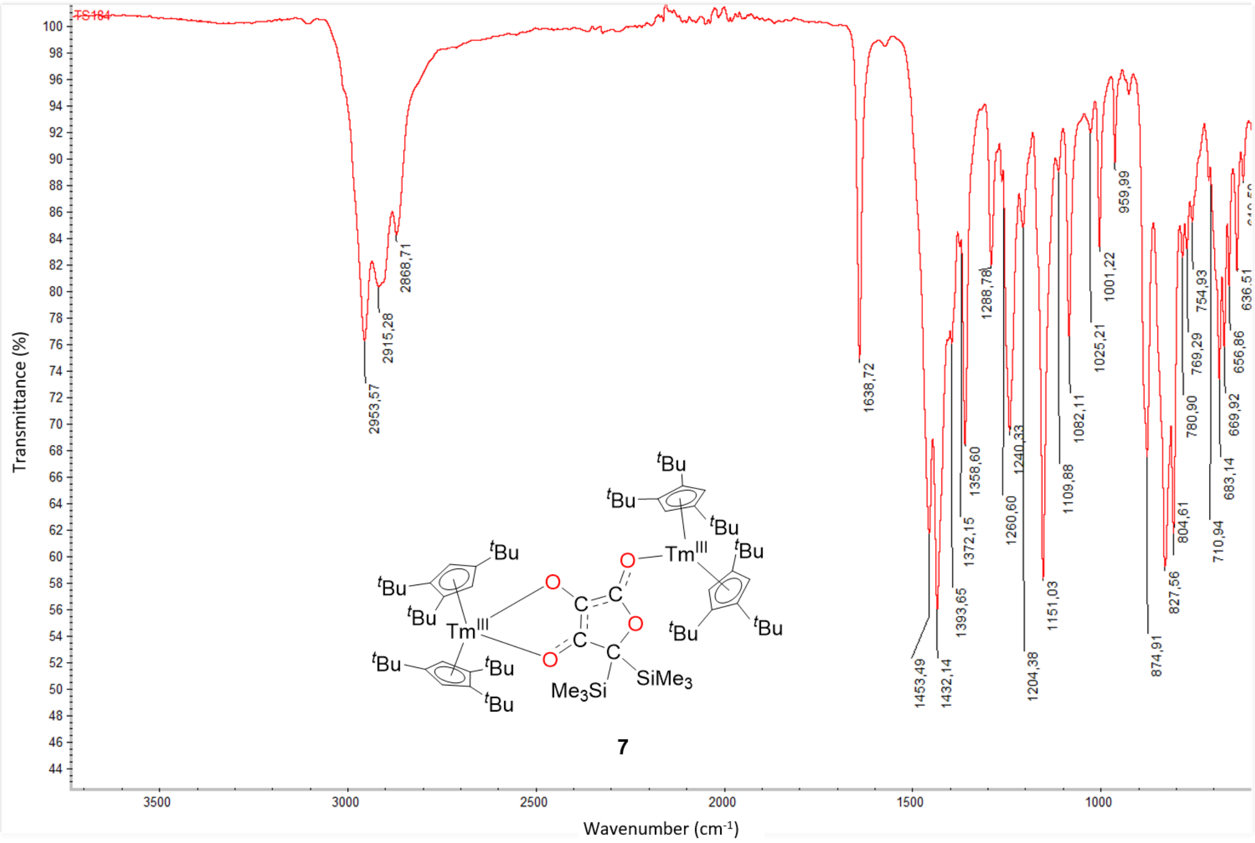

Fig. S31. IR spectrum of **7**.

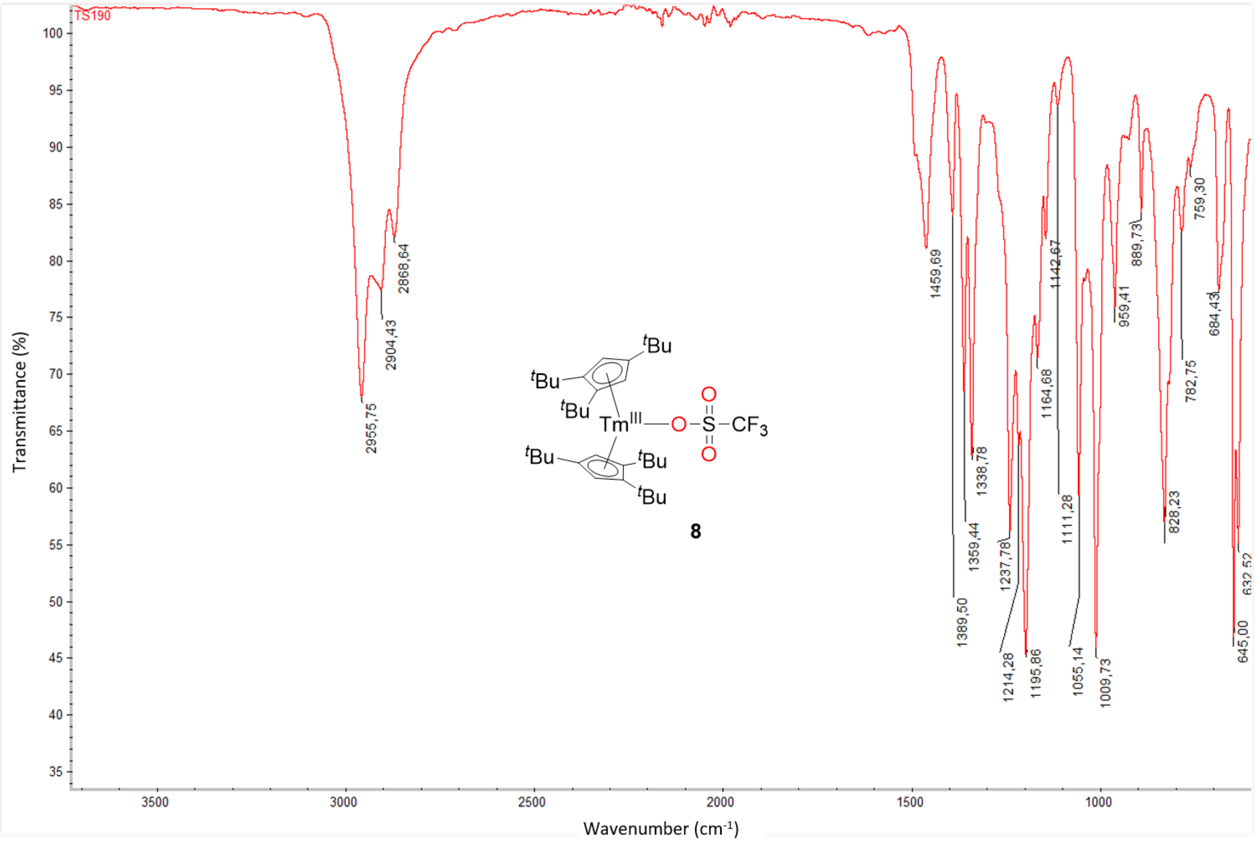

Fig. S32. IR spectrum of **8**.

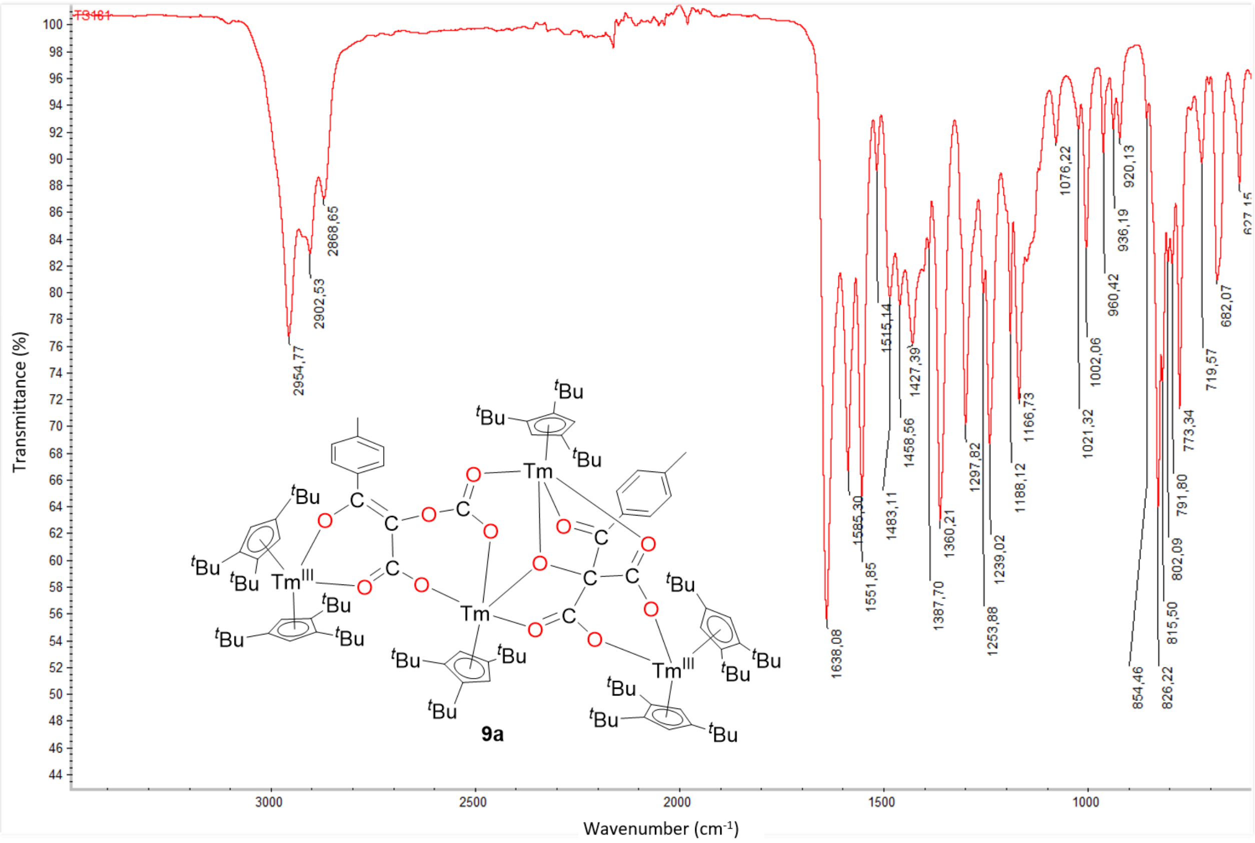

Fig. S33. IR spectrum of **9a**.

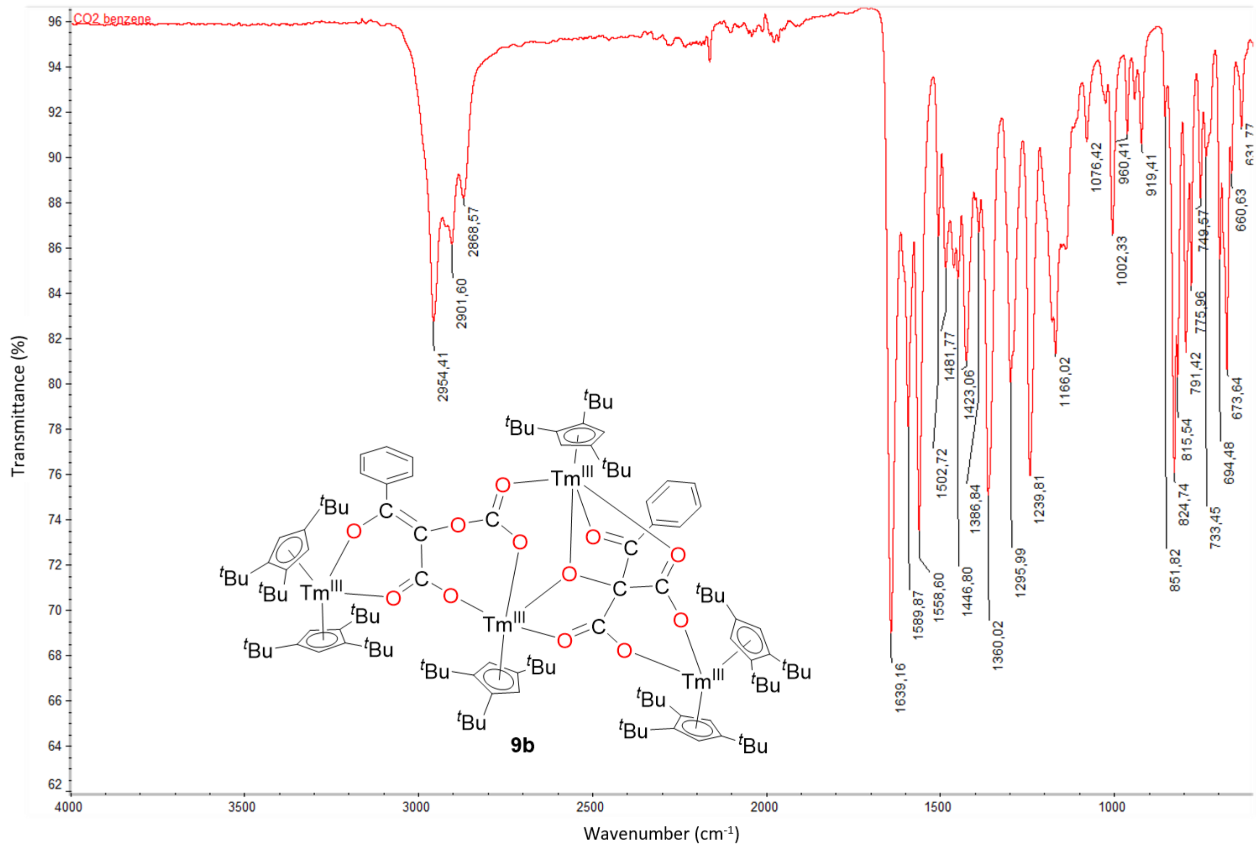

Fig. S34. IR spectrum of **9b**.

III.2. IR spectra of 2-^{13}C and 3-^{13}C

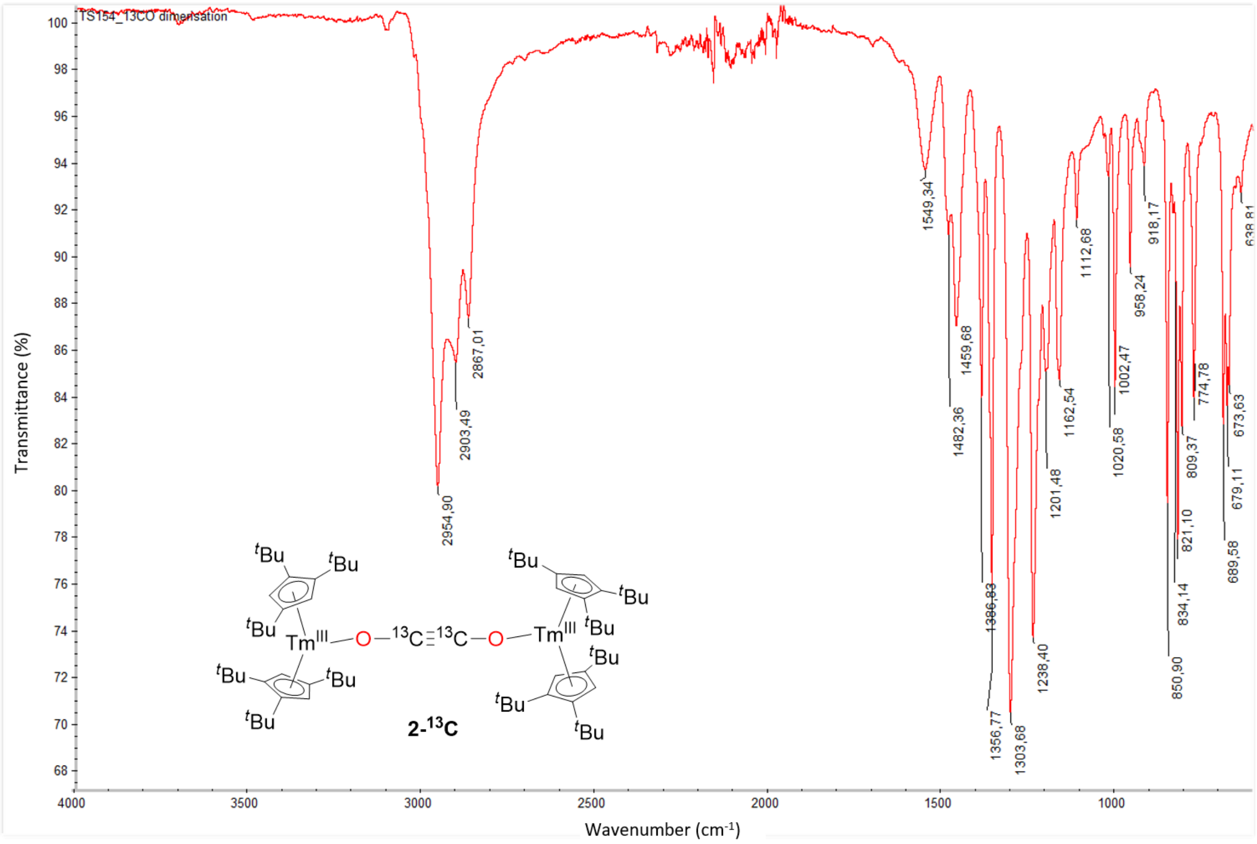

Fig. S35. IR spectrum of **2-^{13}C**.

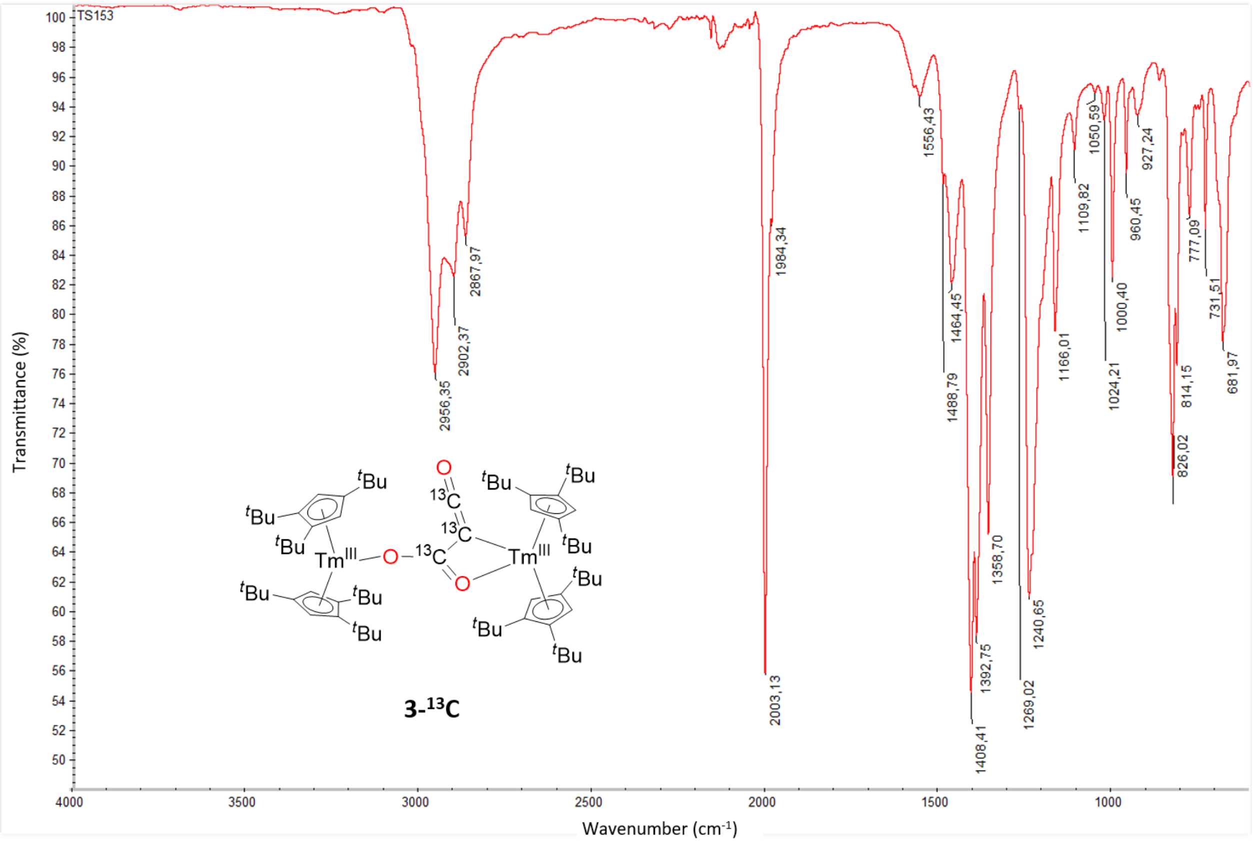

Fig. S36. IR spectrum of **3-^{13}C**.

III.3. Comparison of IR spectra with and without ^{13}CO labelling

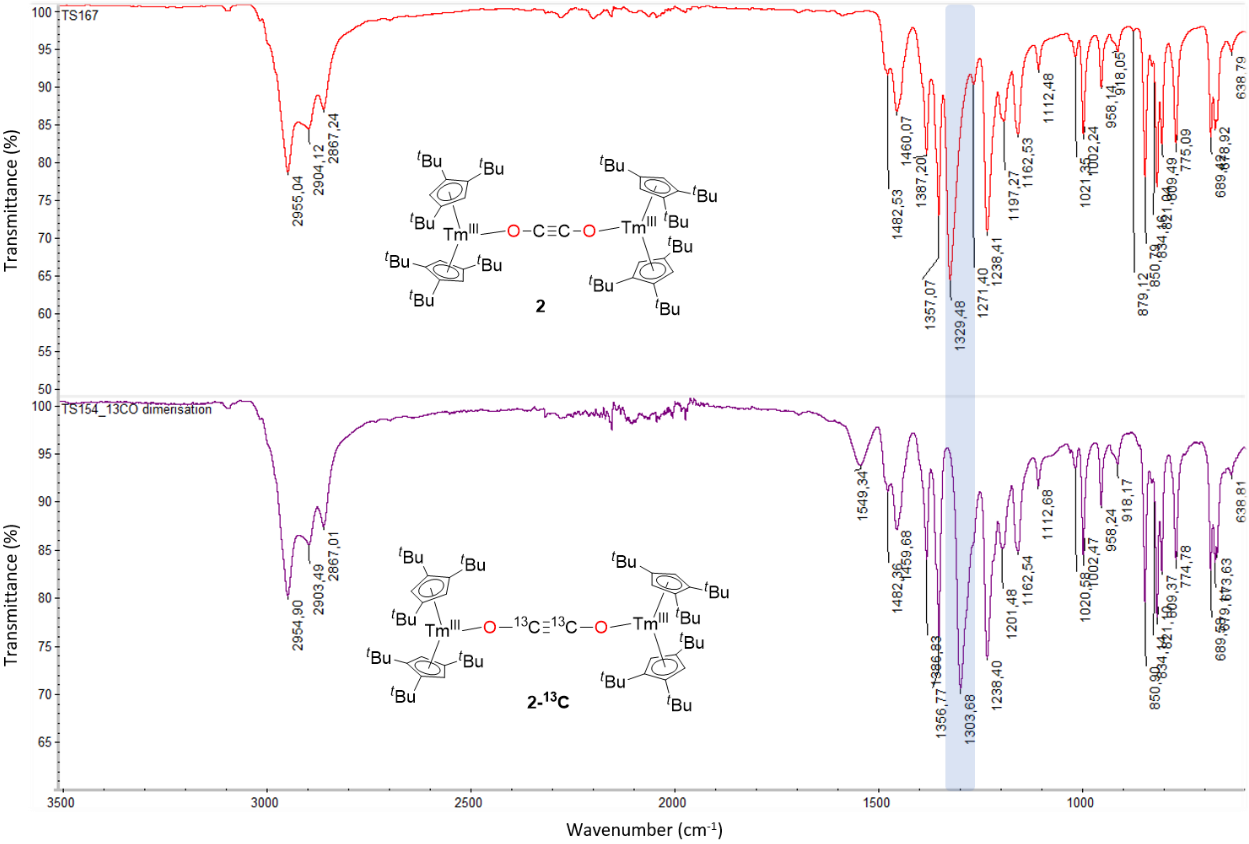

Fig. S37. Comparison of the IR spectra of **2** and **2-^{13}C**.

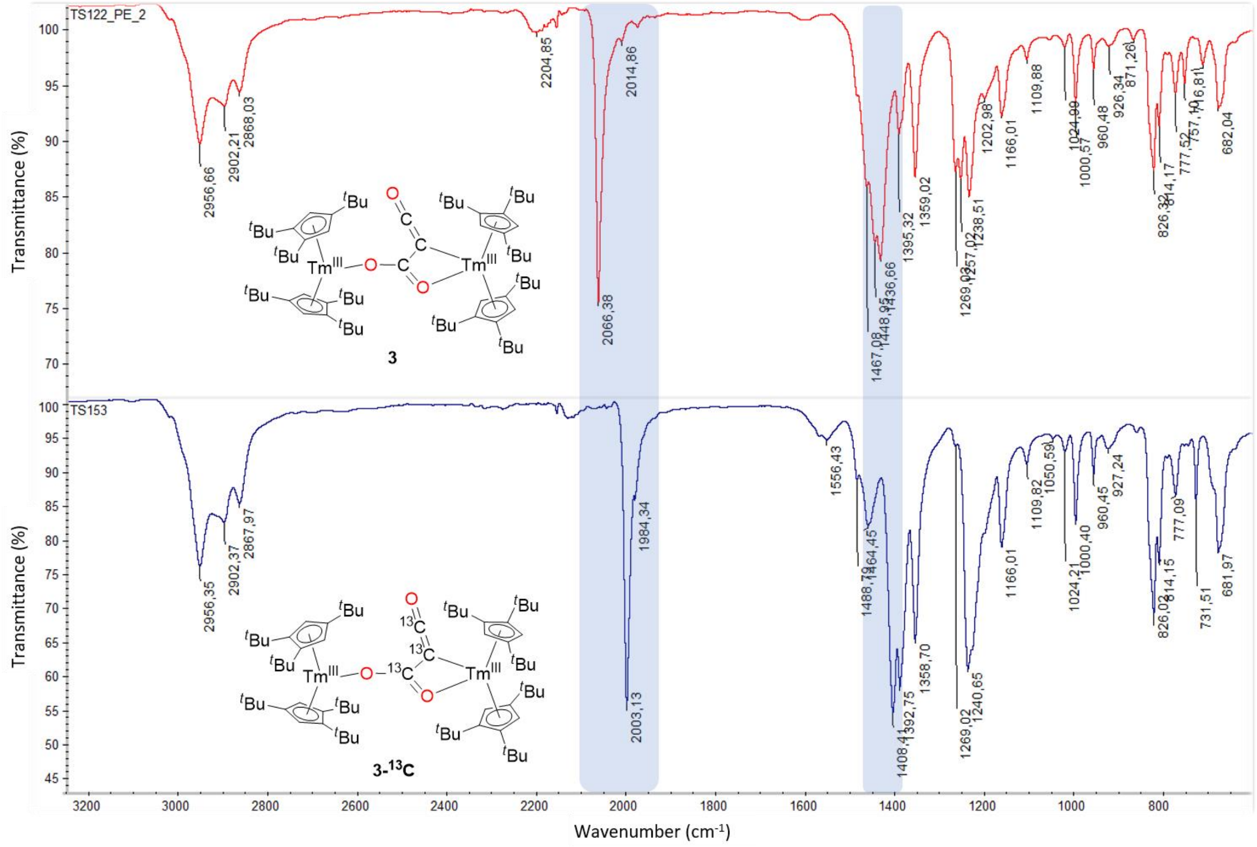

Fig. S38. Comparison of the IR spectra of **3** and **3-^{13}C** (full spectrum).

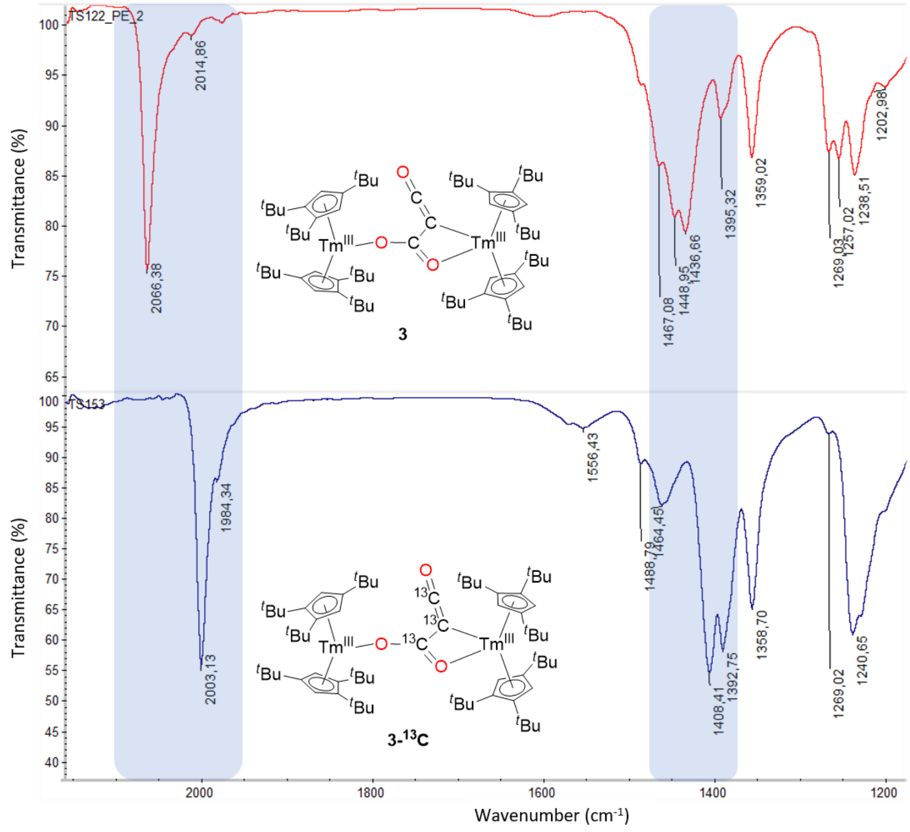

Fig. S39. Comparison of the IR spectra of **3** and **3-^{13}C** (detail of the region 2200–1200 cm^{-1}).

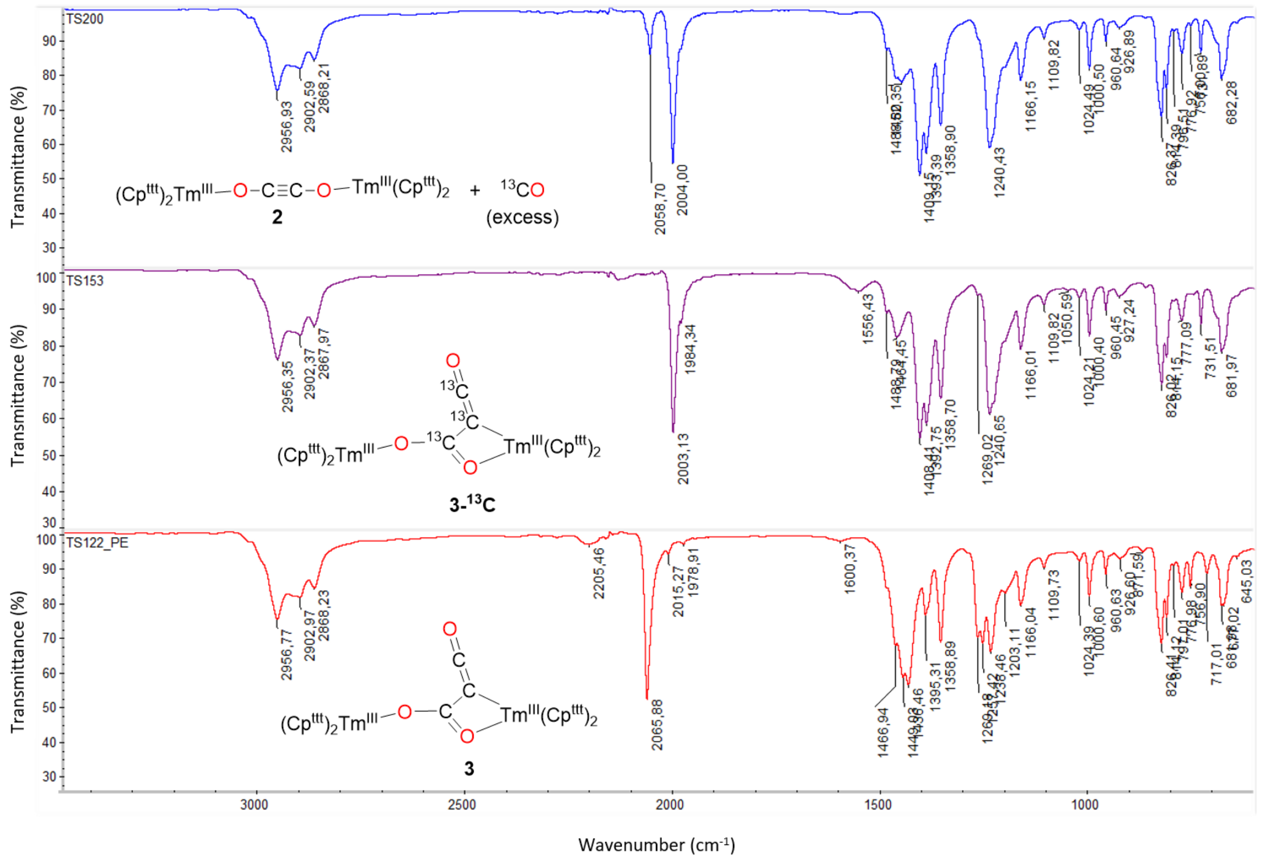

Fig. S40. IR spectrum of the product from the reaction of **2** with excess ^{13}CO (top) and comparison with the IR spectra of isolated **3-^{13}C** (middle) and **3** (bottom).

The IR spectrum of the product form the reaction between **2** and excess ^{13}CO, displayed in Fig. S40, reveals the formation of **3-^{13}C** as a major species.

To account for the formation of the fully ^{13}C-labelled isotopologue **3-^{13}C** from the reaction of **2** with excess ^{13}CO, we suggest the mechanism depicted in Scheme S1: the coordination of ^{13}CO to **2** leads to the adduct **A1** and, after the **TS2** transition state associated with an activation barrier of *ca.* 17.0 kcal.mol^{-1}, to the intermediate product **B1**. In this intermediate, the coordination modes of the two Tm centers can be easily exchanged leading to an equilibrium with the isotopomer **B2**. From **B2**, the retro-insertion of ^{12}CO leads to **A2**, with an associated activation barrier of similar energy (*ca*. 12.7 kcal.mol^{-1}). This reverse activation barrier is notably of lower magnitude than that of **TS3** (*ca*. 37.7 kcal.mol^{-1}) meaning that, in the presence of excess ^{13}CO, the scrambling of ^{13}C labels will occur faster than the formation of **3*** featuring mono-insertion of ^{13}CO. The intermediate **A2** is then in equilibrium with its isotopologue **A3**, and by further applying the same reasoning, accumulation of the **A5**/**B5** intermediate species occurs in the presence of excess ^{13}CO. Finally, through a rate-determining step, **3-^{13}C** is obtained as the major product.

Thus, the formation of the fully ^{13}C-labelled isotopologue **3-^{13}C** from the reaction of **2** with excess ^{13}CO is consistent with the energetic profile depicted in Fig. 5.

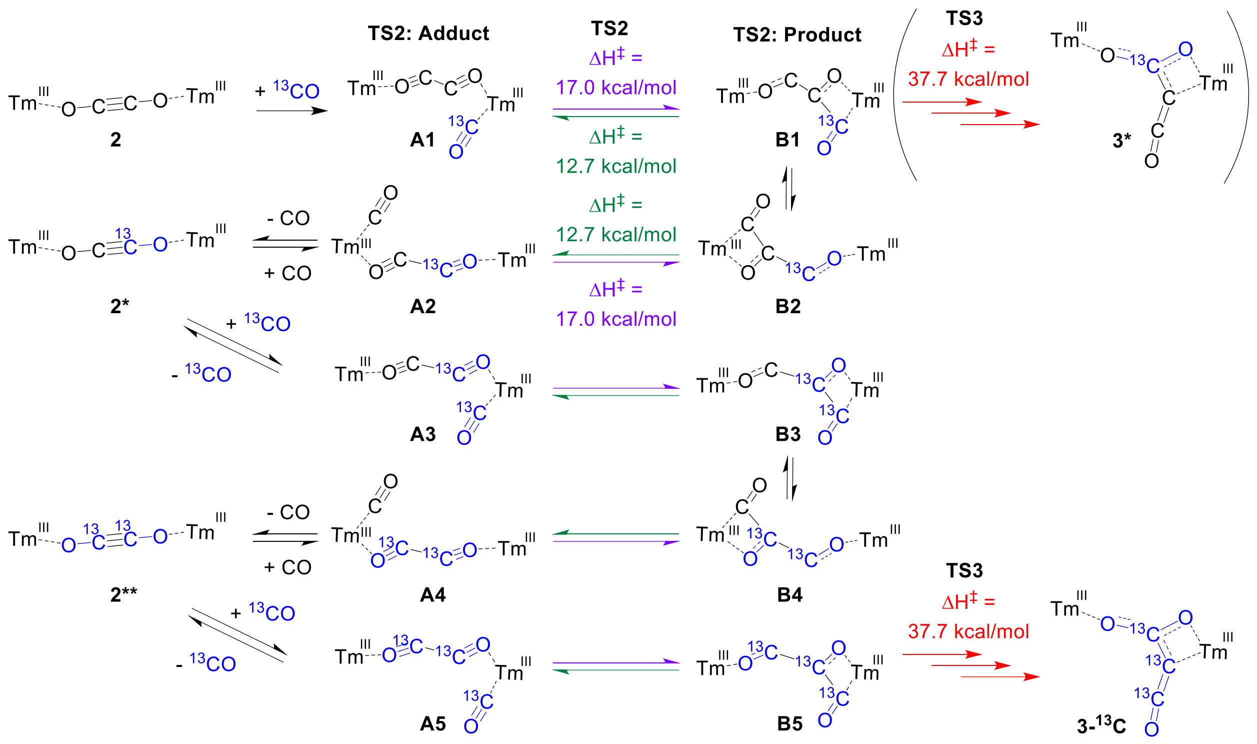

Scheme S1. Possible mechanism for the scrambling of ^{13}C labels upon reaction of **2** with excess ^{13}CO.

IV. X-ray crystallography

IV.1. General methods

Single crystals of the complexes suitable for X-ray analysis were mounted on a Kapton loop using a Paratone N oil on a Nonius diffractometer equipped with an APEX II CCD BRUKER detector and a graphite Mo-Kα monochromator were used for the data acquisition. All measurements were done at 150 K and a refinement method was used for solving the structure. The resolution of the solid-state structure was accomplished using the SHELXS-97 or SHELXT programs.[5] The refinement was performed with the SHELXL program[6] using the Olex2 software.[7] All atoms – except hydrogens – were refined anisotropically. The position of the hydrogen atoms was determined using residual electronic densities, which are calculated by a Fourier difference. A final weighting step was performed, followed by multiples loops of refinement.

Specific comments for each data set are given below. Summary of the crystal data, data collection and refinement for the different complexes are given in Tables S1–S3. Crystallographic data for the structures reported in this paper have been deposited with the Cambridge Crystallographic Data Centre as a supplementary publication no. CCDC 2118417–2118424, 2128950.

The following special comments apply to the models of the structures:

- The asymmetric unit of **1** contains two independent molecules of the complex. On each molecule, one ^{t}Bu group is disordered over two positions (C32–C34 and C66–C68) with refined occupancy ratios of 0.786/0.214 and 0.788/0.212.
- The crystals of **2** were found to be systematically twinned and an HKLF5 file was generated using the program Platon.[8] The compound was refined as a 2-component twin with BASF = 0.1281(9). Rigid body (RIGU 0.001 0.001) restraints have been added on two ^{t}Bu groups (C5, C12–C15 and C20, C25–C28).
- The asymmetric unit of **4**·(C_5H_{12}) contains half a molecule of the complex and half a molecule of pentane. The pentane fragment could be successfully modeled using similarity restraints on the corresponding bond distances (SADI 0.01) and displacement parameters (SIMU 0.01 0.02). Additionally, rigid body (RIGU 0.001 0.001) restraints have been added on the corresponding atoms. The carbonate ligand (C1, O1, O2, O3) is disordered over two positions with occupancy ratios of 0.5/0.5.
- Complex **5** crystallizes with 0.45 molecule of pentane disordered over multiple positions in the asymmetric unit. It was removed from the electron density map using the Olex2 solvent mask command: 35 electrons were found in a volume of 324 Å^3 in 3 voids per unit cell. This is consistent with the presence of 0.45 [C_5H_{12}] per asymmetric unit which account for 38 electrons per unit cell.

- Complex **7** crystallizes with three molecules of pentane disordered over multiple positions in the asymmetric unit. They were removed from the electron density map using the Olex2 solvent mask command: 263 electrons were found in a volume of 665 A^3 in 2 voids per unit cell. This is consistent with the presence of 3 [C_5H_{12}] per asymmetric unit which account for 252 electrons per unit cell.
- Complex **9a** crystallizes with seven molecules of pentane in the asymmetric unit. Only one pentane molecule could be satisfactorily modeled, the six others being disordered over multiple positions. They were removed from the electron density map using the Olex2 solvent mask command: 538 electrons were found in a volume of 1207 Å^3 in 1 void per unit cell. This is consistent with the presence of 6 [C_5H_{12}] per asymmetric unit which account for 504 electrons per unit cell.
- Complex **9b** crystallizes with one molecule of pentane in the asymmetric unit. The pentane fragment could be successfully modeled using restraints on the corresponding bond distances (DFIX and SADI) and displacement parameters (SIMU 0.01 0.02). Additionally, rigid body (RIGU) restraints with standard uncertainties have been added to the corresponding atoms.

IV.2. Summary of crystal data

Table S1. Crystal data, data collection and refinement for compounds **1**–**3**.

**Compound**	**1**	**2**	**3**
CCDC Number	2118417	2118418	2118419
Chemical formula	$C_{34}H_{58}Tm$	$C_{70}H_{116}O_2Tm_2$	$C_{71}H_{116}O_3Tm_2$
Formula weight	635.73	1327.48	1355.49
Temperature / K	150.0	150.0	150.0
Crystal system	monoclinic	monoclinic	orthorhombic
Space group	$P2_1/c$	$P2_1/n$	$Pca2_1$
a / Å	19.4715(9)	18.1868(6)	21.2510(8)
b / Å	18.0905(10)	11.4972(4)	10.8765(4)
c / Å	19.5394(10)	31.4355(14)	28.7940(10)
α / °	90	90	90
β / °	109.702(2)	93.696(2)	90
γ / °	90	90	90
Volume / $Å^3$	6479.8(6)	6559.4(4)	6655.3(4)
Z	8	4	4
ρ_{calc} / g.cm^{-3}	1.303	1.344	1.353
μ/mm^{-1}	2.757	2.728	2.692
F(000)	2648.0	2760.0	2816.0
Crystal size/mm^3	0.12 × 0.08 × 0.02	0.20 × 0.16 × 0.04	0.38 × 0.22 × 0.08
Radiation	MoKα (λ = 0.71073)	MoKα (λ = 0.71073)	MoKα (λ = 0.71073)
2Θ range for data collection / °	3.158 to 53.582	2.518 to 56.01	3.744 to 56.88
Index ranges	-24 ≤ h ≤ 24, -22 ≤ k ≤ 22, -24 ≤ l ≤ 24	-23 ≤ h ≤ 23, -15 ≤ k ≤ 15, -4 ≤ l ≤ 41	-28 ≤ h ≤ 28, -13 ≤ k ≤ 14, -38 ≤ l ≤ 38
Reflections collected	54376	15689	111505
Independent reflections	13780 [R_{int} = 0.0736, R_{sigma} = 0.0784]	15689 [R_{int} = n.a.[a], R_{sigma} = 0.0760]	16567 [R_{int} = 0.0961, R_{sigma} = 0.0670]
Data/restraints/parameters	13780/246/729	15689/60/704	16567/1/721
Goodness-of-fit on F^2	1.006	1.140	1.014
Final R indexes [I>=2σ (I)]	R_1 = 0.0406, wR_2 = 0.0722	R_1 = 0.0609, wR_2 = 0.1371	R_1 = 0.0356, wR_2 = 0.0608
Final R indexes [all data]	R_1 = 0.0821, wR_2 = 0.0833	R_1 = 0.0784, wR_2 = 0.1432	R_1 = 0.0465, wR_2 = 0.0645
Largest diff. peak/hole / e.Å^{-3}	1.48/-1.42	2.12/-2.06	0.94/-1.10
Flack parameter	–	–	-0.028(6)

[a] not applicable, refined as a 2-component twin

Table S2. Crystal data, data collection and refinement for compounds **4**–**6**.

**Compound**	**4·C_5H_{12}**	**5·(0.45 C_5H_{12})**	**6**
CCDC Number	2118420	2118421	2118422
Chemical formula	$C_{69}H_{116}O_3Tm_2$·(C_5H_{12})	$C_{72}H_{116}O_5Tm_2$·(0.45 C_5H_{12})	$C_{109}H_{177}O_6Tm_3$
Formula weight	1403.62	1431.97	2090.29
Temperature / K	150.0	150.0	150.15
Crystal system	monoclinic	triclinic	monoclinic
Space group	*P*2/*n*	$P\bar{1}$	*I*2/*a*
a / Å	18.6229(11)	10.5960(13)	29.457(2)
b / Å	10.3489(5)	17.174(2)	16.1306(12)
c / Å	19.6817(12)	21.404(3)	46.333(4)
α / °	90	98.599(4)	90
β / °	111.234(3)	99.006(4)	105.969(5)
γ / °	90	106.877(4)	90
Volume / Å^3	3535.7(4)	3602.1(8)	21166(3)
Z	2	2	8
ρ_{calc} / g.cm^{-3}	1.318	1.320	1.312
μ/mm^{-1}	2.536	2.493	2.542
F(000)	1468.0	1490.0	8688.0
Crystal size/mm^3	0.08 × 0.08 × 0.040	0.40 × 0.26 × 0.12	0.12 × 0.10 × 0.04
Radiation	MoKα (λ = 0.71073)	MoKα (λ = 0.71073)	MoKα (λ = 0.71073)
2Θ range for data collection / °	4.44 to 56.88	1.968 to 55.306	3.216 to 55.112
Index ranges	-24 ≤ h ≤ 24, -13 ≤ k ≤ 13, -26 ≤ l ≤ 26	-13 ≤ h ≤ 13, -17 ≤ k ≤ 22, -27 ≤ l ≤ 27	-36 ≤ h ≤ 38, -20 ≤ k ≤ 20, -60 ≤ l ≤ 60
Reflections collected	211360	74423	142322
Independent reflections	8890 [R_{int} = 0.1047, R_{sigma} = 0.0349]	16616 [R_{int} = 0.0875, R_{sigma} = 0.0782]	24372 [R_{int} = 0.0858, R_{sigma} = 0.0635]
Data/restraints/parameters	8890/47/417	16616/0/748	24372/0/1118
Goodness-of-fit on F^2	1.029	1.043	1.006
Final R indexes [I>=2σ (I)]	R_1 = 0.0259, wR_2 = 0.0502	R_1 = 0.0609, wR_2 = 0.1533	R_1 = 0.0366, wR_2 = 0.0674
Final R indexes [all data]	R_1 = 0.0434, wR_2 = 0.0559	R_1 = 0.0835, wR_2 = 0.1680	R_1 = 0.0710, wR_2 = 0.0784
Largest diff. peak/hole / e.Å^{-3}	1.14/-0.92	4.55/-2.91	2.38/-1.87

Table S3. Crystal data, data collection and refinement for compounds **7**, **9a** and **9b**.

**Compound**	**7·(3 C_5H_{12})**	**9a·(7 C_5H_{12})**	**9b·C_5H_{12}**
CCDC Number	2118423	2118424	2128950
Chemical formula	$C_{78}H_{134}O_4Si_2Tm_2$· (3 C_5H_{12})	$C_{124}H_{188}O_{12}Tm_4$· (7 C_5H_{12})	$C_{122}H_{184}O_{12}Tm_4$·(C_5H_{12})
Formula weight	1746.32	3051.47	2590.55
Temperature / K	150.0	150.0	150.0
Crystal system	triclinic	triclinic	triclinic
Space group	$P\bar{1}$	$P\bar{1}$	$P\bar{1}$
a / Å	13.4591(11)	14.9953(7)	17.966(2)
b / Å	17.3610(14)	17.8272(9)	18.395(2)
c / Å	20.0159(14)	28.3472(16)	19.483(2)
α / °	65.739(2)	75.491(2)	83.851(3)
β / °	85.671(3)	82.620(2)	83.324(3)
γ / °	77.074(3)	70.215(2)	75.907(3)
Volume / Å^3	4155.2(6)	6895.2(6)	6182.0(10)
Z	2	2	2
ρ_{calc} / g.cm^{-3}	1.396	1.470	1.392
μ/mm^{-1}	2.201	2.610	2.898
F(000)	1852.0	3196.0	2660.0
Crystal size/mm^3	0.32 × 0.16 × 0.09	0.40 × 0.38 × 0.08	0.38 × 0.18 × 0.1
Radiation	MoKα (λ = 0.71073)	MoKα (λ = 0.71073)	MoKα (λ = 0.71073)
2Θ range for data collection / °	2.232 to 57.616	3.428 to 54.666	3.314 to 52.044
Index ranges	-18 ≤ h ≤ 18, -23 ≤ k ≤ 23, -27 ≤ l ≤ 27	-18 ≤ h ≤ 19, -21 ≤ k ≤ 22, -36 ≤ l ≤ 36	-22 ≤ h ≤ 22, -21 ≤ k ≤ 22, -24 ≤ l ≤ 24
Reflections collected	181424	112106	116323
Independent reflections	21582 [R_{int} = 0.0566, R_{sigma} = 0.0319]	30786 [R_{int} = 0.0559, R_{sigma} = 0.0574]	24333 [R_{int} = 0.0997, R_{sigma} = 0.0889]
Data/restraints/parameters	21582/0/817	30786/24/1364	24333/55/1344
Goodness-of-fit on F^2	1.044	1.014	1.036
Final R indexes [I>=2σ (I)]	R_1 = 0.0249, wR_2 = 0.0591	R_1 = 0.0350, wR_2 = 0.0801	R_1 = 0.0421, wR_2 = 0.0760
Final R indexes [all data]	R_1 = 0.0375, wR_2 = 0.0654	R_1 = 0.0531, wR_2 = 0.0879	R_1 = 0.0871, wR_2 = 0.0932
Largest diff. peak/hole / e.Å^{-3}	1.34/-0.78	1.34/-0.72	1.74/-0.86

IV.3. Crystal structures

IV.3.1. The crystal structure of **1**

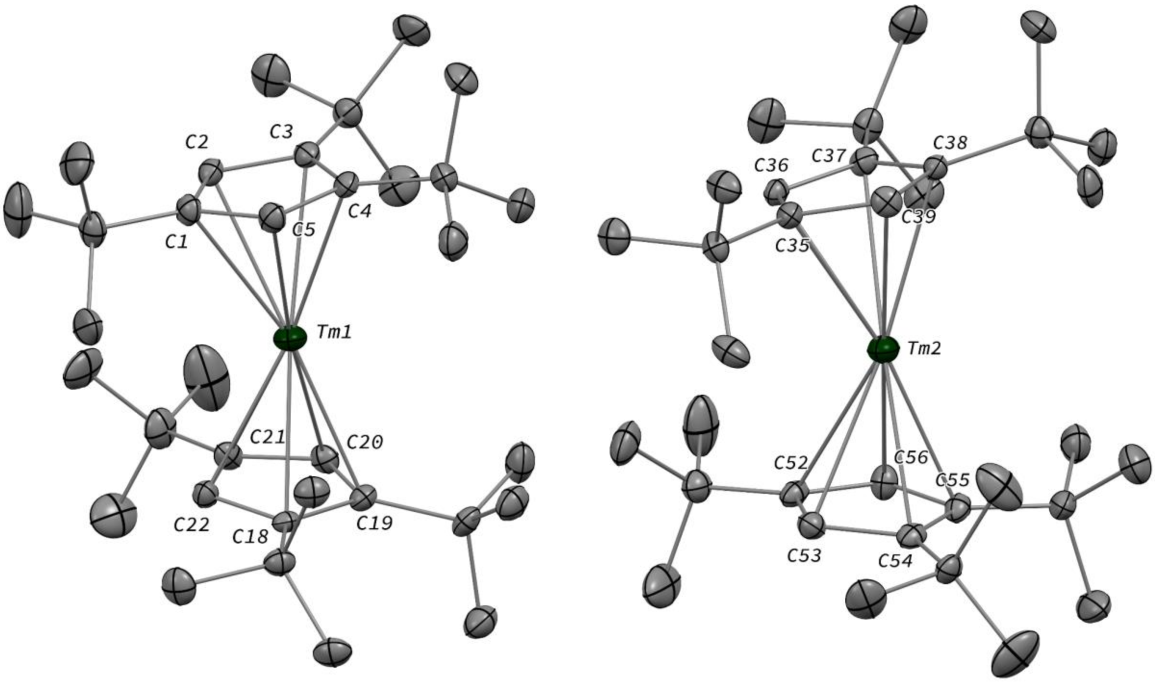

Fig. S41. Molecular structures of the two independent molecules of **1** in the solid state with thermal ellipsoids at the 40% probability level. Only one disordered position for the ^{t}Bu groups has been depicted and H atoms have been omitted for clarity. Selected bond distances (Å): Tm1–C1 2.712(4), Tm1–C2 2.676(4), Tm1–C3 2.681(4), Tm1–C4 2.664(4), Tm1–C5 2.670(4), Tm1–C18 2.673(4), Tm1–C19 2.677(4), Tm1–C20 2.686(4), Tm1–C21 2.703(4), Tm1–C22 2.658(4), Tm2–C35 2.684(4), Tm2–C36 2.668(4), Tm2–C37 2.705(4), Tm2–C38 2.699(4), Tm2–C39 2.652(4), Tm2–C52 2.717(4), Tm2–C53 2.675(4), Tm2–C54 2.661(4), Tm2–C55 2.656(4), Tm2–C56 2.671(4).

Table S4. Selected geometric parameters for **1** and related complexes (distances in Å and angles in °).

	[Tm(Cp^{ttt})$_2$] (**1**)[a]	[Sm(Cp^{ttt})$_2$][a,b]	[Tm(Cp^{ttt})$_2$I][c]	[Tm(Cp^{ttt})$_2$(THF)][c]
Ln–C	2.652(4)–2.717(4)	2.757(4)–2.839(5)	2.587(5)–2.756(5)	2.709(4)–2.791(3)
Ln–Cp(ctr)	2.390/2.393<br>2.387/2.393	2.523/2.529<br>2.513/2.519	2.370/2.372	2.463/2.479
Σ(Ln–Cp(ctr))	4.783<br>4.780	5.052<br>5.032	4.742	4.942
Cp(ctr)–Ln–Cp(ctr)	167.7<br>164.3	161.1<br>163.5	147.6	148.6
Mean Cp^Cp angle	11.0<br>13.9	17.0<br>13.5	35.1	31.8

[a] Both of the two independent molecules present in the asymmetric unit are considered;
[b] data from ref 9;
[c] data from ref 3.

IV.3.2. The crystal structure of **2**

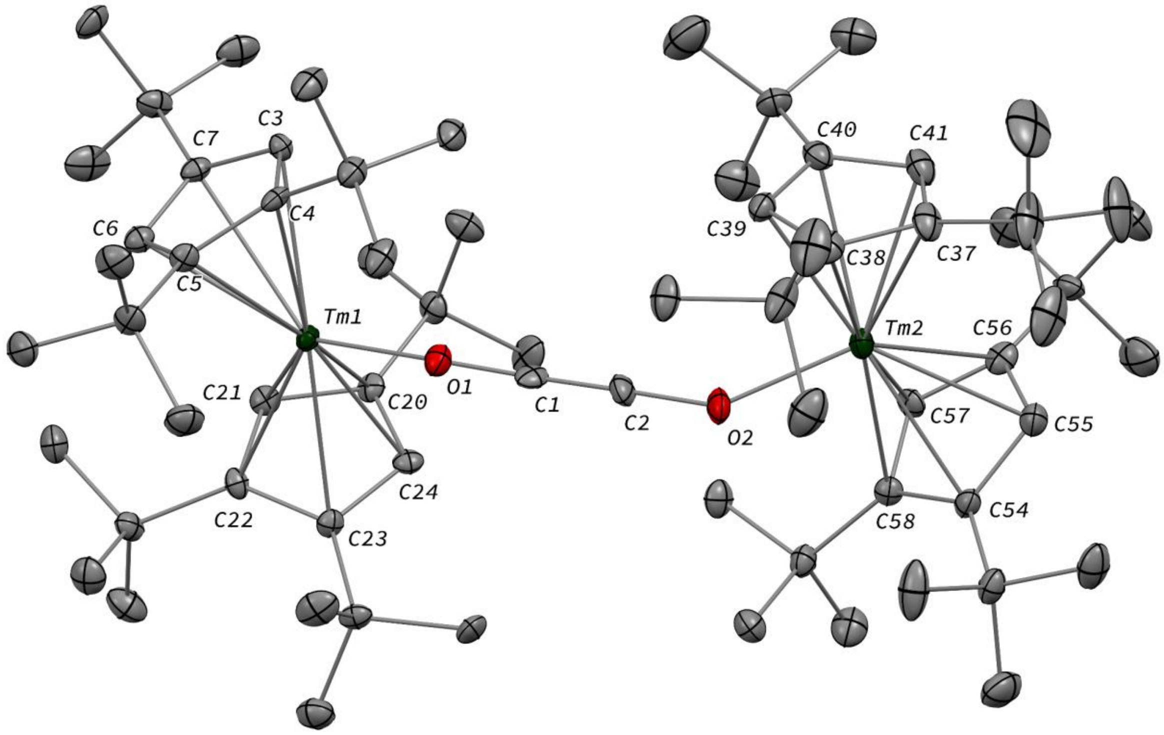

Fig. S42. Molecular structure of **2** in the solid state with thermal ellipsoids at the 40% probability level. H atoms have been omitted for clarity. Selected bond distances (Å) and angles [°]: C1–C2 1.226(10), C1–O1 1.265(9), C2–O2 1.296(9), Tm1–O1 2.066(5), Tm2–O2 2.078(5); C2–C1–O1 178.4(8), C1–C2–O2 178.1(9), C1–O1–Tm1 151.9(5), C2–O2–Tm2 146.7(5).

Table S5. Selected geometric parameters for **2**–**5** (distances in Å and angles in °).

	[{Tm(Cp^{ttt})$_2$}$_2$(CO)$_2$] (**2**)	[{Tm(Cp^{ttt})$_2$}$_2$(CO)$_3$] (**3**)	[{Tm(Cp^{ttt})$_2$}$_2$(CO$_3$)] (**4**)	[{Tm(Cp^{ttt})$_2$}$_2$(C$_4$O$_5$)] (**5**)
Tm–C	2.568(8)–2.730(8)	2.601(6)–2.769(6)	2.617(3)–2.712(3)	2.618(7)–2.733(7)
Tm–Cp(ctr)	2.333/2.375	2.403/2.390	2.369/2.372	2.367/2.368
	2.373/2.319	2.356/2.366		2.371/2.375
Σ(Tm–Cp(ctr))	4.708	4.793	4.741	4.735
	4.692	4.722		4.746
Tm–O	2.066(5)/2.078(5)	2.098(5)/2.307(5)	2.330(3)/2.322(3)	2.282(5)/2.354(5)
			2.018(4)	2.305(5)/2.318(5)
Cp(ctr)–Tm–	145.6	141.5	144.0	144.6
Cp(ctr)	146.1	144.5		147.0
Mean Cp^Cp angle	34.2	38.3	35.6	32.2
	33.0	35.2		35.0

IV.3.3. The crystal structure of **3**

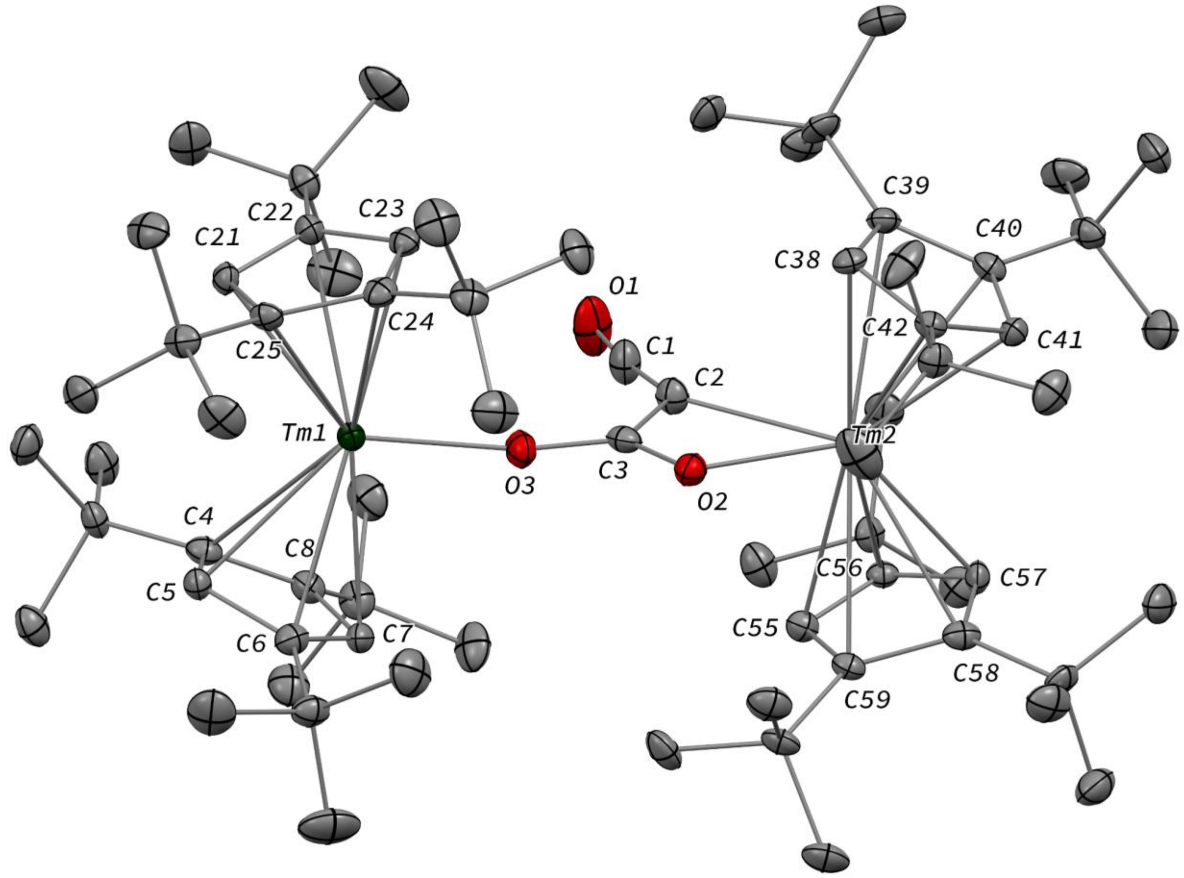

Fig. S43. Molecular structure of **3** in the solid state with thermal ellipsoids at the 40% probability level. H atoms have been omitted for clarity. Selected bond distances (Å) and angles [°]: O1–C1 1.181(9), O2–C3 1.261(8), O3–C3 1.296(8), C1–C2 1.284(10), C2–C3 1.430(9), Tm1–O3 2.098(5), Tm2–O2 2.307(5), Tm2–C2 2.473(7); C1–C2–C3 128.6(7), C3–O2–Tm2 99.2(4), C3–O3–Tm1 170.8(4), O1–C1–C2 172.2(8), O2–C3–C2 116.0(6), O3–C3–C2 123.5(6), C1–C2–Tm2 143.5(5), C3–C2–Tm2 87.5(4).

IV.3.4. The crystal structure of **4**

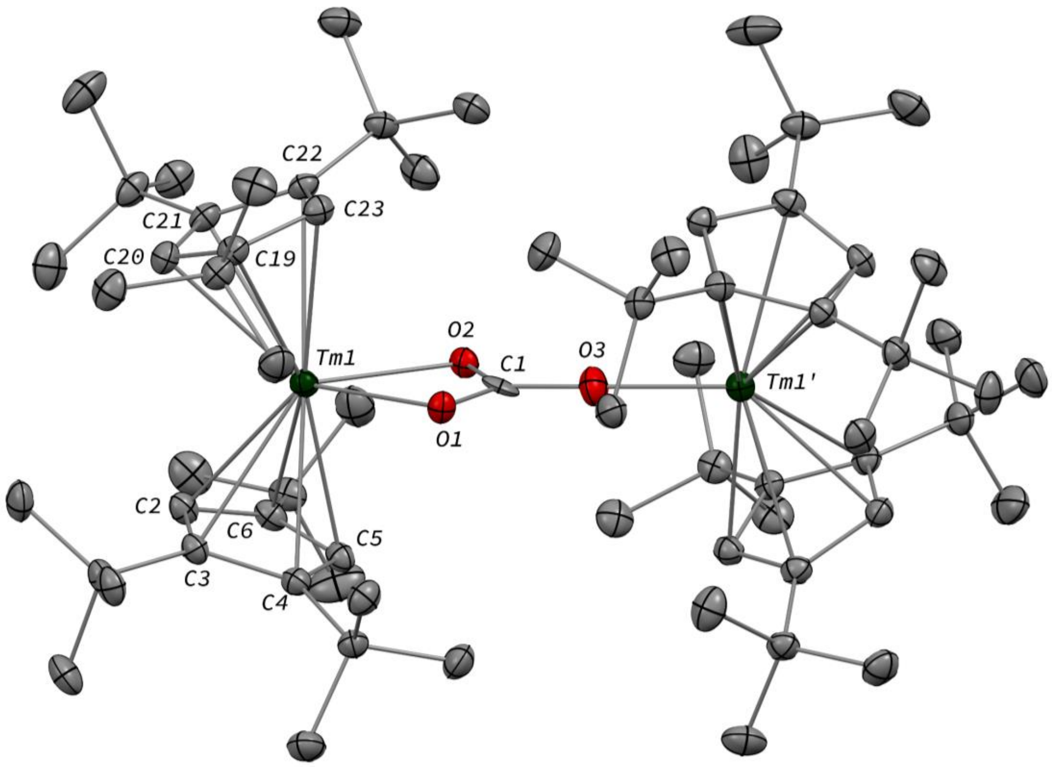

Fig. S44. Molecular structure of **4** in the solid state with thermal ellipsoids at the 40% probability level. H atoms and non-coordinating solvent molecules have been omitted and only one disordered position for the carbonate ligand is depicted for clarity. Selected bond distances (Å) and angles [°]: Tm1-O1 2.330(3), Tm1-O2 2.322(3), Tm1′-O3 2.018(4), O1-C1 1.283(10), O2-C1 1.275(9), O3-C1 1.286(10); O1-Tm1-O2 56.27(12), C1-O1-Tm1 92.5(4), C1-O2-Tm1 93.1(4), C1-O3-Tm1′ 179.3(8), O1-C1-O3 120.2(9), O1-C1-O2 118.1(6), O2-C1-O3 121.7(9). Sum of the angles around C1 = 360.0°

Atoms with the prime character in the atom labels (′) are at equivalent position (3/2-x, y, 1/2-z).

In this centrosymmetric structure, the CO_3^{2-} ligand is disordered over two positions: in each of them, the oxocarbon anion is coordinating one Tm center in a κ[1](*O*) mode and the other Tm center in a chelating κ[2](*O*′,*O*′′) coordination mode. The Tm–C bond distances in **4** are ranging from 2.617(3) to 2.712(3) Å (2.67 ± 0.04 Å in average) with Tm–Cp(ctr) separations of 2.369 and 2.372 Å, very similar to those in the ketenedicarboxylate complex **5** (see Table S5). The C–O bond distances in the carbonate ligand are identical within experiment error (1.275(9)–1.286(10) Å), pointing to delocalization of the negative charge and C=O double bond.

IV.3.5. The crystal structure of **5**

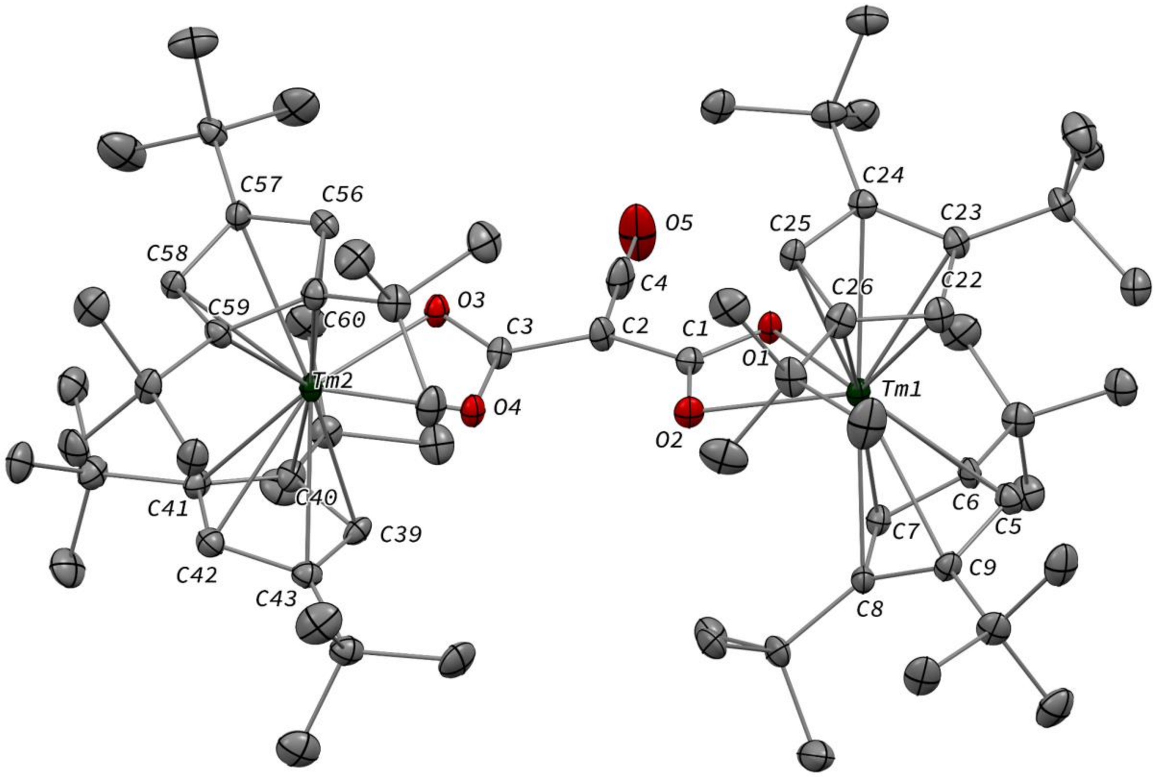

Fig. S45. Molecular structure of **5** in the solid state with thermal ellipsoids at the 40% probability level. H atoms have been omitted for clarity. Selected bond distances (Å) and angles [°]: Tm1-O1 2.282(5), Tm1-O2 2.354(5), Tm1-C1 2.671(7), Tm2-O3 2.305(5), Tm2-O4 2.318(5), Tm2-C3 2.661(7), O1-C1 1.283(8), O2-C1 1.263(8), O3-C3 1.273(8), O4-C3 1.267(8), O5-C4 1.142(10), C1-C2 1.460(9), C2-C3 1.461(10), C2-C4 1.343(10); O1-Tm1-O2 56.9(2), O3-Tm2-O4 57.0(2), O1-C1-C2 116.0(6), O2-C1-O1 120.3(6), O2-C1-C2 123.6(6), C1-C2-C3 131.7(6), C4-C2-C1 113.0(7), C4-C2-C3 115.3(6), O3-C3-C2 118.1(6), O4-C3-O3 120.5(6), O4-C3-C2 121.4(6), O5-C4-C2 178.5(10).

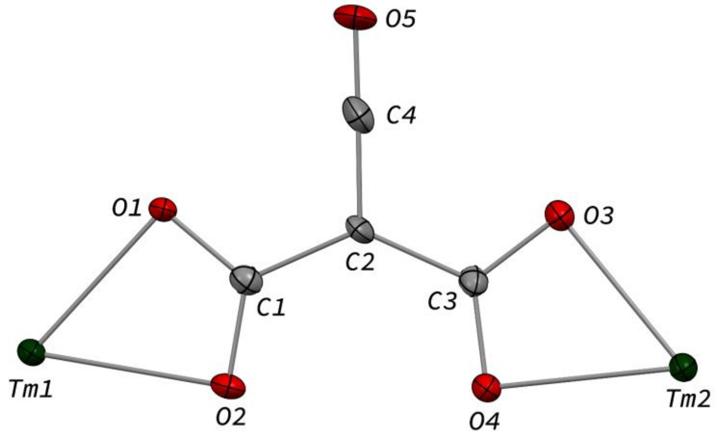

Fig. S46. Simplified view of the {μ-κ^2(*O*):κ^2(*O*)-C_4O_5} core in the molecular structure of **5**.

IV.3.6. The crystal structure of **6**

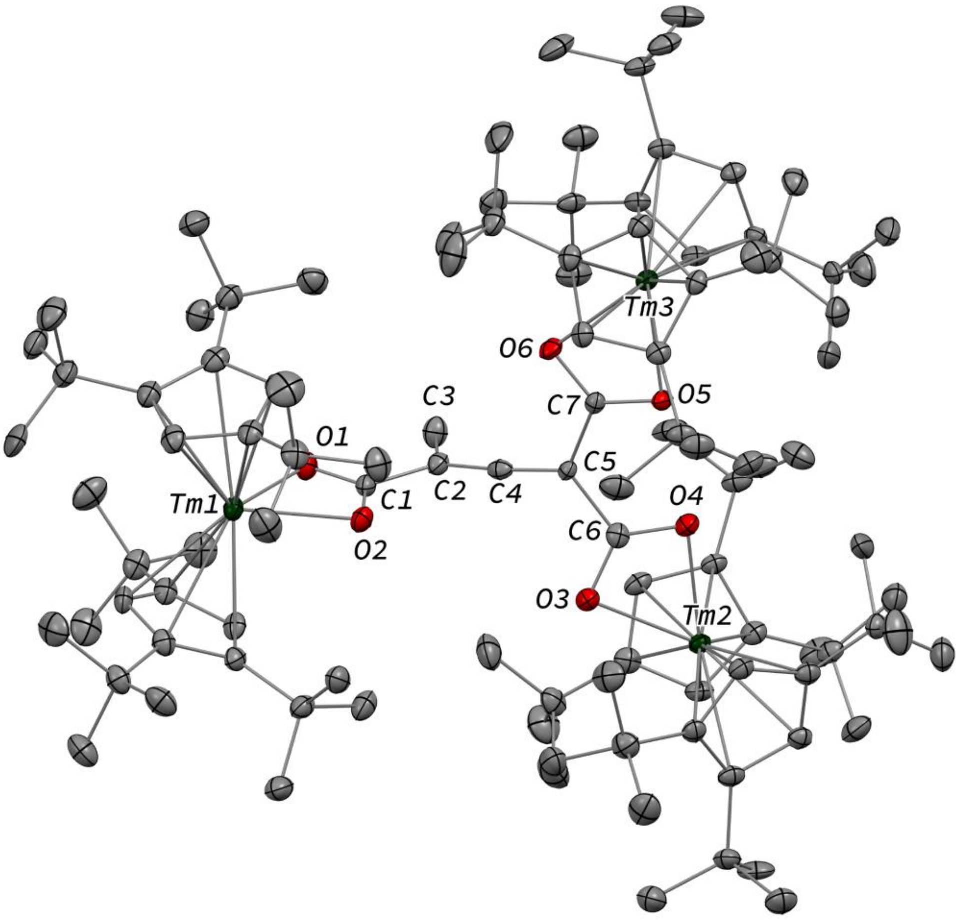

Fig. S47. Molecular structure of **6** in the solid state with thermal ellipsoids at the 40% probability level. H atoms have been omitted for clarity. Selected bond distances (Å) and angles [°]: Tm1-O1 2.299(3), Tm1-O2 2.315(3), Tm2-O3 2.319(3), Tm2-O4 2.307(3), Tm3-O5 2.304(3), Tm3-O6 2.333(3), O1-C1 1.274(5), O2-C1 1.268(5), O3-C6 1.284(5), O4-C6 1.269(5), O5-C7 1.271(4), O6-C7 1.268(4), C1-C2 1.486(6), C2-C3 1.501(6), C2-C4 1.300(6), C4-C5 1.325(5), C5-C6 1.512(5), C5-C7 1.492(5); O1-Tm1-O2 56.88(10), O4-Tm2-O3 57.64(10), O5-Tm3-O6 57.01(9), O1-C1-C2 118.1(4), O2-C1-O1 119.6(4), O2-C1-C2 122.2(4), C1-C2-C3 116.6(4), C4-C2-C1 121.1(4), C4-C2-C3 122.3(4), C2-C4-C5 172.2(4), C4-C5-C6 117.6(3), C4-C5-C7 119.4(3), C7-C5-C6 122.3(3), O3-C6-C5 118.2(4), O4-C6-O3 121.8(4), O4-C6-C5 119.9(3), O5-C7-C5 119.7(3), O6-C7-O5 121.4(4), O6-C7-C5 119.0(3).

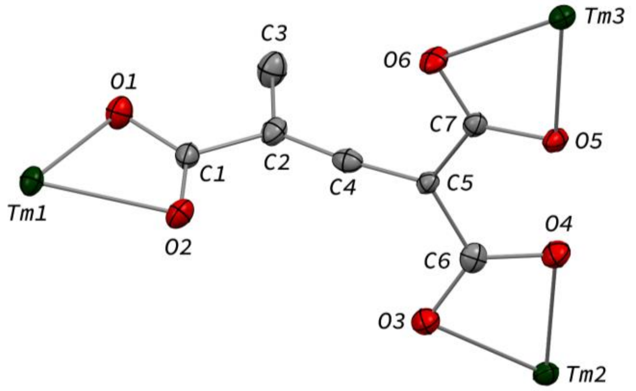

Fig. S48. Simplified view of the {μ_3-κ^2(*O*):κ^2(*O*):κ^2(*O*)-$H_3CC_3(CO_2)_3$} core in the molecular structure of **6**.

IV.3.7. The crystal structure of **7**

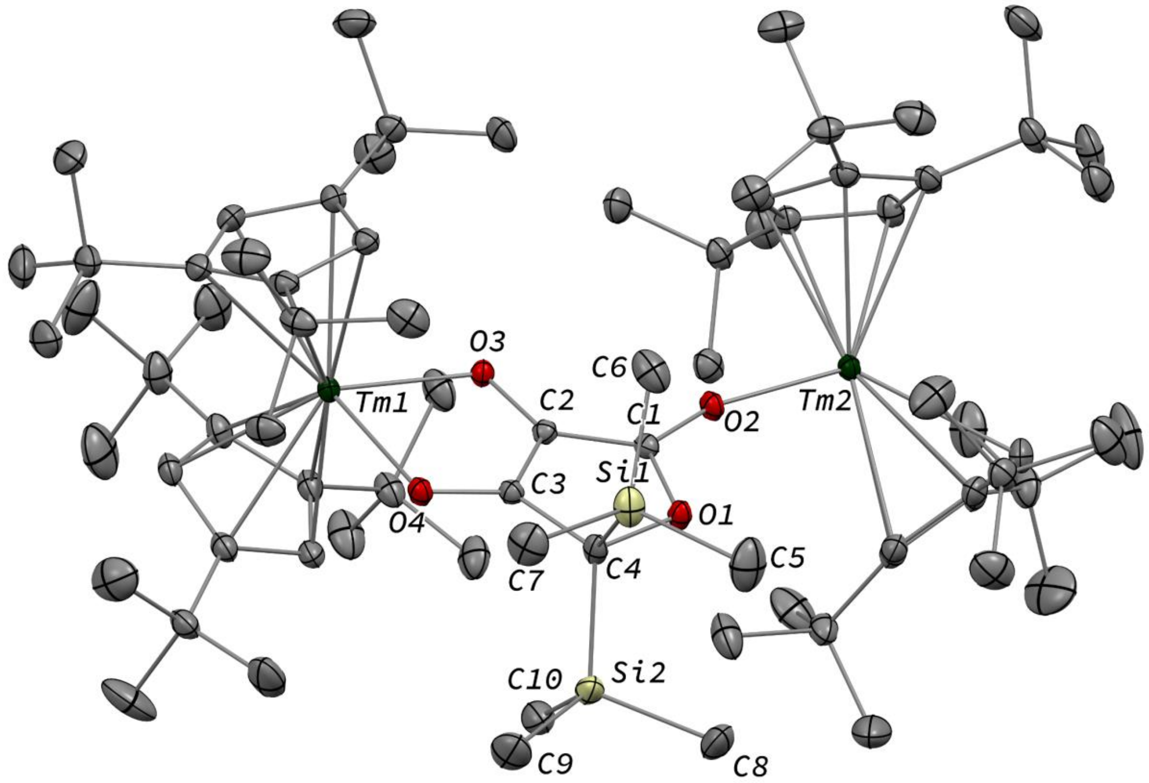

Fig. S49. Molecular structure of **7** in the solid state with thermal ellipsoids at the 40% probability level. H atoms have been omitted for clarity. Selected bond distances (Å) and angles [°]: Tm1-O3 2.200(2), Tm1-O4 2.333(2), Tm2-O2 2.146(2), Si1-C4 1.917(3), Si2-C4 1.933(3), O1-C1 1.368(3), O1-C4 1.486(3), O2-C1 1.280(3), O3-C2 1.339(3), O4-C3 1.290(3), C1-C2 1.397(3), C2-C3 1.396(3), C3-C4 1.494(3); O3-Tm1-O4 74.78(6), C1-O2-Tm2 163.1(2), C2-O3-Tm1 112.88(13), C3-O4-Tm1 109.86(14).

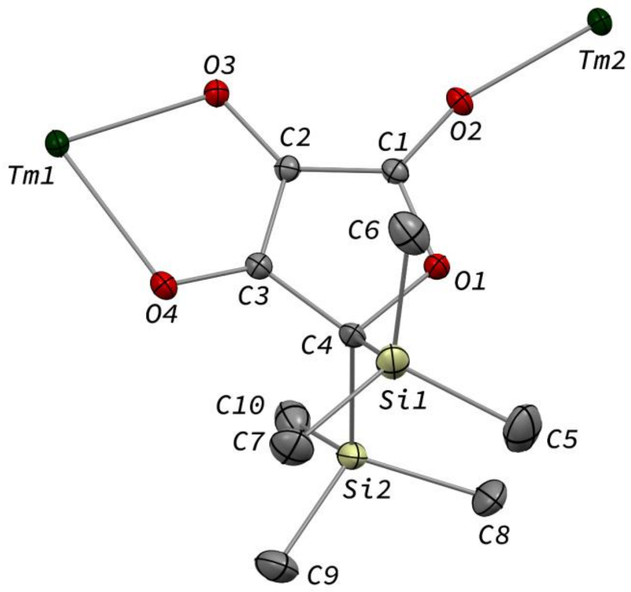

Fig. S50. Simplified view of the {μ-κ(*O*):κ^2(*O*′,*O*′′)-$C_4O_4(SiMe_3)_2$} core in the molecular structure of **7**.

IV.3.8. The crystal structure of **9a**

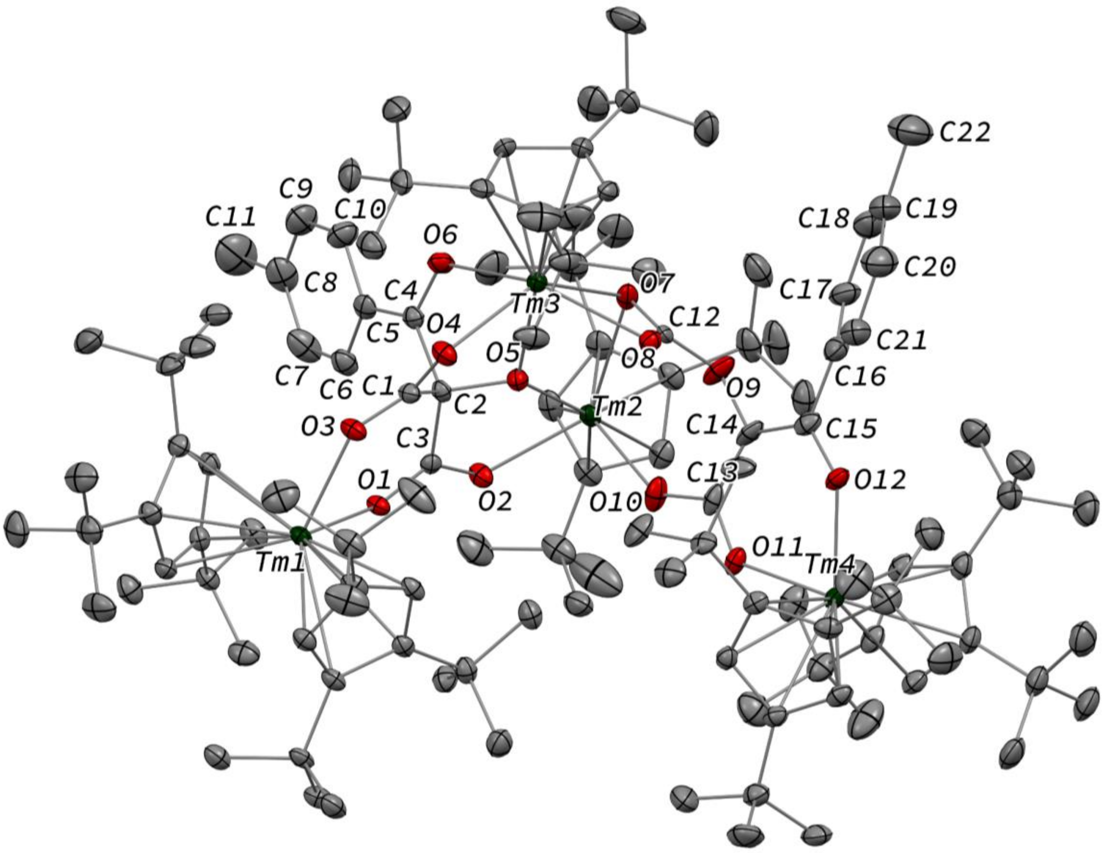

Fig. S51. Molecular structure of **9a** in the solid state with thermal ellipsoids at the 40% probability level. H atoms and non-coordinating solvent molecules have been omitted for clarity. Selected bond distances (Å): Tm1-O1 2.273(3), Tm1-O3 2.242(3), Tm2-O2 2.266(3), Tm2-O5 2.263(3), Tm2-O7 2.643(3), Tm2-O10 2.156(3), Tm2-C12 2.968(4), Tm3-O4 2.261(3), Tm3-O5 2.294(3), Tm3-O6 2.386(3), Tm3-O7 2.428(3), Tm3-O8 2.333(3), Tm4-O11 2.234(3), Tm4-O12 2.195(3), O1-C3 1.244(4), O2-C3 1.252(5), O3-C1 1.248(4), O4-C1 1.259(4), O5-C2 1.402(4), O6-C4 1.235(5), O7-C12 1.271(4), O8-C12 1.231(5), O9-C12 1.338(5), O9-C14 1.428(5), O10-C13 1.291(5), O11-C13 1.260(5), O12-C15 1.300(5), C1-C2 1.565(5), C2-C3 1.550(5), C2-C4 1.567(5), C13-C14 1.426(6), C14-C15 1.384(6).

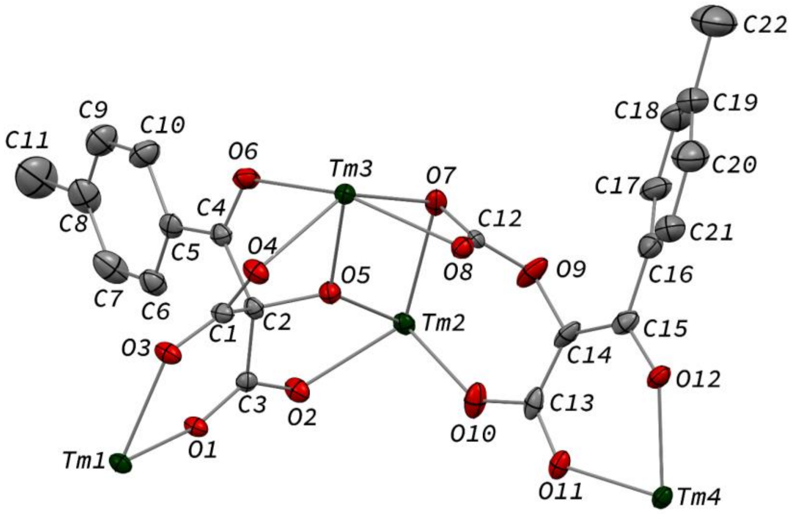

Fig. S52. Simplified view of the two {μ_3-$C_7H_7(C_4O_6)$} cores in the molecular structure of **9a**.

IV.3.9. The crystal structure of **9b**

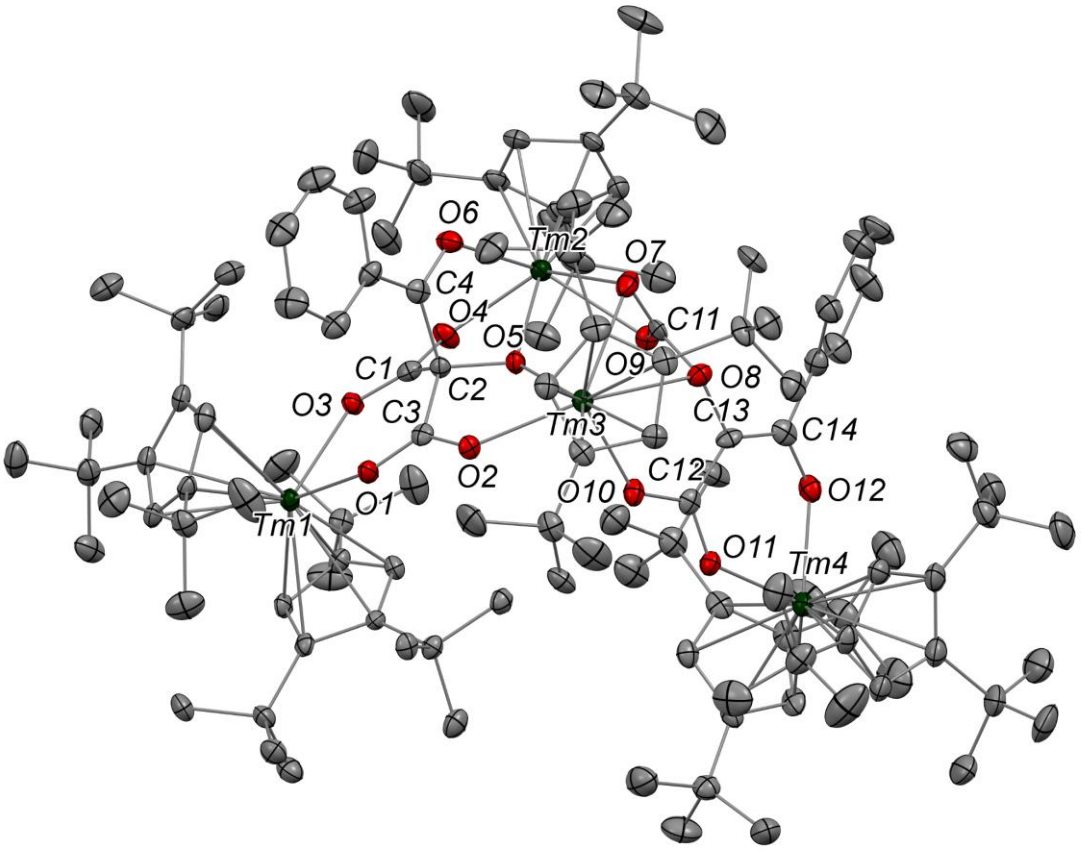

Fig. S53. Molecular structure of **9b** in the solid state with thermal ellipsoids at the 40% probability level. H atoms and non-coordinating solvent molecules have been omitted for clarity. Selected bond distances (Å): Tm1-O1 2.286(4), Tm1-O3 2.243(4), Tm2-O4 2.259(4), Tm2-O5 2.330(4), Tm2-O6 2.373(4), Tm2-O7 2.413(4), Tm2-O9 2.345(4), Tm3-O2 2.274(4), Tm3-O5 2.277(4), Tm3-O7 2.765(4), Tm3-O8 2.677(4), Tm3-O10 2.165(4), Tm4-O11 2.229(4), Tm4-O12 2.188(4), Tm2-C11 2.661(6), Tm3-C11 2.931(6), O1-C3 1.242(6), O2-C3 1.276(6), O3-C1 1.243(6), O4-C1 1.263(7), O5-C2 1.405(6), O6-C4 1.242(7), O7-C11 1.251(7), O8-C11 1.351(7), O8-C13 1.435(6), O9-C11 1.252(7), O10-C12 1.294(7), O11-C12 1.258(7), C1-C2 1.572(8), C2-C3 1.544(8), C2-C4 1.572(8), C4-C5 1.467(8), C5-C6 1.403(8), C12-C13 1.452(8), C13-C14 1.365(8).

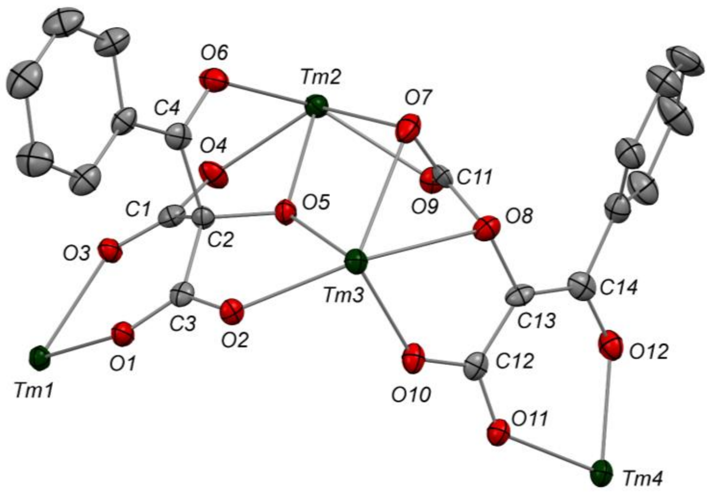

Fig. S54. Simplified view of the two {μ_3-$C_6H_5(C_4O_6)$} cores in the molecular structure of **9b**.

V. DFT calculations

V.1. Mechanistic insights in the formation of 2 and 3

Computational Details:

The calculations were conducted using a DFT focused methodology – using the Becke's 3-parameter hybrid functional combined with the non-local correlation functions designed by Perdew/Yang (B3PW91).[10] All calculations were computed using the Gaussian09 software.[11] Metallic atoms (**Tm**) were treated with a small-core Stuttgart Dresden relativistic effective core potential[12] (SDDALL) in combination with a double quality basis set[13] – SDDALL. Carbon, Oxygen and Hydrogen atoms were described using a double ζ 6-31G** basis set including d and p polarization respectively.[14] Additional single-point calculations were performed using a triple ζ 6-311G** basis set[15] (for the corresponding energetic profile, see Fig. S63) or the functionals wB97xd[16] and M062X[17] (the corresponding energetic profiles are depicted in Fig. S64 and S65, respectively). Geometry optimizations of the various structures were computed without symmetry constraints. Furthermore, frequency analyses enabled the computation of force constants and vibrational frequencies on the system. Natural Bonding Orbital (NBO) analyses enabled deeper understanding of the nature of the bonding within these systems.[18]

Using this methodology, the following report outlines the mechanism proposed.

a. Coordination of CO

Initial investigations involved three various modes of coordination for the CO ligand, either through an 'end-on' attack through either the carbon or oxygen atoms *vs.* a 'side-on' (η^2) coordination. This investigation concluded a CO coordination would occur only through the η^2-type interaction, requiring 16.3 kcal·mol^{-1}.

b. First coupling reaction

A radical coupling is proposed due to the densities present throughout the carbonyl ligand. The interaction of the formed radical species enables the formation of a new C–C bond. The adduct generated (30.6 kcal·mol^{-1}) prior to the coupling is largely due to the bulkiness of the Cp^{ttt} system which would hinder a close coordination site. The transition state barrier (33.4 kcal·mol^{-1}) is therefore relatively dependent on the sterics about the Tm complex. Once the coupling occurs, the product is thermodynamically stabilizing and will subsequently drive the mechanism further. To demonstrate this type of coupling reaction, a more detailed bonding orbital analysis (NBO) was conducted regarding the structure and electronic density through this transition state (**TS1**) (Fig. S55–S56).

The other typical mechanisms described in the literature in the case of CO reductive coupling, such as μ^2-CO coordination[19] and C−C coupling by a $[\mu_2\text{-}\eta^1\text{:}\eta^1\text{-CO}]_2$ intermediate,[20] have been tested and only the radical mechanism was found to be realistic. Because of the high steric demand of the ligand, μ_2-CO coordination and formation of a doubly-reduced CO sandwiched between two oxidized fragments[19] is impossible here. Every attempt led to the decoordination of one of the $(Cp^{ttt})_2Tm$ fragments because of steric effects. Similarly, C−C coupling on an $[\mu_2\text{-}\eta^1\text{:}\eta^1\text{-CO}]_2$ intermediate[20] is also difficult because of the important sterics of the Cp^{ttt} ligand. In the same way, computationally, this complex was found not to be stable either. Although the steric repulsion is reduced with respect to the side-on coordination of CO with an end-on coordination, this is not sufficient and one $(Cp^{ttt})_2Tm$ fragment dissociates away. This steric effect was ensured by a calculation using a Cp* ligand, in which case the formation of a $[\mu_2\text{-}\eta^1\text{:}\eta^1\text{-CO}]_2$ intermediate was possible.

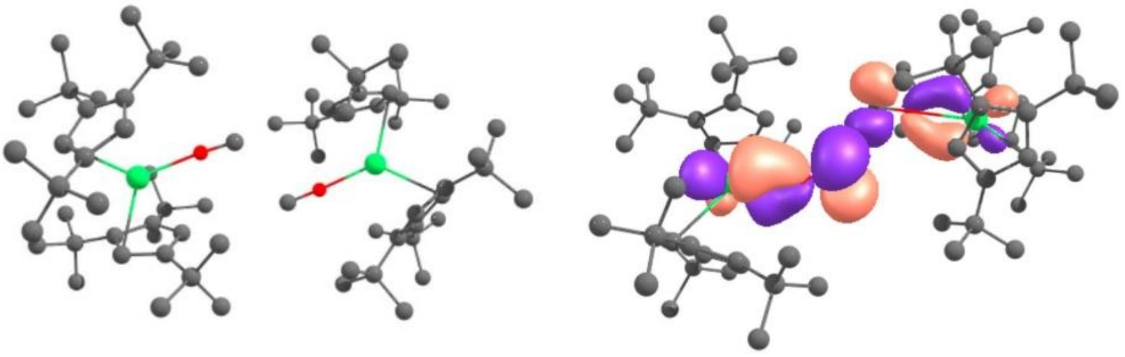

Fig. S55. Geometry of **TS1** (left) and HOMO of the system (right). Note the occupation of the O–C bond highlighting the radical nature of this coupling.

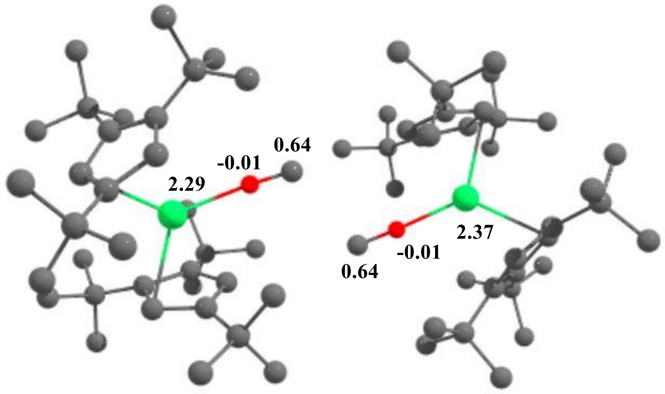

Fig. S56. Spin densities present on the carbonyl atoms and the Tm metal.

The above analysis highlights the fact that this coupling reaction involves radicals being present on each of the C atoms, both present in the HOMO of the system. It is further validated by the spin densities on each of the metallic or carbonyl atoms (2.37, -0.01 and 0.64 respectively). The product (**iso2**) presents a 'zig-zag' form as the C–C bond is formed. Notably, the electronic density is largely placed on the C atoms, as validated by analysis of the HOMO (Fig. S57).

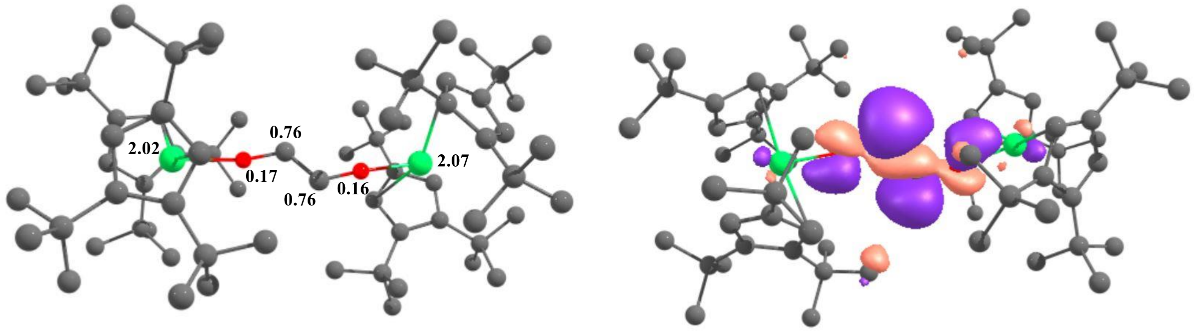

Fig. S57. Product of the first coupling (left) and HOMO of this complex (**iso2**), located on the O–CC–O bond (right). Note the 'zig-zag' form of the C–C bond.

c. Formation of the CO trimerized product 3

The formation of complex **3** involves the location of two transition states: the first one corresponds to the coordination and insertion of the CO molecule, resembling a 2,1-type insertion into the CO bond (**TS2**) (Fig. S58–S59), followed by a rearrangement (**TS3**) to form the desired product.

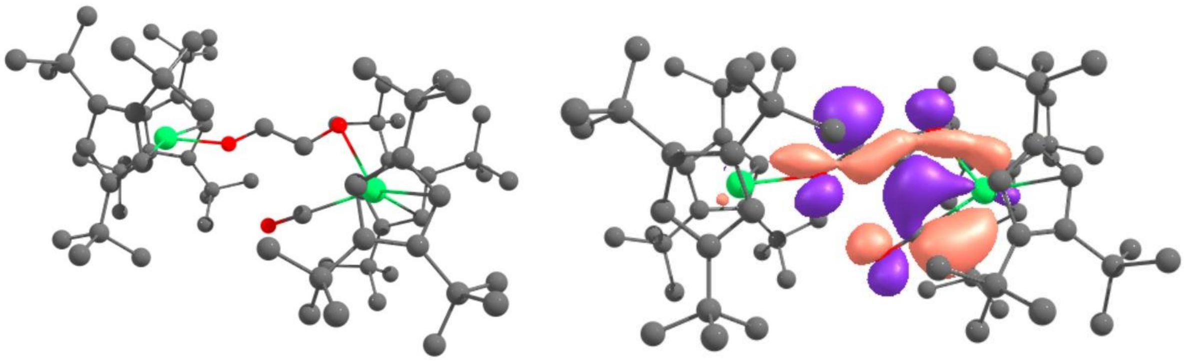

Fig. S58. Geometry of the second transition state (**TS2**) (left) and HOMO of this system (right).

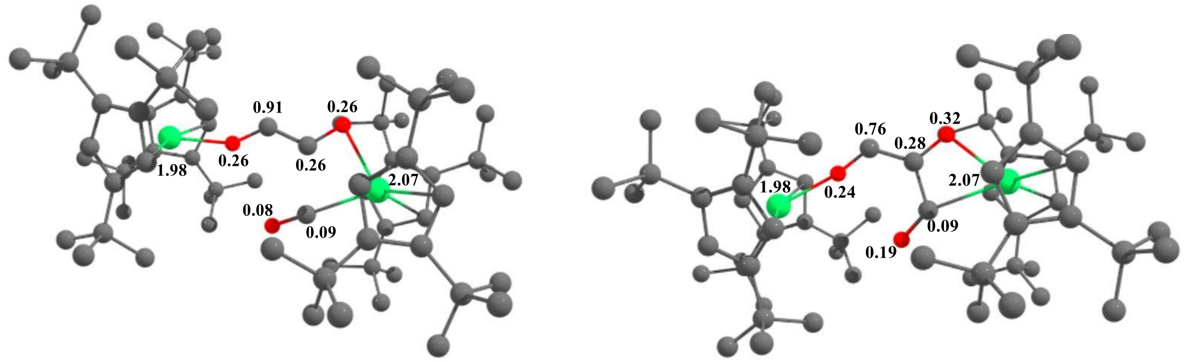

Fig. S59. The various spin densities about **TS2** (left) and the spin densities about the product of the CO insertion (right). Note that most of the density remains on the Tm metal adjacent to the CO insertion.

The product of **TS2** is a CO trimerized intermediate (Fig. S59, right) in which the **Tm** atom on the right retains its interaction with one oxygen and one carbon atoms. This intermediate demands a further rearrangement (**TS3**) to form the CO trimerized product **3** observed experimentally (Fig. S60–S61).

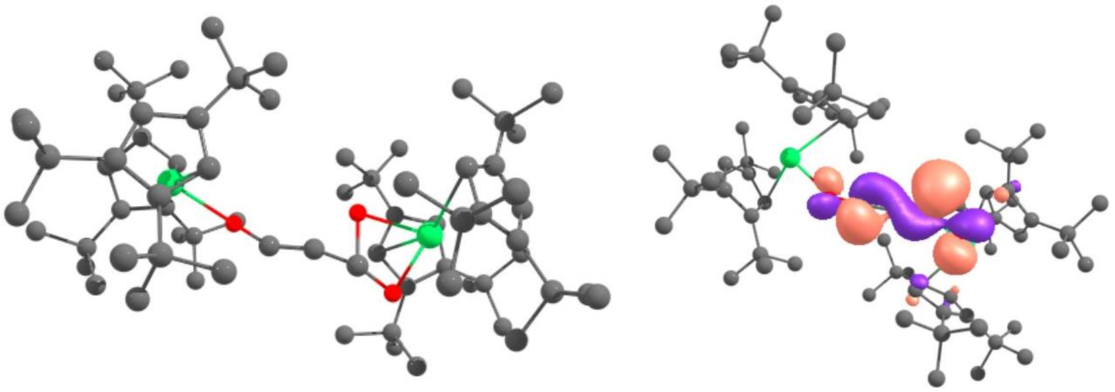

Fig. S60. Geometry of **TS3** (left) along with the HOMO of the system presenting a π interaction localized across the O–C–C– O bond (right).

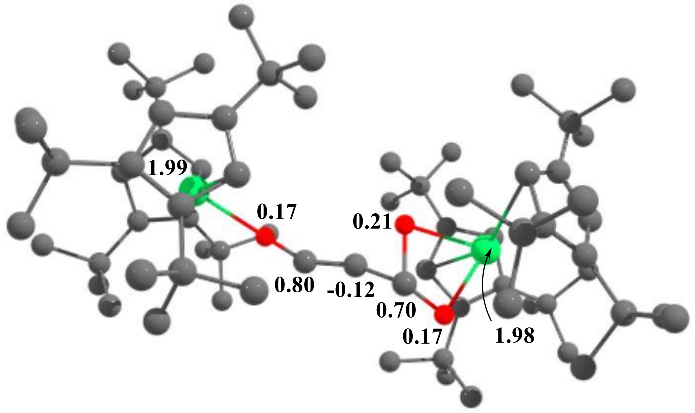

Fig. S61. The spin densities about this transition state (**TS3**).

The obtained product (**Iso3**) is considered an isomer compared to the experimental, X-ray authenticated product **3** (Fig. S62). Both computed isomers are shown below, with the experimental structure **3** being more stable by 65.4 kcal·mol^{-1}.

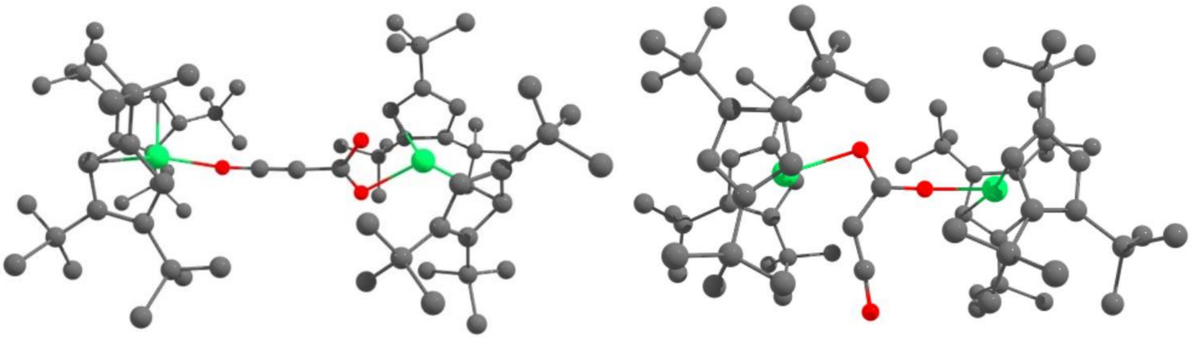

Fig. S62. Structures of both CO trimerized products, **Iso3** (left, the product of **TS3** in the profile) and **3** (right, the experimentally observed and more stable product).

The product of this CO trimerization reaction is thus explored further to understand the bonding that exists between the carbon, oxygen and metal atoms where possible.

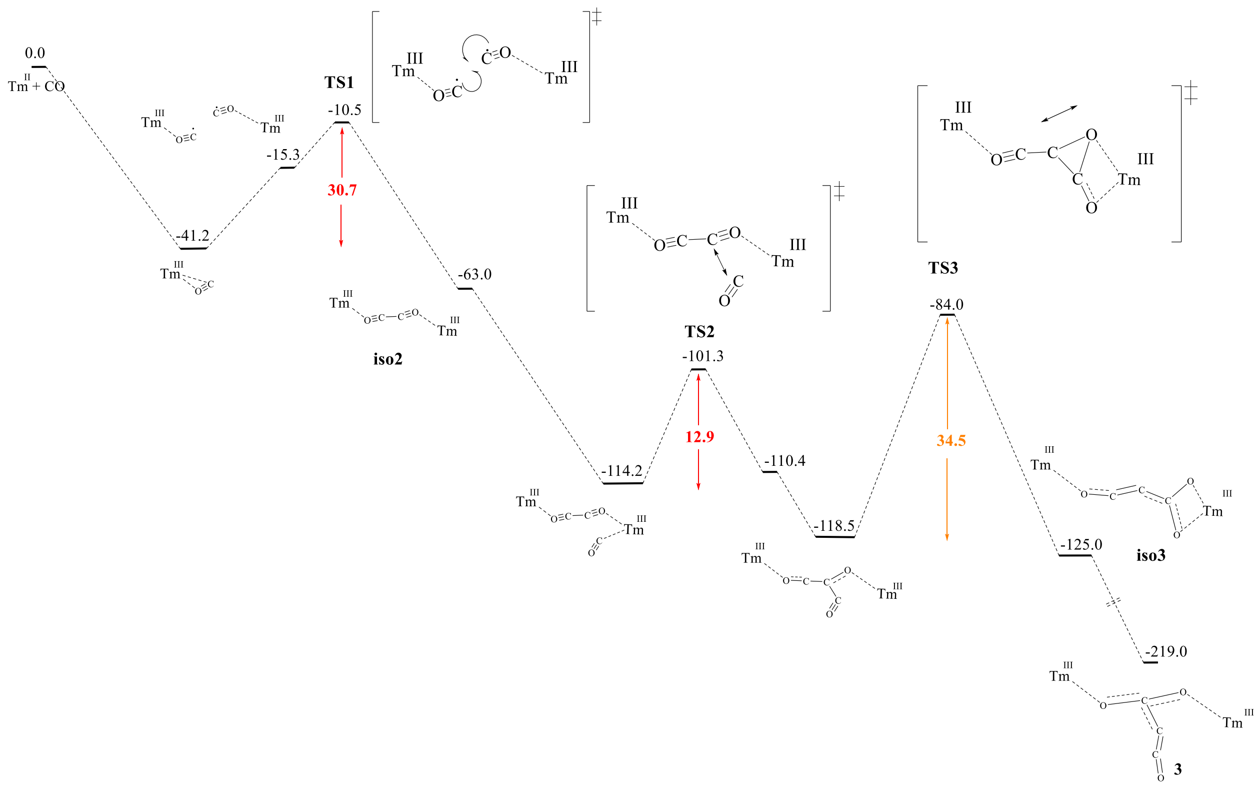

Fig. S63. Energies from single-point calculations on the different species using a larger basis set (triple-zeta basis set).

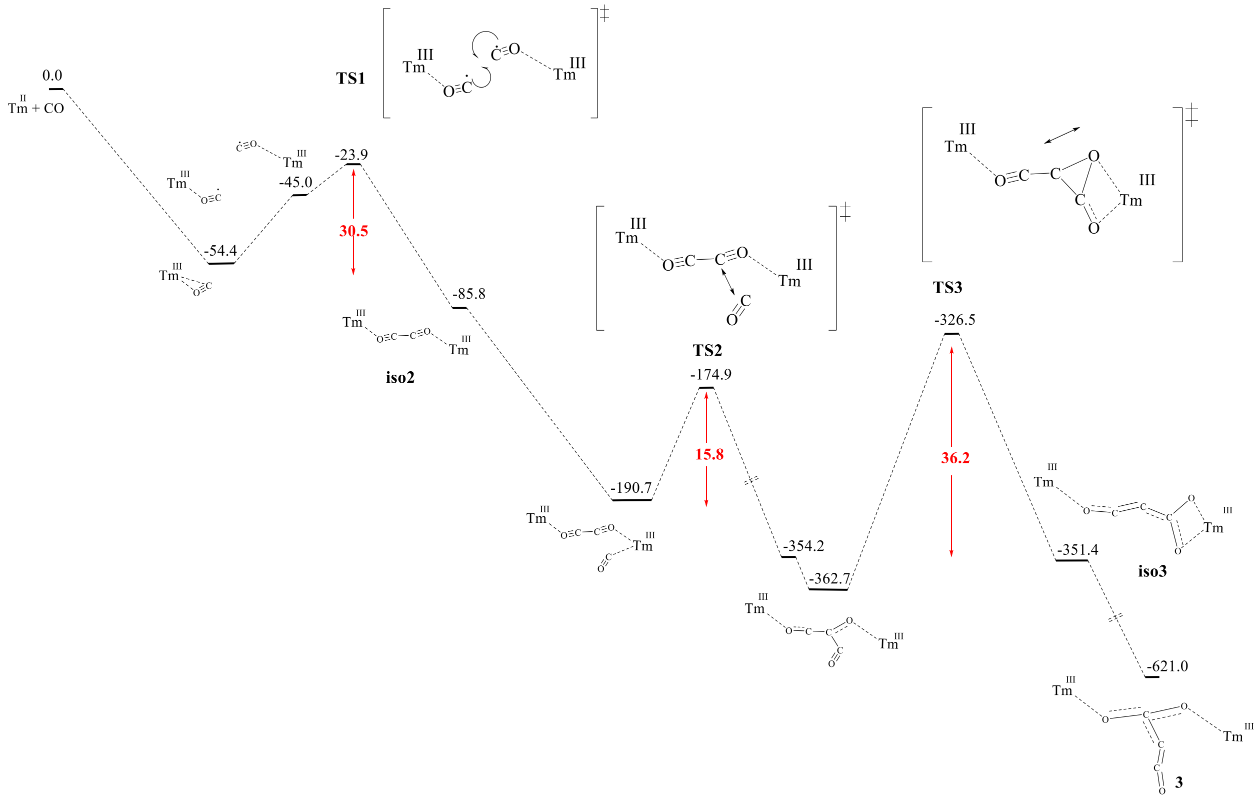

Fig. S64. Energies from single-point calculations using the wB97xd functional.

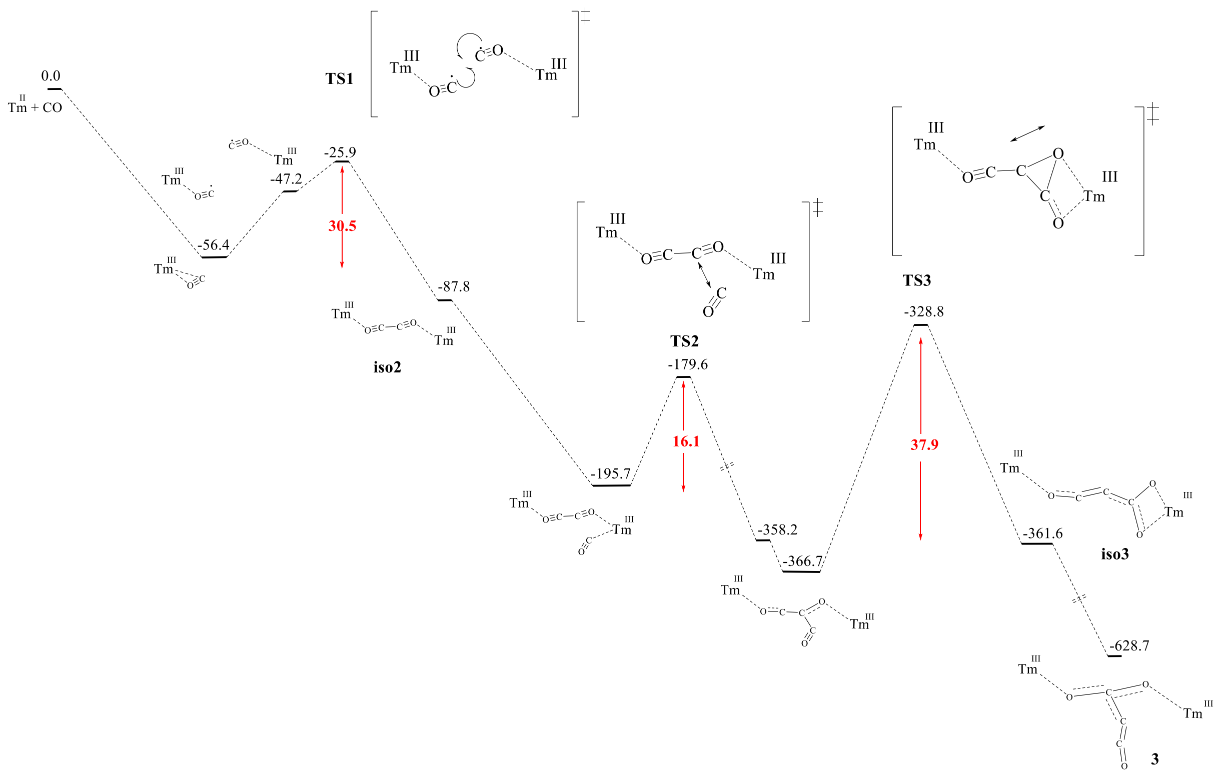

Fig. S65. Energies from single-point calculations using the M062X functional.

V.2. Computed IR spectra of 3 and 3-^{13}C

The IR spectrum for complex **3** was computed at the same level of theory and is presented below (Fig. S66) and the corresponding vibration modes are discussed.

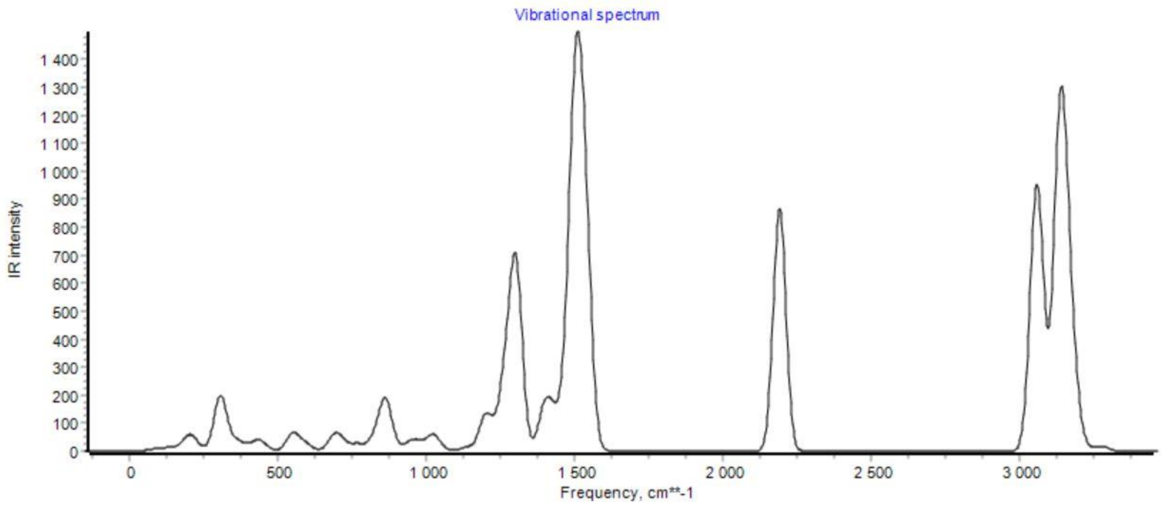

Fig. S66. Computed IR spectrum of **3**.

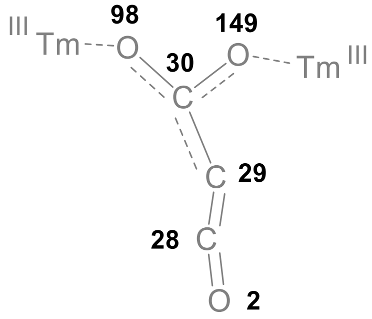

Fig. S67. The structure of complex **3**, labelled as to distinguish the different modes about the atoms.

Table S6. The various modes corresponding to the computed IR spectrum of **3**. Note the intensity at *ca.* 1300 cm^{-1} corresponds almost exclusively to an asymmetric stretch about all C–C and O–C bonds.

**Frequency (cm^{-1})**	**Mode**
550.56	Stretch – about C_{28} and C_{149}
578.13	Stretch – about C_{29} and C_{98}
723.53	'Wiggle' – about C_{28}, C_{29}, C_{30} $O_{98,}$ and C_{149}
961.85	Stretch – about C_{30}
1301.85	Asymmetric Stretch – general
2189.50	Asymmetric Stretch – about C_{28}, C_{29} and O_2

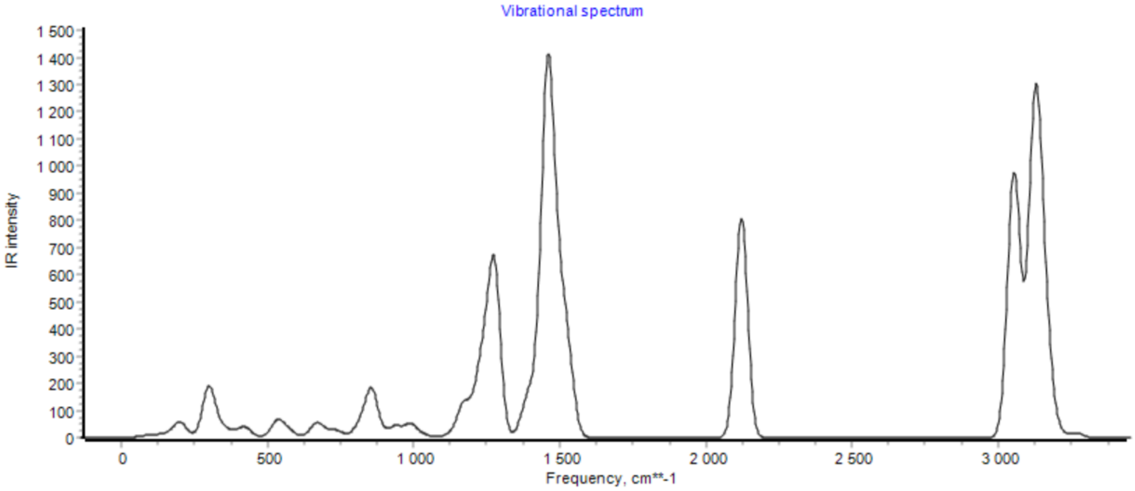

Fig. S68. Computed IR spectrum of ^{13}C labelled **3-^{13}C**.

Table S7. The various modes corresponding to the computed IR spectrum of **3-^{13}C** and comparison with the modes in **3**.

	**Frequency (cm^{-1})**	
**Mode**	**C^{12}**	**C^{13}**
Stretch – about C_{28} and C_{149}	550.56	539.21
Stretch – about C_{29} and C_{98}	578.13	572.10
'Wiggle' – about C_{28}, C_{29}, C_{30} $O_{98,}$ and C_{149}	723.53	705.36
Stretch – about C_{30}	961.85	945.03
Asymmetric Stretch – general	1301.85	1275.91
Asymmetric Stretch – about C_{28}, C_{29} and O_2	2189.50	2121.05

V.3. Computed coordinates

! CO !

C 0.000000 0.000000 0.146493

O 0.000000 0.000000 1.283507

! Optimized complex **1** !

Spin multiplicity: 2

C 2.995919 13.013345 10.894580
C 4.408011 12.853562 11.052456
C 5.022091 14.086340 11.368940
C 3.971906 15.030206 11.440987
C 2.717036 14.415236 11.141992
Tm 3.510312 13.382468 13.509482
C 2.335307 13.899781 15.864821
C 2.617596 12.497596 15.883806
C 4.061706 12.377235 15.943947
C 4.580109 13.707245 15.939415
C 3.527248 14.654350 15.912124
C 1.458506 11.490622 16.036981
C 0.127360 12.119640 15.572538
C 5.014609 11.164577 15.994157
C 5.067123 10.437898 14.632830
C 3.659664 16.152789 16.155843
C 4.780349 16.762064 15.298684
C 6.522566 14.353205 11.401568
C 7.091092 14.140477 9.983246
C 1.424733 15.256313 11.087861
C 0.710906 15.133601 9.728774
C 2.164236 11.840228 10.332542
C 0.732277 11.728545 10.881497
C 1.273171 11.137477 17.529372
C 1.611161 10.199094 15.215149
C 7.232612 13.388776 12.366952
C 6.825251 15.793308 11.835343
C 0.454151 14.900715 12.233502
C 1.746802 16.755131 11.254735
C 2.838480 10.490306 10.659289
C 2.112139 11.946810 8.792077
C 2.349684 16.889845 15.845371
C 4.001189 16.373479 17.644985
C 4.656102 10.166788 17.110384
C 6.455570 11.621821 16.298281
H 4.110254 16.086000 11.630067
H 4.946169 11.931269 10.869958
H 6.382993 16.519250 11.145432
H 7.907199 15.965907 11.849739

H	6.440206	16.000744	12.839018
H	6.927980	13.113091	9.642749
H	8.169548	14.340221	9.959813
H	6.603458	14.809413	9.266916
H	6.889571	13.542197	13.398342
H	8.317602	13.547627	12.355166
H	7.048723	12.343143	12.098142
H	2.205436	16.973767	12.224579
H	0.819461	17.335240	11.195146
H	2.417948	17.114798	10.468805
H	1.390062	15.410894	8.916069
H	-0.153326	15.807683	9.691900
H	0.345720	14.126170	9.527706
H	0.141339	13.855566	12.216426
H	-0.452923	15.515761	12.183204
H	0.917911	15.101866	13.209451
H	1.617277	12.862107	8.461306
H	1.571194	11.094345	8.362334
H	3.125385	11.951884	8.378371
H	0.727425	11.613363	11.972673
H	0.243195	10.841618	10.462512
H	0.107670	12.585911	10.631382
H	3.812594	10.381251	10.175757
H	2.208653	9.668578	10.301571
H	2.979628	10.350753	11.738037
H	5.629189	13.960519	16.024358
H	1.340809	14.325476	15.882609
H	3.221730	15.951665	18.287640
H	4.091976	17.443029	17.871713
H	4.946861	15.888085	17.906198
H	5.741711	16.271701	15.483490
H	4.905537	17.828086	15.522650
H	4.553244	16.670210	14.230000
H	2.051090	16.758561	14.799149
H	2.466916	17.964216	16.025785
H	1.528892	16.537558	16.478369
H	6.859733	12.272479	15.516892
H	7.111141	10.746397	16.361578
H	6.513605	12.155026	17.252147
H	4.089998	10.068292	14.313149
H	5.746259	9.577117	14.668953
H	5.457880	11.104925	13.850428
H	4.624859	10.674137	18.079961
H	5.415770	9.378150	17.167685
H	3.694119	9.677606	16.957829
H	0.176201	12.472010	14.535531
H	-0.175669	12.961919	16.200114
H	-0.671252	11.372094	15.630248

H	2.152188	10.648484	17.951859
H	0.414423	10.468066	17.665340
H	1.090825	12.046891	18.110576
H	1.693214	10.415271	14.141879
H	0.725962	9.566300	15.346872
H	2.476486	9.601674	15.502298

! Eta2 coordinated CO to **1** !

Spin multiplicity: 4

C	3.331177	14.755563	16.076631
C	2.273618	13.899127	15.693649
C	2.707421	12.537315	15.634996
C	4.129435	12.556847	15.920939
C	4.466853	13.917128	16.195091
Tm	3.938708	13.881210	13.636592
C	2.814051	14.438244	11.264036
C	2.940112	12.991755	11.246659
C	4.340783	12.722512	11.281428
C	5.101965	13.913778	11.236890
C	4.145789	14.956882	11.255637
C	2.007149	11.858464	10.762579
C	1.939097	11.937493	9.218020
C	6.574645	13.992555	10.831927
C	7.138993	15.415151	10.950969
C	1.595256	15.384295	11.200881
C	2.050082	16.857320	11.232582
C	1.641341	11.426730	15.742595
C	1.911636	10.159748	14.922530
C	5.212807	11.456203	15.942192
C	6.578326	12.044302	16.351907
C	3.164394	16.189459	16.576871
C	2.284303	16.130751	17.845494
C	0.255847	11.950820	15.312907
C	1.511075	11.049502	17.237328
C	5.399675	10.858779	14.533731
C	4.916651	10.334132	16.954207
C	4.508904	16.820923	16.965577
C	2.466540	17.081365	15.538599
C	6.647898	13.581559	9.342609
C	7.452568	13.029614	11.645272
C	0.802712	15.220030	9.889286
C	0.653390	15.196777	12.405299
C	0.579782	11.862992	11.323199
C	2.609464	10.474306	11.084330
H	4.391617	16.009142	11.254427
H	4.770499	11.731854	11.209734
H	6.556559	16.129284	10.359478

H	8.166581	15.436766	10.571610
H	7.162361	15.763497	11.986490
H	6.295285	12.555774	9.194952
H	7.680759	13.641032	8.978931
H	6.030252	14.241569	8.724506
H	7.463542	13.308158	12.702477
H	8.484227	13.054238	11.275618
H	7.101595	11.995240	11.559900
H	2.624046	17.095598	12.133619
H	1.168871	17.507748	11.223389
H	2.658836	17.114649	10.360279
H	1.463893	15.316580	9.022086
H	0.037509	16.001835	9.817735
H	0.289957	14.260023	9.819161
H	0.317265	14.165993	12.519866
H	-0.234110	15.832687	12.302120
H	1.152015	15.489426	13.337195
H	1.468344	12.857578	8.869588
H	1.362833	11.092032	8.822262
H	2.944609	11.894319	8.788171
H	0.579079	11.754257	12.410593
H	0.020074	11.016771	10.908774
H	0.023802	12.766217	11.070989
H	3.504724	10.269319	10.490655
H	1.881599	9.692281	10.844124
H	2.876642	10.370221	12.138732
H	5.452258	14.255855	16.479911
H	1.242288	14.213375	15.598846
H	1.288220	15.734819	17.623568
H	2.162238	17.132998	18.273617
H	2.738385	15.488293	18.607081
H	5.013282	16.232870	17.739566
H	4.341442	17.824055	17.373529
H	5.179614	16.915370	16.109666
H	3.075078	17.192036	14.636215
H	2.296241	18.082427	15.951094
H	1.489288	16.675477	15.255533
H	6.909115	12.833587	15.670346
H	7.332968	11.250667	16.331809
H	6.554134	12.449944	17.368369
H	4.480771	10.422719	14.137055
H	6.167177	10.075220	14.539362
H	5.753017	11.626050	13.828400
H	4.775118	10.746584	17.958375
H	5.764856	9.640967	16.994239
H	4.032763	9.746428	16.702420
H	0.274340	12.404074	14.318263
H	-0.136774	12.693150	16.013630

H	-0.457882	11.120680	15.290471
H	2.424535	10.604224	17.633954
H	0.695586	10.329003	17.375016
H	1.285319	11.936961	17.836875
H	1.928926	10.382772	13.853284
H	1.113176	9.428632	15.093033
H	2.852072	9.674055	15.183827
C	6.028812	15.070806	13.999957
O	5.177078	15.912890	13.862437

! **TS1**: Adduct !

Spin multiplicity: 5

C	12.966295	0.438760	16.883064
C	13.178794	1.175150	18.074970
C	14.515349	1.645662	17.989099
C	15.153695	1.148395	16.808095
C	14.160394	0.369424	16.100491
Tm	13.261013	2.930942	16.106864
C	11.262986	4.625687	16.252504
C	11.941772	4.939827	15.055548
C	13.227803	5.489511	15.323786
C	13.388404	5.468225	16.770181
C	12.164392	4.929042	17.298600
C	14.060531	6.097307	14.171659
C	14.278730	7.608393	14.384184
C	14.369994	6.164409	17.737401
C	15.836101	6.191057	17.281896
C	9.805773	4.186196	16.339297
C	9.622003	2.797547	15.699787
C	12.356136	1.057367	19.356374
C	13.000721	-0.068646	20.197462
C	16.657380	1.399847	16.565955
C	17.452845	0.083336	16.472226
C	14.234375	-0.667427	14.957204
C	15.122588	-0.293814	13.759402
O	12.974360	2.412290	13.991353
C	12.752759	2.180568	12.830419
C	13.883336	7.613018	17.968943
C	14.367699	5.468927	19.113453
C	13.298109	5.969990	12.837823
C	15.408510	5.387884	13.953331
C	8.940223	5.198710	15.563290
C	9.325666	4.161600	17.795467
C	10.904095	0.654989	19.065498
C	12.373058	2.344449	20.190442
C	16.904563	2.259142	15.313517
C	17.275831	2.164831	17.753349

C	12.830803	-0.970648	14.388470
C	14.740055	-1.997253	15.566749
C	10.085530	4.393715	11.620207
O	9.628900	3.914768	10.614345
Tm	8.882164	2.930331	8.797046
C	10.002877	0.936652	7.615086
C	10.420996	0.807669	8.958735
C	11.263987	1.912409	9.207843
C	11.438476	2.699957	8.033781
C	10.611612	2.087227	7.003797
C	10.184704	-0.344938	9.928056
C	9.457641	0.150587	11.191614
C	12.496954	3.828127	8.016174
C	11.907552	5.219645	7.728297
C	10.569596	2.211202	5.465250
C	10.716032	3.635750	4.912310
C	6.677077	4.328495	9.547001
C	6.422139	2.930930	9.705404
C	6.356420	2.278411	8.448793
C	6.660977	3.281048	7.491409
C	6.835609	4.552123	8.125693
C	6.439385	5.267106	10.751610
C	7.343716	6.507416	10.828419
C	5.641724	0.958934	8.164826
C	4.176869	1.310769	7.817351
C	7.035100	5.827807	7.279020
C	8.417805	6.460108	7.513108
C	13.179923	3.940878	9.393613
C	13.625614	3.507181	7.015693
C	9.236479	1.674983	4.905901
C	11.691008	1.323277	4.879319
C	9.380066	-1.469380	9.265214
C	11.554228	-0.916415	10.346080
C	6.247309	0.205817	6.973847
C	5.627236	0.044993	9.397486
C	5.928598	6.869790	7.531931
C	6.949076	5.497357	5.775693
C	4.959617	5.718211	10.710653
C	6.611653	4.510517	12.086921
H	15.019011	2.196848	18.771189
H	12.053864	-0.091388	16.646374
H	13.394349	2.657149	20.429128
H	11.846861	2.189291	21.139081
H	11.883211	3.167881	19.664801
H	13.019078	-1.010267	19.639585
H	12.435527	-0.229475	21.123522
H	14.031623	0.182230	20.466418
H	10.398159	1.382861	18.425435

H	10.338383	0.582599	20.000842
H	10.847947	-0.321817	18.574517
H	16.798783	3.135457	17.919600
H	18.334851	2.354094	17.548576
H	17.215135	1.589409	18.682592
H	17.267294	-0.543948	17.350174
H	18.526132	0.303403	16.436944
H	17.214032	-0.500125	15.583244
H	16.453827	1.838585	14.414059
H	17.979587	2.376901	15.130115
H	16.496278	3.266497	15.453334
H	15.749743	-1.914804	15.972196
H	14.746010	-2.783430	14.802033
H	14.081206	-2.320345	16.379272
H	14.782700	0.622378	13.269380
H	15.079443	-1.096944	13.015325
H	16.171751	-0.170910	14.029556
H	12.181551	-1.449624	15.127406
H	12.930001	-1.673561	13.554929
H	12.327801	-0.078116	14.009827
H	11.934541	4.862123	18.352342
H	11.520390	4.807112	14.068038
H	13.398194	5.529631	19.614629
H	15.098017	5.953912	19.770348
H	14.639849	4.412431	19.028868
H	16.223742	5.179986	17.116865
H	16.452903	6.653944	18.060650
H	15.989974	6.765235	16.368854
H	13.867131	8.194308	17.044925
H	14.538339	8.127425	18.682833
H	12.868272	7.612827	18.378719
H	16.051090	5.408539	14.833949
H	15.959601	5.864566	13.133827
H	15.244309	4.343041	13.667074
H	13.320074	8.118614	14.523424
H	14.764086	8.040650	13.501329
H	14.909120	7.836654	15.244395
H	13.118175	4.927853	12.561571
H	13.899489	6.420269	12.040610
H	12.334019	6.485605	12.859365
H	8.279109	3.841789	17.844425
H	9.392766	5.155876	18.249855
H	9.911206	3.471790	18.410317
H	10.210916	2.031146	16.221956
H	9.925632	2.804917	14.647677
H	8.572392	2.482873	15.743756
H	7.880495	4.927547	15.639979
H	9.206770	5.220123	14.502934

H	9.063634	6.208601	15.968191
H	11.737356	2.108320	10.160664
H	9.401397	0.213682	7.082944
H	14.058823	2.524076	7.227468
H	14.424254	4.252619	7.106796
H	13.296603	3.511400	5.975659
H	11.402841	5.282763	6.764025
H	12.702594	5.974992	7.731406
H	11.194687	5.497799	8.512402
H	12.473214	4.206395	10.184204
H	13.935388	4.733010	9.351961
H	13.684454	3.015588	9.685375
H	12.680481	1.639210	5.215787
H	11.676174	1.359192	3.783049
H	11.553778	0.281813	5.186931
H	9.932977	4.297653	5.296749
H	10.620648	3.616792	3.820770
H	11.681795	4.084963	5.140942
H	9.089622	0.611615	5.110928
H	9.221748	1.795224	3.817207
H	8.380976	2.220188	5.316041
H	12.120796	-1.252062	9.471160
H	11.417322	-1.775288	11.014083
H	12.151797	-0.168718	10.875004
H	10.044871	0.916598	11.709220
H	9.289393	-0.674222	11.894403
H	8.474053	0.575674	10.950019
H	8.395256	-1.131114	8.931004
H	9.224658	-2.289675	9.974311
H	9.909671	-1.874305	8.396306
H	6.202817	2.457785	10.652811
H	6.639710	3.128615	6.421330
H	6.636862	-0.197280	9.740789
H	5.119727	-0.896903	9.162414
H	5.089524	0.505336	10.232451
H	3.703607	1.862840	8.635601
H	3.593546	0.399796	7.635922
H	4.124776	1.933297	6.918511
H	6.256488	0.821644	6.069063
H	5.659826	-0.692686	6.752904
H	7.274643	-0.105517	7.178293
H	7.707551	4.773564	5.462457
H	7.112524	6.411227	5.194776
H	5.965273	5.102259	5.503022
H	8.603318	6.697803	8.561299
H	8.526691	7.383846	6.931847
H	9.209398	5.780730	7.177908
H	4.937616	6.425803	7.392185

H	6.030854	7.695164	6.817880
H	5.969301	7.301227	8.531829
H	5.858525	3.727470	12.215552
H	6.478847	5.214684	12.914866
H	7.601606	4.060717	12.191047
H	8.400983	6.237669	10.896304
H	7.095362	7.080193	11.728995
H	7.213467	7.181218	9.981198
H	4.723306	6.301286	9.819376
H	4.730808	6.335131	11.588127
H	4.294414	4.848761	10.725234

! **TS1** !

Spin multiplicity: 5

C	12.851251	0.540919	16.818585
C	13.126787	1.268092	18.002175
C	14.467139	1.716602	17.860691
C	15.046912	1.209523	16.653977
C	14.009417	0.454445	15.984733
Tm	13.170506	3.032333	16.030065
C	11.201917	4.742211	16.304010
C	11.830971	5.061110	15.079573
C	13.131142	5.602980	15.297902
C	13.358164	5.557701	16.733384
C	12.152267	5.023907	17.309747
C	13.918438	6.221765	14.119707
C	14.185355	7.721729	14.356391
C	14.392925	6.223825	17.665301
C	15.838839	6.222969	17.147971
C	9.745939	4.315271	16.454026
C	9.518502	2.932095	15.816391
C	12.364467	1.147937	19.320590
C	13.042395	0.014538	20.124817
C	16.545183	1.427121	16.353106
C	17.304334	0.090128	16.247641
C	14.012920	-0.579777	14.835687
C	14.887365	-0.243211	13.617664
O	12.889141	2.575134	13.910147
C	12.732358	2.394048	12.729980
C	13.947905	7.678952	17.935924
C	14.432096	5.511082	19.032055
C	13.084827	6.151913	12.824951
C	15.237801	5.487439	13.820626
C	8.856633	5.343097	15.726943
C	9.332808	4.286088	17.930491
C	10.898614	0.752315	19.097361
C	12.427544	2.429794	20.160209

C	16.764999	2.269938	15.084058
C	17.224400	2.189010	17.508873
C	12.585257	-0.817525	14.296843
C	14.478884	-1.929862	15.431839
C	10.115114	3.729612	11.949208
O	9.691861	3.465852	10.852714
Tm	8.969119	2.823748	8.873825
C	10.032170	0.889284	7.557781
C	10.477704	0.678088	8.884432
C	11.350731	1.748580	9.170536
C	11.512370	2.596618	8.037657
C	10.652963	2.056793	6.993482
C	10.239503	-0.524333	9.792258
C	9.555216	-0.095856	11.102610
C	12.591053	3.704561	8.056696
C	12.024908	5.118495	7.843830
C	10.587795	2.258744	5.464244
C	10.753302	3.706309	4.980687
C	6.803478	4.232611	9.699532
C	6.512266	2.835733	9.786409
C	6.424295	2.253765	8.498314
C	6.743628	3.298469	7.591352
C	6.954130	4.528333	8.289017
C	6.605816	5.115955	10.951470
C	7.604313	6.274818	11.100756
C	5.679619	0.966410	8.150398
C	4.220108	1.366772	7.835256
C	7.191040	5.839094	7.507059
C	8.609596	6.387468	7.735283
C	13.295690	3.739429	9.426634
C	13.696732	3.411168	7.022715
C	9.234187	1.777761	4.905269
C	11.679734	1.380451	4.811819
C	9.390606	-1.588972	9.086853
C	11.606044	-1.151967	10.133295
C	6.260091	0.264353	6.916713
C	5.655942	-0.011547	9.332724
C	6.154326	6.926522	7.845667
C	7.048672	5.594739	5.991108
C	5.163033	5.674318	10.926174
C	6.697571	4.278117	12.244979
H	15.015011	2.250000	18.625125
H	11.921968	0.024936	16.619591
H	13.460724	2.734759	20.353263
H	11.944765	2.272065	21.131258
H	11.919624	3.259040	19.662013
H	13.035712	-0.922911	19.559675
H	12.516486	-0.152912	21.072646

H	14.084153	0.261668	20.352233
H	10.366742	1.480452	18.479317
H	10.376862	0.684967	20.058264
H	10.815908	-0.225465	18.612066
H	16.776047	3.171877	17.682456
H	18.279246	2.351508	17.263550
H	17.184892	1.624030	18.445534
H	17.144522	-0.515230	17.145859
H	18.379977	0.281756	16.158141
H	17.008795	-0.504814	15.383963
H	16.278974	1.850074	14.202986
H	17.835207	2.367967	14.864788
H	16.378357	3.285656	15.228185
H	15.498163	-1.883358	15.819963
H	14.444231	-2.713126	14.664863
H	13.823755	-2.232486	16.255173
H	14.573227	0.684636	13.132389
H	14.794974	-1.045462	12.876987
H	15.946340	-0.161949	13.862662
H	11.924761	-1.255929	15.050586
H	12.632033	-1.530892	13.467566
H	12.122046	0.097430	13.920324
H	11.971370	4.942796	18.372131
H	11.369405	4.929656	14.109333
H	13.490310	5.600359	19.579606
H	15.209789	5.961062	19.658686
H	14.659539	4.446636	18.920975
H	16.195488	5.205050	16.956720
H	16.499060	6.663870	17.903478
H	15.967203	6.801560	16.233803
H	13.905758	8.275024	17.022380
H	14.642137	8.168852	18.629724
H	12.950760	7.693440	18.387354
H	15.912188	5.462626	14.677455
H	15.770166	5.980504	12.998315
H	15.039079	4.457749	13.503718
H	13.249363	8.250146	14.565091
H	14.627834	8.165193	13.456696
H	14.870898	7.914834	15.182164
H	12.844731	5.126216	12.535288
H	13.659672	6.598657	12.006537
H	12.146179	6.707156	12.914952
H	8.283696	3.985118	18.024338
H	9.439725	5.274209	18.390865
H	9.932179	3.578477	18.510597
H	10.122845	2.158832	16.310455
H	9.772651	2.938848	14.750955
H	8.469027	2.627293	15.909708

H	7.798875	5.078392	15.842641
H	9.080010	5.376118	14.657107
H	9.003882	6.347300	16.138157
H	11.841930	1.892427	10.123907
H	9.401078	0.210456	7.001810
H	14.113206	2.410690	7.179251
H	14.511860	4.135546	7.135990
H	13.349642	3.472118	5.990438
H	11.498489	5.233739	6.896662
H	12.835279	5.857010	7.861304
H	11.336262	5.377376	8.656348
H	12.602597	3.953344	10.244122
H	14.052623	4.531291	9.418332
H	13.802608	2.798195	9.655886
H	12.682109	1.661023	5.141518
H	11.643009	1.471277	3.719241
H	11.528654	0.327676	5.070655
H	9.991038	4.362075	5.414236
H	10.636693	3.744308	3.891603
H	11.732546	4.124767	5.211403
H	9.077358	0.705232	5.045579
H	9.193599	1.967929	3.827137
H	8.398422	2.306046	5.373513
H	12.136222	-1.450910	9.223067
H	11.469042	-2.043182	10.757591
H	12.241117	-0.449701	10.680610
H	10.152246	0.647201	11.641462
H	9.411007	-0.957729	11.765222
H	8.565429	0.340446	10.914156
H	8.406402	-1.206316	8.801805
H	9.232305	-2.446139	9.750272
H	9.885615	-1.953945	8.180638
H	6.292298	2.319093	10.710254
H	6.708289	3.202859	6.515301
H	6.663771	-0.289258	9.654072
H	5.129675	-0.930427	9.051723
H	5.132769	0.413571	10.195205
H	3.766995	1.887527	8.684851
H	3.615538	0.478498	7.615061
H	4.173894	2.034313	6.969009
H	6.287572	0.930382	6.048776
H	5.644986	-0.601757	6.647028
H	7.277355	-0.090074	7.100739
H	7.767915	4.860699	5.615737
H	7.231640	6.531991	5.455121
H	6.042124	5.253019	5.729635
H	8.821562	6.587940	8.786766
H	8.762453	7.320122	7.178627

H	9.357177	5.675771	7.368428
H	5.134874	6.547293	7.722507
H	6.280578	7.776998	7.165718
H	6.255594	7.309517	8.861071
H	5.886013	3.547522	12.315255
H	6.597180	4.945196	13.107391
H	7.648501	3.750462	12.343338
H	8.635016	5.912306	11.151092
H	7.400743	6.816275	12.031553
H	7.534642	7.002752	10.291214
H	4.974780	6.324856	10.072038
H	4.968836	6.250676	11.838858
H	4.439342	4.853841	10.884649

! **TS1**: Product (**iso2**) !

Spin multiplicity: 5

C	12.602026	0.856869	16.689395
C	13.078929	1.548094	17.827748
C	14.373715	2.000903	17.473698
C	14.739847	1.523907	16.174887
C	13.599913	0.787431	15.669849
Tm	12.800596	3.335919	15.794229
C	10.975471	5.043115	16.536831
C	11.413998	5.468846	15.262917
C	12.737383	5.990667	15.325298
C	13.172452	5.837977	16.701604
C	12.069467	5.249739	17.407105
C	13.358061	6.681583	14.088478
C	13.686020	8.159917	14.378537
C	14.335266	6.453712	17.508886
C	15.690408	6.480183	16.786354
C	9.564810	4.562405	16.861957
C	9.312740	3.181984	16.226855
C	12.550364	1.379692	19.251988
C	13.361293	0.224236	19.884663
C	16.174835	1.717154	15.641798
C	16.869955	0.360558	15.412955
C	13.377937	-0.183738	14.489416
C	14.086153	0.171617	13.173716
O	12.384078	3.036691	13.761992
C	11.918899	3.157618	12.569094
C	13.941805	7.891557	17.916697
C	14.569052	5.673978	18.817305
C	12.350448	6.705959	12.920565
C	14.604095	5.949499	13.557318
C	8.547793	5.565466	16.285577
C	9.348775	4.485769	18.378470

C	11.069977	0.975296	19.271222
C	12.749170	2.627478	20.120346
C	16.212359	2.565727	14.358278
C	17.046069	2.453456	16.678582
C	11.876758	-0.300220	14.148973
C	13.842155	-1.585624	14.951362
C	10.595267	2.748921	12.239014
O	10.100663	2.862453	11.055450
Tm	9.334784	2.504406	9.140349
C	10.085427	0.667754	7.427316
C	10.487358	0.196565	8.697629
C	11.513528	1.070477	9.123515
C	11.802726	2.044303	8.127648
C	10.878576	1.796103	7.032685
C	10.028258	-1.054279	9.442970
C	9.218148	-0.672854	10.695762
C	13.011341	2.992621	8.315323
C	12.595424	4.470206	8.429882
C	10.848246	2.249006	5.556144
C	11.130858	3.739599	5.318621
C	7.199828	3.860257	10.028662
C	6.777550	2.510683	9.835309
C	6.714349	2.185169	8.460417
C	7.198384	3.330804	7.782938
C	7.477510	4.388596	8.708196
C	7.039281	4.502862	11.422992
C	8.085251	5.571005	11.777298
C	5.830144	1.095987	7.852501
C	4.454343	1.753473	7.591732
C	7.842017	5.801697	8.202471
C	9.277722	6.201454	8.588463
C	13.748706	2.659022	9.627550
C	14.061528	2.813222	7.200839
C	9.468414	1.978277	4.926434
C	11.862401	1.396037	4.759796
C	9.181818	-1.951515	8.530732
C	11.265350	-1.857913	9.885147
C	6.360270	0.562408	6.516607
C	5.610849	-0.075785	8.818670
C	6.846398	6.864838	8.704257
C	7.763624	5.863882	6.663976
C	5.620371	5.113763	11.506885
C	7.118144	3.439121	12.538885
H	15.049007	2.509029	18.148116
H	11.646912	0.351761	16.637255
H	13.793791	2.952299	20.127376
H	12.463143	2.416018	21.156883
H	12.138747	3.463078	19.769811

H	13.261782	-0.691982	19.293985
H	13.005531	0.017165	20.901325
H	14.425506	0.473721	19.940339
H	10.432117	1.724586	18.795044
H	10.727463	0.855945	20.304930
H	10.907137	0.020074	18.761938
H	16.652920	3.444379	16.924848
H	18.051613	2.595327	16.268626
H	17.144264	1.883528	17.607974
H	16.848604	-0.241632	16.327221
H	17.919466	0.523073	15.140568
H	16.413993	-0.225101	14.615062
H	15.572421	2.174181	13.568429
H	17.234988	2.629874	13.967378
H	15.890312	3.592139	14.569218
H	14.907287	-1.611031	15.192311
H	13.655592	-2.324386	14.162539
H	13.292347	-1.898143	15.845196
H	13.753474	1.139402	12.791182
H	13.840831	-0.587316	12.422140
H	15.172994	0.184774	13.260760
H	11.290901	-0.720184	14.972117
H	11.757245	-0.984151	13.301852
H	11.443609	0.662784	13.861721
H	12.052797	5.077861	18.473194
H	10.821314	5.411525	14.360823
H	13.714625	5.728874	19.496805
H	15.427206	6.100225	19.347950
H	14.781255	4.619734	18.622390
H	16.005640	5.473660	16.492391
H	16.457902	6.880205	17.458563
H	15.690696	7.104189	15.893583
H	13.777744	8.537053	17.052036
H	14.730454	8.341762	18.531901
H	13.017858	7.881380	18.503549
H	15.396684	5.864397	14.302577
H	15.020931	6.487117	12.697095
H	14.333017	4.946179	13.212206
H	12.800637	8.686049	14.750775
H	14.003559	8.653374	13.452743
H	14.486790	8.294191	15.105913
H	12.086865	5.697611	12.584154
H	12.808372	7.222992	12.069959
H	11.436632	7.248883	13.183092
H	8.331313	4.145023	18.599132
H	9.484170	5.467287	18.844675
H	10.041072	3.788088	18.857875
H	10.007181	2.427802	16.623375

H	9.424186	3.211192	15.136721
H	8.300122	2.822427	16.446900
H	7.525516	5.256961	16.534287
H	8.621534	5.631315	15.196440
H	8.711720	6.567251	16.695569
H	12.023679	1.005186	10.074158
H	9.360755	0.183309	6.789570
H	14.369753	1.764883	7.127658
H	14.952658	3.405722	7.438104
H	13.718140	3.131008	6.216555
H	12.060353	4.830333	7.550198
H	13.480405	5.105170	8.558473
H	11.965788	4.618424	9.314847
H	13.115229	2.798113	10.511509
H	14.605224	3.334730	9.732593
H	14.136453	1.635242	9.621643
H	12.885856	1.529955	5.113145
H	11.832190	1.662548	3.696200
H	11.616749	0.333077	4.849995
H	10.415899	4.370163	5.857043
H	11.028675	3.969547	4.251878
H	12.133256	4.041884	5.616037
H	9.225628	0.913002	4.892031
H	9.462342	2.341316	3.892974
H	8.670796	2.490530	5.470009
H	11.880169	-2.136420	9.023115
H	10.958484	-2.777350	10.397729
H	11.892358	-1.285045	10.574292
H	9.802631	-0.047107	11.378845
H	8.901899	-1.565459	11.248782
H	8.304107	-0.125455	10.429563
H	8.280503	-1.442627	8.177864
H	8.863702	-2.849788	9.071109
H	9.753311	-2.270380	7.652730
H	6.448609	1.860092	10.634203
H	7.218658	3.438858	6.708055
H	6.547588	-0.574197	9.080108
H	4.953978	-0.822183	8.358490
H	5.131283	0.251396	9.746804
H	4.034777	2.160991	8.517002
H	3.747506	1.017482	7.189666
H	4.539956	2.573392	6.871883
H	6.522563	1.368376	5.794724
H	5.636803	-0.132404	6.075256
H	7.303635	0.026203	6.642688
H	8.453977	5.166958	6.180322
H	8.032479	6.871190	6.328702
H	6.752821	5.651367	6.301478

H	9.458962	6.151169	9.663050
H	9.498501	7.223855	8.259077
H	10.004746	5.547656	8.092953
H	5.819884	6.588117	8.443121
H	7.066689	7.827956	8.229110
H	6.892118	7.017812	9.782019
H	6.298080	2.716853	12.485529
H	7.028436	3.939964	13.509108
H	8.070681	2.902155	12.529820
H	9.094873	5.152087	11.753413
H	7.898119	5.936801	12.793354
H	8.052241	6.440589	11.120205
H	5.455936	5.904356	10.773055
H	5.453233	5.538245	12.504251
H	4.862977	4.341287	11.337672

! Complex **2** !

Spin multiplicity: 5

C	12.168875252	1.435419081	17.307542624
C	12.604590180	2.290926146	18.346453683
C	13.875365668	2.761605512	17.933599261
C	14.270982735	2.141727448	16.705704250
C	13.173020290	1.288003391	16.302537397
Tm	12.241335938	3.786117111	16.101139151
C	10.326718827	5.476754927	16.623673624
C	10.747431822	5.757878650	15.304439103
C	12.041531670	6.351818466	15.295636872
C	12.478569394	6.399883798	16.678931934
C	11.405019009	5.850186234	17.457169478
C	12.630163804	6.909846744	13.978653021
C	12.879398999	8.428275019	14.075710388
C	13.604494108	7.173997759	17.397271221
C	14.959234596	7.178333760	16.673515424
C	8.941796418	4.968678715	17.011748308
C	8.764591758	3.506923023	16.559725327
C	12.079945647	2.279230179	19.782006220
C	12.947569359	1.258441729	20.555428549
C	15.696014260	2.339705364	16.148202014
C	16.461864266	1.003150026	16.093363712
C	13.006655131	0.163172675	15.257098193
C	13.700523751	0.383329695	13.904696332
O	11.848978484	3.207316991	14.125270250
C	11.370444300	3.167891060	12.932177164
C	13.134947993	8.629738387	17.618326153
C	13.873508722	6.581837841	18.794260502
C	11.627243543	6.731521413	12.820097908
C	13.914774202	6.181787788	13.542321794
C	7.876097669	5.834994552	16.314287271

C	8.724103426	5.076030517	18.526159490
C	10.622611558	1.803849649	19.857122443
C	12.209964279	3.636768201	20.482523898
C	15.694248389	3.017410638	14.766311926
C	16.523566663	3.247429458	17.079493790
C	11.514946655	-0.074294535	14.938628368
C	13.541571086	-1.141686915	15.893795286
C	10.838463189	3.123245868	11.611953463
O	10.359299433	3.083778493	10.417302554
Tm	9.580612729	3.015974630	8.475285616
C	10.241066524	1.381067683	6.535667714
C	10.615698435	0.729604527	7.732542564
C	11.683885150	1.487977114	8.263554896
C	12.024351279	2.566700528	7.400906500
C	11.091300311	2.509459458	6.287021503
C	10.092074282	-0.582407407	8.311644592
C	9.299495711	-0.325977259	9.606756103
C	13.279062516	3.420382483	7.704560880
C	12.938336280	4.889810999	8.011242497
C	11.087332754	3.150732600	4.881686177
C	11.445696156	4.643319188	4.837923664
C	7.515129750	4.351567892	9.540855766
C	7.025410854	3.061498590	9.176231746
C	6.948916546	2.920412547	7.771053789
C	7.491954878	4.118187236	7.245513690
C	7.822246728	5.031754350	8.298718336
C	7.384197098	4.815462185	11.007324790
C	8.482168976	5.774448699	11.492420731
C	6.012011703	1.965257630	7.031063132
C	4.671912648	2.719703812	6.863545424
C	8.259081066	6.478196684	7.978627878
C	9.712325521	6.751476926	8.406930105
C	13.995576740	2.882781903	8.959362903
C	14.321336889	3.333962589	6.571711791
C	9.696985881	3.033967021	4.228182851
C	12.058746609	2.357903505	3.977245043
C	9.203310314	-1.309975904	7.294583151
C	11.285820321	-1.498062921	8.640719203
C	6.517441829	1.583072822	5.635058811
C	5.731389959	0.690822305	7.838335387
C	7.317505736	7.516369483	8.618118539
C	8.187443541	6.743001358	6.461483899
C	5.997898232	5.481316636	11.175720054
C	7.406485651	3.613539445	11.975759827
H	14.520572539	3.386631147	18.535348384
H	11.241629667	0.878586955	17.323323403
H	13.236178266	4.013941210	20.444887354
H	11.931293972	3.545367663	21.538423766

H	11.558153772	4.387452874	20.029385984
H	12.898319313	0.270084318	20.087472969
H	12.599108588	1.165194697	21.591250303
H	13.997088417	1.568268861	20.575646990
H	9.948398264	2.451505340	19.290531839
H	10.282736705	1.800355914	20.898556843
H	10.511834915	0.783996428	19.475070000
H	16.078285558	4.239849533	17.197682629
H	17.521939838	3.387883624	16.651903252
H	16.647716596	2.807347337	18.074051076
H	16.468374692	0.522930284	17.077395009
H	17.502527069	1.183993691	15.799432359
H	16.040217090	0.296959440	15.378493399
H	15.078717307	2.494881120	14.035028981
H	16.713663817	3.084227179	14.367544506
H	15.318258810	4.044161106	14.844713853
H	14.605623870	-1.080287368	16.133022965
H	13.396906815	-1.984265171	15.206808458
H	13.005314291	-1.365260632	16.821879132
H	13.319305264	1.275332470	13.402084258
H	13.498087247	-0.477517263	13.257401467
H	14.784847726	0.464313015	13.986282752
H	10.948573944	-0.415229166	15.810494046
H	11.434608411	-0.866545383	14.186602226
H	11.033271260	0.819946475	14.531405698
H	11.393155419	5.815850907	18.536542458
H	10.162091515	5.554422784	14.418822909
H	13.014720676	6.678726676	19.463495114
H	14.706163682	7.116885163	19.263418245
H	14.141194081	5.523839999	18.735630348
H	15.327706631	6.160274383	16.510244263
H	15.702166444	7.700831602	17.286685756
H	14.930467344	7.681816034	15.708061295
H	12.940825247	9.149522238	16.678453368
H	13.896611185	9.195728299	18.168464635
H	12.210494202	8.646664272	18.204223416
H	14.707792567	6.234519850	14.290113112
H	14.306364088	6.625654551	12.619062009
H	13.697773066	5.129704611	13.329558793
H	11.966315439	8.950824771	14.379847260
H	13.174389508	8.814806827	13.093366700
H	13.669210826	8.696401905	14.777595845
H	11.417955336	5.675966471	12.616588057
H	12.060901471	7.158382099	11.908901532
H	10.685320406	7.255142058	13.013356279
H	7.724948425	4.713713184	18.791572205
H	8.806269696	6.114966137	18.862192192
H	9.449973644	4.482744635	19.089175791

H	9.495876468	2.847160379	17.047785597
H	8.878640082	3.401831137	15.474546019
H	7.771258055	3.126149962	16.826926162
H	6.870341909	5.507953828	16.603392756
H	7.950576891	5.764348290	15.225501166
H	7.985920016	6.888281652	16.591891349
H	12.187904718	1.274399259	9.195535499
H	9.494217935	1.020572054	5.843702026
H	14.576179069	2.289697579	6.362095905
H	15.240818975	3.844914729	6.879841974
H	13.996743802	3.793507013	5.638343426
H	12.424215898	5.387430992	7.187901166
H	13.854077612	5.457200639	8.217138364
H	12.315012395	4.953812337	8.910655795
H	13.367956344	2.938069418	9.856613625
H	14.885011122	3.495008975	9.147306804
H	14.330930498	1.850102974	8.819290765
H	13.086868252	2.393001598	4.340540645
H	12.044507724	2.761106136	2.957243983
H	11.759312435	1.306008106	3.930128477
H	10.762403527	5.234179005	5.456528094
H	11.357731395	5.014361076	3.810378924
H	12.461451665	4.853457976	5.167645795
H	9.400580231	1.995772107	4.057202019
H	9.711680395	3.527636311	3.250456683
H	8.925130527	3.511177556	4.836936338
H	11.887688283	-1.693308675	7.747241856
H	10.931566502	-2.459439111	9.031272014
H	11.939496951	-1.051721371	9.395519635
H	9.913396976	0.175665242	10.362574936
H	8.937139367	-1.265544940	10.040920458
H	8.414992374	0.296803817	9.417610387
H	8.329761276	-0.714750306	7.014457972
H	8.838830776	-2.253424653	7.715485277
H	9.759898257	-1.540928354	6.380232301
H	6.662089688	2.330391488	9.885585387
H	7.520127644	4.363380829	6.193588520
H	6.641056584	0.116021237	8.029004378
H	5.038569495	0.044491791	7.288233770
H	5.266918080	0.918868457	8.803076028
H	4.271443154	3.024643461	7.835588179
H	3.929585959	2.078679254	6.372631004
H	4.800624644	3.620561468	6.255542101
H	6.722032302	2.466500803	5.022901771
H	5.760683814	0.988756281	5.110593297
H	7.432117832	0.988041637	5.686589811
H	8.842672691	6.080577066	5.888700923
H	8.507786971	7.770353042	6.258285285

H	7.167993446	6.630614818	6.078886332
H	9.888336695	6.553339242	9.465171695
H	9.985406740	7.795443570	8.211731223
H	10.406391939	6.131440884	7.827816130
H	6.278878861	7.328067076	8.327784363
H	7.587425609	8.520531971	8.270713821
H	7.368469831	7.525958426	9.706407565
H	6.550979999	2.946666888	11.832882389
H	7.340078446	3.988394987	13.003036475
H	8.330590839	3.034794380	11.893145386
H	9.469304515	5.311637745	11.410147926
H	8.311511950	6.014559461	12.548080664
H	8.494786095	6.722359689	10.953586092
H	5.875429428	6.367588520	10.551135256
H	5.850227435	5.780952961	12.220358950
H	5.202699892	4.776559914	10.911191333

! **TS2**: Adduct !

Spin multiplicity: 5

C	17.193051061	3.793991448	5.363055117
C	16.570449966	3.716526995	4.096758439
C	16.177747131	2.379580221	3.796747093
C	16.625816455	1.563355996	4.912035852
C	17.262436182	2.462851312	5.830698251
Tm	18.857987841	2.530436005	3.743048389
C	21.117602744	1.742291254	2.740424920
C	21.547058084	2.621593991	3.778551494
C	21.173706986	1.986803132	5.030635295
C	20.553924072	0.742820029	4.676420159
C	20.555410880	0.558822444	3.276702678
C	22.421589151	3.851161923	3.436338531
C	23.852866226	3.650672790	3.977072914
C	21.640935686	2.171903622	6.492914482
C	20.639070143	1.545026281	7.482982951
C	20.295857122	-0.725164269	2.492331421
C	21.618770293	-1.133386771	1.810940291
C	15.263972658	2.105684757	2.580268269
C	15.813573437	1.052786555	1.603115542
C	16.220949588	0.165325887	5.433963538
C	17.297273137	-0.415945626	6.371501034
C	17.447930705	5.086037713	6.134671212
C	16.098715532	5.822963390	6.274260124
O	18.957508043	4.053011427	2.227899175
C	19.055463585	5.044000803	1.466443438
O	19.033953508	5.983159990	-0.735848881
C	18.913560244	4.966986494	0.046875746
Tm	18.730076612	5.046613648	-2.789967102
C	16.298307259	4.507738836	-3.708924419

C	16.115963673	5.000464329	-2.395614306
C	16.368779293	6.403330686	-2.333530878
C	16.786651585	6.800975323	-3.662364506
C	16.758370156	5.609639983	-4.463472821
C	15.967379343	7.204373840	-1.071862003
C	17.086566010	8.075077781	-0.471459873
C	16.830733312	8.162972457	-4.396970483
C	17.799473554	8.122722680	-5.592133553
C	15.757003041	3.190939652	-4.257155873
C	16.631341208	2.635593385	-5.385886742
C	21.204186701	5.958002394	-2.895019985
C	20.753501462	5.941111352	-4.235708864
C	20.669642783	4.596565770	-4.729039237
C	21.021906778	3.739139550	-3.616506237
C	21.314590223	4.603523267	-2.517649695
C	21.734244445	7.169697677	-2.134186041
C	21.997558647	6.842344128	-0.657953259
C	20.658516250	4.388207992	-6.263182158
C	20.158715963	3.033221660	-6.782854917
C	21.314296652	2.226628942	-3.509973103
C	20.105940701	1.333763106	-3.843564931
C	14.725829208	8.067511445	-1.384007886
C	15.530499935	6.250419659	0.058745740
C	17.250278629	9.385736886	-3.569600109
C	15.416257174	8.420307500	-4.969013113
C	15.624901735	2.123561537	-3.162659232
C	14.350057149	3.482905207	-4.825096016
C	19.781850513	5.442054628	-6.963643744
C	22.107621101	4.601408089	-6.762763258
C	21.733013192	1.856983447	-2.072985988
C	22.524998914	1.853591407	-4.392094403
C	20.768749670	8.359702350	-2.216441764
C	23.071719330	7.575534852	-2.791088862
C	22.970572057	1.399160962	6.659037381
C	21.847893395	3.620281213	6.955712762
C	19.224405119	-0.518795286	1.412679164
C	19.871175831	-1.868387713	3.422509477
C	21.850507315	5.192133537	3.930604621
C	22.577061180	3.993435681	1.908832952
C	13.853344391	1.709148125	3.062365210
C	15.079840666	3.390999080	1.749328769
C	15.959466488	-0.908190135	4.368810807
C	14.937856751	0.343795782	6.279897545
C	18.433628243	6.002151328	5.393851931
C	17.975473504	4.797069783	7.545112137
C	18.426789275	2.878141253	-1.572283523
O	18.278646614	1.820486183	-0.980777116
H	20.393566337	0.277080085	-3.780109955

H	13.830784862	0.744371870	3.571029188
H	16.015121744	0.093612115	2.077031819
H	15.084394386	0.874257612	0.804510764
H	16.729743320	1.408819341	1.121200721
H	14.613522354	-0.621189729	6.687913348
H	16.967603828	-1.393121494	6.740498769
H	18.247451899	-0.558686651	5.851507521
H	16.830474792	-1.062906080	3.725117718
H	15.745198836	-1.862093247	4.863394235
H	15.103043561	-0.680070591	3.734580531
H	21.491354733	-2.078736744	1.270637742
H	21.948382762	-0.377066465	1.092769669
H	19.464593871	0.314147452	0.745744193
H	19.113127204	-1.419749367	0.798287402
H	18.247359823	-0.314348497	1.862792981
H	18.942229211	-1.641282660	3.953806731
H	19.703092243	-2.780990863	2.840706194
H	20.641203546	6.822022932	-4.854114289
H	20.950501157	2.094417595	-1.348271424
H	19.716056108	1.504870885	-4.845621244
H	19.299474312	1.498709234	-3.122835496
H	22.153375907	4.496507203	-7.853705962
H	19.121702209	2.849653055	-6.493682692
H	20.195783006	3.036682758	-7.878247527
H	20.763398544	2.191444782	-6.448495382
H	20.116822776	6.465430150	-6.781404699
H	19.815267991	5.282840992	-8.047185037
H	18.740153560	5.361936291	-6.643478999
H	22.746184282	6.049708464	-0.553529865
H	22.387066922	7.729065735	-0.145035786
H	21.083199115	6.525313249	-0.149269056
H	19.825473254	8.139841513	-1.706112311
H	15.766291023	4.403725587	-1.565681621
H	16.929080577	5.594741702	-5.531702136
H	17.617958383	2.352146946	-5.009082909
H	16.172620304	1.740136615	-5.820710183
H	16.763276143	3.362658858	-6.193787789
H	14.931599036	2.442376801	-2.377819419
H	15.225609880	1.198153423	-3.593420793
H	16.584295285	1.894411286	-2.691000385
H	14.396225343	4.212733448	-5.639867372
H	13.893798084	2.564527860	-5.214509321
H	13.693530957	3.888617457	-4.048675564
H	17.530602846	7.366913133	-6.332675074
H	17.787558128	9.091008818	-6.104777589
H	18.823741868	7.928782675	-5.264304709
H	18.251052170	9.262745784	-3.147860465
H	17.276198723	10.265369289	-4.223020783

H	16.559388827	9.614285721	-2.758842484
H	14.661758698	8.471369057	-4.180482187
H	15.395118308	9.367361538	-5.522124549
H	15.124179752	7.618904599	-5.654765127
H	17.507155220	8.782323043	-1.183418864
H	17.895452553	7.444630424	-0.095338529
H	13.920080703	7.449523495	-1.794577539
H	14.356944326	8.535999704	-0.463683796
H	14.928132223	8.867698079	-2.097211521
H	17.622575482	2.173077131	6.807219134
H	16.382426699	4.567415896	3.457823264
H	13.453386109	2.461668796	3.750093089
H	13.174633075	1.641408305	2.204347337
H	16.032368264	3.767846257	1.366147333
H	14.443662915	3.166897200	0.886933573
H	14.588296894	4.185753624	2.318806827
H	15.358545291	5.193656970	6.779007109
H	16.226263110	6.739604920	6.861850077
H	15.692029884	6.103372580	5.298112690
H	18.105404781	6.211224958	4.370805272
H	18.536784206	6.961124647	5.914550741
H	19.430658088	5.553640940	5.338687539
H	18.923319745	4.251674177	7.525311421
H	18.146380681	5.736235752	8.082117498
H	17.258398200	4.207598765	8.126062413
H	14.115163754	0.756682832	5.691805877
H	15.119332821	1.022777719	7.119090217
H	17.479762513	0.209299800	7.248721430
H	20.246624550	-0.005532370	5.392597865
H	21.668726720	5.207430113	5.004377712
H	20.915837760	5.430606799	3.414073920
H	20.513095683	0.469673066	7.334670557
H	21.005037731	1.684126730	8.505881901
H	19.655542251	2.015701653	7.413337033
H	20.938445575	4.215739765	6.833903736
H	16.689427860	8.654764096	0.370851596
H	16.331733948	5.565143979	0.347057410
H	15.270754855	6.842671203	0.942902223
H	14.646542148	5.665154711	-0.213417777
H	21.915727852	0.777835983	-2.024076349
H	22.797752545	0.804370312	-4.226823377
H	22.416056149	-1.266226888	2.549487674
H	20.643515520	-2.086359098	4.167629478
H	21.617961589	4.267097630	-1.536835189
H	22.657240552	2.361005003	-1.774162668
H	23.391900963	2.471364798	-4.134173657
H	22.335292794	1.976096498	-5.458546223
H	22.801754642	3.880497383	-6.324272035

H	22.463024203	5.603203222	-6.502066372
H	21.205073076	9.242094362	-1.734017042
H	20.549357950	8.627751478	-3.255296428
H	22.929831365	7.858400294	-3.839177477
H	23.514721516	8.429544117	-2.264419589
H	23.786823877	6.747106101	-2.761299197
H	21.250897837	1.930459322	1.683944760
H	24.269873838	2.700889555	3.626039507
H	24.499527888	4.456997193	3.612383311
H	23.907567756	3.659171809	5.066245280
H	22.556797302	6.000279257	3.707289255
H	21.614997923	4.125834834	1.407560686
H	23.181354349	4.881094516	1.694937841
H	23.088553265	3.132782393	1.467207261
H	23.751134452	1.778384571	5.996424848
H	23.331753692	1.481496486	7.691309376
H	22.829748384	0.337576163	6.432643312
H	22.105104435	3.628457808	8.020685385
H	22.656918988	4.125720011	6.429199025

! **TS2** !

Spin multiplicity: 5

C	17.085857	3.459973	4.775816
C	16.483012	3.219980	3.521542
C	16.113596	1.850982	3.382846
C	16.542248	1.186789	4.604264
C	17.151391	2.198426	5.413475
Tm	18.775551	1.997021	3.346802
C	21.036116	1.127173	2.386592
C	21.454493	2.138887	3.297852
C	21.120605	1.653164	4.626872
C	20.528820	0.361838	4.442145
C	20.513452	0.006309	3.074280
C	22.276964	3.344826	2.787373
C	23.715222	3.286658	3.341929
C	21.578209	2.045957	6.049600
C	20.598971	1.510456	7.113007
C	20.328616	-1.384753	2.471230
C	21.700137	-1.830424	1.917740
C	15.268454	1.396346	2.172391
C	15.941203	0.287581	1.346536
C	16.163340	-0.153208	5.272638
C	17.200656	-0.549706	6.342767
C	17.316543	4.839955	5.385636
C	15.963589	5.582721	5.391430
O	18.748560	3.059867	1.455767
C	18.854483	4.138698	0.804395

O	18.906794	5.519111	-1.126822
C	18.763556	4.382407	-0.575892
Tm	18.572021	4.524065	-3.122221
C	16.078638	3.935217	-3.872995
C	15.949092	4.585103	-2.618897
C	16.216466	5.983416	-2.737550
C	16.603372	6.208557	-4.112132
C	16.541161	4.926998	-4.761998
C	15.836691	6.942154	-1.583095
C	16.938036	7.922142	-1.136762
C	16.644090	7.462888	-5.018328
C	17.611396	7.265140	-6.200198
C	15.505846	2.579650	-4.275906
C	16.462801	1.813177	-5.196658
C	21.058753	5.350582	-3.445542
C	20.547340	5.148327	-4.748971
C	20.448900	3.749112	-5.051875
C	20.849977	3.051380	-3.851854
C	21.179500	4.062062	-2.888580
C	21.623161	6.657289	-2.896541
C	21.993006	6.534617	-1.412492
C	20.364793	3.351912	-6.546292
C	19.931208	1.916024	-6.870912
C	21.228836	1.577598	-3.575886
C	20.107940	0.550049	-3.818331
C	14.564832	7.723972	-1.978591
C	15.455792	6.143980	-0.319236
C	17.061896	8.786570	-4.364691
C	15.228029	7.641881	-5.616653
C	15.172044	1.706992	-3.059713
C	14.192858	2.848474	-5.044833
C	19.382058	4.259205	-7.308810
C	21.766992	3.583750	-7.158585
C	21.662456	1.393126	-2.107355
C	22.469310	1.205787	-4.418920
C	20.639459	7.822150	-3.074680
C	22.908927	6.973561	-3.691664
C	22.940821	1.362203	6.309384
C	21.720429	3.549863	6.320279
C	19.315912	-1.388809	1.317217
C	19.899033	-2.400372	3.537268
C	21.641704	4.711007	3.098559
C	22.420941	3.282962	1.254243
C	13.862412	0.954073	2.625191
C	15.047550	2.571720	1.200453
C	16.034512	-1.359417	4.332999
C	14.818388	0.052380	6.007916
C	18.322749	5.657085	4.561884

C	17.804521	4.732297	6.835824
C	18.321561	2.650610	-1.750263
O	18.184390	1.715161	-1.058333
H	20.504953	-0.463805	-3.683231
H	13.870744	0.046879	3.230543
H	16.161518	-0.602740	1.934003
H	15.286794	-0.018508	0.522114
H	16.867290	0.652578	0.890090
H	14.529552	-0.865268	6.534367
H	16.925999	-1.517823	6.774612
H	18.203580	-0.643812	5.918497
H	16.959366	-1.535642	3.775797
H	15.830637	-2.260019	4.922628
H	15.221080	-1.258109	3.614994
H	21.635899	-2.850619	1.521563
H	22.036085	-1.175104	1.108726
H	19.569092	-0.652323	0.548242
H	19.288145	-2.372968	0.835623
H	18.303276	-1.173139	1.670065
H	18.955010	-2.122067	4.014464
H	19.761510	-3.386839	3.081919
H	20.412375	5.934758	-5.480162
H	20.876831	1.689316	-1.407756
H	19.686381	0.605048	-4.818913
H	19.294722	0.680894	-3.101958
H	21.766472	3.325888	-8.224873
H	18.940947	1.691324	-6.467655
H	19.875230	1.799375	-7.959484
H	20.632944	1.164797	-6.509309
H	19.641586	5.317621	-7.244452
H	19.383801	3.991086	-8.371301
H	18.363362	4.134976	-6.932949
H	22.735908	5.745317	-1.254588
H	22.433979	7.474436	-1.060468
H	21.114843	6.317328	-0.799881
H	19.728713	7.658532	-2.491252
H	15.605223	4.101523	-1.715427
H	16.672863	4.782128	-5.826021
H	17.404906	1.596899	-4.683686
H	16.020384	0.860358	-5.510611
H	16.687766	2.385459	-6.102479
H	14.448786	2.202081	-2.403361
H	14.721865	0.764873	-3.392712
H	16.060791	1.465208	-2.473078
H	14.377524	3.437138	-5.948902
H	13.719609	1.904682	-5.342681
H	13.483937	3.404680	-4.422830
H	17.359318	6.401380	-6.818545

H	17.578996	8.146211	-6.851053
H	18.640551	7.145260	-5.850752
H	18.055582	8.718256	-3.915054
H	17.101861	9.565833	-5.134641
H	16.359687	9.129423	-3.604772
H	14.473902	7.784746	-4.838825
H	15.201642	8.515142	-6.280171
H	14.938514	6.762469	-6.200214
H	17.311660	8.544859	-1.946035
H	17.782753	7.379370	-0.708529
H	13.762641	7.036039	-2.266864
H	14.211210	8.319476	-1.128155
H	14.730968	8.409415	-2.811036
H	17.496513	2.037612	6.424598
H	16.300998	3.981847	2.776809
H	13.377813	1.743904	3.208653
H	13.238248	0.754207	1.746763
H	15.991671	2.962676	0.811826
H	14.462332	2.217092	0.346113
H	14.489929	3.390951	1.664500
H	15.209338	5.018789	5.949607
H	16.072650	6.567023	5.861638
H	15.586540	5.735330	4.375876
H	18.028533	5.726816	3.509335
H	18.408536	6.677253	4.953259
H	19.321482	5.210974	4.601296
H	18.746244	4.180982	6.913785
H	17.971161	5.731744	7.251355
H	17.065677	4.228318	7.467703
H	14.011508	0.315389	5.320698
H	14.901523	0.856066	6.746272
H	17.247870	0.163553	7.169782
H	20.253532	-0.301910	5.249497
H	21.484026	4.874421	4.164089
H	20.683727	4.821805	2.578124
H	20.547849	0.419010	7.130473
H	20.931553	1.828438	8.106772
H	19.587958	1.895192	6.957818
H	20.781130	4.083468	6.147908
H	16.536280	8.592349	-0.366591
H	16.278976	5.512687	0.027397
H	15.215254	6.847644	0.485461
H	14.573399	5.516276	-0.478029
H	21.882318	0.334180	-1.931882
H	22.829075	0.209021	-4.136212
H	22.463706	-1.815661	2.702123
H	20.656468	-2.500219	4.321811
H	21.531046	3.860345	-1.886757

H	22.569081	1.959361	-1.873958
H	23.281250	1.919881	-4.244896
H	22.265616	1.186672	-5.490601
H	22.530183	2.978075	-6.663550
H	22.064473	4.632411	-7.062190
H	21.089967	8.762166	-2.734830
H	20.362199	7.953735	-4.125903
H	22.697131	7.094101	-4.759021
H	23.366748	7.901783	-3.328490
H	23.641095	6.166341	-3.585554
H	21.147752	1.186125	1.313056
H	24.176759	2.317989	3.123249
H	24.323948	4.063936	2.866075
H	23.768143	3.449353	4.419056
H	22.293461	5.515338	2.738519
H	21.451539	3.316748	0.750415
H	22.995270	4.151301	0.916393
H	22.956821	2.385977	0.928856
H	23.706193	1.693422	5.604516
H	23.292202	1.588527	7.323222
H	22.850967	0.275409	6.215696
H	21.995016	3.704109	7.369652
H	22.494728	4.021463	5.716309

! **TS2**: Product !

Spin multiplicity: 5

C	17.082931	3.357062	5.022308
C	16.472929	3.262274	3.751863
C	16.086868	1.921771	3.464732
C	16.519420	1.121955	4.598854
C	17.144182	2.031417	5.513225
Tm	18.764254	2.055393	3.448497
C	21.015628	1.284948	2.383315
C	21.448550	2.212888	3.374744
C	21.128069	1.614690	4.660524
C	20.533958	0.343240	4.371221
C	20.496653	0.110423	2.977878
C	22.278318	3.452551	2.968234
C	23.714988	3.335328	3.518251
C	21.599204	1.886446	6.107231
C	20.631565	1.258513	7.130735
C	20.263623	-1.212599	2.253363
C	21.596490	-1.622008	1.591511
C	15.211567	1.621319	2.226340
C	15.839009	0.604338	1.257543
C	16.138167	-0.283094	5.115346
C	17.195090	-0.810012	6.107839

C	17.325267	4.660231	5.777741
C	15.962831	5.368907	5.937253
O	18.806287	3.398651	1.753737
C	18.903421	4.407893	1.015008
O	18.928281	5.435742	-1.142932
C	18.798108	4.383513	-0.407434
Tm	18.607034	4.588633	-3.229508
C	16.122516	3.846830	-3.847635
C	16.008676	4.598155	-2.654852
C	16.244853	5.984868	-2.896695
C	16.583891	6.102031	-4.301095
C	16.523934	4.770081	-4.838223
C	15.906372	7.027435	-1.804288
C	17.048459	7.997994	-1.450505
C	16.579414	7.283697	-5.300886
C	17.474228	6.983035	-6.516791
C	15.578898	2.441748	-4.087743
C	16.424085	1.670780	-5.107200
C	21.083630	5.452325	-3.566398
C	20.585409	5.177467	-4.860960
C	20.487855	3.761941	-5.082839
C	20.886509	3.135298	-3.840412
C	21.208812	4.196467	-2.937997
C	21.636410	6.788152	-3.079901
C	21.944844	6.759811	-1.576757
C	20.408802	3.259666	-6.544417
C	19.866309	1.840957	-6.762685
C	21.201472	1.671687	-3.454547
C	19.992209	0.723293	-3.543695
C	14.645153	7.814971	-2.219937
C	15.535854	6.323062	-0.483471
C	17.054404	8.643131	-4.769343
C	15.133939	7.439034	-5.828280
C	15.502084	1.627527	-2.789101
C	14.149802	2.596322	-4.652661
C	19.514709	4.173639	-7.401965
C	21.835752	3.341376	-7.137175
C	21.701193	1.590526	-1.997384
C	22.368712	1.136289	-4.311568
C	20.667352	7.943596	-3.368449
C	22.952571	7.048509	-3.844388
C	22.965859	1.187809	6.295288
C	21.739337	3.363381	6.498980
C	19.191759	-1.078400	1.162563
C	19.864292	-2.321046	3.235952
C	21.654882	4.794012	3.394840
C	22.431948	3.518655	1.435184
C	13.807382	1.154482	2.659983

C	14.989917	2.907462	1.405942
C	15.967643	-1.370346	4.046052
C	14.814857	-0.148956	5.904426
C	18.274608	5.589131	5.005786
C	17.885977	4.398930	7.180789
C	18.520676	3.113127	-1.239045
O	18.361330	2.025074	-0.717831
H	20.307048	-0.302435	-3.314723
H	13.813190	0.183966	3.157552
H	16.039399	-0.359611	1.723779
H	15.158495	0.424077	0.417704
H	16.768859	0.992592	0.827842
H	14.523480	-1.119477	6.323870
H	16.919512	-1.819363	6.431690
H	18.187695	-0.862538	5.652589
H	16.872120	-1.492509	3.443288
H	15.766685	-2.330447	4.534066
H	15.134180	-1.175248	3.372050
H	21.491136	-2.596019	1.099336
H	21.907085	-0.894073	0.836509
H	19.417938	-0.270898	0.460200
H	19.105821	-2.008087	0.588341
H	18.207716	-0.875582	1.596908
H	18.944305	-2.080515	3.776638
H	19.694235	-3.257701	2.694359
H	20.450884	5.922491	-5.634587
H	20.956438	1.962047	-1.289679
H	19.537992	0.710867	-4.533047
H	19.229350	1.003005	-2.811595
H	21.830512	3.019634	-8.186034
H	18.843397	1.741220	-6.392717
H	19.847396	1.626762	-7.837457
H	20.476534	1.068274	-6.296998
H	19.868106	5.206423	-7.436147
H	19.502905	3.806137	-8.434284
H	18.484878	4.177523	-7.035660
H	22.697596	5.999880	-1.341057
H	22.348591	7.728679	-1.261211
H	21.046409	6.552217	-0.989335
H	19.742252	7.836536	-2.793041
H	15.717432	4.182813	-1.700984
H	16.631864	4.535052	-5.889357
H	17.434098	1.498124	-4.725416
H	15.976334	0.692629	-5.317438
H	16.500449	2.208129	-6.058435
H	14.834578	2.102278	-2.062466
H	15.099101	0.630223	-2.999795
H	16.482253	1.506999	-2.319525

H	14.156769	3.141835	-5.601903
H	13.696769	1.612853	-4.827537
H	13.511664	3.147468	-3.954257
H	17.157144	6.095543	-7.068643
H	17.436729	7.824674	-7.217128
H	18.515549	6.841826	-6.215501
H	18.079364	8.592065	-4.392945
H	17.042054	9.370359	-5.589327
H	16.416705	9.044645	-3.982132
H	14.429586	7.665499	-5.024236
H	15.081384	8.251242	-6.563856
H	14.797557	6.517459	-6.313500
H	17.423650	8.549615	-2.310016
H	17.882209	7.454115	-1.000394
H	13.822193	7.130492	-2.452467
H	14.322741	8.464150	-1.396878
H	14.805795	8.450469	-3.091555
H	17.497617	1.752790	6.495791
H	16.290720	4.104485	3.099746
H	13.349560	1.879971	3.340804
H	13.161058	1.064653	1.779572
H	15.929348	3.325381	1.034473
H	14.370354	2.669786	0.535261
H	14.464722	3.676536	1.981232
H	15.249314	4.729779	6.467546
H	16.081700	6.296121	6.509939
H	15.527780	5.625456	4.966864
H	17.910237	5.795116	3.994253
H	18.380923	6.549861	5.522571
H	19.276262	5.155709	4.916921
H	18.844234	3.872706	7.150352
H	18.047118	5.347456	7.704011
H	17.192082	3.801775	7.781714
H	13.996288	0.207178	5.275531
H	14.931024	0.557905	6.731978
H	17.267250	-0.197286	7.010107
H	20.265151	-0.386276	5.122185
H	21.477950	4.858545	4.468138
H	20.709215	4.969664	2.871825
H	20.574713	0.170328	7.047715
H	20.978606	1.482021	8.145175
H	19.620434	1.660554	7.025959
H	20.800501	3.908061	6.362274
H	16.690482	8.733902	-0.719879
H	16.359369	5.713525	-0.102258
H	15.311019	7.082133	0.273705
H	14.648360	5.691090	-0.587929
H	21.898695	0.541664	-1.749900

H	22.669625	0.144530	-3.952973
H	22.396202	-1.699752	2.335185
H	20.652323	-2.503435	3.974306
H	21.547364	4.053696	-1.922200
H	22.634310	2.144084	-1.853874
H	23.237905	1.798791	-4.236239
H	22.117297	1.036186	-5.367762
H	22.542570	2.710748	-6.593409
H	22.211742	4.368597	-7.098821
H	21.119229	8.902825	-3.090393
H	20.411565	7.997636	-4.432112
H	22.778921	7.116648	-4.923221
H	23.409509	7.988687	-3.512492
H	23.669719	6.239659	-3.670577
H	21.109305	1.438399	1.317255
H	24.168205	2.384644	3.218541
H	24.330804	4.145187	3.110919
H	23.768387	3.404412	4.605506
H	22.326109	5.617879	3.125761
H	21.467453	3.588645	0.926094
H	23.004329	4.414436	1.173726
H	22.974947	2.653744	1.041470
H	23.724254	1.580247	5.614484
H	23.325769	1.327757	7.321679
H	22.877463	0.112722	6.110079
H	22.007308	3.435360	7.558882
H	22.515731	3.881661	5.937379

! **TS3** Adduct !

Spin multiplicity: 5

C	7.658635	5.141983	9.230007
C	6.913389	4.045590	9.763848
C	6.334369	3.271266	8.729587
C	6.803247	3.848277	7.524223
C	7.588745	5.013780	7.787832
Tm	8.990599	2.880090	8.668178
C	9.484794	0.338053	8.920179
C	10.743001	0.960338	8.769857
C	10.924195	1.439558	7.440890
C	9.695472	1.135907	6.727443
C	8.836609	0.479953	7.672015
C	12.303129	1.988965	7.004303
C	13.296295	1.954664	8.183028
C	9.346838	1.048228	5.225872
C	7.820458	1.034108	5.012660
C	9.012297	-0.392748	10.172613
C	7.740658	-1.201504	9.888021

C	5.103792	2.375813	8.866390
C	5.054891	1.266115	7.808559
C	8.037014	5.934667	6.630783
C	7.484842	7.364173	6.792028
C	8.105401	6.275031	10.179052
C	6.959632	7.311573	10.261259
O	10.402352	3.615814	10.111159
C	11.435386	4.007871	10.708523
C	12.004385	3.804508	11.944706
O	11.589063	3.008684	12.940013
Tm	12.589142	3.260088	14.883239
C	10.879498	5.261587	15.049236
C	12.003007	5.862814	15.691175
C	12.191184	5.143536	16.937021
C	11.159154	4.152521	16.983258
C	10.313244	4.245570	15.858065
C	12.902406	5.490140	18.267034
C	11.949681	6.391864	19.088706
C	12.584448	7.190045	15.144536
C	11.950069	7.538308	13.781659
C	8.912890	3.649157	15.701204
C	7.916831	4.822485	15.584182
C	12.964978	0.855903	16.030916
C	14.053199	1.653010	16.465483
C	14.955288	1.915662	15.391613
C	14.380994	1.289201	14.217231
C	13.161665	0.684422	14.641852
C	12.095640	-0.041215	16.909202
C	10.804981	-0.462270	16.194185
C	16.321122	2.587166	15.658935
C	16.477016	3.945647	14.951598
C	14.940613	0.911631	12.828501
C	13.791678	0.567065	11.857433
C	11.746273	0.605356	18.254551
C	12.921230	-1.316816	17.196644
C	16.504392	2.854736	17.165645
C	17.490064	1.658907	15.271900
C	15.773005	-0.383775	12.984552
C	15.798468	1.976399	12.128264
C	13.143080	4.229185	19.120849
C	14.261714	6.193003	18.150735
C	14.108794	7.204878	14.922771
C	12.194526	8.350922	16.085300
C	8.519111	2.830561	16.938688
C	8.775480	2.765969	14.452272
C	12.934697	1.106028	5.909329
C	12.249009	3.454275	6.539819
C	9.877382	-0.302007	4.690132

C	9.892071	2.187210	4.352890
C	10.109443	-1.373110	10.628028
C	8.750424	0.617661	11.304746
C	4.991162	1.756014	10.265486
C	3.875140	3.291121	8.654440
C	7.470635	5.427649	5.289461
C	9.566803	5.978277	6.476922
C	8.302566	5.743990	11.613830
C	9.413944	6.983633	9.796381
H	14.237461	1.918870	17.495508
H	12.513347	0.107738	13.996520
H	12.644259	0.899174	18.806620
H	11.191768	-0.102156	18.881629
H	11.126794	1.494291	18.120841
H	13.197140	-1.822058	16.265776
H	12.345899	-2.019410	17.812287
H	13.844566	-1.071393	17.731108
H	10.187788	0.401796	15.933261
H	10.208926	-1.117870	16.839157
H	11.016645	-1.014487	15.273063
H	15.739794	3.526589	17.563531
H	17.475496	3.333237	17.332132
H	16.486046	1.928489	17.749036
H	17.390530	0.684333	15.761248
H	18.437227	2.104386	15.598409
H	17.566247	1.491959	14.197693
H	16.331955	3.884400	13.873698
H	17.475944	4.357325	15.140876
H	15.747281	4.667379	15.333101
H	16.649984	-0.244786	13.618732
H	16.118106	-0.730979	12.002439
H	15.163695	-1.176654	13.430197
H	15.231154	2.888859	11.934377
H	16.141798	1.585504	11.163403
H	16.687942	2.249044	12.695861
H	13.284027	-0.359291	12.142997
H	14.204385	0.406996	10.855918
H	13.046321	1.364543	11.811552
H	10.975604	3.517516	17.836828
H	10.478304	5.572628	14.094477
H	12.215812	3.721068	19.395515
H	13.639904	4.511758	20.055652
H	13.781736	3.510390	18.604851
H	14.964475	5.620185	17.538700
H	14.703198	6.297586	19.148356
H	14.184584	7.196584	17.731663
H	11.713078	7.322445	18.569370
H	12.404046	6.647546	20.054188

H	11.005942	5.873179	19.285111
H	14.671598	6.943465	15.816936
H	14.423581	8.211082	14.619856
H	14.394377	6.511449	14.130682
H	11.116627	8.350768	16.278980
H	12.455443	9.309258	15.620213
H	12.709654	8.307589	17.046000
H	12.111247	6.758249	13.032537
H	12.405604	8.458717	13.400322
H	10.873347	7.716778	13.868128
H	7.502601	2.439068	16.819681
H	8.537386	3.443380	17.845956
H	9.183377	1.975908	17.093556
H	9.400127	1.869800	14.524161
H	9.087603	3.294980	13.549792
H	7.735263	2.439148	14.330184
H	6.888245	4.446098	15.521243
H	8.111503	5.424088	14.691951
H	7.986400	5.482684	16.454906
H	11.478310	1.044736	9.557042
H	7.872947	0.059894	7.423461
H	12.979895	0.061722	6.235023
H	13.959781	1.440208	5.712409
H	12.396593	1.140153	4.961798
H	11.581311	3.607231	5.692021
H	13.247017	3.792672	6.237849
H	11.927433	4.106602	7.360229
H	12.972569	2.571727	9.026700
H	14.261594	2.347186	7.846627
H	13.463314	0.935134	8.542719
H	10.962113	-0.385005	4.777222
H	9.611656	-0.422357	3.632991
H	9.434878	-1.133565	5.247598
H	9.537334	3.163621	4.697325
H	9.543597	2.057788	3.322301
H	10.980657	2.216846	4.319308
H	7.343329	0.153388	5.450356
H	7.601350	1.014193	3.939822
H	7.346307	1.922357	5.437137
H	10.333746	-2.101423	9.842017
H	9.780986	-1.921743	11.518292
H	11.036723	-0.852040	10.881176
H	9.653839	1.172781	11.577355
H	8.386806	0.114906	12.208350
H	7.973281	1.343850	11.022101
H	6.909562	-0.566350	9.568777
H	7.420772	-1.731628	10.791422
H	7.915252	-1.946857	9.104982

H	6.735088	3.892512	10.819782
H	6.497132	3.529718	6.538572
H	5.840476	1.108900	10.499302
H	4.082548	1.147989	10.333500
H	4.926975	2.523778	11.042922
H	3.864234	4.106999	9.384020
H	2.946815	2.718405	8.767100
H	3.882607	3.734774	7.653908
H	5.117666	1.670373	6.793574
H	4.110653	0.715558	7.884132
H	5.869705	0.549289	7.936347
H	7.803086	4.413392	5.051321
H	7.816325	6.080766	4.481421
H	6.376176	5.440380	5.281702
H	10.076975	6.288488	7.389774
H	9.856357	6.673987	5.680425
H	9.951083	4.993617	6.191343
H	6.399600	7.348636	6.934882
H	7.696317	7.942274	5.885237
H	7.930043	7.902267	7.628438
H	7.366950	5.396043	12.060104
H	8.669056	6.558982	12.246442
H	9.031896	4.932719	11.654477
H	10.250950	6.280737	9.750664
H	9.657518	7.731170	10.558992
H	9.351705	7.512251	8.845048
H	6.749982	7.786006	9.301732
H	7.219530	8.099501	10.978047
H	6.036383	6.834141	10.605315
O	13.802395	4.500238	13.278290
C	13.201358	4.539639	12.204811

! **TS3** !

Spin multiplicity: 5

C	7.214493	5.150344	9.127347
C	6.580960	3.973751	9.631889
C	6.112011	3.154758	8.575888
C	6.541984	3.793369	7.387386
C	7.194143	5.031633	7.682722
Tm	8.792653	3.056418	8.575176
C	9.528925	0.562978	8.838205
C	10.721128	1.316733	8.756411
C	10.916958	1.829037	7.442479
C	9.756585	1.419316	6.668289
C	8.928287	0.662118	7.562187
C	12.260377	2.505596	7.075592
C	13.213504	2.507998	8.288144

C	9.479169	1.335784	5.151770
C	7.970841	1.176648	4.875112
C	9.125604	-0.305662	10.026400
C	7.907731	-1.173374	9.687814
C	4.989070	2.124773	8.678429
C	5.093762	1.025634	7.614279
C	7.603050	5.994722	6.546063
C	6.948701	7.381073	6.693977
C	7.522716	6.314216	10.094749
C	6.295500	7.255413	10.122391
O	9.981075	3.904225	10.152193
C	11.064361	4.008957	10.795993
C	11.856597	3.703906	11.774349
O	11.887699	2.618235	12.944464
Tm	12.816880	3.133131	15.045697
C	11.157077	5.151438	14.888656
C	12.283224	5.770747	15.506783
C	12.390357	5.188915	16.831059
C	11.305762	4.258296	16.946018
C	10.510710	4.262803	15.781872
C	13.080571	5.645157	18.137955
C	12.164231	6.694757	18.810995
C	12.979425	6.969539	14.818772
C	12.411492	7.179248	13.400495
C	9.093058	3.717315	15.617857
C	8.151271	4.927984	15.440013
C	12.977823	0.791679	16.348734
C	14.089657	1.562587	16.770606
C	15.045692	1.694857	15.716644
C	14.487472	1.008025	14.569977
C	13.217963	0.507135	14.984592
C	12.030043	0.008960	17.254026
C	10.764218	-0.434440	16.508015
C	16.421734	2.345876	15.973411
C	16.620853	3.637108	15.159199
C	15.088425	0.500294	13.238853
C	13.973785	0.098007	12.249325
C	11.639117	0.788306	18.515681
C	12.790660	-1.262056	17.698091
C	16.568883	2.730235	17.458513
C	17.578898	1.365530	15.696954
C	15.873867	-0.798951	13.539709
C	15.999315	1.486016	12.489682
C	13.214377	4.478322	19.137229
C	14.484916	6.245937	17.991454
C	14.502211	6.823418	14.641498
C	12.664605	8.267882	15.592494
C	8.633407	2.957794	16.868758

C	8.954606	2.806391	14.389412
C	13.001173	1.717359	5.977088
C	12.099361	3.976861	6.656153
C	10.162803	0.060462	4.606199
C	9.939466	2.547278	4.328629
C	10.300325	-1.246562	10.361928
C	8.825665	0.561529	11.262026
C	4.921447	1.488607	10.073331
C	3.667961	2.894387	8.444571
C	7.116932	5.457090	5.185199
C	9.130688	6.143611	6.445190
C	7.694710	5.798693	11.538460
C	8.789779	7.120953	9.771015
H	14.248376	1.895740	17.785710
H	12.564139	-0.082868	14.356997
H	12.520338	1.098265	19.086012
H	11.026441	0.161315	19.173490
H	11.063910	1.684241	18.273264
H	13.093022	-1.858783	16.831720
H	12.159266	-1.886281	18.342581
H	13.695286	-1.000384	18.256069
H	10.196285	0.418728	16.126803
H	10.107644	-1.002175	17.177021
H	11.004541	-1.082527	15.659241
H	15.808466	3.447007	17.778855
H	17.544956	3.202144	17.614661
H	16.513846	1.855086	18.114337
H	17.441432	0.433839	16.255729
H	18.525174	1.815137	16.020171
H	17.685119	1.115093	14.642031
H	16.465316	3.493407	14.090588
H	17.631907	4.033964	15.312973
H	15.917112	4.410844	15.484220
H	16.724605	-0.634823	14.202560
H	16.253021	-1.232117	12.605758
H	15.220877	-1.538391	14.014869
H	15.464185	2.403128	12.234027
H	16.342271	1.021133	11.557896
H	16.891639	1.758005	13.053935
H	13.408594	-0.770241	12.601941
H	14.430038	-0.187695	11.295074
H	13.274031	0.916738	12.069337
H	11.061761	3.728589	17.855147
H	10.815375	5.366428	13.885028
H	12.247705	4.068301	19.439863
H	13.709154	4.833233	20.047952
H	13.812914	3.662974	18.725850
H	15.174087	5.552142	17.501219

H	14.891259	6.465109	18.985514
H	14.489040	7.181897	17.432657
H	12.014958	7.572918	18.179566
H	12.600425	7.029105	19.760556
H	11.179013	6.266913	19.022089
H	15.026369	6.639329	15.577910
H	14.910810	7.748046	14.215321
H	14.725755	6.007470	13.951508
H	11.584507	8.384567	15.732372
H	13.026910	9.133908	15.025636
H	13.137598	8.303964	16.575270
H	12.548916	6.294700	12.774011
H	12.941519	8.012038	12.925253
H	11.347380	7.436431	13.418749
H	7.602295	2.611255	16.737497
H	8.657967	3.599433	17.755730
H	9.251998	2.079545	17.069351
H	9.576790	1.910386	14.479018
H	9.260278	3.320845	13.474642
H	7.913412	2.483004	14.269386
H	7.109352	4.594683	15.357321
H	8.397855	5.496044	14.538760
H	8.224932	5.608396	16.294749
H	11.409311	1.458495	9.578312
H	8.026639	0.148564	7.261664
H	13.130819	0.671664	6.274395
H	13.997694	2.147612	5.825906
H	12.493120	1.732641	5.012809
H	11.462474	4.103674	5.780244
H	13.077052	4.410938	6.416853
H	11.682220	4.569217	7.479522
H	12.816494	3.068353	9.139189
H	14.156481	2.982776	7.997580
H	13.446627	1.493360	8.624566
H	11.246559	0.085219	4.731404
H	9.947343	-0.057608	3.537392
H	9.789200	-0.826957	5.126719
H	9.458644	3.469716	4.668369
H	9.662113	2.403413	3.278520
H	11.017708	2.700056	4.355090
H	7.568767	0.238832	5.267205
H	7.796888	1.170014	3.793922
H	7.393609	1.999074	5.305434
H	10.563806	-1.866440	9.498821
H	10.026742	-1.911891	11.189070
H	11.190990	-0.688410	10.663090
H	9.693761	1.160227	11.554574
H	8.552195	-0.064400	12.119227

H	7.981633	1.242444	11.085352
H	7.029686	-0.572733	9.435927
H	7.642600	-1.800273	10.545802
H	8.117687	-1.836108	8.841714
H	6.399520	3.786759	10.681716
H	6.292471	3.456762	6.391579
H	5.848629	0.971682	10.334907
H	4.109079	0.754963	10.113051
H	4.722259	2.236576	10.847285
H	3.551571	3.702522	9.173562
H	2.809024	2.219572	8.540953
H	3.642450	3.338070	7.444319
H	5.129218	1.442874	6.603265
H	4.220817	0.365437	7.665591
H	5.986032	0.411867	7.757063
H	7.537368	4.475970	4.947724
H	7.429662	6.144509	4.392309
H	6.025626	5.380801	5.147816
H	9.585518	6.493495	7.373214
H	9.402502	6.851590	5.653232
H	9.590464	5.184950	6.180499
H	5.862603	7.289982	6.795462
H	7.153203	7.979114	5.798642
H	7.324898	7.941839	7.548984
H	6.771484	5.369723	11.938596
H	7.956641	6.641003	12.187000
H	8.489874	5.054273	11.610051
H	9.675704	6.479635	9.769270
H	8.939941	7.886638	10.539952
H	8.733659	7.641945	8.814823
H	6.090178	7.713215	9.154126
H	6.459338	8.060439	10.848608
H	5.399483	6.704607	10.426482
O	13.840814	4.006017	13.262385
C	13.079443	3.431390	12.402312

! **TS3**: Product (**iso3**) !

Spin multiplicity: 5

C	6.009893	4.810495	8.808260
C	5.975734	4.411342	10.278201
C	5.359029	3.110428	10.339290
C	4.754225	2.858041	9.087021
C	5.276374	3.832201	8.154758
Tm	7.664339	2.571942	9.128166
C	8.610448	1.282438	7.001415
C	7.750273	0.408235	7.746554
C	8.317168	0.088514	9.007158

C	9.540227	0.800554	9.048639
C	9.767133	1.517643	7.840242
C	7.918849	-1.028532	9.963835
C	8.730199	-2.286181	9.583383
C	11.111497	2.243290	7.610423
C	11.856780	1.644905	6.400505
C	8.319624	1.528932	5.506883
C	8.929863	0.365528	4.692572
C	6.123409	5.202978	11.580142
C	6.404564	4.249678	12.762828
C	3.575990	1.946077	8.809185
C	3.174155	1.149706	10.057617
C	6.477972	6.084662	8.096145
C	6.253441	5.953037	6.578475
O	8.937704	3.552367	10.537050
C	9.767888	3.659235	11.468936
C	10.564743	3.794099	12.388894
C	11.443816	3.897261	13.502721
O	12.235303	4.901632	13.625749
Tm	12.892902	4.211609	15.698147
C	12.057740	4.924679	18.117778
C	10.949579	4.283832	17.521730
C	10.545468	5.138421	16.469687
C	11.324298	6.331132	16.444386
C	12.309743	6.194067	17.501171
C	10.157799	3.087285	18.051148
C	10.738471	2.591120	19.382259
C	10.898364	7.515929	15.545343
C	11.988111	8.007307	14.574890
C	13.188167	7.208772	18.268395
C	14.339222	6.503601	19.013004
C	14.486277	2.121938	15.324524
C	14.844485	2.607970	16.602622
C	15.434823	3.875181	16.369257
C	15.517518	4.146777	14.968135
C	14.912243	3.014941	14.296589
C	15.011969	1.754842	17.857708
C	14.918458	2.576862	19.148761
C	16.272587	5.392386	14.453153
C	16.903679	6.163432	15.629290
C	14.925666	2.513355	12.835291
C	16.293794	1.837890	12.577500
C	10.397585	8.687646	16.415677
C	9.700977	7.116088	14.659054
C	13.841400	8.309054	17.420517
C	12.306770	7.873191	19.352380
C	10.107847	1.921164	17.050695
C	8.712214	3.558261	18.317725

C	16.427504	1.136326	17.787841
C	13.996147	0.605780	17.910154
C	17.455526	5.011979	13.541499
C	15.343469	6.381398	13.725770
C	14.676999	3.582480	11.759929
C	13.866384	1.409602	12.620549
O	11.446484	2.996831	14.412789
C	8.235935	-0.655105	11.419840
C	6.427276	-1.361153	9.850492
C	10.969037	3.769280	7.451957
C	12.048964	2.028912	8.816528
C	8.824762	2.863929	4.942669
C	6.801668	1.515037	5.239292
C	7.222860	6.275105	11.614130
C	4.752978	5.880525	11.848247
C	2.386289	2.845884	8.399294
C	3.875054	0.973280	7.654551
C	7.979497	6.364921	8.292938
C	5.626424	7.290695	8.552038
H	15.879100	4.490407	17.137663
H	14.020411	1.163461	15.140760
H	15.650036	3.390634	19.163548
H	15.118413	1.939790	20.017921
H	13.925451	3.014206	19.273209
H	16.546357	0.535259	16.880909
H	16.610234	0.489988	18.655272
H	17.194991	1.916727	17.775702
H	12.966365	0.972851	17.932459
H	14.155367	0.001999	18.810619
H	14.096036	-0.060856	17.047825
H	16.155880	6.503337	16.350754
H	17.414681	7.053379	15.246156
H	17.643879	5.557979	16.162530
H	18.113690	4.292806	14.040103
H	18.045724	5.907513	13.314328
H	17.142369	4.582436	12.590379
H	14.780955	5.920245	12.914664
H	15.921597	7.217750	13.313732
H	14.614979	6.808540	14.423517
H	17.129580	2.533981	12.661414
H	16.314516	1.404841	11.569673
H	16.461376	1.029251	13.296477
H	13.714235	4.078324	11.901596
H	14.675376	3.107318	10.772397
H	15.452411	4.348783	11.741294
H	14.106397	0.500562	13.180640
H	13.849832	1.132667	11.561061
H	12.862413	1.727592	12.907780

H	12.570003	4.571757	19.001135
H	9.729309	4.925125	15.793631
H	13.985235	5.790673	19.761846
H	14.941460	7.249660	19.543032
H	14.997281	5.969952	18.323585
H	14.466761	7.889768	16.626540
H	14.487133	8.925217	18.056724
H	13.115377	8.979043	16.960559
H	11.461198	8.412952	18.920896
H	12.897526	8.584488	19.943146
H	11.904117	7.117301	20.033971
H	12.903744	8.309921	15.081592
H	11.620582	8.878262	14.018271
H	12.230870	7.224215	13.852737
H	9.625348	8.350727	17.115694
H	9.959355	9.464129	15.777072
H	11.192290	9.157963	16.996219
H	9.945524	6.284755	13.993068
H	9.425781	7.971066	14.031306
H	8.822425	6.848639	15.255655
H	10.143748	1.752491	19.760907
H	10.725744	3.380571	20.141238
H	11.769093	2.240813	19.276486
H	11.103804	1.506903	16.861567
H	9.707596	2.233954	16.083600
H	9.480379	1.111178	17.441973
H	8.118435	2.741537	18.746009
H	8.220861	3.881651	17.394987
H	8.695487	4.398244	19.019956
H	10.222011	0.788844	9.887542
H	6.830201	-0.011892	7.363342
H	11.975438	0.562642	6.518826
H	12.857408	2.086217	6.325998
H	11.352209	1.824573	5.450724
H	10.329055	4.055120	6.617266
H	11.954907	4.220463	7.286308
H	10.555084	4.208735	8.365071
H	11.646520	2.450933	9.741653
H	13.001379	2.531959	8.619363
H	12.263446	0.968014	8.980573
H	10.014887	0.315138	4.804279
H	8.703251	0.482282	3.625676
H	8.518240	-0.592567	5.025489
H	8.419885	3.707229	5.512106
H	8.493830	2.970711	3.903101
H	9.910920	2.947289	4.942506
H	6.340864	0.551202	5.470356
H	6.612741	1.716478	4.179231

H	6.293130	2.289747	5.823829
H	8.524118	-2.587021	8.550787
H	8.476701	-3.125841	10.241829
H	9.804984	-2.098644	9.669692
H	9.303619	-0.467545	11.569335
H	7.948671	-1.469267	12.095095
H	7.693481	0.249272	11.718225
H	5.815279	-0.491357	10.110709
H	6.164465	-2.174796	10.535569
H	6.157519	-1.684388	8.839124
H	5.061146	2.632426	11.258491
H	5.045819	3.838531	7.098547
H	3.997234	0.532976	10.428313
H	2.339758	0.482752	9.818767
H	2.849324	1.810430	10.867574
H	2.151982	3.573522	9.182713
H	1.498059	2.226440	8.232848
H	2.590908	3.394759	7.475054
H	4.172791	1.499503	6.743515
H	2.976977	0.389536	7.424340
H	4.671256	0.275580	7.924811
H	6.774375	5.081565	6.170031
H	6.652420	6.841715	6.079838
H	5.191651	5.882640	6.320231
H	8.285547	6.378485	9.337253
H	8.234543	7.335449	7.851757
H	8.571356	5.597189	7.784704
H	4.560782	7.096231	8.390400
H	5.900802	8.171681	7.961838
H	5.768632	7.544674	9.602260
H	5.566595	3.580331	12.974787
H	6.568142	4.847158	13.664801
H	7.300367	3.647589	12.594727
H	8.212073	5.837740	11.467611
H	7.215886	6.747274	12.601395
H	7.076907	7.068739	10.882020
H	4.486518	6.598720	11.068979
H	4.795369	6.416483	12.803002
H	3.953547	5.135834	11.911741

! Trimerization Product **3** (Experimentally Observed) !

Spin multiplicity: 5

H	20,333581000	-0,986392000	-3,838057000
O	17,616175000	-0,526639000	-1,168977000
H	14,072786000	-0,461433000	4,012013000
C	15,961795000	-0,560053000	1,938225000
H	16,259832000	-1,302662000	2,675873000

H	15,274992000	-1,048022000	1,237863000
H	16,844733000	-0,273892000	1,360691000
C	16,566176000	-0,131114000	5,798987000
H	15,097651000	-0,657571000	7,329101000
C	17,721305000	-0,245540000	6,813144000
H	17,535965000	-1,093916000	7,480430000
H	18,677095000	-0,414984000	6,312639000
C	16,432668000	-1,514558000	5,148467000
H	17,304175000	-1,763008000	4,535886000
H	16,357067000	-2,276027000	5,932658000
H	15,541591000	-1,608891000	4,528215000
C	20,642251000	-1,419316000	3,027936000
C	21,986004000	-1,866641000	2,414894000
H	21,968808000	-2,942866000	2,206757000
H	22,191593000	-1,347409000	1,474052000
C	19,523729000	-1,692137000	2,012862000
H	19,655812000	-1,111302000	1,094656000
H	19,500036000	-2,750597000	1,729736000
H	18,542204000	-1,447948000	2,431260000
C	20,399235000	-2,252642000	4,292394000
H	19,467574000	-1,975955000	4,794340000
H	20,331825000	-3,314832000	4,034156000
C	17,890329000	0,617281000	-1,078657000
C	18,181198000	1,879800000	-1,121775000
C	18,543038000	2,770487000	-0,061312000
C	20,979349000	3,487721000	-2,783189000
C	20,587915000	2,535323000	-3,773873000
C	20,057414000	3,295679000	-4,885976000
C	20,158231000	4,672678000	-4,504747000
H	19,932360000	5,496090000	-5,165518000
C	20,781430000	4,810404000	-3,247693000
C	21,022289000	1,059945000	-3,633770000
H	20,767336000	1,008454000	-1,458945000
C	19,917155000	0,025828000	-3,904138000
H	19,462033000	0,128754000	-4,885313000
H	19,126013000	0,099070000	-3,157585000
C	19,884721000	3,006050000	-6,400456000
H	21,170811000	3,182144000	-8,160743000
C	19,504135000	1,579332000	-6,818715000
H	18,556186000	1,261585000	-6,378403000
H	19,383616000	1,552377000	-7,907803000
H	20,267652000	0,843064000	-6,568185000
C	18,816125000	3,919676000	-7,029593000
H	19,026855000	4,981745000	-6,891298000
H	18,770578000	3,738446000	-8,109361000
H	17,826950000	3,712879000	-6,614747000
C	20,825500000	6,442821000	-1,293368000
H	20,890282000	5,610120000	-0,588775000

H	21,356233000	7,301238000	-0,863599000
H	19,769877000	6,717657000	-1,377678000
H	20,189996000	7,500485000	-3,795632000
C	15,798581000	4,011378000	-2,123279000
H	15,475658000	3,424486000	-1,276509000
C	15,855793000	3,524353000	-3,447169000
C	16,360698000	4,593832000	-4,220763000
H	16,462526000	4,579770000	-5,296942000
C	16,536133000	5,766777000	-3,416086000
C	16,181493000	5,384406000	-2,064261000
C	15,230194000	2,236222000	-3,976083000
C	16,217296000	1,431425000	-4,831724000
H	17,093874000	1,122374000	-4,251584000
H	15,743947000	0,522545000	-5,221520000
H	16,562195000	2,017739000	-5,688710000
C	14,719640000	1,353071000	-2,830198000
H	13,955883000	1,871820000	-2,241465000
H	14,265385000	0,442003000	-3,234852000
H	15,525617000	1,053827000	-2,156223000
C	14,022010000	2,624044000	-4,854882000
H	14,330745000	3,225034000	-5,715979000
H	13,517652000	1,726090000	-5,231907000
H	13,295316000	3,208346000	-4,281191000
C	16,601919000	7,138003000	-4,129260000
C	17,384777000	7,058510000	-5,452823000
H	16,966033000	6,329538000	-6,149945000
H	17,352015000	8,033485000	-5,951445000
H	18,433394000	6,807200000	-5,281960000
C	17,235731000	8,291002000	-3,338500000
H	18,254742000	8,050410000	-3,021604000
H	17,292286000	9,179362000	-3,977921000
H	16,662975000	8,572984000	-2,455184000
C	15,151665000	7,528839000	-4,503638000
H	14,506568000	7,608719000	-3,626462000
H	15,139363000	8,494799000	-5,023551000
H	14,712811000	6,779033000	-5,169362000
C	15,956675000	6,192722000	-0,766068000
C	17,208685000	6,917913000	-0,243493000
H	17,636450000	7,607820000	-0,968822000
H	17,974535000	6,189321000	0,034276000
C	14,794176000	7,191297000	-0,943599000
H	13,896656000	6,679508000	-1,306987000
H	14,551839000	7,653270000	0,020984000
H	15,024879000	7,998449000	-1,639604000
O	18,797575000	3,976032000	-0,406321000
Tm	18,379869000	3,813520000	-2,676593000
C	16,777313000	1,064980000	4,841161000
C	17,311747000	2,274278000	5,393821000

H	17,741985000	2,349932000	6,381809000
C	17,045349000	3,375938000	4,551820000
C	16,403379000	2,834412000	3,413465000
H	16,074480000	3,415025000	2,563822000
C	16,190527000	1,432354000	3,564623000
C	15,293659000	0,676272000	2,559260000
C	13,957736000	0,283575000	3,223474000
H	13,469499000	1,161805000	3,658866000
H	13,281594000	-0,139004000	2,471385000
C	14,921128000	1,591070000	1,376462000
H	15,806062000	1,941976000	0,837659000
H	14,308183000	1,024534000	0,668281000
H	14,336754000	2,459029000	1,695996000
C	17,126008000	4,861421000	4,895404000
C	15,690493000	5,428168000	4,878460000
H	15,049189000	4,889910000	5,583860000
H	15,697166000	6,487205000	5,162018000
H	15,238116000	5,348396000	3,885742000
C	17,972279000	5,634604000	3,874174000
H	17,601759000	5,495331000	2,854022000
H	17,957881000	6,709241000	4,089949000
H	19,018092000	5,312911000	3,899852000
C	17,695969000	5,075445000	6,303182000
H	18,704479000	4,665459000	6,406813000
H	17,750103000	6,146071000	6,527440000
H	17,062289000	4,607745000	7,063899000
C	15,283403000	0,154490000	6,615692000
H	14,403460000	0,250980000	5,976106000
H	15,385813000	1,086002000	7,181120000
H	17,820175000	0,640139000	7,445328000
C	20,733816000	0,682955000	4,630458000
H	20,516823000	0,172944000	5,557396000
C	20,749265000	0,067545000	3,359419000
C	21,167881000	1,073745000	2,457736000
C	21,203610000	2,034371000	4,551173000
H	21,177734000	5,103835000	3,462790000
H	20,397769000	4,627456000	1,937032000
C	20,781002000	2,300594000	7,047992000
H	20,844694000	1,229176000	7,253530000
H	21,120027000	2,818197000	7,951612000
H	19,730872000	2,554935000	6,888574000
H	20,630580000	4,647358000	5,603356000
H	16,954352000	7,500403000	0,650738000
C	15,518931000	5,252425000	0,373027000
H	16,275018000	4,486852000	0,565140000
H	15,390620000	5,839861000	1,288918000
H	14,563952000	4,762279000	0,160785000
O	18,614934000	2,408764000	1,182758000

Tm	18,828509000	1,902510000	3,240501000
H	21,777216000	-0,288319000	-2,119134000
H	22,621783000	-0,208779000	-4,393491000
H	22,816108000	-1,663890000	3,099227000
H	21,217668000	-2,135476000	5,010400000
H	21,431616000	3,232268000	-1,835922000
C	21,530884000	0,775536000	-2,206347000
H	22,438252000	1,341123000	-1,972958000
C	22,225078000	0,798826000	-4,567059000
H	23,027647000	1,517533000	-4,370490000
H	21,965504000	0,870571000	-5,624530000
C	21,232805000	3,352999000	-7,078830000
H	22,051605000	2,744322000	-6,686561000
H	21,493001000	4,403061000	-6,914743000
C	21,411851000	6,074569000	-2,662702000
C	21,243031000	7,265303000	-3,616083000
H	21,711111000	8,158063000	-3,186792000
H	21,716492000	7,072419000	-4,584442000
C	22,924528000	5,815905000	-2,502493000
H	23,378152000	5,542131000	-3,460574000
H	23,428384000	6,715573000	-2,128885000
H	23,119836000	5,002721000	-1,797376000
H	21,276728000	0,931442000	1,391773000
C	21,488646000	2,278909000	3,149484000
C	22,168831000	3,444901000	2,399770000
C	23,602444000	3,677786000	2,918061000
H	24,182960000	2,750232000	2,876660000
H	24,106192000	4,417294000	2,285063000
H	23,637393000	4,051829000	3,941323000
C	21,359520000	4,752962000	2,447132000
H	21,899192000	5,548176000	1,920127000
C	22,323005000	3,095837000	0,908347000
H	21,354382000	2,922703000	0,433770000
H	22,797816000	3,937701000	0,394612000
H	22,956352000	2,216470000	0,756319000
C	21,655547000	2,731122000	5,854207000
C	23,093648000	2,255565000	6,168379000
H	23,799071000	2,523445000	5,379586000
H	23,445887000	2,704577000	7,104946000
H	23,121140000	1,167307000	6,282753000
C	21,628575000	4,265644000	5,836886000
H	21,900853000	4,646720000	6,827464000
H	22,329939000	4,699267000	5,125040000

! C13 Isotope (Trimerization Product **3-^{13}C**) !

Spin multiplicity: 5

H	20,294361895	-1,001099164	-3,828252224

O	17,630882164	-0,541346164	-1,080734014
H	14,072786000	-0,481042552	3,982598671
C	15,942185448	-0,530638671	1,952932164
H	16,225515283	-1,292857224	2,675873000
H	15,235772895	-0,989193343	1,252570164
H	16,830025836	-0,264087224	1,365593388
C	16,561273612	-0,131114000	5,794084612
H	15,102553388	-0,657571000	7,338905776
C	17,711500224	-0,245540000	6,803339224
H	17,540867388	-1,098818388	7,470625224
H	18,667290224	-0,400276836	6,297931836
C	16,432668000	-1,524362776	5,148467000
H	17,313979776	-1,767910388	4,540788388
H	16,361969388	-2,276027000	5,937560388
H	15,546493388	-1,633402941	4,523312612
C	20,642251000	-1,414413612	3,023033612
C	21,990906388	-1,871543388	2,414894000
H	21,963905612	-2,947768388	2,216561776
H	22,206300164	-1,357213776	1,469149612
C	19,518826612	-1,692137000	2,007959612
H	19,641104836	-1,121106776	1,084851224
H	19,500036000	-2,755499388	1,739540776
H	18,537301612	-1,457752776	2,431260000
C	20,399235000	-2,242837224	4,282589224
H	19,472476388	-1,961247836	4,784535224
H	20,331825000	-3,309929612	4,034156000
C	17,851109895	0,636890552	-1,323776406
C	18,181198000	1,894507164	-1,200213210
C	18,538135612	2,701853566	0,002419046
C	20,974446612	3,502428164	-2,783189000
C	20,592817388	2,530420612	-3,764068224
C	20,057414000	3,280971836	-4,876171224
C	20,153328612	4,662873224	-4,519454164
H	19,922555224	5,471578059	-5,199834717
C	20,776527612	4,820208776	-3,262400164
C	21,027191388	1,045237836	-3,638672388
H	20,762433612	1,028063552	-1,468749776
C	19,902447836	0,016023224	-3,899235612
H	19,437521059	0,123851612	-4,875508224
H	19,121110612	0,108874776	-3,147780224
C	19,884721000	3,010952388	-6,395553612
H	21,156103836	3,172339224	-8,160743000
C	19,509037388	1,594039164	-6,818715000
H	18,561088388	1,271389776	-6,373500612
H	19,383616000	1,567084164	-7,907803000
H	20,272554388	0,857771164	-6,573087388
C	18,816125000	3,919676000	-7,029593000
H	19,017050224	4,986647388	-6,906005164

H	18,780382776	3,723738836	-8,109361000
H	17,822047612	3,707976612	-6,624551776
C	20,820597612	6,447723388	-1,283563224
H	20,885379612	5,619924776	-0,574067836
H	21,346428224	7,311042776	-0,853794224
H	19,764974612	6,717657000	-1,377678000
H	20,185093612	7,480875448	-3,790729612
C	15,793678612	4,021182776	-2,108571836
H	15,470755612	3,448997941	-1,251997059
C	15,855793000	3,519450612	-3,427559448
C	16,355795612	4,579124836	-4,215860612
H	16,452721224	4,540550895	-5,292039612
C	16,536133000	5,766777000	-3,430793164
C	16,186395388	5,384406000	-2,074065776
C	15,235096388	2,246026776	-3,980985388
C	16,217296000	1,431425000	-4,826821612
H	17,084069224	1,112569224	-4,246681612
H	15,729239836	0,522545000	-5,206812836
H	16,571999776	1,998129448	-5,688710000
C	14,729444776	1,362875776	-2,840002776
H	13,970590164	1,881624776	-2,246367388
H	14,270287388	0,451807776	-3,244656776
H	15,535421776	1,058729388	-2,170930164
C	14,022010000	2,624044000	-4,854882000
H	14,316037836	3,220131612	-5,725783776
H	13,522554388	1,716285224	-5,222102224
H	13,290413612	3,203443612	-4,281191000
C	16,601919000	7,147807776	-4,129260000
C	17,384777000	7,058510000	-5,452823000
H	16,956228224	6,324635612	-6,135237836
H	17,356917388	8,028582612	-5,961249776
H	18,428491612	6,797395224	-5,277057612
C	17,240633388	8,300806776	-3,333597612
H	18,259644388	8,055312388	-3,021604000
H	17,297188388	9,189166776	-3,968116224
H	16,672779776	8,577886388	-2,445379224
C	15,146762612	7,528839000	-4,503638000
H	14,501665612	7,603816612	-3,631364388
H	15,124655836	8,489896612	-5,028453388
H	14,717713388	6,769228224	-5,169362000
C	15,956675000	6,178014836	-0,780775164
C	17,193977836	6,913010612	-0,248395388
H	17,616840448	7,612722388	-0,968822000
H	17,969632612	6,199125776	0,039178388
C	14,803980776	7,181492224	-0,938696612
H	13,896656000	6,684410388	-1,297182224
H	14,581253329	7,633660448	0,035691164
H	15,034683776	7,998449000	-1,624896836

O	18,782867836	3,882886626	-0,337687566
Tm	18,379869000	3,813520000	-2,676593000
C	16,801824941	1,064980000	4,850965776
C	17,306844612	2,298789941	5,388918612
H	17,737082612	2,413663046	6,372004224
C	17,025739448	3,371035612	4,527308059
C	16,383769448	2,819704836	3,398757836
H	16,035260895	3,375805895	2,539310059
C	16,215038941	1,422549224	3,574427776
C	15,308366164	0,700783941	2,583771941
C	13,967540776	0,283575000	3,208766836
H	13,444987059	1,142195448	3,649061224
H	13,330617881	-0,134101612	2,422361119
C	14,921128000	1,581265224	1,381364388
H	15,791354836	1,917464059	0,808244671
H	14,308183000	0,970607731	0,707500105
H	14,322046836	2,454126612	1,661679283
C	17,130910388	4,832006671	4,895404000
C	15,715004941	5,418363224	4,878460000
H	15,058993776	4,899714776	5,578957612
H	15,746189881	6,477400224	5,157115612
H	15,267530329	5,348396000	3,880839612
C	17,957571836	5,624799224	3,888881164
H	17,582149448	5,495331000	2,868729164
H	17,923564283	6,694533836	4,129168105
H	19,013189612	5,327618164	3,899852000
C	17,695969000	5,080347388	6,303182000
H	18,709381388	4,699775717	6,431324941
H	17,725591059	6,160778164	6,478416119
H	17,062289000	4,642061717	7,078606164
C	15,278500612	0,154490000	6,625496776
H	14,388752836	0,255882388	5,995715552
H	15,390715388	1,086002000	7,190924776
H	17,800565448	0,640139000	7,435523224
C	20,743620776	0,692759776	4,645165164
H	20,536432552	0,202358329	5,586810329
C	20,744362612	0,062642612	3,383930941
C	21,158076224	1,059037836	2,467540776
C	21,198707612	2,044175776	4,536465836
H	21,167929224	5,108737388	3,462790000
H	20,392866612	4,617651224	1,937032000
C	20,785904388	2,305496388	7,047992000
H	20,844694000	1,238980776	7,278041941
H	21,144538941	2,837806552	7,932002448
H	19,735774388	2,569642164	6,898378776
H	20,635482388	4,632650836	5,608258388
H	16,920035283	7,490598224	0,645835612
C	15,518931000	5,257327388	0,373027000

H	16,270115612	4,491754388	0,584749552
H	15,390620000	5,869275329	1,274210836
H	14,559049612	4,767181388	0,175492164
O	18,663957881	2,541128479	1,305317703
Tm	18,828509000	1,902510000	3,235598612
H	21,782118388	-0,283416612	-2,089719671
H	22,651197329	-0,203876612	-4,413100552
H	22,816108000	-1,668792388	3,104129388
H	21,217668000	-2,120768836	5,000595224
H	21,421811224	3,271487105	-1,826117224
C	21,530884000	0,775536000	-2,206347000
H	22,428447224	1,355830164	-1,982762776
C	22,234882776	0,798826000	-4,576863776
H	23,022744612	1,532240164	-4,365587612
H	21,980211164	0,880375776	-5,634334776
C	21,232805000	3,352999000	-7,078830000
H	22,046702612	2,739419612	-6,691463388
H	21,497903388	4,398158612	-6,924547776
C	21,416753388	6,079471388	-2,657799612
C	21,243031000	7,260400612	-3,616083000
H	21,706208612	8,162965388	-3,201499164
H	21,711589612	7,057711836	-4,584442000
C	22,934332776	5,815905000	-2,502493000
H	23,378152000	5,542131000	-3,465476388
H	23,443091164	6,715573000	-2,133787388
H	23,134543164	5,002721000	-1,797376000
H	21,266923224	0,892222895	1,401577776
C	21,483743612	2,278909000	3,134776836
C	22,173733388	3,454705776	2,399770000
C	23,617151164	3,677786000	2,922963388
H	24,187862388	2,745329612	2,876660000
H	24,125801552	4,417294000	2,294867776
H	23,652100164	4,046926612	3,946225388
C	21,354617612	4,757864388	2,447132000
H	21,884484836	5,562883164	1,915224612
C	22,323005000	3,095837000	0,913249388
H	21,344577224	2,917800612	0,453379552
H	22,788011224	3,927896224	0,379904836
H	22,951449612	2,211567612	0,766123776
C	21,650644612	2,716414836	5,839499836
C	23,088745612	2,260467388	6,168379000
H	23,799071000	2,538152164	5,389390776
H	23,416472671	2,729088941	7,104946000
H	23,135847164	1,172209388	6,297460164
C	21,628575000	4,246034448	5,841788388
H	21,900853000	4,607500895	6,837268776
H	22,334841388	4,684559836	5,139747164

VI. References

1 (a) M. N. Bochkarev and A. A. Fagin, *Chem.—Eur. J.*, 1999, **5**, 2990-2992; (b) W. J. Evans, N. T. Allen and J. W. Ziller, *J. Am. Chem. Soc.*, 2000, **122**, 11749-11750.

2 M. Reiners, N. Ehrlich and M. D. Walter, in *Inorganic Syntheses*, John Wiley & Sons, 2018, vol. 37, pp. 199-204.

3 F. Jaroschik, F. Nief and L. Ricard, *Chem. Commun.*, 2006, 426-428.

4 (a) J.-M. Lalancette, G. Rollin and P. Dumas, *Can. J. Chem.*, 1972, **50**, 3058-3062; (b) D. E. Bergbreiter and J. M. Killough, *J. Am. Chem. Soc.*, 1978, **100**, 2126-2134.

5 (a) G. Sheldrick, *Acta Crystallogr., Sect. A: Found. Adv.*, 2008, **64**, 112-122; (b) G. Sheldrick, *Acta Crystallogr., Sect. A: Found. Adv.*, 2015, **71**, 3-8.

6 G. Sheldrick, *Acta Crystallogr., Sect. C: Struct. Chem.*, 2015, **71**, 3-8.

7 O. V. Dolomanov, L. J. Bourhis, R. J. Gildea, J. A. K. Howard and H. Puschmann, *J. Appl. Crystallogr.*, 2009, **42**, 339-341.

8 A. Spek, *J. Appl. Crystallogr.*, 2003, **36**, 7-13.

9 G. Nocton and L. Ricard, *Dalton Trans.*, 2014, **43**, 4380-4387.

10 (a) A. D. Becke, *J. Chem. Phys.*, 1993, **98**, 5648-5652; (b) J. P. Perdew and Y. Wang, *Phys. Rev. B, Condens. Matter*, 1992, **45**, 13244-13249.

11 *Gaussian 09, Revision D.01*; M. J. Frisch, G. W. Trucks, H. B. Schlegel, G. E. Scuseria, M. A. Robb, J. R. Cheeseman, G. Scalmani, V. Barone, B. Mennucci, G. A. Petersson, H. Nakatsuji, M. Caricato, X. Li, H. P. Hratchian, A. F. Izmaylov, J. Bloino, G. Zheng, J. L. Sonnenberg, M. Hada, M. Ehara, K. Toyota, Fukuda, J. Hasegawa, M. Ishida, T. Nakajima, Y. Honda, O. Kitao, H. Nakai, T. Vreven, J. A. J. Montgomery, J. E. Peralta, F. Ogliaro, M. Bearpark, J. J. Heyd, E. Brothers, K. N. Kudin, V. N. Staroverov, R. Kobayashi, J. Normand, K. Raghavachari, A. Rendell, J. C. Burant, S. S. Iyengar, J. Tomasi, M. Cossi, N. Rega, J. M. Millam, M. Klene, J. E. Knox, J. B. Cross, V. Bakken, C. Adamo, J. Jaramillo, R. Gomperts, R. E. Stratmann, O. Yazyev, A. J. Austin, R. Cammi, C. Pomelli, J. W. Ochterski, R. L. Martin, K. Morokuma, V. G. Zakrzewski, G. A. Voth, P. Salvador, J. J. Dannenberg, S. Dapprich, A. D. Daniels, Ö. Farkas, J. B. Foresman, J. V. Ortiz, J. Cioslowski and D. J. Fox, Gaussian Inc., Wallingford CT, Ed. 2009.

12 (a) M. Dolg, U. Wedig, H. Stoll and H. Preuss, *J. Chem. Phys.*, 1987, **86**, 866-872; (b) D. Andrae, U. Häußermann, M. Dolg, H. Stoll and H. Preuß, *Theor. Chim. Acta*, 1990, **77**, 123-141; (c) M. Dolg, *Modern Methods and Algorithm of Quantum Chemistry*, John von Neuman Institute for Computing, Jülich (Germany), Grotendorst, J. edn., 2000, pp. 1-479.

13 A. W. Ehlers, M. Böhme, S. Dapprich, A. Gobbi, A. Höllwarth, V. Jonas, K. F. Köhler, R. Stegmann, A. Veldkamp and G. Frenking, *Chem. Phys. Lett.*, 1993, **208**, 111-114.

14 P. C. Hariharan and J. A. Pople, *Theor. Chim. Acta*, 1973, **28**, 213-222.

15 (a) A. D. McLean and G. S. Chandler, *J. Chem. Phys.*, 1980, **72**, 5639-5648; (b) R. Krishnan, J. S. Binkley, R. Seeger and J. A. Pople, *J. Chem. Phys.*, 1980, **72**, 650-654.

16 J.-D. Chai and M. Head-Gordon, *Phys. Chem. Chem. Phys.*, 2008, **10**, 6615-6620.

17 Y. Zhao, N. E. Schultz and D. G. Truhlar, *J. Chem. Phys.*, 2005, **123**, 161103.

18 A. E. Reed, L. A. Curtiss and F. Weinhold, *Chem. Rev.*, 1988, **88**, 899-926.

19 K. Yuvaraj, I. Douair, A. Paparo, L. Maron and C. Jones, *J. Am. Chem. Soc.*, 2019, **141**, 8764-8768.

20 D. McKay, A. S. P. Frey, J. C. Green, F. G. N. Cloke and L. Maron, *Chem. Commun.*, 2012, **48**, 4118-4120.
